# A novel approach to assessing quality issues and component annotation in TCM prescription: Insights from 100 common TCM products

**DOI:** 10.1016/j.jpha.2025.101332

**Published:** 2025-05-09

**Authors:** Huiting Ou, Chunxiang Liu, Saiyi Ye, Lin Yang, Qirui Bi, Wenlong Wei, Hua Qu, Yaling An, Jianqing Zhang, De-an Guo

**Affiliations:** a National Engineering Research Center of TCM Standardization Technology, Shanghai Institute of Materia Medica, Chinese Academy of Sciences, Shanghai, 201203, China; b University of Chinese Academy of Sciences, Beijing, 100049, China; c Shanghai University of Traditional Chinese Medicine, Shanghai, 201203, China

**Keywords:** Traditional Chinese medicine prescription, Quality consistency, Automated annotation, Large-scale products

## Abstract

The quality of traditional Chinese medicine (TCM) prescriptions (TCMPs) is critical to clinical efficacy; however, evaluating their consistency and identifying sources of variability remain challenging. This study proposes an integrated strategy to assess the quality of 100 widely sold TCMPs. A “one-for-all” chromatographic method was employed to analyze 645 sample batches. This large-scale data collection enabled statistical evaluations, such as hierarchical cluster analysis (HCA) and similarity heatmap, to identify quality inconsistencies. The introduction of a TCM-specific mass spectrometry (MS) database allowed for rapid, automated annotation of chemicals across 100 prescriptions and facilitated the tracing of raw material sources. Results indicate that 19% of prescriptions exhibited chemical inconsistencies, which are associated with high market value, low pricing, and substantial price disparities. The MS database allowed rapid annotation of 761 and 673 compounds in positive and negative modes, respectively, in 100 TCMPs, with 73 prescriptions reported for the first time. The tracing efforts succeeded in identifying >40% of the raw material sources for 51 prescriptions. P93 (Yinianjin (YNJ)) is a case in which the chromatographic profiles from three manufacturers displayed inconsistencies. Analysis using the database traced divergent peaks to *Rhei Radix* et *Rhizoma* (RRER). Verification with self-prepared samples confirmed that manufacturers utilized three distinct botanical sources. This integrated strategy provides a scalable framework for quality control in TCMPs.

## Highlights

• First large-scale quality consistency study on 100 TCM prescriptions (645 batches).
• 19% of prescriptions exhibited inconsistencies, associated with price disparities.
• Simultaneous annotation of chemicals in 100 prescriptions, 73 newly reported.
• Case study: TCM-PCDL for compound annotation and ingredient tracing.

## Graphical abstract

**Figure:**
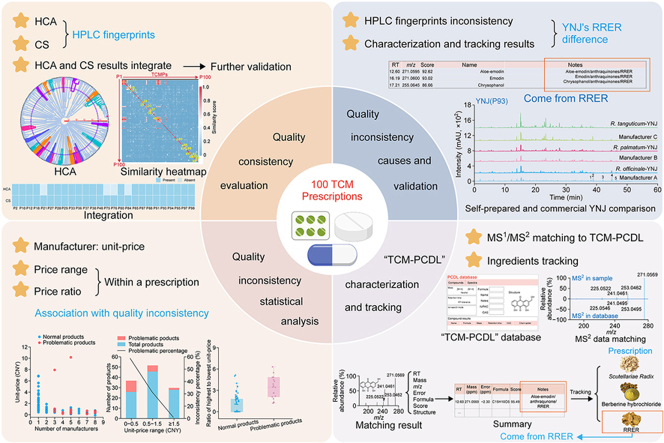

## 1 Introduction

The utilization of herbs in healthcare management has been practiced for centuries. Traditional Chinese medicine (TCM) prescriptions (TCMPs) contain medicinal herbs based on TCM theories for disease prevention and treatment. These formulations are available in various forms, such as capsules, granules, tablets, pills, and oral liquids, facilitating their transport and use. TCMPs are widely utilized in clinical practice [1,2]. According to the “2022 National Blue Book on the Regulation of Traditional Chinese Medicine”, by the end of 2022, China had 2,319 TCM production enterprises, offering approximately 9,000 different TCMPs and approximately 57,000 valid approval numbers [3]. Notably, many TCMPs are produced by multiple manufacturers. For instance, Liuwei Dihuang Wan has 651 manufacturers with approval numbers, and products from at least 349 different manufacturers are available in the market. Prices for Liuwei Dihuang Wan vary significantly, ranging from Chinese Yuan (CNY) 4.7 to 31 per bottle, indicating a price difference of more than sixfold [4]. As the TCM market continues to expand, concerns regarding product quality have become more pronounced. This raises several critical questions. How consistent is the quality of TCMPs across different manufacturers? Does a higher price necessarily indicate superior quality? Are lower-priced products produced in strict adherence to the prescribed formulations?

TCMPs often comprise several to dozens of medicinal ingredients, resulting in complex compositions that present notable challenges during quality assessment [5]. Chromatographic methods (e.g., high performance liquid chromatography-ultraviolet/evaporative light scattering detection (HPLC-UV/ELSD), capillary electrophoresis (CE), and gas chromatography (GC)) and spectroscopic techniques (e.g., infrared (IR) spectroscopy, UV-visible (UV-vis) spectroscopy, and nuclear magnetic resonance (NMR)) are widely employed to assess quality consistency [[6], [7], [8], [9], [10], [11]]. With technological advancements, more sophisticated methods are increasingly utilized. For example, Liu et al. [12] applied HPLC-charged aerosol detection (HPLC-CAD) to evaluate the quality consistency of 19 batches of Shengmai formula. Moreover, mass spectrometry (MS) techniques such as liquid chromatography-tandem MS (LC-MS/MS) and GC-MS/MS exhibit significant potential, with research expanding from small active molecules to macromolecules such as polysaccharides and peptides [13]. Given the inherent complexity of TCM formulations, combining multiple techniques is common [14]. For instance, Zhao et al. [15] employed HPLC-diode array detection (HPLC-DAD) and GC-MS to comprehensively assess the quality of 75 batches of Danshen tablets from 15 manufacturers based on content and dissolution rates. However, the current research predominantly focuses on individual varieties, particularly high-demand products such as Danshen tablet, Liuwei Dihuang Wan, and Fufang Gancao tablet. There is a notable lack of studies that conducted horizontal comparisons across multiple varieties, manufacturers, and batches of TCMPs available in the market.

The traceability of medicinal ingredients in TCMPs is a critical approach to address quality issues. This process typically relies on chemical annotation. Significant progress has been made in annotating the chemicals of individual medicinal herbs, with LC-high resolution MS (LC-HRMS) capable of identifying hundreds of compounds from a single herb. Advances in chromatographic separation techniques (e.g., offline two-dimensional (2D) and 3D methods) and data acquisition methods (e.g., parent ion lists, dynamic exclusion, and mass tagging) have further expanded the scope of chemical annotation. For instance, Li et al. [16] annotated more than 1,500 compounds from ginseng and related species, whereas Pan et al. [17] annotated more than 1,227 compounds across five *Uncariae Ramulus* species. However, annotating components in TCMPs is challenging owing to their complex and diverse compositions, resulting in lower identification capabilities compared with single herbs. Recent studies, such as that of Hu et al. [18], have adopted hybrid strategies to identify 294 compounds in Xuebijing injection. Despite these advancements, current techniques still face notable limitations primarily owing to their time-intensive nature, i.e., component annotation for a single prescription can take over a month. To address this, database-based annotation has gained popularity, substantially reducing the processing time. The common MS databases are UNIFI, Compound Discovery, MassBank, MoNA, Global Natural Product Social Molecular Networking (GNPS), MZedDB, mzCloud, and METLIN. Among these, MassBank and GNPS are widely used because they support simultaneous library searching and MS/MS spectral matching with reference compound spectra. However, the relevance of MassBank to TCM is limited because it requires manual input of *m*/*z* values and response intensities, whereas GNPS primarily provides compound clustering visualizations and necessitates standardized analytical workflows [19]. To overcome these challenges, a dedicated database tailored to TCM, constructed using authentic reference compounds specific to TCM ingredients, holds notable promise to improve component annotation and quality traceability.

This study proposes a novel strategy for the early and rapid detection of quality fluctuations in large-scale TCMPs and quality traceability to predict potential adverse drug events and enhance the safety of clinical medication. To horizontally compare across different prescriptions, we developed a universal chromatographic method with a 50-min runtime that is suitable for large-scale quality screening of diverse samples. Meanwhile, Traditional Chinese Medicine Personal Compound Database and Library (TCM-PCDL) database was employed to perform rapid and intelligent chemical annotation, facilitating high-quality traceability of medicinal ingredients. To ensure the relevance and representativeness, the study focused on 100 high-sales prescriptions frequently associated with quality issues in the market. Samples were collected from multiple manufacturers and batches (5–10 batches per prescription), resulting in 645 batches being analyzed.

## 2 Materials and methods

### 2.1 Chemicals and reagents

HPLC-grade acetonitrile (Honeywell, Charlotte, NC, USA), phosphoric acid (Tedia, Fairfield, OH, USA), and LC-MS grade formic acid (ROE Scientific Inc., Newark, NJ, USA) were used in the mobile phase. Methanol (Sinopharm Chemical Reagent Co., Ltd., Beijing, China) was the extraction solvent. Six reference standards, namely, baicalin, aloe-emodin, rhein, emodin, chrysophanol, and physcion, with purity of >98%, were obtained from Standard Technology Co., Ltd. (Shanghai, China). Three reference materials, namely, *Rheum palmatum* L. (*R. palmatum*), *Rheum tanguticum* Maxim. ex Balf. (*R*. *tanguticum*), and *Rheum officinale* Baill. (*R. officinale*), were obtained from the National Institute for Food and Drug Control (Beijing, China).

### 2.2 TCM prescriptions

This study aimed to collect TCMPs with annual sales exceeding CNY 100 million from pharmacies, online platforms, and secondary and tertiary hospitals across China. Sales data were obtained from three authoritative databases: Menet, Drugdataexpy, and Sinohealth. In addition, prescriptions flagged as inconsistent in national drug sampling reports were included. A total of 100 widely used TCMPs were selected. Samples were obtained through online and offline channels, with each batch comprising one box. To ensure representativeness, at least five batches were collected for each prescription. For difficult-to-source prescriptions, adjustments were made, and for those with poor consistency, the number of batches was increased from 6 to 10. In total, 645 sample batches were collected. Detailed information, including serial numbers, prescription names, ingredients, manufacturers, dosage forms, and batch numbers, is provided in Table S1.

### 2.3 Sample preparation

Different dosage forms require specific pretreatment methods. For tablets and small pills, the coating was removed and the material was ground into a uniform powder. Granules were similarly ground into a powder. For the patch, a section was cut into small fragments and the capsule contents were thoroughly mixed. For big honeyed pills, an equivalent amount of diatomite was measured and finely ground with the pills into a flocculent mixture. For HPLC analysis, approximately 0.5 g of the prepared powder or fragments was weighed and combined with 25 mL of 70% methanol (*v*/*v*). The mixture underwent ultrasonic treatment (1,130 W, 37 kHz) for 45 min and centrifuged (14,000 rpm, 10 min) at room temperature. The supernatant (1.5 mL) was used as a test solution. Liquid formulations were handled differently, where 1 mL of the sample was diluted with 25 mL of 70% methanol and directly injected. In total, 645 samples were generated. For LC-HRMS analysis, 0.02 g or 100 μL of respective sample of each TCMP was subjected to ultrasonic processing for 30 min with 2 mL of 70% methanol, followed by the same procedure as used for HPLC. The standards were dissolved in methanol with a concentration of approximately 0.1 mg/mL for analysis.

### 2.4 Chromatographic systems and MS parameters

For quality evaluation, chromatographic separation was conducted on an Agilent 1260 system (Agilent Technologies, Santa Clara, CA, USA ) equipped with an XSelect HSS T_3_ column (2.7 μm, 4.6 mm × 150 mm; Waters Corporation, Milford, MA, USA). The mobile phase comprised 0.05% phosphoric acid in water (A) and acetonitrile (B), with gradient elution (0–30 min, 0%–30% B and 30–50 min, 30%–100% B) at a flow rate of 0.8 mL/min. The injection volume was 5 μL, the column temperature was 30 °C, and detection was conducted at 230 nm.

For metabolite annotation, Agilent 1290 UPLC system (Agilent Technologies) was used for the separation procedure. A 28-min chromatographic gradient based on a ACQUITY HSS UPLC T_3_ column (1.8 μm, 2.1 mm × 100 mm; Waters Corporation) at 30 °C was used. The mobile phase comprised 0.1% formic acid in water (A) and acetonitrile (B), with a gradient program as follows: 0–10 min, 0%–30% (B); 10–25 min, 30%–100% (B); and 25–28 min, 100% (B), at a flow rate of 0.25 mL/min. Sample (1 μL) were injected for analysis. MS detection was conducted on an Agilent 6530 Q-TOF HRMS (Agilent Technologies) equipped with an electrospray ionization (ESI) source operating in positive/negative ion mode. Data were acquired in ddMS^2^ mode (MS^1^, *m/z* 100–1200; MS^2^, *m/z* 50–1200), selecting two precursor ions with collision energies of 10, 20, and 40 eV. Other parameters were applied as described in our previous study [20].

## 3 Results and discussion

### 3.1 Selection of 100 TCMPs

**Fig. 1:**
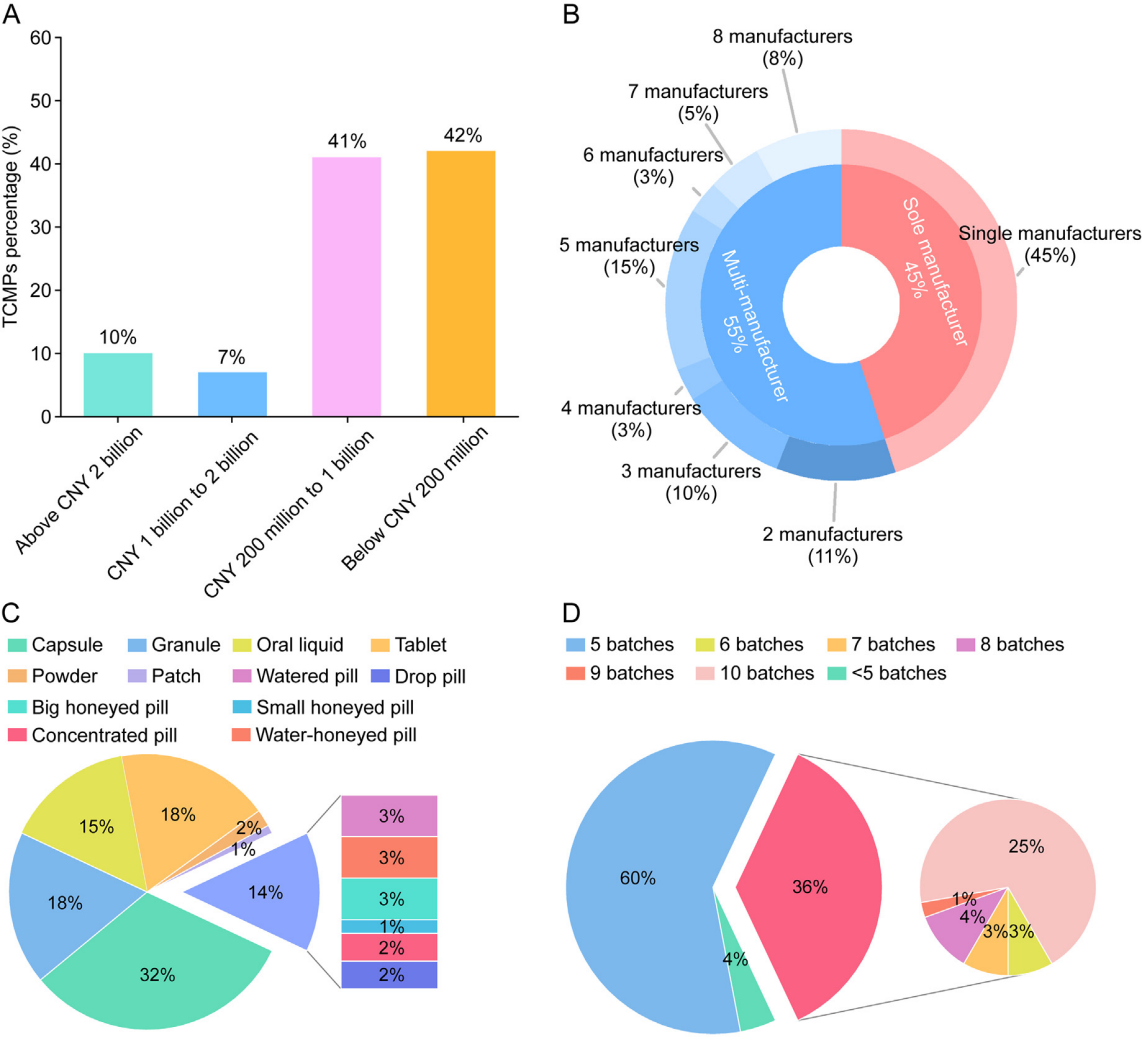
Sample distribution of 100 traditional Chinese medicine (TCM) prescriptions (TCMPs) (645 batches) according to (A) annual sales, (B) number of manufacturers, (C) dosage forms, and (D) batch numbers. CNY: Chinese Yuan.

This study evaluated 100 TCMPs based on annual sales, number of manufacturers, and dosage forms. Fig. 1A presents the distribution of annual sales volumes: 10% of the selected varieties had sales exceeding CNY 2 billion, 7% ranged between CNY 1 and 2 billion, 41% fell between CNY 200 million and 1 billion, and 42% were below CNY 200 million. This selection strategy ensured the inclusion of major TCMPs while capturing a wide range of sales. To evaluate the quality of products from single vs. multiple manufacturers, 45 TCMPs were produced by single manufacturer, whereas 55 were produced by multiple manufacturers. Among the latter, 39% was produced by two to five manufacturers and 16% by more than five manufacturers (Fig. 1B). The samples represented a wide range of dosage forms, with capsules being the most common (32%). Powders and patches were less prevalent, whereas granules, oral liquids, tablets, and pills accounted for 14%–18%. Moreover, pills were categorized into subtypes: small honeyed pills, big honeyed pills, concentrated pills, water- honeyed pills, drop pills, and watered pills (Fig. 1C). To enhance the representativeness of the sample set, a minimum of five batches was collected for each TCMP, resulting in a 96% coverage rate, with only a few difficult-to-source prescriptions as exceptions. For TCMPs exhibiting notable differences in fingerprint profiles, additional one to five batches were acquired, with 25% reaching a total of 10 batches (Fig. 1D). Overall, 645 batches were collected. Detailed information is provided in Table S1.

### 3.2 Quality consistency evaluation of 100 TCMPs

To assess the quality of 100 TCMPs, an “one-for-all” HPLC separation method was employed to analyze 645 batch samples. Subsequently, multivariate statistical analysis and cosine similarity (CS) calculations were conducted, facilitating the screening of prescriptions with quality variations.

**Fig. 2:**
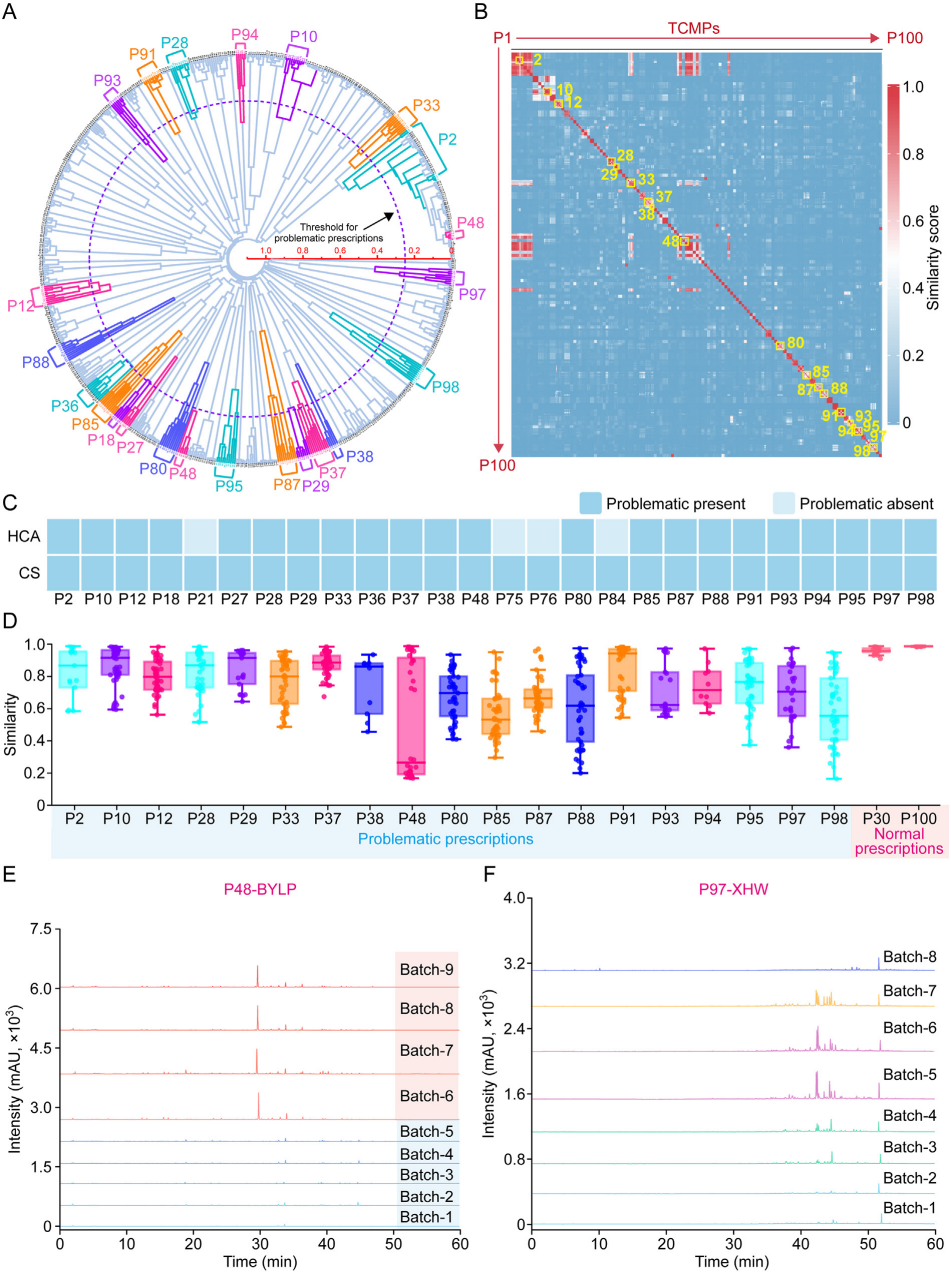
Quality consistency analysis of 100 traditional Chinese medicine (TCM) prescriptions (TCMPs). (A, B) Circular hierarchical cluster analysis (HCA) cluster map (A) and similarity heatmap (B) of 645 batch fingerprint data for identifying potential quality issues. (C) Products with quality inconsistencies detected via HCA and cosine similarity (CS). (D) Intraproduct similarity comparison, showing significant variations in inconsistent products. (E, F) Fingerprint spectra of inconsistent products P48-Biyanling tablet (BYLP) (E) and P97-Xihuang pill (XHW) (F) from different manufacturers.

Retention time calibration was conducted to HPLC fingerprint data from 645 batches, aligning 898 time points to construct a data matrix of 898 × 645. This matrix was processed using OriginPro 2022 for hierarchical cluster analysis (HCA), generating a dendrogram, wherein short clustering distances indicate high similarity between data points. Therefore, most TCMPs grouped into a single cluster, with even those containing the same medicinal materials being grouped together (Fig. 2A). For instance, P4 and P52 were grouped together owing to their shared ingredient, such as *Scutellariae Radix* and *Asari Radix* et *Rhizoma*. A clustering distance threshold of 0.25 was set, beyond which samples exhibited low similarity. For instance, P48 exhibited the largest clustering distance, with its nine batches divided into two primary clusters. Overall, HCA identified 22 TCMPs below the threshold, highlighting these as products with potentially inconsistent quality. To provide an intuitive representation of the proximity among each prescription, CS was calculated across 645 sample batches, and Figs. S1–S5 presents the detailed results. A similarity score of >0.9 generally indicates high-quality consistency. However, owing to variations in raw material sources and manufacturing processes, the threshold was relaxed to 0.7 for samples from multiple manufacturers. TCMPs with similarity scores of <0.9 (single manufacturer) or 0.7 (multiple manufacturers) were classified as potential quality issues. Fig. 2B presents a similarity heatmap of the 645 batches. Red squares along the diagonal indicate high similarity between batches within the same TCMPs. By contrast, white squares signal notable intravariety variations, observed in TCMPs such as P37, P48, P85, and P87. Notably, P48 exhibits two distinct red squares, consistent with HCA clustering results. Overall, the similarity heatmap identified 23 TCMPs with similarities below 0.7, which are potential quality concerns.

Fig. 2C presents screening results from the HCA and CS heatmap. By integrating both methods, 26 TCMPs were flagged for further validation owing to low response levels or possible excipient interference. P18, P27, P75, and P76, containing elevated levels of saponins and ginsenosides from *Panax notoginseng*, exhibited strong responses at 210 nm. Adjusting the absorption wavelength to 210 nm increased their similarity values to 0.98, indicating that there were no quality issues. Similarly, P21 exhibited high consistency at its maximum absorption wavelength (254 nm). For P36 and P84, after removing excipient peaks at 8 and 4 min, respectively, and recalculating, similarity rose to 0.74 and 0.71, both exceeding the 0.70 threshold; thus, they were categorized as nonproblematic. Ultimately, 19 TCMPs (19% of total prescriptions) were marked as potential quality concerns.

Fig. 2D presents batch-to-batch similarities for problematic TCMPs. These prescriptions exhibited higher variability compared with consistent prescriptions, such as P30 and P100, with median similarity values notably diverging from 1. Some problematic products exhibited distinct clustering of similarity scores into two or more groups, whereas others displayed a more continuous distribution. For instance, P48 reveals two similarity clusters, indicating two predominant fingerprint profile types (Fig. 2E), whereas P97 displays a continuous distribution (Fig. 2F).

### 3.3 Statistical analysis of quality inconsistencies in TCMPs

**Fig. 3:**
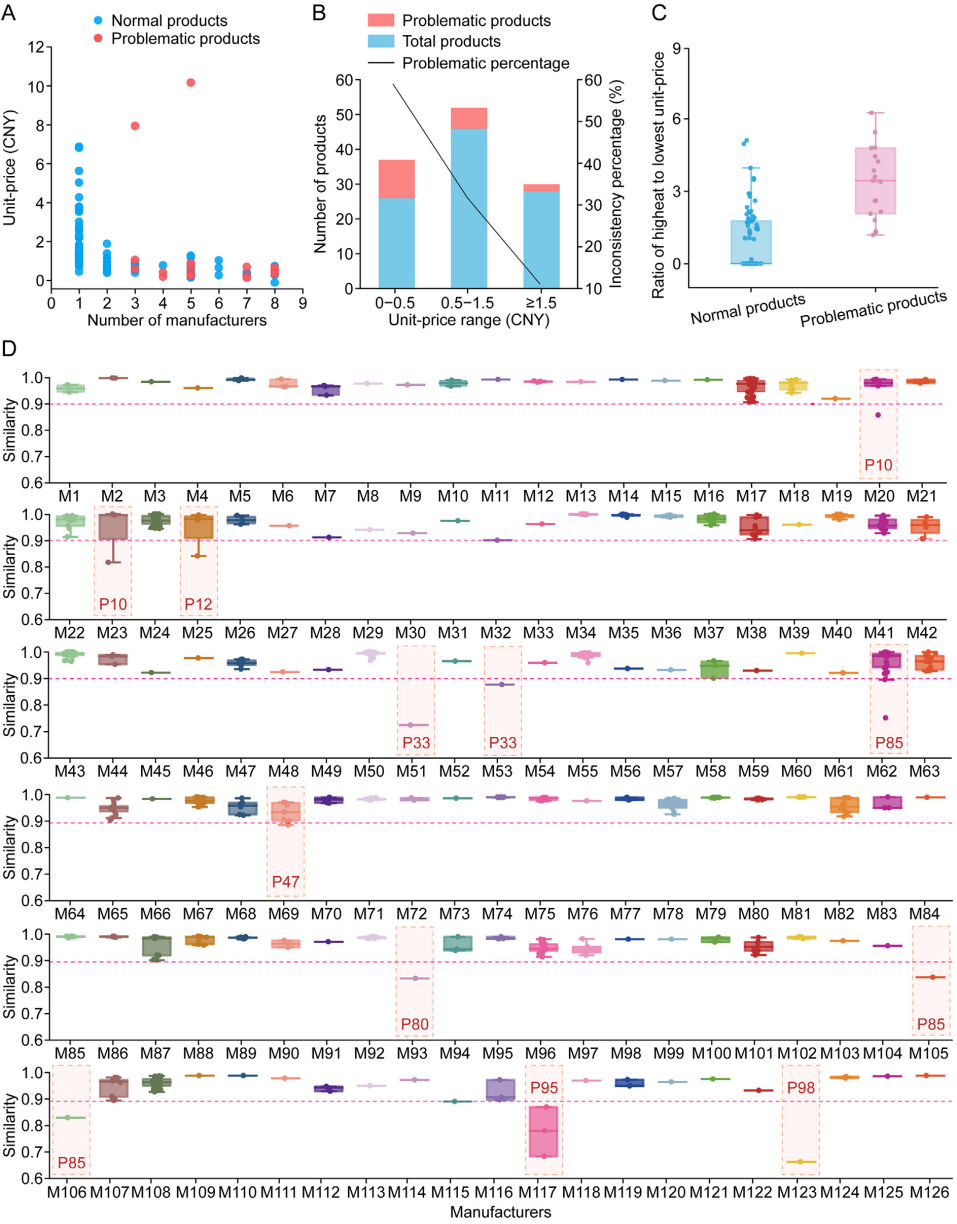
Statistical analysis of quality inconsistencies in 100 traditional Chinese medicine (TCM) prescriptions (TCMPs). (A) Relationship between problematic products and unit price, manufacturer (M) number, where red dots signify problematic prescriptions. (B) Percentage of quality inconsistencies across different unit price ranges. Products priced below Chinese Yuan (CNY) 2 and produced by multiple manufacturers are prone to quality issues. (C) Ratio of the highest to lowest unit price in normal vs. problematic products, showing that significant price variations correlate with high quality issues. (D) Intraproduct similarity of 126 TCMPs across manufacturers (batches ≥ 2), with 12 manufacturers exhibiting interbatch similarities below 0.9.

A statistical analysis was conducted on TCMPs with inconsistent quality. These prescriptions, produced by various manufacturers, vary in price. Data on the unit prices (per dose in CNY) were sourced from www.yaofangwang.com. Fig. 3A presents the manufacturers vs. unit prices. The results indicate that problematic TCMPs are predominantly found among those produced by multiple manufacturers and priced below CNY 2. In particular, 57.89% of problematic prescriptions was priced under CNY 0.5, 31.58% fell within CNY 0.5–1.5, and 10.53% exceeded CNY 1.5 (Fig. 3B). The probability of inconsistencies was generally high for low-priced products owing to limited funds for high-quality materials. However, this was not an absolute rule. For instance, the lowest-priced product, P3 (CNY 0.13), which was sourced from five manufacturers, maintained a high similarity (0.98).

Quality issues were particularly evident in prescriptions containing costly medicinal ingredients, such as P94 and P97, which had unit prices exceeding CNY 7. Among them, P97 contained valuable medicinal ingredients such as *Bovis Calculus*, *Moschus*, and vinegar-processed *Olibanum*/*Myrrha*. This study analyzed eight batches of P97, revealing profile variations among different manufacturers, whereas batch-to-batch consistency within the same manufacturer remained high. This suggests that the differences may be attributed to fluctuations in raw material quality. This finding is consistent with Xu [21]'s study, which reported that five out of 20 samples showed evidence of rosin adulteration.

Price variations were associated with quality consistency. Normal prescriptions exhibited a median price ratio close to 0, while problematic prescriptions had a median price ratio of approximately 3 (Fig. 3C). Price ratios for 11 prescriptions exceed 3, accounting for 57.89% of the total problematic prescriptions. This relationship underscores that substantial pricing variations were linked to a high incidence of quality issues, which can be attributed to variations in raw material quality. Taking P95 (Fengshiantai tablet (FSATP)) as an example, the price fluctuated between CNY 0.13 and 0.82 per dose, a difference of more than sixfold. There were significant profile differences among the four manufacturers. Given that P95 contained toxic medicinal ingredients such as *Aconiti Radix*, *Aconiti Kusnezoffii Radix*, and *Strychni Semen*, whose quality fluctuated considerably, it was speculated that this variability mainly stems from raw materials. This finding was consistent with Zhang et al. [22]'s research, which showed that one out of three manufacturers had poor raw material quality.

A total of 126 manufacturers produced the same prescriptions across two or more batches. Fig. 3D presents the interbatch similarities. For prescriptions from the same manufacturer, a similarity threshold of 0.90 was established. Twelve manufacturers exhibited interbatch similarities below this threshold, in which nine TCMPs were identified as problematic, accounting for 47.37% of the total problematic prescriptions. This indicated potential issues with GMP compliance or corporate standards. For instance, P33, produced by manufacturer 53, exhibited batch-to-batch variations, which may attributed to inconsistencies in production process. In 2016, the China Food and Drug Administration disclosed that manufacturer 53 had engaged in noncompliant manufacturing practices for another TCMP [23].

From a dosage form perspective, problematic TCMPs were prevalent among granules, oral liquids, tablets, and powders, as depicted in Fig. S6. Granules had the highest proportion of problematic prescriptions at 33.33%. This product type is commonly produced by multiple manufacturers, resulting in considerable differences in profiles and peak responses, as observed in P2, P33, P37, P38, P80, and P87.

### 3.4 Automated metabolite annotation using TCM-PCDL enhances quality tracking

The chemical annotation of TCMPs presents a greater challenge than single herbal materials. Research has traditionally focused on metabolite annotation of individual herbs. This study represents the first attempt to simultaneously annotate the chemical components of 100 TCMPs in positive and negative modes. This endeavor posed notable challenges because comprehensive chemical profiling for the majority of TCMPs has yet to be performed. To facilitate rapid annotation, we attempted to introduce Agilent-Nature Standard HRMS database (TCM-PCDL). This comprehensive database, constructed with authentic reference standards and HRMS, contains 2,565 common TCM compounds. It features MS^1^ acquired under different modes and MS^2^ at various collision energies (10, 20, and 40 eV) for these compounds.

**Fig. 4:**
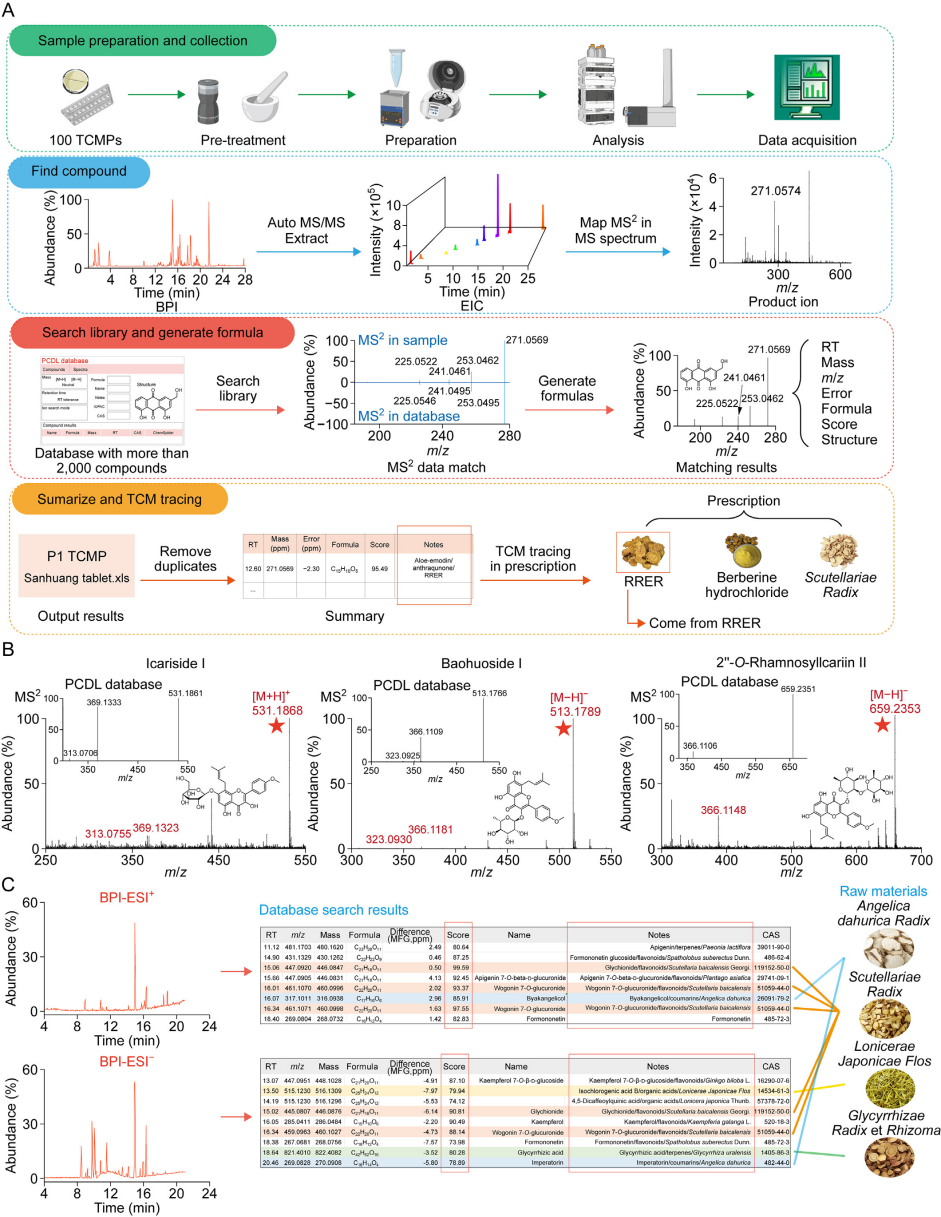
Metabolite annotation workflow and result presentation in Traditional Chinese Medicine Personal Compound Database and Library (TCM-PCDL) database. (A) Workflow for automated metabolite annotation. (B) Illustration of MS^2^ spectra matching with reference spectra in TCM-PCDL database, using three expensive reference standards as examples. The insets indicate the mass spectrometry (MS) spectra in PCDL database. (C) Tracking medicinal ingredients using the TCM-PCDL database, with P6 as a case study. BPI: base peak intensity; EIC: extracted ion chromatogram; RT: retention time; IUPAC: International Union of Pure and Applied Chemistry; CAS: Chemical Abstracts Service; RRER: *Rhei Radix* et *Rhizoma*; ESI: electrospray ionization; MFG: mass fine global.

Fig. 4A presents the workflow. Initially, samples underwent pretreatment, such as sugar coating removal and grinding. Subsequently, solvent extraction was conducted, followed by LC-MS analysis in both positive and negative modes, yielding 200 total ion chromatogram (TIC) spectra from 100 TCMPs. The subsequent step employed the auto MS/MS extract function to extract TIC data, linking primary sample spectrum information with secondary fragment data to obtain compound-specific mass spectral fragments. The third and crucial step was database searching, where the sample's MS data was matched to database entries, focusing on aligning MS^2^ spectra with reference standards. This matching generated detailed output, including molecular formulas, masses, mass deviations, overall scores, Chemical Abstracts Service (CAS) numbers, structural formulas, and comparative MS^2^ diagrams. The overall scores and MS^2^ comparison diagrams are important criteria for assessing annotation accuracy. High scores indicated notable confidence in the results. In the final step, a filtering criterion was applied to refine the matches. Annotation results with peak intensities of >10,000 and overall scores of >70 were deemed reliable, and compounds with retention times within 0.2 min were classified as the same.

The database, enriched with multi-stage MS data from authenticated standards, notably minimized the reliance on costly reference compounds. For instance, icariside I, baohuoside I, and 2″-*O*-rhamnosyl-icariin II were essential quality markers for *Epimedii Folium*. Conventionally, these compounds need to be purchased for manual metabolite annotation, with each costing over CNY 1,000, thereby imposing a financial burden on *Epimedii Folium* research. By employing TCM-PCDL database, we successfully identified these markers within samples P12, P29, and P89, with their MS data matching scores > 70 (Fig. 4B).

The TCM-PCDL database not only facilitated chemical identification but also supported raw material tracing. For instance, in sample P6, which included 14 medicinal ingredients, TIC data were matched against the database, and compounds with scores exceeding 70 were identified. This analysis revealed 21 and 20 compounds in positive and negative modes, respectively. Key flavonoids, such as wogonin 7-*O*-glucuronide (score 93), were traced to *Scutellariae Radix*. Coumarin compounds, such as byakangelicol (score 85) and imperatorin (score 78), were linked to Angelicae Dahuricae Radix. Glycyrrhizin (score 80) was traced to *Glycyrrhizae Radix* et *Rhizome* (Fig. 4C). These distinctive components facilitated raw material tracking, for instance, precisely identifying Scutellariae Radix in 23 TCMPs, aligning with product specifications. Additionally, the TCM-PCDL database proved invaluable for tracking high-value medicinal materials. For example, 11-keto-β-boswellic acid (score 99) was detected in six prescriptions, verifying the inclusion of vinegar-processed Olibanum as declared in the product specifications.

### 3.5 Metabolite annotation results across 100 TCMPs

**Fig. 5:**
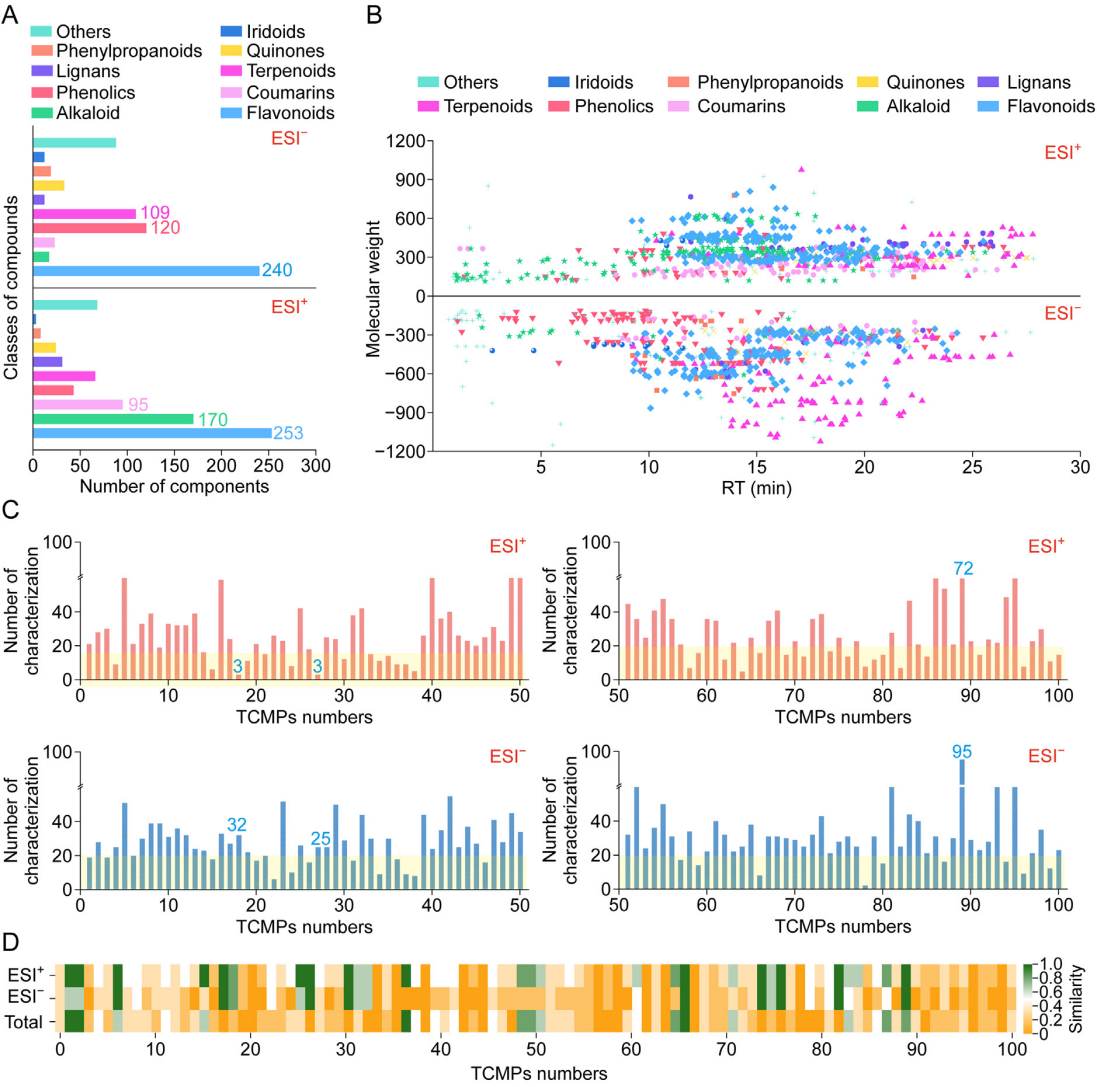
Summary of metabolite annotation results of 100 traditional Chinese medicine (TCM) prescriptions (TCMPs). (A) Distribution of different components annotated in negative (up) and positive (down) modes. (B) Two-dimensional (2D) scatter plot of retention time (RT) vs. molecular weight for compounds annotated in postive (up) and negative (down) modes. (C) Number of characterization annotated per TCMPs. (D) Number of medicinal ingredients traced across 100 TCMPs. ESI: electrospray ionization; RT: retention time.

The automated annotation using TCM-PCDL identified 761 and 673 compounds across 100 TCMPs in positive and negative modes, respectively, and Tables S2 and S3 present detailed information. These compounds comprised 10 primary categories, including flavonoids, phenolic acids, terpenoids, alkaloids, coumarins, lignans, phenylpropanoids, quinones, iridoids, and others (Fig. 5A). Flavonoids were the most abundant, with 253/240 compounds identified in positive/negative modes, which can be attributed to the frequent use of flavonoid-rich raw materials such as *Scutellariae Radix* and *Glycyrrhizae Radix* et *Rhizoma*. Notably, *Scutellariae Radix* was present in 23 prescriptions and *Glycyrrhizae Radix* in 21. Beyond flavonoids, a significant number of alkaloids (170), coumarins (95), terpenoids (66), and phenolic acids (41) were detected in positive mode. Alkaloids and coumarins, which exhibited strong responses in the ESI-positive mode, were predominantly sourced from *Angelicae Dahuricae Radix*, *Coptidis Rhizoma*, and so forth. In negative mode, phenolic acids (120) and terpenes (109), derived from *Glycyrrhizae Radix* et *Rhizoma*, *Chuanxiong Rhizoma*, etc., exhibited favorable responses and formed a notable proportion.

The retention times and molecular weight distributions of various compound categories exhibited distinct patterns (Fig. 5B). Flavonoids, lignans, and coumarins shared similar retention time distributions, primarily within 10–25 min. The molecular weights of flavonoids and lignans were mostly within 300–700 Da, whereas coumarins typically remained below 300 Da. Alkaloids, which exhibited strong responses in positive mode, were primarily detected before 20 min, with molecular weights between 100 and 600 Da. Phenolic compounds, which responded well in negative mode, were generally distributed before 15 min, with molecular weights in the 100–700 Da range. Terpenes exhibited a wider distribution, with retention times between 14 and 27 min and molecular weights spanning from 200 to 1200 Da.

A statistical analysis of TCMP-annotated compounds was conducted (Fig. 5C). Prescription P89, comprising six medicinal ingredients, exhibited the highest number of annotated compounds, with 72 and 95 annotated compounds detected in positive and negative modes, respectively. Across 100 prescriptions, 18% featured more than 40 identified compounds in both modes. The majority of prescriptions had more than 20 annotated compounds, with 64% and 76% exceeding this threshold in positive and negative modes, respectively.

This study is the first to report the chemical constituents of 73 TCMPs. Only 27 were previously documented in the literature, with four having limited compound identification. For instance, prior studies on P40 reported only 28 compounds, whereas our analysis identified 60/24 compounds in positive/negative modes. Additionally, this study significantly expanded upon previous research, presenting novel findings. Although 118 compounds from P89 had been identified using linear ion trap/orbitrap MS, herein, we report new compounds, such as baohuoside V, baohuoside VII, and 2″-*O*-rhamnosylicariside II from *Epimedii Folium*; 3-hydroxybakuchio, neobavaisoflavone, and 8-prenyldaidzein from *Psoraleae Fructus*; officinalisinin I and timosaponin C from *Anemarrhenae Rhizoma*; tanshinone I and dihydrotanshinone I from *Salviae Miltiorrhizae Radix* et *Rhizoma*; and uridine and catalpol from *Rehmanniae Radix*. These newly identified compounds provided valuable references for further in-depth studies on P89's chemical constituents and quality control.

Beyond compound analysis, we traced the medicinal ingredients in 100 TCMPs and generated a heatmap illustrating the tracing rates (Fig. 5D). The results indicated that 100 TCMPs could be linked to between 0 and 10 raw materials. For instance, P2 was successfully traced to *Scutellariae Radix*, *Rhei Radix* et *Rhizoma* (RRER), and *Coptidis Rhizoma*, achieving a 100% tracing rate. Overall, 13 prescriptions exhibited complete tracing rates of 100%. When the number of medicinal ingredients increased, the likelihood of successful tracing decreased. In positive mode, 25 prescriptions traced at least 40% of their ingredients, whereas in negative mode, 27 prescriptions met this threshold. Altogether, 51 prescriptions exceeded a 40% tracing threshold. High tracing rates were prevalent among prescriptions with distinctive chemical profiles, such as anthraquinones in RRER, flavonoids in *Scutellariae Radix*, alkaloids in *Coptidis Rhizoma*, and phenolic compounds in *Salviae Miltiorrhizae Radix* et *Rhizoma*.

### 3.6 Causes of inconsistent TCMP quality

**Fig. 6:**
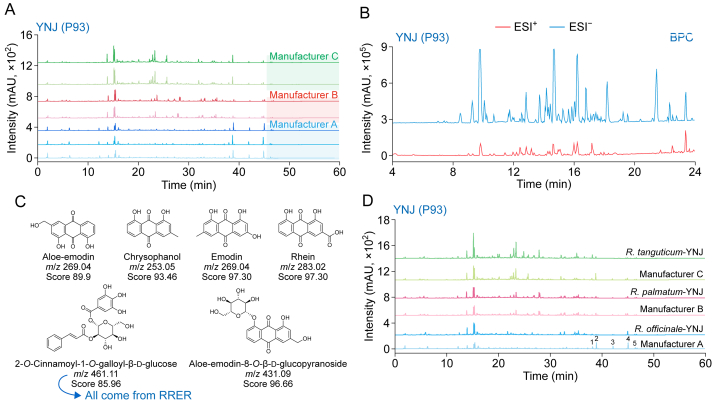
Causes of inconsistent quality in Yinianjin (YNJ) (P93) prescription and validation. (A) Fingerprint spectra of seven batches of P93 from different manufacturers. (B) Base peak intensity (BPI) plot of P93 in positive and negative modes. (C) Compound annotation and ingredient tracking results for P93 across manufacturers, based on differential peaks identified using Traditional Chinese Medicine Personal Compound Database and Library (TCM-PCDL) database. (D) Comparison spectra from self-prepared YNJs and three commercial YNJs from different manufacturers (1, aloe-emodin; 2, rhein; 3, emodin; 4, chrysophanol; and 5, physcion). BPC: base peak chromatogram; RRER: *Rhei Radix* et *Rhizoma*; *R. tanguticum*: *Rheum tanguticum* Maxim. ex Balf.; *R. palmatum*: *Rheum palmatum* L.; *R. officinale*: *Rheum officinale* Baill.

Metabolite annotation using the TCM-PCDL database and raw material tracing facilitated the identification of factors underlying quality inconsistencies. For instance, the analysis of P93 (Yinianjin (YNJ)) revealed notable variability across three manufacturers (Fig. 6A). P93, comprising RRER, processed *PharbitidisSemen*, *Arecae Semen*, *Ginseng Radix* et *Rhizoma*, and *Cinnabaris*, was used for upset stomach caused by overeating. According to Chinese Pharmacopeia (2020 edition), *Cinnabaris* was ground into extremely fine powder using the water-floating method, whereas the other four ingredients were pulverized into fine powder [24]. All the powder was mixed, sieved, and uniformly blended to obtain the final product. Although the production process was straightforward, batch-to-batch consistency from the same manufacturer was relatively high (similarity = 0.93–0.98), indicating that production methods had a limited impact on quality variations. Instead, the raw materials were the primary causes of inconsistencies. To explore this, base peak intensity (BPI) data were collected and subjected to automated metabolite annotation using TCM-PCDL (Fig. 6B). Key differential peaks (peaks 1 to 5) within 20–45 min retention range were aloe-emodin, rhein, emodin, chrysophanol, and physcion, compounds derived from RRER. Verification with reference standards confirmed this annotation, indicating that RRER contributed to the quality discrepancies (Fig. 6C). RRER can be derived from three botanical sources: *R. officinale*, *R*. *tanguticum*, and *R. palmatum*. Manufacturers may employ different sources, resulting in divergent fingerprint profiles. To validate this, three reference samples of RRER were obtained from China National Center for Food Safety Risk Assessment and processed according to the Chinese Pharmacopeia (2020 edition) [24].

The results (Fig. 6D) indicate that the fingerprint of self-prepared YNJ with *R. palmatum* closely matched that of manufacturer B's product, yielding a high similarity score of 0.97, particularly aligning within the 22–28 min retention range. By contrast, manufacturer C's product exhibited highest similarity (0.87) to YNJ made with *R. tanguticum*. Manufacturer A's YNJ exhibited similarity (0.56) to YNJ using *R. officinale*, indicating *R. officinale* as the likely source, with the possibility of mixed raw materials. In summary, three manufacturers utilized different sources of RRER, contributing to the batch variability observed in P93. Despite these variations, all products adhered to regulatory standards.

### 3.7 Method advantages and limitations

The consistency evaluation of TCMPs through chromatographic spectroscopy has traditionally focused on individual product, such as Danshen and Licorice tablets. In such cases, method development for each TCMP typically consumes at least one week. This study proposed a universal fingerprinting method that achieved effective separation across 100 TCMPs, involving 645 batches and 214 manufacturers. This approach significantly reduced time required for condition development and, in the future, can continue to expand the sample size, allowing large-scale TCM quality assessment. For the first time, it achieved a synchronous quality comparison of over a hundred TCMPs. However, a key limitation is the lack of automation in data processing, particularly the need for manual correction of retention time deviations. Therefore, addressing this challenge is a priority for future improvements.

For metabolite annotation through HRMS, previous studies have only reported chemical profiling using LC-HRMS for 27 TCMPs. This study is the first to identify the chemical constituents of 73 TCMPs. Traditional methods for analyzing a single prescription typically required at least one month, whereas LC-HRMS combined with TCM-PCDL achieved rapid annotation for 100 TCMPs within the same timeframe. In addition, TCM-PCDL enhanced annotation through secondary fragment matching and scoring functions. The database comprised chemical compounds derived from TCM, making it particularly suitable for annotating metabolites in TCMPs. However, TCM-PCDL includes MS data for only 2,565 components from TCMs listed in Chinese Pharmacopeia (2020 edition) [24]. Therefore, compounds that are not found in the database cannot be identified, and it does not distinguish between isomers. Moreover, data acquisition is restricted to Agilent Technologies instruments. To address these limitations, one approach is to expand TCM-PCDL to broaden the scope of identification or to develop a custom database compatible with multiple platforms. Furthermore, incorporating chromatographic parameters such as retention time could enhance the accuracy of isomer identification.

## 4 Conclusions

This study proposes a novel strategy to assess TCMP quality consistency and identify the underlying causes of variability. A dataset comprising 645 batches from 100 TCMPs was analyzed, and HCA combined with similarity identified quality inconsistencies in 19 prescriptions. The findings reveal that products from multiple manufacturers are significantly more prone to quality issues, particularly when prescription costs exceed CNY 7 per tablet, the selling price falls below CNY 0.5 per tablet, or the price differences between manufacturers exceed twofold. To investigate the causes of inconsistencies, we utilized LC-HRMS combined with TCM-PCDL database, which is an MS database built from authentic standards derived from TCM. This approach enables simultaneous analysis of 100 prescriptions, identifying up to 95 components per prescription. Furthermore, the approach facilitates tracking of medicinal ingredients, achieving 100% traceability for 13 prescriptions. Finally, YNJ preparations were used as a representative case study to validate the effectiveness of the integrated approach.

While these results are promising, certain limitations remain. In chromatographic analysis, the lack of automation in data processing hampers analytical efficiency. At the MS level, many compounds, particularly isomers, are not effectively identified owing to the limited scale and isomer resolution capability of the TCM-PCDL database. These limitations are expected to be mitigated by developing a dedicated TCM database that integrates data processing and management functionalities while ensuring sample scalability and multiplatform compatibility. For quality assessment, comprehensively considering key factors such as active ingredient content, dissolution rate, and biological activity is crucial.

These results provide valuable insights for regulatory authorities regarding the direction of their inspections. Regulatory agencies should prioritize inspections of products with low prices, those containing high-value materials, or those exhibiting significant price discrepancies across multiple manufacturers. Additionally, integrating fingerprint database into a sample consistency monitoring system can be a complementary approach to statutory testing methods.

## CRediT authorship contribution statement

**Huiting Ou:** Writing – original draft, Methodology, Formal analysis, Data curation. **Chunxiang Liu:** Formal analysis. **Saiyi Ye:** Data curation. **Lin Yang:** Data curation. **Qirui Bi:** Validation. **Wenlong Wei:** Validation. **Hua Qu:** Validation. **Yaling An:** Supervision. **Jianqing Zhang:** Writing – review & editing, Methodology. **De-an Guo:** Writing – review & editing, Funding acquisition, Conceptualization.

## Declaration of competing interest

The authors declare that there are no conflicts of interest.
