# Supplementary material for: A novel approach to assessing quality issues and component annotation in TCM prescription: Insights from 100 common TCM products
Source: J Pharm Anal. 2025 May 9;15(10):101332. doi: 10.1016/j.jpha.2025.101332 (PMC12634852; doi:10.1016/j.jpha.2025.101332)
Supplement: Multimedia component 1 [file mmc1.docx]

Supporting Information

A Novel Approach to Assessing Quality Issues and Component Annotation in Traditional Chinese Medicine Prescription: Insights from 100 Common TCM Products

Contents

[Table S1 Detailed information of 100 species of CPM (645 batches) 3](#_Toc183526258)

[Fig S1 Scatter diagram of similarity among 100 Traditional Chinese Medicine prescriptions 11](#_Toc183526259)

[Fig S2 Dosage forms between problematic and normal products 11](#_Toc183526260)

[Table S2 761 chemical components characterized in 100 TCMPs (ESI+) 12](#_Toc183526261)

[Table S3 673 chemical components characterized in 100 TCMPs (ESI-) 74](#_Toc183526262)

# **Table S1 Detailed information of 100 species of Traditional Chinese Medicine prescriptions (645 batches)**

| NO. | Abbreviation | Chinese Name | Medicinal ingredients | Manufacturers | Dosage Form | Batches |
| --- | --- | --- | --- | --- | --- | --- |
| P1 | XCHKL | Xiaochaihu Granule | 7 | 8 | Granule | 10 |
| P2 | YQKL | Yiqing Granule | 3 | 4 | Granule | 6 |
| P3 | SHP | Sanhuang Tablet | 3 | 5 | Tablet | 5 |
| P4 | XQKL | Xinqin Granule | 10 | 6 | Granule | 6 |
| P5 | JWQHW | Jiuwei Qianghuo Pill | 9 | 3 | Pill | 5 |
| P6 | SXBDYKL | Shuangxin Bidouyan Granule | 14 | 2 | Granule | 5 |
| P7 | SHLKFY | Shuanghuanglian Oral Liquid | 3 | 5 | Oral Liquid | 10 |
| P8 | LHQWJN | Lianhua Qingwen Capsule | 13 | 1 | Capsule | 5 |
| P9 | SFJDJN | Shufeng Jiedu Capsule | 7 | 1 | Capsule | 5 |
| P10 | FFYXCP | Fufang Yuxingcao Tablet | 5 | 7 | Tablet | 10 |
| P11 | XECQQRKL | Xiao´er Chiqiao Qingre Granule | 14 | 1 | Granule | 10 |
| P12 | ASBNY | Anshen Bunao Syrup | 7 | 5 | Oral Liquid | 10 |
| P13 | ASJN | Anshen Capsule | 8 | 3 | Capsule | 5 |
| P14 | KBDKFY | Kangbingdu Oral Liquid | 9 | 5 | Oral Liquid | 10 |
| P15 | XSJKL | Xiasangju Granule | 3 | 6 | Granule | 6 |
| P16 | QLSTJN | Qianlie Shutong Capsule | 8 | 1 | Capsule | 5 |
| P17 | ZKBP | Zhikebao Tablet | 14 | 1 | Tablet | 4 |
| P18 | XSTRJN | Xuesaitong Soft Capsule | 1 | 2 | Capsule | 5 |
| P19 | FFXSTJN | Fufang Xueshuantong Capsule | 4 | 1 | Capsule | 5 |
| P20 | SYKFP | Shenyan Kangfu Tablet | 13 | 1 | Tablet | 5 |
| P21 | JSBJN | Jinshuibao Capsule | 1 | 1 | Capsule | 5 |
| P22 | GMQRKL | Ganmao Qingre Granule | 11 | 7 | Granule | 7 |
| P23 | NXTJN | Naoxintong Capsule | 16 | 1 | Capsule | 5 |
| P24 | BZYXW | Baizi Yangxin Pill | 12 | 2 | Pill | 5 |
| P25 | SSYXJN | Shensong Yangxin Capsule | 9 | 1 | Capsule | 5 |
| P26 | FFDSDW | Fufang Danshen Dripping Pill | 3 | 1 | Pill | 5 |
| P27 | XSTP | Xuesaitong Tablet | 1 | 3 | Tablet | 5 |
| P28 | XYW | Xiaoyao Pill | 7 | 5 | Pill | 10 |
| P29 | DZZGJN | Duzhong Zhuangui Capsule | 23 | 2 | Capsule | 7 |
| P30 | LWDHW | Liuwei Dihuang Pill | 6 | 7 | Pill | 7 |
| P31 | YXYP | Yinxingye Tablet | 1 | 5 | Tablet | 5 |
| P32 | FFDSP | Fufang Danshen Tablet | 3 | 5 | Tablet | 5 |
| P33 | FFJYHKL | Fufang Jinyinhua Granule | 3 | 5 | Granule | 10 |
| P34 | JWXSP | Jianwei Xiaoshi Tablet | 5 | 5 | Tablet | 5 |
| P35 | GZFLJN | Guizhi Fuling Capsule | 5 | 1 | Capsule | 5 |
| P36 | WYNKL | Weiyanning Granule | 8 | 5 | Granule | 10 |
| P37 | BLGKL | Banlangen Granule | 1 | 7 | Granule | 10 |
| P38 | FFBLGKL | Fufang Banlangen Granule | 2 | 5 | Granule | 5 |
| P39 | NXQP | Naoxinqing Tablet | 1 | 1 | Tablet | 5 |
| P40 | XFZYJN | Xuefu Zhuyu Capsule | 11 | 1 | Capsule | 5 |
| P41 | YXQNKL | Yangxue Qingnao Granule | 11 | 1 | Granule | 5 |
| P42 | YXSFKL | Yangxue Shengfa Capsule | 9 | 4 | Capsule | 10 |
| P43 | CYNP | Changyanning Tablet | 5 | 1 | Tablet | 5 |
| P44 | JZTJ | Jizhi Syrup | 8 | 1 | Oral Liquid | 5 |
| P45 | WSKL | Weisu Granule | 8 | 1 | Granule | 5 |
| P46 | KTJN | Kuntai Capsule | 6 | 1 | Capsule | 4 |
| P47 | LDXGW | Longdan Xiegan Pill | 10 | 5 | Pill | 5 |
| P48 | BYLP | Biyanling Tablet | 8 | 3 | Tablet | 9 |
| P49 | PDLXYP | Pudilan Xiaoyan Tablet | 4 | 5 | Tablet | 5 |
| P50 | PDLXYKFY | Pudilan Xiaoyan Oral Liquid | 4 | 1 | Oral Liquid | 5 |
| P51 | LQKFY | Lanqin Oral Liquid | 5 | 1 | Oral Liquid | 5 |
| P52 | LBP | Libi Tablet | 7 | 2 | Tablet | 5 |
| P53 | FLKHJ | Feilike Mixture | 7 | 1 | Oral Liquid | 5 |
| P54 | SHZKJN | Suhuang Zhike Capsule | 9 | 1 | Capsule | 5 |
| P55 | FKQJJN | Fuke Qianjin Capsule | 8 | 1 | Capsule | 5 |
| P56 | TWBXW | Tianwang Buxin Pill | 15 | 3 | Pill | 5 |
| P57 | WLJN | Wuling Capsule | 1 | 1 | Capsule | 5 |
| P58 | WXKL | Wenxin Granule | 5 | 1 | Granule | 5 |
| P59 | RLQKL | Relinqing Granule | 1 | 1 | Granule | 5 |
| P60 | WMW | Wumei Pill | 10 | 2 | Pill | 5 |
| P61 | TTKFY | Tongtian Oral Liquid | 11 | 1 | Oral Liquid | 5 |
| P62 | YDXNTRJN | Yindan Xinnaotong Soft Capsule | 8 | 1 | Capsule | 5 |
| P63 | JZKFY | Jinzhen Oral Liquid | 8 | 1 | Oral Liquid | 5 |
| P64 | XZKJN | Xuezhikang Capsule | 1 | 1 | Capsule | 5 |
| P65 | TMXNJN | Tianma Xingnao Capsule | 6 | 1 | Capsule | 5 |
| P66 | BDGJN | Beidougen Capsule | 1 | 1 | Capsule | 5 |
| P67 | HKJN | Huangkui Capsule | 1 | 1 | Capsule | 5 |
| P68 | SLXMKJN | Songling Xuemaikang Capsule | 3 | 1 | Capsule | 4 |
| P69 | SMYWKL | Shenmei Yangwei Granule | 11 | 5 | Granule | 5 |
| P70 | TXLJN | Tongxinluo Capsule | 12 | 1 | Capsule | 5 |
| P71 | XKSP | Xinkeshu Tablet | 5 | 1 | Tablet | 5 |
| P72 | DGXJN | Danguixiang Capsule | 23 | 1 | Capsule | 5 |
| P73 | HXZQKFY | Huoxiang Zhengqi Oral Liquid | 10 | 1 | Oral Liquid | 5 |
| P74 | SQTSJN | Sanqi Tongshu Capsule | 1 | 1 | Capsule | 5 |
| P75 | YNBYJN | Yunnan Baiyao Capsule | 5 | 1 | Capsule | 5 |
| P76 | RSBQJN | Renshen Beiqi Capsule | 2 | 1 | Capsule | 3 |
| P77 | SLBZS | Shenling Baizhu San | 10 | 6 | Powder | 10 |
| P78 | CXLNZDW | Chuanxinlianneizhi Dripping Pill | 1 | 1 | Pill | 5 |
| P79 | SXBHJ | Shengxuebao Mixture | 7 | 1 | Oral Liquid | 5 |
| P80 | XEGMKL | Xiao’er Ganmao Granule | 10 | 8 | Granule | 10 |
| P81 | JWGTJN | Jiuwei Gantai Capsule | 9 | 2 | Capsule | 6 |
| P82 | DHTBKL | Dahuang Tongbian Granule | 1 | 2 | Granule | 10 |
| P83 | HGP | Hugan Tablet | 6 | 8 | Tablet | 10 |
| P84 | HXZTJN | Huoxue Zhitong Capsule | 6 | 3 | Capsule | 10 |
| P85 | QLPPL | Qiangli Pipa Syrup | 7 | 7 | Oral Liquid | 10 |
| P86 | ZFTGW | Zhuifeng Tougu Pill | 21 | 2 | Pill | 10 |
| P87 | FFYJKL | Fufang Yangjiao Granule | 4 | 8 | Granule | 10 |
| P88 | SMY | ShengMaiYin(DangCanFang) | 3 | 8 | Oral Liquid | 10 |
| P89 | XLGBJN | Xianling Gubao Capsule | 6 | 1 | Capsule | 10 |
| P90 | CBZKL | Chuanbei Zhike Syrup | 7 | 8 | Oral Liquid | 10 |
| P91 | JKJN | Jingkang Capsule | 9 | 8 | Capsule | 10 |
| P92 | DHZCW | Dahuang Zhechong Pill | 12 | 3 | Pill | 8 |
| P93 | YNJ | Yinianjin | 5 | 3 | Powder | 7 |
| P94 | DKD | Dingkun Pill | 30 | 3 | Pill | 5 |
| P95 | FSATP | Fengshi Antai Tablet | 25 | 4 | Tablet | 10 |
| P96 | JGLZDP | Jiaogulan Zongdai Tablet | 1 | 2 | Tablet | 8 |
| P97 | XHW | Xihuang Pill | 4 | 5 | Pill | 8 |
| P98 | YYP | Yanyan Tablet | 12 | 8 | Tablet | 10 |
| P99 | HXZTG | Huoxue Zhitong Patch | 28 | 2 | Patch | 5 |
| P100 | FFYXCHJ | Fufang Yuxingcao Syrup | 5 | 3 | Oral Liquid | 5 |





# Fig S1 Scatter diagram of similarity among 100 Traditional Chinese Medicine prescriptions-1





# Fig S2 Scatter diagram of similarity among 100 Traditional Chinese Medicine prescriptions-2





# Fig S3 Scatter diagram of similarity among 100 Traditional Chinese Medicine prescriptions-3





# Fig S4 Scatter diagram of similarity among 100 Traditional Chinese Medicine prescriptions-4





# Fig S5 Scatter diagram of similarity among 100 Traditional Chinese Medicine prescriptions-5

# Fig S6 Dosage forms between problematic and normal products

# Table S2 761 chemical components characterized in 100 Traditional Chinese Medicine prescriptions (ESI+)

| No. | RT(min) | Mass(ppm) | Precursors/Adduction | Formula | Error(ppm) | Score | Product ion | Identification | Class | Source | CAS |
| --- | --- | --- | --- | --- | --- | --- | --- | --- | --- | --- | --- |
| 1 | 0.94 | 147.1126 | [M+H]⁺ | C6H14N2O2 | -1.81 | 88.85 | 147.11,130.08,112.07,84.08 | L-Lysine | Amino acid | 21 | 56-87-1 |
| 2 | 0.98 | 175.1186 | [M+H]⁺ | C6H14N4O2 | 2.24 | 94.91 | 175.11,158.09,130.09,116.06,70.06,60.05 | L(+)-Arginine | Amino acid | 1,2,3,4,5,11,14,16,19,20,21,22,23,24,28,32,34,37,38,40,47,48,61,63,70,73,74,75,76,80,84,87,88,90,93,94,95,98 | 74-79-3 |
| 3 | 1.09 | 162.1122 | [M+H]⁺ | C7H15NO3 | 1.90 | 92.18 | 162.11,103.04,85.02,60.08 | Carnitine | Alkaloid | 25,64,70,84 | 406-76-8 |
| 4 | 1.10 | 118.0861 | [M+H]⁺ | C5H11NO2 | -2.34 | 97.48 | 118.08,59.07 | Betaine or its isomer | Alkaloid | 11,16,29,40,44,54,55,59,66,67,95 | 107-43-7 |
| 5 | 1.15 | 138.0546 | [M+H]⁺ | C7H7NO2 | 3.58 | 90.94 | 138.05,94.06 | Trigonelline | Alkaloid | 54,67,77,81 | 535-83-1 |
| 6 | 1.17 | 144.1013 | [M+H]⁺ | C7H13NO2 | 2.31 | 91.38 | 144.10,84.08 | Stachydrine | Alkaloid | 20,44,45,54,59,66,72,73 | 32039-73-9 |
| 7 | 1.25 | 127.0389 | [M+H]⁺ | C6H6O3 | -0.36 | 78.85 | 127.03,109.02,81.03,71.04,53.03 | 5-Hydroxymethyl-2-Furaldehyde or its isomer | Furan | 12,85,90 | 67-47-0 |
| 8 | 1.26 | 365.1045 | [M+Na]⁺ | C19H18O6 | 2.09 | 89.84 | 365.10,265.11,163.11 | Pd-Ib or its isomer | Coumarins | 1,23,33,36,71,85 | 78416-90-7 |
| 9 | 1.33 | 118.0859 | [M+H]⁺ | C5H11NO2 | 1.25 | 92.46 | 118.08,72.08,55.05 | L-Valine | Amino acid | 57,70 | 72-18-4 |
| 10 | 1.38 | 136.0615 | [M+H]⁺ | C5H5N5 | -6.11 | 82.74 | 136.06,119.03,94.02 | Adenine or its isomer | Alkaloid | 11,21,23,39,57,61,66,71,74,76,77,93,96,97 | 73-24-5 |
| 11 | 1.39 | 130.0860 | [M+H]⁺ | C6H11NO2 | -0.77 | 76.05 | 130.08,84.08 | Pipecolinic acid | Organic acid | 71 | 535-75-1 |
| 12 | 1.48 | 152.0564 | [M+H]⁺ | C5H5N5O | -2.30 | 89.43 | 152.05,135.03 | Hydroxytyrosol or its isomer | Phenolics | 21 | 10597-60-1 |
| 13 | 1.48 | 148.0979 | [M+H]⁺ | C6H13NO3 | 6.74 | 86.82 | 148.09,74.02 | 4-Hydroxyisoleucine | Amino acid | 61 | 781658-23-9 |
| 14 | 1.53 | 244.0928 | [M+H]⁺ | C9H13N3O5 | -1.77 | 88.89 | 244.09,112.05 | Cytidine | Alkaloid | 57 | 65-46-3 |
| 15 | 1.60 | 168.1020 | [M+H]⁺ | C9H13NO2 | 0.49 | 89.55 | 168.10,150.09,135.06,119.05,107.04,91.05 | Synephrine or its isomer | Alkaloid | 34,40,44,45,73 | 94-07-5 |
| 16 | 1.61 | 123.0552 | [M+H]⁺ | C6H6N2O | -0.98 | 96.24 | 123.05,108.02,80.04 | Nicotinamide | Alkaloid | 11,26,31,39,41,57,64,65,67,70,74,76,79,84,93 | 98-92-0 |
| 17 | 1.63 | 365.1050 | [M+Na]⁺ | C19H18O6 | -1.75 | 89.84 | 365.10,265.11,163.11 | Pd-Ib or its isomer | Coumarins | 7,33,85 | 78416-90-7 |
| 18 | 1.70 | 527.1544 | [M+Na]⁺ | C18H32O16 | 0.68 | 92.37 | 527.15,365.10,247.03,185.03 | 1-Kestose | Carbohydrate | 23 | 470-69-9 |
| 19 | 1.76 | 364.0650 | [M+H]⁺ | C10H14N5O8P | -1.44 | 94.79 | 364.06,248.07,152.05 | Guanosine-5'-monophosphate | Alkaloid | 57 | 85-32-5 |
| 20 | 1.78 | 130.0498 | [M+H]⁺ | C5H7NO3 | 0.45 | 93.63 | 130.04,84.04,56.04 | L-Pyroglutamic acid | Amino acid | 1,3,5,7,9,10,12,13,15,16,20,21,22,23,24,25,26,28,29,30,32,33,34,37,38,40,41,42,45,47,48,54,56,57,58,59,65,67,69,70,72,75,76,77,79,80,82,84,85,86,87,88,89,90,98 | 98-79-3 |
| 21 | 1.83 | 137.0457 | [M+H]⁺ | C5H4N4O | 0.23 | 86.33 | 137.04,119.03,110.04,94.03 | Hypoxanthine or its isomer | Alkaloid | 54,66,70,87 | 68-94-0 |
| 22 | 1.96 | 127.0386 | [M+H]⁺ | C6H6O3 | 3.06 | 77.19 | 127.03,109.02,81.03,53.03 | 5-Hydroxymethyl-2-Furaldehyde or its isomer | Furan | 7,14 | 67-47-0 |
| 23 | 2.18 | 132.1019 | [M+H]⁺ | C6H13NO2 | -0.76 | 95.15 | 132.10,86.09 | L-leucine or its isomer | Amino acid | 5,9,10,12,19,20,26,32,34,37,41,42,47,57,58,63,65,70,71,72,73,75,76,77,79,80,88,89,90,92,93,94,95,98 | 61-90-5 |
| 24 | 2.19 | 132.1015 | [M+H]⁺ | C6H13NO2 | 3.81 | 94.61 | 132.10,86.09,69.06 | L-Isoleucine | Amino acid | 14,16,40,59,65,70,73,75,87,88,89,90,98 | 73-32-5 |
| 25 | 2.22 | 153.0399 | [M+H]⁺ | C5H4N4O2 | 3.65 | 92.18 | 153.04,136.01,110.03 | 2,6-Dihydroxypurine | Alkaloid | 66,70,81,84,87 | 69-89-6 |
| 26 | 2.31 | 365.1045 | [M+Na]⁺ | C19H18O6 | -4.86 | 89.84 | 365.10,265.11,163.11 | Pd-Ib or its isomer | Coumarins | 4,7,85 | 78416-90-7 |
| 27 | 2.42 | 113.0344 | [M+H]⁺ | C4H4N2O2 | 1.45 | 98.75 | 113.03,96.00,70.02 | Uracil or its isomer | Alkaloid | 9,11,13,16,21,25,26,28,29,37,40,52,55,57,65,67,72,76,89,92,98 | 66-22-8 |
| 28 | 2.46 | 180.1014 | [M+H]⁺ | C10H13NO2 | -2.20 | 92.02 | 180.10,163.07,145.06,137.05,117.07,91.05 | (-)-Salsolinol or its isomer | Alkaloid | 16,30,60,67,72,75,81,86,87,95 | 27740-96-1 |
| 29 | 2.47 | 156.0760 | [M+H]⁺ | C6H9N3O2 | 3.89 | 81.71 | 156.07,110.07,95.06 | L-Histidine | Amino acid | 54 | 71-00-1 |
| 30 | 2.49 | 245.0759 | [M+H]⁺ | C9H12N2O6 | -0.87 | 78.05 | 245.07,113.03 | Uridine | Alkaloid | 57 | 58-96-8 |
| 31 | 2.50 | 182.0811 | [M+H]⁺ | C9H11NO3 | -0.71 | 91.55 | 182.08,165.05,147.04,136.07,123.04,119.05,107.04,95.04,91.05,77.03,65.03 | L-Tyrosine or ots isomer | Amino acid | 3,11,14,19,20,21,41,52,57,59,63,64,70,73,75,77,79,84,87,88,89,90,93,98 | 60-18-4 |
| 32 | 2.57 | 851.2618 | [M+Na]⁺ | C30H52O26 | -5.97 | 83.06 | 851.26,689.21,527.15,509.14,347.09,185.04 | 1F-fructofuranosylnystose | Carbohydrate | 98 | 59432-60-9 |
| 33 | 2.80 | 132.1013 | [M+H]⁺ | C6H13NO2 | -5.13 | 85.76 | 132.10,86.09 | L-leucine or its isomer | Amino acid | 44,61 | 61-90-5 |
| 34 | 3.10 | 182.0806 | [M+H]⁺ | C9H11NO3 | -2.67 | 88.03 | 182.08,165.05,147.04,136.07,123.04,119.05,107.04,95.04,91.05,77.03,65.03 | L-Tyrosine or ots isomer | Amino acid | 44,61 | 60-18-4 |
| 35 | 3.50 | 113.0342 | [M+H]⁺ | C4H4N2O2 | -3.50 | 82.52 | 113.03,96.00,70.02 | Uracil or its isomer | Alkaloid | 61 | 66-22-8 |
| 36 | 4.02 | 268.1037 | [M+H]⁺ | C10H13N5O4 | 0.98 | 92.17 | 268.10,136.06,85.02,73.02,57.03 | Adenosine or its isomer | Alkaloid | 1,2,3,7,10,12,14,16,18,22,25,26,30,31,32,34,36,37,38,40,42,54,55,56,58,59,60,63,65,66,67,69,70,71,72,73,74,75,76,79,80,85,86,87,88,89,90,93,94,95,98 | 58-61-7 |
| 37 | 4.10 | 136.0616 | [M+H]⁺ | C5H5N5 | 1.70 | 90.26 | 136.06,119.03,94.02 | Adenine or its isomer | Alkaloid | 14,20,21,32,40,57,64,67,75 | 73-24-5 |
| 38 | 4.38 | 166.1226 | [M+H]⁺ | C10H15NO | 1.44 | 90.68 | 166.12,121.06,103.05,93.07,77.03 | Hordenine | Alkaloid | 31,54,86 | 539-15-1 |
| 39 | 4.86 | 136.0610 | [M+H]⁺ | C5H5N5 | -6.71 | 81.73 | 136.06,119.03,94.02 | Adenine or its isomer | Alkaloid | 21 | 73-24-5 |
| 40 | 5.09 | 268.1028 | [M+H]⁺ | C10H13N5O4 | 4.47 | 91.14 | 268.10,136.06,85.02,73.02,57.03 | Adenosine or its isomer | Alkaloid | 27 | 58-61-7 |
| 41 | 5.22 | 152.0570 | [M+H]⁺ | C5H5N5O | -2.20 | 80.76 | 152.05,135.03 | Hydroxytyrosol or its isomer | Phenolics | 16,20,21,28,40,41,52,55,57,67,75,81 | 10597-60-1 |
| 42 | 5.23 | 137.0457 | [M+H]⁺ | C5H4N4O | 1.69 | 92.16 | 137.04,119.03,110.03,94.03 | Hypoxanthine or its isomer | Alkaloid | 52,54,65,86,92 | 68-94-0 |
| 43 | 5.29 | 284.0992 | [M+H]⁺ | C10H13N5O5 | -0.47 | 92.09 | 284.09,152.09,135.03 | Guanosine | Alkaloid | 14,16,21,25,32,37,40,55,67,73,74,75,80,81,89,94,95,98 | 118-00-3 |
| 44 | 5.31 | 166.0827 | [M+H]⁺ | C9H11NO2 | 0.63 | 98.17 | 166.08,120.08,107.04,103.05,93.06,79.05 | L-Phenylalanine or its isomer | Amino acid | 3,11,14,16,19,20,21,32,34,37,40,45,52,57,58,63,64,65,70,73,74,75,77,79,80,81,84,85,87,88,89,90,93,98,100 | 63-91-2 |
| 45 | 5.35 | 284.1006 | [M+H]⁺ | C10H13N5O5 | 7.09 | 81.64 | 284.10,152.05 | Crotonoside | Alkaloid | 45 | 1818-71-9 |
| 46 | 5.55 | 180.1012 | [M+H]⁺ | C10H13NO2 | 1.81 | 81.53 | 180.10,163.07,145.06,137.05,117.07,91.05 | (-)-Salsolinol or its isomer | Alkaloid | 67 | 27740-96-1 |
| 47 | 5.77 | 125.0481 | [M+H]⁺ | C7H8O2 | 2.83 | 89.78 | 125.04,107.04 | P-hydroxylbenzyl alcohol or its isomer | Phenolics | 42 | 623-05-2 |
| 48 | 5.92 | 127.0387 | [M+H]⁺ | C6H6O3 | 2.11 | 91.76 | 127.03,109.02,81.03,53.03 | 5-Hydroxymethyl-2-Furaldehyde or its isomer | Furan | 10,12,13,25,30,32,34,39,40,42,54,55,56,60,65,67,69,73,75,86,88,89,94,95,96,98 | 67-47-0 |
| 49 | 6.02 | 168.0898 | [M+H]⁺ | C9H13NO2 | -5.75 | 83.03 | 168.10,150.09,135.06,119.05,107.04,91.05 | Synephrine or its isomer | Alkaloid | 45 | 94-07-5 |
| 50 | 6.10 | 268.1030 | [M+H]⁺ | C10H13N5O4 | 2.86 | 90.75 | 268.10,136.06,85.02,73.02,57.03 | Adenosine or its isomer | Alkaloid | 15 | 58-61-7 |
| 51 | 6.12 | 136.0609 | [M+H]⁺ | C5H5N5 | -5.00 | 82.05 | 136.06,119.03,94.02 | Adenine or its isomer | Alkaloid | 61 | 73-24-5 |
| 52 | 6.18 | 152.0571 | [M+H]⁺ | C5H5N5O | 0.77 | 91.99 | 152.05,135.03 | Hydroxytyrosol or its isomer | Phenolics | 21,44,57,61 | 10597-60-1 |
| 53 | 6.21 | 166.0860 | [M+H]⁺ | C9H11NO2 | -1.31 | 96.32 | 166.08,120.08,107.04,103.05,93.06,79.05 | L-Phenylalanine or its isomer | Amino acid | 23,44,61 | 63-91-2 |
| 54 | 6.45 | 118.0842 | [M+H]⁺ | C5H11NO2 | 2.07 | 90.17 | 118.08,59.07 | Betaine or its isomer | Alkaloid | 23 | 107-43-7 |
| 55 | 6.47 | 130.0316 | [M+H]⁺ | C5H7NOS | 5.20 | 89.59 | 130.02,96.04,89.94,70.06,68.05,60.97 | Epigoitrin | Alkaloid | 37 | 1072-93-1 |
| 56 | 6.64 | 125.0490 | [M+H]⁺ | C7H8O2 | -2.96 | 94.33 | 125.04,107.04 | P-hydroxylbenzyl alcohol or its isomer | Phenolics | 61 | 623-05-2 |
| 57 | 6.74 | 127.0380 | [M+H]⁺ | C6H6O3 | -9.39 | 75.20 | 127.03,109.02,81.03,53.03 | 5-Hydroxymethyl-2-Furaldehyde or its isomer | Furan | 61 | 67-47-0 |
| 58 | 6.76 | 355.1032 | [M+H]⁺ | C16H18O9 | 3.30 | 84.76 | 355.10,192.04,163.03,145.02,117.03,89.03 | 1-O-Caffeoylquinic acid | Phenolics | 7 | 1241-87-8 |
| 59 | 6.83 | 286.1441 | [M+H]⁺ | C17H19NO3 | -0.49 | 87.94 | 286.14,201.09 | Morphine | Alkaloid | 85 | 57-27-2 |
| 60 | 7.13 | 127.0385 | [M+H]⁺ | C6H6O3 | 1.80 | 88.69 | 127.03,109.02,81.03,53.03 | Maltol | Heterocyclic phenols | 9,13,29,42,47,57,72,76,81,99 | 118-71-8 |
| 61 | 7.13 | 249.1955 | [M+H]⁺ | C15H24N2O | 0.76 | 92.86 | 249.19,148.11,112.07 | Matrine | Alkaloid | 32 | 519-02-8 |
| 62 | 7.49 | 247.1815 | [M+H]⁺ | C15H22N2O | -3.13 | 83.20 | 247.18,179.15,150.12,136.11,96.07 | Sophocarpine | Alkaloid | 9,28,29,32,56,61,73 | 6483-15-4 |
| 63 | 7.91 | 166.1226 | [M+H]⁺ | C10H15NO | -1.73 | 88.72 | 166.12,148.11,133.08,117.07,91.05,85.00,70.06 | Pseudoephedrine or its isomer | Alkaloid | 44,54,80,86,95 | 90-82-4 |
| 64 | 7.96 | 181.0721 | [M+H]⁺ | C7H8N4O2 | 0.23 | 79.26 | 181.07,163.06,124.05 | Theophylline | Alkaloid | 61 | 58-55-9 |
| 65 | 8.38 | 230.0814 | [M+H]⁺ | C13H11NO3 | 1.01 | 84.57 | 230.08,215.05,200.03,186.05,172.03 | Fagarine or its isomer | Alkaloid | 75 | 524-15-2 |
| 66 | 8.39 | 181.0390 | [M+H]⁺ | C9H8O4 | 2.06 | 94.48 | 181.04.163.03,145.02,135.04,117.03 | Caffeic acid or its isomer | Phenolics | 10,48,52,91 | 331-39-5 |
| 67 | 8.40 | 205.0970 | [M+H]⁺ | C11H12N2O2 | 0.19 | 93.03 | 205.09,188.07,146.05 | L-Tryptophan | Amino acid | 16,19,20,21,28,31,41,57,61,70,74,77,84,87,93 | 73-22-3 |
| 68 | 8.42 | 166.1220 | [M+H]⁺ | C10H15NO | 3.73 | 82.97 | 166.12,148.11,133.08,117.06,85.00 | Ephedrine | Alkaloid | 95 | 299-42-3 |
| 69 | 8.47 | 166.1224 | [M+H]⁺ | C10H15NO | -1.37 | 92.53 | 166.12,148.11,133.08,117.07,91.05,85.00,70.06 | Pseudoephedrine or its isomer | Alkaloid | 44 | 90-82-4 |
| 70 | 8.49 | 163.0380 | [M+H]⁺ | C9H6O3 | 5.74 | 90.97 | 163.03,145.02,117.03 | 7-Hydroxycoumarin or its isomer | Coumarins | 12,19,60 | 93-35-6 |
| 71 | 8.51 | 355.1018 | [M+H]⁺ | C16H18O9 | -1.10 | 97.19 | 355.10,192.04,163.03,145.02,117.03,89.03 | 1-O-Caffeoylquinic acid or its isomer | Phenolics | 7,60 | 1241-87-8 |
| 72 | 8.53 | 139.0388 | [M+H]⁺ | C7H6O3 | 0.39 | 89.71 | 139.03,111.04,93.03,65.03 | Protocatechuic aldehyde | Phenolics | 26,32 | 139-85-5 |
| 73 | 9.02 | 336.1799 | [M+H]⁺ | C18H25NO5 | 2.30 | 83.69 | 336.17,290.17,220.13 | Senecionine | Alkaloid | 98 | 130-01-8 |
| 74 | 9.17 | 291.0878 | [M+H]⁺ | C15H14O6 | -5.42 | 85.79 | 291.08,207.06,165.05,147.04,139.03 | (-)-Epicatechin or its isomer | Phenolics | 2 | 490-46-0 |
| 75 | 9.21 | 272.1295 | [M+H]⁺ | C16H17NO3 | -0.56 | 83.19 | 272.12,255.10 | Higenamine | Alkaloid | 16 | 5843-65-2 |
| 76 | 9.23 | 579.1722 | [M+H]⁺ | C27H30O14 | 1.36 | 94.64 | 579.17,433.11 | 2''-O-Rhamnosylvitexin | Flavonoids | 22 | 64820-99-1 |
| 77 | 9.25 | 330.1706 | [M+H]⁺ | C19H23NO4 | 3.88 | 79.25 | 330.16,270.14,239.067,213.09,181.06,58.06 | Sinomenine or its isomer | Alkaloid | 60 | 115-53-7 |
| 78 | 9.34 | 247.1440 | [M+H]⁺ | C14H18N2O2 | -0.44 | 98.19 | 247.14,188.07,146.06 | Hypaphorine | Alkaloid | 12,13,42,71,79,91 | 487-58-1 |
| 79 | 9.34 | 329.0868 | [M+H]⁺ | C14H16O9 | -0.70 | 91.76 | 329.08,293.06,209.04,181.04,159.04,133.02,103.03,85.02,57.03 | Bergenin | Phenolics | 16 | 477-90-7 |
| 80 | 9.35 | 163.0768 | [M+H]⁺ | C10H10O2 | -8.99 | 81.99 | 163.07,103.05,77.03,51.02 | Methyl cinnamate | Coumarins | 55 | 103-26-4 |
| 81 | 9.64 | 500.2850 | [M+H]⁺ | C25H41NO9 | 1.24 | 87.38 | 500.28,450.24,418.22,125.01 | Aconine | Alkaloid | 87,95 | 509-20-6 |
| 82 | 9.67 | 585.1444 | [M+H]⁺ | C25H28O16 | -0.66 | 78.40 | 585.14,567.12,549.13,519.10,489.09,465.09,435.08,405.08,369.05,327.04,303.04273.04 | Neomangiferin | Flavonoids | 89 | 64809-67-2 |
| 83 | 9.75 | 193.0858 | [M+H]⁺ | C11H12O3 | 0.75 | 88.41 | 193.08,161.06,133.06 | Methyl 4-Methoxycinnamate | Alkaloid | 16,31,73,88 | 3901-07-3 |
| 84 | 9.76 | 330.1701 | [M+H]⁺ | C19H23NO4 | 0.01 | 90.80 | 330.16,270.14,239.067,213.09,181.06,58.06 | Sinomenine or its isomer | Alkaloid | 66 | 115-53-7 |
| 85 | 9.77 | 314.1747 | [M+H]⁺ | C19H23NO3 | 0.45 | 92.91 | 314.17,283.13,189.09,107.04 | Armepavine or its isomer | Alkaloid | 55,89 | 524-20-9 |
| 86 | 9.77 | 360.2523 | [M+H]⁺ | C22H33NO3 | 2.32 | 88.06 | 360.25,342.23 | 12-Epinapelline | Alkaloid | 87,95 | 110064-71-6 |
| 87 | 9.80 | 163.0391 | [M+H]⁺ | C9H6O3 | 1.16 | 91.33 | 163.03,145.02,117.03 | 7-Hydroxycoumarin or its isomer | Coumarins | 16,25,55,60,87,95 | 93-35-6 |
| 88 | 9.80 | 181.0378 | [M+H]⁺ | C9H8O4 | 2.69 | 94.84 | 181.04.163.03,145.02,135.04,117.03 | Caffeic acid or its isomer | Phenolics | 5,7,10,20,29,33,41,42,47,72,91,93 | 331-39-5 |
| 89 | 9.81 | 395.1316 | [M+H]⁺ | C19H22O9 | -5.96 | 72.95 | 395.13,275.09,85.02 | Aloesin | Chromones | 1 | 30861-27-9 |
| 90 | 9.81 | 355.1013 | [M+H]⁺ | C16H18O9 | 3.59 | 95.97 | 355.10,192.04,163.03,145.02,117.03,89.03 | 1-O-Caffeoylquinic acid or its isomer | Phenolics | 5,10,13,16,20,23,25,33,40,41,42,44,52,55,60,61,72,87,89,91,95,98,100 | 1241-87-8 |
| 91 | 9.81 | 355.1011 | [M+H]⁺ | C16H18O9 | -2.39 | 94.73 | 355.10,229.88,163.03 | Chlorogenic Acid | Phenolics | 12 | 327-97-9 |
| 92 | 9.84 | 291.0857 | [M+H]⁺ | C15H14O6 | -0.63 | 79.83 | 291.08,139.03 | Catechin/(+)-Catechin | Phenolics | 9 | 154-23-4 |
| 93 | 9.86 | 291.0857 | [M+H]⁺ | C15H14O6 | 2.47 | 91.61 | 291.08,207.06,165.05,147.04,139.03 | (-)-Epicatechin or its isomer | Phenolics | 30,82 | 490-46-0 |
| 94 | 9.91 | 195.0866 | [M+H]⁺ | C8H10N4O2 | -7.69 | 79.74 | 195.08,167.06,138.06,110.07 | Caffeine or its isomer | Alkaloid | 10,61 | 58-08-2 |
| 95 | 9.93 | 433.1120 | [M+H]⁺ | C21H20O10 | 2.53 | 89.62 | 433.11,415.10,397.09,367.08,337.07,313.07 | Vitexin | Flavonoids | 22 | 3681-93-4 |
| 96 | 10.06 | 181.0382 | [M+H]⁺ | C9H8O4 | -2.60 | 93.92 | 181.04.163.03,145.02,135.04,117.03 | Caffeic acid or its isomer | Phenolics | 7,10,33,44,91,93 | 331-39-5 |
| 97 | 10.14 | 314.1751 | [M+H]⁺ | C19H23NO3 | 1.19 | 83.93 | 314.17,283.13,189.09,107.04 | Armepavine or its isomer | Alkaloid | 2,5,11,12,16,25,36,41,55,60,61,67,73,75,86,89,95 | 524-20-9 |
| 98 | 10.15 | 179.0332 | [M+H]⁺ | C9H6O4 | 4.26 | 86.39 | 179.03,123.04 | Daphentin | Coumarins | 39,44 | 486-35-1 |
| 99 | 10.17 | 355.1013 | [M+H]⁺ | C16H18O9 | -2.61 | 96.01 | 355.10,192.04,163.03,145.02,117.03,89.03 | 1-O-Caffeoylquinic acid or its isomer | Phenolics | 7,93 | 1241-87-8 |
| 100 | 10.26 | 195.0868 | [M+H]⁺ | C8H10N4O2 | -6.19 | 78.19 | 195.08,167.06,138.06,110.07 | Caffeine or its isomer | Alkaloid | 61 | 58-08-2 |
| 101 | 10.31 | 470.2739 | [M+H]⁺ | C24H39NO8 | 1.22 | 90.68 | 470.27,438.24 | Hypaconine | Alkaloid | 86,86,95 | 63238-68-6 |
| 102 | 10.36 | 425.1412 | [M+H]⁺ | C20H24O10 | 6.61 | 80.93 | 425.14,407.23 | Ginkgolide J | Terpenoids | 60,69,74,88,98 | 107438-79-9 |
| 103 | 10.42 | 209.0429 | [M+H]⁺ | C10H8O5 | -6.18 | 79.96 | 209.04,108.04 | Fraxetin or its isomer | Coumarins | 5 | 574-84-5 |
| 104 | 10.55 | 427.1198 | [M+Na]⁺ | C17H24O11 | -2.10 | 96.20 | 427.12,265.06 | Oleoside 11-methyl ester or its isomer | Iridoids | 79 | 60539-23-3 |
| 105 | 10.66 | 187.0387 | [M+H]⁺ | C11H6O3 | 0.55 | 91.60 | 187.03,159.04,143.04,131.04,115.05 | Isopsoralen or its isomer | Coumarins | 54 | 523-50-2 |
| 106 | 10.67 | 314.1380 | [M+H]⁺ | C18H19NO4 | 2.66 | 90.16 | 314.13,297.11,282.08,265.08,237.09,205.06 | Norisoboldine or its isomer | Alkaloid | 94,100 | 23599-69-1 |
| 107 | 10.68 | 483.1465 | [M+Na]⁺ | C20H28O12 | -1.64 | 92.41 | 483.15,317.08,189.05 | Apiopaeonoside | Phenolics | 1,92 | 100291-86-9 |
| 108 | 10.70 | 286.1437 | [M+H]⁺ | C17H19NO3 | 0.01 | 97.40 | 286.14,269.11,219.08,187.28,145.06,107.05 | Coclaurine or its isomer | Alkaloid | 12,25,56,66,70,72,94 | 2196-60-3 |
| 109 | 10.70 | 342.1702 | [M+H]⁺ | C20H23NO4 | 0.42 | 98.08 | 342.16,192.10 | Phellodendrine or its isomer | Alkaloid | 13,95 | 6873-13-8 |
| 110 | 10.75 | 149.0594 | [M+H]⁺ | C9H8O2 | -2.45 | 78.89 | 149.05,77.03 | 2-Chromanone | Coumarins | 29 | 119-84-6 |
| 111 | 10.79 | 417.1183 | [M+H]⁺ | C21H20O9 | 4.76 | 87.62 | 417.11,399.10,381.09,363.08,351.08,335.09,321.07,307.09,297.07,279.06,267.06 | Puerarin or its isomer | Isoflavone | 22,85 | 3681-99-0 |
| 112 | 10.81 | 469.1667 | [M+H]⁺ | C22H28O11 | 7.41 | 75.87 | 469.16,307.11 | Prim-O-glucosylcimifugin or its isomer | Chromones | 59 | 80681-45-4 |
| 113 | 10.83 | 389.1441 | [M+H]⁺ | C17H24O10 | -0.41 | 95.62 | 389.14,227.09,209.08,195.06,177.05,167.07,149.05,121.06,85.02,69.03 | Verbenalin | Iridoids | 16 | 548-37-8 |
| 114 | 10.84 | 193.0491 | [M+H]⁺ | C10H8O4 | 2.08 | 81.62 | 193.05,165.04 | Scopoletin or its isomer | Alkaloid | 13 | 92-61-5 |
| 115 | 10.84 | 342.1699 | [M+H]⁺ | C20H23NO4 | 1.05 | 99.59 | 342.17,192.10 | Isocorypalmine or its isomer | Alkaloid | 60 | 483-34-1 |
| 116 | 10.87 | 423.0930 | [M+H]⁺ | C19H18O11 | 0.65 | 75.75 | 423.09,303.04,273.04 | Mangiferin | Flavonoids | 14 | 4773-96-0 |
| 117 | 10.87 | 342.1715 | [M+H]⁺ | C20H23NO4 | -3.02 | 96.37 | 342.16,178.08 | Tetrahydrojateorrhizine or its isomer | Alkaloid | 16 | 13063-54-2 |
| 118 | 10.88 | 328.1540 | [M+H]⁺ | C19H21NO4 | -0.93 | 95.68 | 328.15,297.10,265.08,237.09,205.06 | Boldine or its isomer | Alkaloid | 22,41,85,86,94,100 | 476-70-0 |
| 119 | 10.88 | 335.1756 | [M+H]⁺ | C21H22N2O2 | -0.81 | 97.24 | 335.17,307.14,289.13,264.10,222.09,184.07,156.08,129.06,108.08,79.05,56.05 | Strychnine | Alkaloid | 95 | 57-24-9 |
| 120 | 10.90 | 314.1380 | [M+H]⁺ | C18H19NO4 | 2.93 | 94.10 | 314.13,297.11,282.08,265.08,237.09,205.06 | Norisoboldine or its isomer | Alkaloid | 94 | 23599-69-1 |
| 121 | 11.00 | 291.0853 | [M+H]⁺ | C15H14O6 | -2.40 | 82.61 | 291.08,207.06,165.05,147.04,139.03 | (-)-Epicatechin or its isomer | Phenolics | 3 | 490-46-0 |
| 122 | 11.02 | 447.1274 | [M+H]⁺ | C22H22O10 | 2.30 | 87.05 | 447.12,429.11,411.10,393.09,381.09,365.10,351.08,337.10,327.08,309.07 | 3'-Methoxypuerarin or its isomer | Isoflavone | 22 | 117047-07-1 |
| 123 | 11.07 | 481.1701 | [M+H]⁺ | C23H28O11 | 1.06 | 96.04 | 481.16,319.11,301.10,267.08,197.08,179.07,151.07,133.06,121.06,105.03,85.02,69.03 | Albiflorin or its isomer | Terpenoids | 16,40,69,70,86,90,94 | 39011-90-0 |
| 124 | 11.11 | 595.1660 | [M+H]⁺ | C27H30O15 | 0.37 | 86.10 | 595.16,577.15,559.14,529.13,511.12,475.12,457.11,427.11,409.09,379.08,229.13,541.13,511.12,475.11,457.11,427.10,409.09,379.08,356.08,325.06 | Vicenin II | Flavonoids | 54 | 23666-13-9 |
| 125 | 11.19 | 209.0435 | [M+H]⁺ | C10H8O5 | -3.81 | 81.79 | 209.04,108.04 | Fraxetin or its isomer | Coumarins | 5 | 574-84-5 |
| 126 | 11.27 | 181.0383 | [M+H]⁺ | C9H8O4 | 2.72 | 88.83 | 181.04.163.03,145.02,135.04,117.03 | Caffeic acid or its isomer | Phenolics | 5 | 331-39-5 |
| 127 | 11.32 | 153.0556 | [M+H]⁺ | C8H8O3 | 3.69 | 71.83 | 153.05,65.03 | Vanillin | Phenolics | 5 | 121-33-5 |
| 128 | 11.34 | 153.0546 | [M+H]⁺ | C8H8O3 | -3.54 | 82.82 | 153.05,93.03,65.03 | Isovanillin | Phenolics | 26 | 621-59-0 |
| 129 | 11.36 | 319.0447 | [M+H]⁺ | C15H10O8 | 1.44 | 90.64 | 319.04,301.03,273.03,245.04,217.04,179.03,165.01,153.01,137.02 | Myricetin or its isomer | Flavonoids | 67 | 529-44-2 |
| 130 | 11.44 | 342.1695 | [M+H]⁺ | C20H23NO4 | 2.63 | 90.91 | 342.16,192.10 | Phellodendrine or its isomer | Alkaloid | 55 | 6873-13-8 |
| 131 | 11.46 | 369.1176 | [M+H]⁺ | C17H20O9 | 1.32 | 97.00 | 369.11,177.05,145.02 | 5-Feruloylquinic acid | Phenolics | 16,60,72 | 40242-06-6 |
| 132 | 11.46 | 342.1702 | [M+H]⁺ | C20H24NO4 | -5.12 | 86.33 | 342.17,297.11,265.08,237.09 | Magnoflorine or its isomer | Alkaloid | 72 | 2141-09-5 |
| 133 | 11.54 | 447.1251 | [M+H]⁺ | C22H22O10 | 3.01 | 81.60 | 447.12,429.11,411.10,393.09,381.09,365.10,351.08,337.10,327.08,309.07 | 3'-Methoxypuerarin or its isomer | Isoflavone | 71 | 117047-07-1 |
| 134 | 11.54 | 595.1632 | [M+H]⁺ | C27H30O15 | 5.40 | 70.33 | 595.16,433.11 | Vitexin -4''-O-glucoside | Flavonoids | 80 | 178468-00-3 |
| 135 | 11.57 | 427.1230 | [M+Na]⁺ | C17H24O11 | -2.32 | 86.66 | 427.12,265.06 | Oleoside 11-methyl ester or its isomer | Iridoids | 33,100 | 60539-23-3 |
| 136 | 11.59 | 161.0598 | [M+H]⁺ | C10H8O2 | 0.44 | 87.16 | 161.05,133.06,117.06,105.07,91.05 | 6-Methylcoumarin | Coumarins | 25 | 92-48-8 |
| 137 | 11.62 | 344.2585 | [M+H]⁺ | C22H33NO2 | 1.42 | 90.97 | 344.25,327.23,221.14,177.11,133.08 | Bullatine A or its isomer | Alkaloid | 75,86,87,95 | 1354-84-3 |
| 138 | 11.63 | 330.1693 | [M+H]⁺ | C19H23NO4 | 2.85 | 71.42 | 330.16,299.12,287.12,267.10,192.10,175.07,143.04 | Sinomenine or its isomer | Alkaloid | 94 | 115-53-7 |
| 139 | 11.63 | 161.0595 | [M+H]⁺ | C10H8O2 | -2.82 | 81.90 | 161.05,117.06 | 7-Methylcoumarin | Coumarins | 41,65 | 2445-83-2 |
| 140 | 11.66 | 303.0496 | [M+H]⁺ | C15H10O7 | 2.57 | 91.44 | 303.04,285.02,257.04,229.04,201.05,165.01,153.01,137.02 | Quercetin or its isomer | Flavonoids | 31,74 | 117-39-5 |
| 141 | 11.67 | 565.1544 | [M+H]⁺ | C26H28O14 | -0.62 | 87.78 | 565.15,547.14,529.13,511.12,475.12,427.10,409.09 | Vicenin -1 | Flavonoids | 10,54,89 | 35927-38-9 |
| 142 | 11.71 | 187.0386 | [M+H]⁺ | C11H6O3 | 1.79 | 97.58 | 187.03,159.04,143.04,131.04,115.05 | Isopsoralen or its isomer | Coumarins | 89 | 523-50-2 |
| 143 | 11.71 | 255.0645 | [M+H]⁺ | C15H10O4 | 5.01 | 82.01 | 255.06,237.05,227.07,199.07,181.06,171.08,153.06,137.02 | Daidzein or its isomer | Isoflavone | 22 | 486-66-8 |
| 144 | 11.73 | 407.1333 | [M+H]⁺ | C20H22O9 | 1.88 | 88.53 | 407.13,245.08,227.06,199.07,151.03,121.06 | (2S,3R,4S,5S,6R)-2-[2,4-dihydroxy-6-[(E)-2-(4-hydroxyphenyl)vinyl]phenoxy]-6-(hydroxymethyl)tetrahydropyran-3,4,5-triol or its isomer | Stilbene glycosides | 12 | 82373-94-2 |
| 145 | 11.73 | 342.1715 | [M+H]⁺ | C20H23NO4 | -0.35 | 86.91 | 342.16,192.10 | Phellodendrine or its isomer | Alkaloid | 16 | 6873-13-8 |
| 146 | 11.75 | 469.1695 | [M+H]⁺ | C22H28O11 | 0.23 | 93.47 | 469.16,307.11 | Prim-O-glucosylcimifugin or its isomer | Chromones | 22,86 | 80681-45-4 |
| 147 | 11.76 | 465.1014 | [M+H]⁺ | C21H20O12 | 0.02 | 92.60 | 465.10,303.04 | Quercetin-7-O-β-D-glucopyranoside or its isomer | Flavonoids | 31 | 491-50-9 |
| 148 | 11.80 | 611.1602 | [M+H]⁺ | C27H30O16 | -2.32 | 87.74 | 611.16,303.04 | Rutin or its isomer | Flavonoids | 31 | 153-18-4 |
| 149 | 11.84 | 625.1769 | [M+H]⁺ | C28H32O16 | 2.71 | 79.05 | 625.17,317.06,85.02,71.04 | Isorhamnetin-3-O-neohespeidoside or its isomer | Flavonoids | 67 | 55033-90-4 |
| 150 | 11.87 | 447.1268 | [M+H]⁺ | C22H22O10 | 4.06 | 87.36 | 447.12,429.11,411.10,393.09,381.09,365.10,351.08,337.10,327.08,309.07 | 3'-Methoxypuerarin or its isomer | Isoflavone | 90 | 117047-07-1 |
| 151 | 11.91 | 312.1553 | [M+H]⁺ | C14H21N3O5 | -0.11 | 93.28 | 312.15,181.04,132.11,114.10,97.07,72.08 | Leonurine | Alkaloid | 12,20,80,87,94 | 24697-74-3 |
| 152 | 11.92 | 319.0447 | [M+H]⁺ | C15H10O8 | -0.32 | 94.43 | 319.04,301.03,273.03,245.04,217.04,179.03,165.01,153.01,137.02 | Myricetin or its isomer | Flavonoids | 31,39,67 | 529-44-2 |
| 153 | 11.95 | 765.2592 | [M+Na]⁺ | C34H46O18 | 2.05 | 86.26 | 765.25,605.17 | Eleutheroside E | Lignans | 31,55,86 | 39432-56-9 |
| 154 | 11.95 | 314.1747 | [M+H]⁺ | C19H23NO3 | 0.56 | 92.75 | 314.17,283.13,189.09,107.04 | Armepavine or its isomer | Alkaloid | 2,16,22,25,55,60,72,89,95 | 524-20-9 |
| 155 | 11.95 | 193.0491 | [M+H]⁺ | C10H8O4 | 1.75 | 95.86 | 193.04,178.02,165.05,150.03,133.02 | Isoscopoletin or its isomer | Coumarins | 39 | 776-86-3 |
| 156 | 11.96 | 481.0969 | [M+H]⁺ | C21H20O13 | 1.33 | 95.68 | 481.09,319.04,290.03,237.11,145.04,85.02 | Myricetin 3-O-β-D-glucopyranoside | Flavonoids | 67 | 19833-12-6 |
| 157 | 11.97 | 418.1581 | [M+H]⁺ | C22H26O8 | -6.01 | 73.93 | 418.15,383.14,330.10,217.09,167.07 | (-)-Syringaresinol | Lignans | 91 | 1177-14-6 |
| 158 | 12.00 | 328.1548 | [M+H]⁺ | C19H21NO4 | 2.84 | 86.49 | 328.15,297.10,265.08,237.09,205.06 | Boldine or its isomer | Alkaloid | 72 | 476-70-0 |
| 159 | 12.03 | 447.1291 | [M+H]⁺ | C22H22O10 | 1.64 | 93.41 | 447.12,285.07 | Tilianin or its isomer | Flavonoids | 11 | 4291-60-5 |
| 160 | 12.07 | 481.0965 | [M+H]⁺ | C21H20O13 | 2.82 | 92.90 | 481.09,319.04,85.02 | Myricetin 3-O-galactoside | Flavonoids | 31,39 | 15648-86-9 |
| 161 | 12.07 | 319.1166 | [M+H]⁺ | C17H18O7 | 2.15 | 92.53 | 319.11,301.10,283.09,255.10,227.10,210.05,192.04,164.04,129.07,105.06 | Isoagarotetrol or its isomer | Terpenoids | 94 | 104060-61-9 |
| 162 | 12.07 | 163.0384 | [M+H]⁺ | C9H6O3 | -4.26 | 80.55 | 163.03,145.02,117.03 | 7-Hydroxycoumarin or its isomer | Coumarins | 5,42,45,61,86 | 93-35-6 |
| 163 | 12.10 | 303.0496 | [M+H]⁺ | C15H10O7 | 1.92 | 92.66 | 303.04,285.02,257.04,229.04,201.05,165.01,153.01,137.02 | Quercetin or its isomer | Flavonoids | 44,67,74 | 117-39-5 |
| 164 | 12.10 | 344.2585 | [M+H]⁺ | C22H33NO2 | 0.80 | 88.80 | 344.25,327.23,221.14,177.11,133.08 | Bullatine A or its isomer | Alkaloid | 75 | 1354-84-3 |
| 165 | 12.13 | 465.1022 | [M+H]⁺ | C21H20O12 | 2.26 | 93.82 | 465.10,303.05 | Isoquercitrin or its isomer | Flavonoids | 67 | 21637-25-2 |
| 166 | 12.17 | 356.1857 | [M+H]⁺ | C21H25NO4 | 0.59 | 98.96 | 356.18,192.10 | Tetrahydropalmatine or its isomer | Alkaloid | 16,60,72 | 2934-97-6 |
| 167 | 12.18 | 597.1468 | [M+H]⁺ | C26H28O16 | -1.10 | 90.20 | 597.14,465.10,303.04 | Quercetin-3-O-β-D-ribosyl-(1→2)-β-D-glucoside | Flavonoids | 74 | 83048-35-5 |
| 168 | 12.18 | 611.3122 | [M+H]⁺ | C37H42N2O6 | 1.05 | 97.92 | 611.31,551.24,192.10 | (+)-Isoliensinine or its isomer | Alkaloid | 66 | 6817-41-0 |
| 169 | 12.19 | 187.0386 | [M+H]⁺ | C11H6O3 | -0.58 | 90.45 | 187.04,159.04,143.04,131.04,115.05 | Psoralen or its isomer | Coumarins | 5,42,86,95 | 66-97-7 |
| 170 | 12.26 | 187.0388 | [M+H]⁺ | C11H6O3 | 1.00 | 85.98 | 187.03,159.04,143.04,131.04,115.05 | Isopsoralen or its isomer | Coumarins | 61 | 523-50-2 |
| 171 | 12.28 | 181.0382 | [M+H]⁺ | C9H8O4 | -0.91 | 93.96 | 181.04.163.03,145.02,135.04,117.03 | Caffeic acid or its isomer | Phenolics | 7,9,10,11 | 331-39-5 |
| 172 | 12.29 | 287.0543 | [M+H]⁺ | C15H10O6 | 1.81 | 94.36 | 287.05,153.01 | Luteolin or its isomer | Flavonoids | 31,74 | 491-70-3 |
| 173 | 12.30 | 342.1700 | [M+H]⁺ | C20H23NO4 | 0.27 | 98.97 | 342.17,192.10 | Isocorypalmine or its isomer | Alkaloid | 41,55 | 483-34-1 |
| 174 | 12.31 | 449.1064 | [M+H]⁺ | C21H20O11 | 2.39 | 95.23 | 449.10,287.05 | Luteolin-7-O-β-D-glucoside or its isomer | Flavonoids | 31 | 5373-11-5 |
| 175 | 12.31 | 592.3843 | [M+H]⁺ | C33H53NO8 | -1.21 | 88.23 | 592.38,575.17,430.32 | Sipeimine-3-β-D-glucoside | Alkaloid | 48,90 | 67968-40-5 |
| 176 | 12.31 | 430.3295 | [M+H]⁺ | C27H43NO3 | -4.97 | 87.55 | 430.32,412.32,138.12 | Sipeimine or its isomer | Alkaloid | 48 | 61825-98-7 |
| 177 | 12.34 | 625.3306 | [M+H]⁺ | C38H44N2O6 | 0.23 | 97.24 | 625.32,206.11 | Neferine | Alkaloid | 66 | 2292-16-2 |
| 178 | 12.36 | 193.0493 | [M+H]⁺ | C10H8O4 | -0.13 | 86.81 | 193.05,165.04 | Scopoletin or its isomer | Alkaloid | 9,13,20,39,41,45,60,67 | 92-61-5 |
| 179 | 12.37 | 193.0492 | [M+H]⁺ | C10H8O4 | -1.64 | 91.97 | 193.04,178.02,165.05,150.03,133.02 | Isoscopoletin or its isomer | Coumarins | 5,11,16,28,29,40,44,52,54,61,79,86,87,90,91,98 | 776-86-3 |
| 180 | 12.38 | 317.0645 | [M+H]⁺ | C16H12O7 | 2.13 | 94.44 | 317.06,302.04,285.03,257.04,229.04,139.03 | Isorhamnetin or its isomer | Flavonoids | 31 | 480-19-3 |
| 181 | 12.39 | 479.1163 | [M+H]⁺ | C22H22O12 | 0.73 | 89.17 | 479.11,317.06 | Isorhamnetin -3-O-b-D-galactoside or its isomer | Flavonoids | 31 | 6743-92-6 |
| 182 | 12.41 | 417.1196 | [M+H]⁺ | C21H20O9 | -3.19 | 82.46 | 417.11,255.06 | Daidzin/Daidzoside or its isomer | Isoflavone | 22,89 | 552-66-9 |
| 183 | 12.42 | 163.0385 | [M+H]⁺ | C9H6O3 | 1.76 | 94.05 | 163.03,145.02,117.03 | 7-Hydroxycoumarin or its isomer | Coumarins | 14 | 93-35-6 |
| 184 | 12.45 | 203.0340 | [M+H]⁺ | C11H6O4 | -0.72 | 89.15 | 203.03,175.03,159.04,147.04,131.05,91.05 | Xanthotoxol or its isomer | Coumarins | 95 | 2009-24-7 |
| 185 | 12.50 | 326.1397 | [M+H]⁺ | C19H19NO4 | 3.17 | 84.39 | 326.13,178.08 | Cheilanthifoline | Alkaloid | 41 | 483-44-3 |
| 186 | 12.51 | 376.2478 | [M+H]⁺ | C22H33NO4 | 1.70 | 77.49 | 376.24,330.24,302.21,276.19,211.14,187.14,159.11,133.10,96.08,70.06,55.01 | Neotuberostemonine or its isomer | Alkaloid | 98 | 143120-46-1 |
| 187 | 12.53 | 447.1277 | [M+H]⁺ | C22H22O10 | 1.04 | 98.85 | 447.12,285.07 | Tilianin or its isomer | Flavonoids | 28,29,71 | 4291-60-5 |
| 188 | 12.53 | 342.1715 | [M+H]⁺ | C20H23NO4 | -1.98 | 94.45 | 342.16,192.10 | Phellodendrine or its isomer | Alkaloid | 13,16 | 6873-13-8 |
| 189 | 12.55 | 342.1694 | [M+H]⁺ | C20H23NO4 | -0.27 | 85.64 | 342.16,311.12,279.09 | Isocorydine | Alkaloid | 41 | 475-67-2 |
| 190 | 12.56 | 271.0594 | [M+H]⁺ | C15H10O5 | -2.70 | 90.28 | 271.05,253.04,225.05,197.05,169.06,141.06,121.02 | Aloeemodin | Anthraquinone | 3,63,92,93 | 481-72-1 |
| 191 | 12.58 | 289.0709 | [M+H]⁺ | C15H12O6 | 2.09 | 77.78 | 289.07,163.03 | Eriodictyol or its isomer | Flavonoids | 40 | 552-58-9 |
| 192 | 12.58 | 303.0501 | [M+H]⁺ | C15H10O7 | 0.05 | 89.82 | 303.04,257.04 | Morin or its isomer | Flavonoids | 10,11 | 480-16-0 |
| 193 | 12.59 | 609.1808 | [M+H]⁺ | C28H32O15 | 0.20 | 93.66 | 609.18,489.13,447.12,429.11,411.10,375.08,351.08,327.08,297.07,85.02 | Spinosin | Flavonoids | 13,25,56,70 | 72063-39-9 |
| 194 | 12.59 | 611.1595 | [M+H]⁺ | C27H30O16 | 1.64 | 94.97 | 611.16,303.04 | Rutin or its isomer | Flavonoids | 31,67 | 153-18-4 |
| 195 | 12.59 | 257.0798 | [M+H]⁺ | C15H12O4 | -3.70 | 96.60 | 257.08,239.06,211.07,163.03,147.04,137.02 | Isoliquiritigenin or its isomer | Chalcones | 1,5,9,11,12,16,28,36,40,44,47,56,61,63,69,72,77,86,92,95 | 961-29-5 |
| 196 | 12.60 | 303.0499 | [M+H]⁺ | C15H10O7 | 0.52 | 95.27 | 303.04,285.02,257.04,229.04,201.05,165.01,153.01,137.02 | Quercetin or its isomer | Flavonoids | 31,67,96 | 117-39-5 |
| 197 | 12.61 | 286.1444 | [M+H]⁺ | C17H19NO3 | 2.26 | 85.45 | 286.14,269.11,219.08,187.28,145.06,107.05 | Coclaurine or its isomer | Alkaloid | 22,72,94 | 2196-60-3 |
| 198 | 12.64 | 611.3122 | [M+H]⁺ | C37H42N2O6 | -0.93 | 98.60 | 611.31,551.24,192.10 | (+)-Isoliensinine or its isomer | Alkaloid | 66 | 6817-41-0 |
| 199 | 12.73 | 433.1128 | [M+H]⁺ | C21H20O10 | -0.07 | 86.49 | 433.11,415.10,397.09,379.08,367.08,337.07,313.07,283.06 | Isovitexin | Flavonoids | 38,80 | 29702-25-8 |
| 200 | 12.74 | 223.0603 | [M+H]⁺ | C11H10O5 | 0.15 | 92.18 | 223.06,208.02,190.02,179.03,163.04,146.10,78.04,59.04 | Isofraxidin or its isomer | Coumarins | 13,16,28,40,47,54,55,87 | 486-21-5 |
| 201 | 12.75 | 319.0442 | [M+H]⁺ | C15H10O8 | 3.29 | 74.26 | 319.04,301.03,273.03,245.04,217.04,179.03,165.01,153.01,137.02 | Myricetin or its isomer | Flavonoids | 59 | 529-44-2 |
| 202 | 12.75 | 257.0807 | [M+H]⁺ | C15H12O4 | 2.46 | 78.70 | 257.07,137.02 | Pinocembrin or its isomer | Flavonoids | 94 | 480-39-7 |
| 203 | 12.77 | 376.2478 | [M+H]⁺ | C22H33NO4 | 1.96 | 83.23 | 376.24,330.24,302.21,276.19,211.14,187.14,159.11,133.10,96.08,70.06,55.01 | Neotuberostemonine or its isomer | Alkaloid | 98 | 143120-46-1 |
| 204 | 12.78 | 319.1166 | [M+H]⁺ | C17H18O7 | 3.23 | 92.82 | 319.11,301.10,283.09,255.10,227.10,210.05,192.04,164.04,129.07,105.06 | Isoagarotetrol or its isomer | Terpenoids | 94 | 104060-61-9 |
| 205 | 12.81 | 463.1216 | [M+H]⁺ | C22H22O11 | 0.39 | 99.90 | 463.12,301.06 | Diosmetin-7-O-β-D-glucopyranoside or its isomer | Flavonoids | 93 | 20126-59-4 |
| 206 | 12.81 | 481.3155 | [M+H]⁺ | C27H44O7 | 2.04 | 78.79 | 481.31,445.29,427.28,409.26,371.21,303.19,165.12,99.07 | Hydroxyecdysone | Steroids | 40 | 5289-74-7 |
| 207 | 12.84 | 289.0709 | [M+H]⁺ | C15H12O6 | 1.60 | 81.48 | 289.07,163.03 | Eriodictyol or its isomer | Flavonoids | 40 | 552-58-9 |
| 208 | 12.84 | 465.1022 | [M+H]⁺ | C21H20O12 | 1.18 | 99.24 | 465.10,303.05 | Isoquercitrin or its isomer | Flavonoids | 39,67 | 21637-25-2 |
| 209 | 12.85 | 481.3152 | [M+H]⁺ | C27H44O7 | 1.91 | 85.64 | 481.31,463.30,445.29,427.28,410.27,391.26,347.22,303.19,143.10 | 25R-Inokosterone | Steroids | 75 | 19595-18-7 |
| 210 | 12.92 | 181.0386 | [M+H]⁺ | C9H8O4 | 2.01 | 94.36 | 181.04.163.03,145.02,135.04,117.03 | Caffeic acid or its isomer | Phenolics | 9,11,47 | 331-39-5 |
| 211 | 12.93 | 407.1339 | [M+H]⁺ | C20H22O9 | 2.06 | 92.00 | 407.13,245.08,227.06,199.07,151.03,121.06 | (2S,3R,4S,5S,6R)-2-[2,4-dihydroxy-6-[(E)-2-(4-hydroxyphenyl)vinyl]phenoxy]-6-(hydroxymethyl)tetrahydropyran-3,4,5-triol or its isomer | Stilbene glycosides | 42 | 82373-94-2 |
| 212 | 12.94 | 287.0550 | [M+H]⁺ | C15H10O6 | -1.22 | 86.77 | 287.05,258.04,165.01,153.01,68.99 | Kaempferol or its isomer | Flavonoids | 74 | 520-18-3 |
| 213 | 12.94 | 273.0754 | [M+H]⁺ | C15H12O5 | 4.20 | 73.77 | 273.07,231.06,153.01 | Naringenin chalcone or its isomer | Chalcones | 40 | 73692-50-9 |
| 214 | 12.94 | 303.0499 | [M+H]⁺ | C15H10O7 | 0.03 | 96.59 | 303.04,285.02,257.04,229.04,201.05,165.01,153.01,137.02 | Quercetin or its isomer | Flavonoids | 31,39,59,67 | 117-39-5 |
| 215 | 12.96 | 305.0655 | [M+H]⁺ | C15H12O7 | 0.63 | 91.76 | 305.06,287.05,259.06,231.06,195.02,179.03,167.03,153.01,123.04 | Taxifolin or its isomer | Flavonoids | 16 | 480-18-2 |
| 216 | 12.96 | 354.1335 | [M+H]⁺ | C20H19NO5 | 0.50 | 78.50 | 354.13,336.12,323.09,305.08,275.07 | Chelidonine or its isomer | Alkaloid | 55 | 476-32-4 |
| 217 | 12.96 | 417.1177 | [M+H]⁺ | C21H20O9 | 1.35 | 88.03 | 417.11,399.10,381.09,363.08,351.08,335.09,321.07,307.09,297.07,279.06,267.06 | Puerarin or its isomer | Isoflavone | 22,91 | 3681-99-0 |
| 218 | 12.96 | 307.1173 | [M+H]⁺ | C16H18O6 | 0.28 | 89.03 | 307.11,289.10,259.05,207.06,177.05 | Cimifugin | Stilbene glycosides | 86 | 37921-38-3 |
| 219 | 12.97 | 463.0865 | [M+H]⁺ | C21H18O12 | -0.44 | 96.94 | 463.08,287.05,211.08 | Scutellarin B | Flavonoids | 3,5,7,10,11,52 | 27740-01-8 |
| 220 | 13.00 | 463.1209 | [M+H]⁺ | C22H22O11 | 7.20 | 76.85 | 463.12,445.11,427.09,409.09,397.08,367.08,343.08,313.06 | Isoscoparin | Flavonoids | 80 | 20013-23-4 |
| 221 | 13.01 | 463.0856 | [M+H]⁺ | C21H18O12 | 2.69 | 94.64 | 463.08,287.05,153.01 | Luteolin-3-D-glucuronide | Flavonoids | 2,16,55,81,100 | 53527-42-7 |
| 222 | 13.02 | 463.0866 | [M+H]⁺ | C21H18O12 | -1.23 | 92.56 | 463.08,287.20 | Luteolin-7-O-β-D-glucuronide or its isomer | Flavonoids | 1 | 29741-10-4 |
| 223 | 13.02 | 449.1066 | [M+H]⁺ | C21H20O11 | -1.28 | 95.86 | 449.10,287.05 | Kaempferol 7-O-β-D-glucoside or its isomer | Flavonoids | 7,33,52 | 16290-07-6 |
| 224 | 13.02 | 317.0656 | [M+H]⁺ | C16H12O7 | 0.00 | 87.24 | 317.06,302.04,285.03,257.04,229.04,201.05,153.01,139.03 | Isorhamnetin or its isomer | Flavonoids | 74 | 480-19-3 |
| 225 | 13.02 | 163.0382 | [M+H]⁺ | C9H6O3 | 6.83 | 79.74 | 163.03,145.02,117.03 | 7-Hydroxycoumarin or its isomer | Coumarins | 32,65 | 93-35-6 |
| 226 | 13.05 | 625.3306 | [M+H]⁺ | C38H44N2O6 | -3.31 | 90.16 | 625.32,582.28 | Dauricine | Alkaloid | 66 | 524-17-4 |
| 227 | 13.07 | 465.1014 | [M+H]⁺ | C21H20O12 | 5.29 | 89.72 | 465.10,303.04 | Quercetin-7-O-β-D-glucopyranoside or its isomer | Flavonoids | 31,59 | 491-50-9 |
| 228 | 13.12 | 247.0950 | [M+H]⁺ | C14H14O4 | -6.38 | 83.55 | 247.09,229.08,175.04, | Nodakenitin | Coumarins | 14,29,86 | 495-32-9 |
| 229 | 13.13 | 433.1103 | [M+H]⁺ | C21H20O10 | -4.65 | 93.86 | 433.11,271.06 | Genistin | Flavonoids | 91 | 529-59-9 |
| 230 | 13.15 | 247.0961 | [M+H]⁺ | C14H14O4 | -0.42 | 83.78 | 247.09,229.08,175.03 | Decursinol or its isomer | Coumarins | 14,54,95 | 23458-02-8 |
| 231 | 13.15 | 419.1337 | [M+H]⁺ | C21H22O9 | 1.04 | 88.63 | 419.13,257.08 | Pinocembrin 7-O-bate-D-glucoside or its isomer | Flavonoids | 82,92,93 | 75829-43-5 |
| 232 | 13.15 | 376.2478 | [M+H]⁺ | C22H33NO4 | 0.80 | 93.09 | 376.24,330.24,302.21,276.19,211.14,187.14,159.11,133.10,96.08,70.06,55.01 | Neotuberostemonine or its isomer | Alkaloid | 98 | 143120-46-1 |
| 233 | 13.16 | 247.0961 | [M+H]⁺ | C14H14O4 | 0.93 | 93.32 | 247.09,229.08,175.03 | Columbianetin or its isomer | Coumarins | 5,42,44,52,61,86,95 | 3804-70-4 |
| 234 | 13.16 | 342.1700 | [M+H]⁺ | C20H23NO4 | 2.44 | 94.31 | 342.16,178.08 | Tetrahydrojateorrhizine or its isomer | Alkaloid | 94 | 13063-54-2 |
| 235 | 13.17 | 611.3122 | [M+H]⁺ | C37H42N2O6 | -1.53 | 95.86 | 611.31,551.24,192.10 | (+)-Isoliensinine or its isomer | Alkaloid | 66 | 6817-41-0 |
| 236 | 13.18 | 433.1120 | [M+H]⁺ | C21H20O10 | 0.73 | 95.34 | 433.11,415.10,271.06,253.04,123.00 | Oroxin A or its isomer | Flavonoids | 55 | 57396-78-8 |
| 237 | 13.19 | 419.1353 | [M+H]⁺ | C21H22O9 | -4.86 | 82.88 | 419.13,257.08,137.02 | Isoliquiritin or its isomer | Chalcones | 2 | 5041-81-6 |
| 238 | 13.20 | 481.0969 | [M+H]⁺ | C21H20O13 | 1.11 | 96.40 | 481.09,319.04,145.04 | Myricetin 3'-O-β-D-glucopyranoside | Flavonoids | 67 | 520-14-9 |
| 239 | 13.22 | 433.1128 | [M+H]⁺ | C21H20O10 | -0.19 | 93.65 | 433.11,271.06 | Apigenin-7-glucoside or its isomer | Flavonoids | 22 | 578-74-5 |
| 240 | 13.22 | 409.1498 | [M+H]⁺ | C20H24O9 | 0.80 | 94.78 | 409.14,247.09,229.08,201.09,187.03,159.04,127.03,97.02,85.02 | Nodakenin | Coumarins | 42,86,95 | 495-31-8 |
| 241 | 13.25 | 592.3839 | [M+H]⁺ | C33H53NO8 | 1.79 | 90.54 | 592.38,574.37,178.88 | SipeiMine-3β-D-glucoside | Alkaloid | 32 | 32685-93-1 |
| 242 | 13.26 | 303.0496 | [M+H]⁺ | C15H10O7 | 4.61 | 72.42 | 303.04,285.02,257.04,229.04,201.05,165.01,153.01,137.02,121.02 | Quercetin or its isomer | Flavonoids | 31,39 | 117-39-5 |
| 243 | 13.30 | 322.1049 | [M+H]⁺ | C19H15NO4 | 5.62 | 86.32 | 322.10,307.08,294.07,279.08 | Berberrubine or its isomer | Alkaloid | 22,25,72 | 17388-19-1 |
| 244 | 13.31 | 356.1849 | [M+H]⁺ | C21H25NO4 | 3.22 | 86.85 | 356.18,341.16,308.12,192.10,165.09 | D-Tetrahydropalmatine | Alkaloid | 94 | 3520-14-7 |
| 245 | 13.32 | 342.1711 | [M+H]⁺ | C20H23NO4 | 2.20 | 91.87 | 342.17,192.10 | Isocorypalmine or its isomer | Alkaloid | 72 | 483-34-1 |
| 246 | 13.36 | 223.0601 | [M+H]⁺ | C11H10O5 | -0.07 | 77.59 | 223.06,208.02,190.02,179.03,163.04,149.02,107.04,89.05,78.04,59.04 | Isofraxidin or its isomer | Coumarins | 5 | 486-21-5 |
| 247 | 13.36 | 430.3315 | [M+H]⁺ | C27H43NO3 | -0.23 | 94.68 | 430.33,412.32 | Peiminine or its isomer | Alkaloid | 90 | 18059-10-4 |
| 248 | 13.40 | 344.2585 | [M+H]⁺ | C22H33NO2 | -6.03 | 75.34 | 344.25,327.23,221.14,177.11,133.08 | Bullatine A or its isomer | Alkaloid | 75 | 1354-84-3 |
| 249 | 13.45 | 273.0754 | [M+H]⁺ | C15H12O5 | 2.64 | 90.72 | 273.07,231.06,153.01 | Naringenin chalcone or its isomer | Chalcones | 40 | 73692-50-9 |
| 250 | 13.47 | 356.1857 | [M+H]⁺ | C21H25NO4 | 0.77 | 95.24 | 356.18,192.10 | Tetrahydropalmatine or its isomer | Alkaloid | 16,55,60,94,95 | 2934-97-6 |
| 251 | 13.47 | 287.0543 | [M+H]⁺ | C15H10O6 | 2.45 | 94.57 | 287.05,153.01 | Luteolin or its isomer | Flavonoids | 31,39 | 491-70-3 |
| 252 | 13.50 | 181.0390 | [M+H]⁺ | C9H8O4 | -1.55 | 91.19 | 181.04.163.03,145.02,135.04,117.03 | Caffeic acid or its isomer | Phenolics | 9,44,47 | 331-39-5 |
| 253 | 13.51 | 453.1749 | [M+H]⁺ | C22H28O10 | 1.59 | 95.81 | 453.17,291.12,273.11 | 4'-O-β-D-glucosyl-5-O-methylvisamminol | Flavonoids | 14,22,86 | 84272-85-5 |
| 254 | 13.53 | 595.1633 | [M+H]⁺ | C27H30O15 | 2.61 | 96.63 | 595.16,449.10,287.05,129.05,85.02 | Kaempferol-3-O-neohesperidoside | Flavonoids | 31 | 32602-81-6 |
| 255 | 13.54 | 163.0382 | [M+H]⁺ | C9H6O3 | 5.67 | 82.72 | 163.03,145.02,117.03 | 7-Hydroxycoumarin or its isomer | Coumarins | 16,65 | 93-35-6 |
| 256 | 13.55 | 179.0693 | [M+H]⁺ | C10H10O3 | 2.10 | 80.83 | 179.06,147.04,119.04,91.05 | Methyl 4-hydroxycinnamate | Phenylpropanoids | 13,95 | 3943-97-3 |
| 257 | 13.55 | 493.1327 | [M+H]⁺ | C23H24O12 | -0.81 | 98.07 | 493.13,331.08 | IristectorinA or its isomer | Isoflavone | 10,81 | 37744-61-9 |
| 258 | 13.57 | 342.1677 | [M+H]⁺ | C20H24NO4 | 5.76 | 74.19 | 342.17,297.11,265.08,237.09 | Magnoflorine or its isomer | Alkaloid | 72 | 2141-09-5 |
| 259 | 13.61 | 465.1041 | [M+H]⁺ | C21H20O12 | -2.85 | 91.11 | 465.10,303.04 | Quercetin-7-O-β-D-glucopyranoside or its isomer | Flavonoids | 54 | 491-50-9 |
| 260 | 13.62 | 322.1069 | [M+H]⁺ | C19H15NO4 | 1.62 | 89.80 | 322.10,307.08,294.07,279.08 | Berberrubine or its isomer | Alkaloid | 2 | 17388-19-1 |
| 261 | 13.63 | 598.2470 | [M+H]⁺ | C28H36O13 | 5.22 | 74.74 | 598.24,401.15,265.10 | Episyringaresinol 4'-O-β-D-glncopyranoside | Lignans | 91 | 137038-13-2 |
| 262 | 13.64 | 118.0860 | [M+H]⁺ | C5H11NO2 | 2.97 | 80.60 | 118.08,59.07 | Betaine or its isomer | Alkaloid | 95 | 107-43-7 |
| 263 | 13.64 | 317.0645 | [M+H]⁺ | C16H12O7 | 3.60 | 91.10 | 317.06,302.04,285.03,257.04,229.04,201.05,177.05,153.01 | Isorhamnetin or its isomer | Flavonoids | 31 | 480-19-3 |
| 264 | 13.66 | 625.1745 | [M+H]⁺ | C28H32O16 | 2.65 | 91.52 | 625.17,317.06,85.02,71.04 | Isorhamnetin-3-O-neohespeidoside or its isomer | Flavonoids | 31 | 55033-90-4 |
| 265 | 13.67 | 368.1121 | [M+H]⁺ | C20H17NO6 | 3.50 | 77.50 | 368.11,327.08 | Bicuculline | Alkaloid | 2 | 485-49-4 |
| 266 | 13.67 | 449.1072 | [M+H]⁺ | C21H20O11 | 1.42 | 99.07 | 449.10,287.05 | Kaempferol 7-O-β-D-glucoside or its isomer | Flavonoids | 31,39 | 16290-07-6 |
| 267 | 13.68 | 574.3010 | [M+H]⁺ | C31H43NO9 | 0.95 | 76.08 | 574.29,540.26,508.22,105.03 | Benzoylhypaconine or its isomer | Alkaloid | 75 | 63238-66-4 |
| 268 | 13.68 | 247.0965 | [M+H]⁺ | C14H14O4 | -2.58 | 91.17 | 247.09,229.08,175.03 | Decursinol or its isomer | Coumarins | 45 | 23458-02-8 |
| 269 | 13.68 | 430.3293 | [M+H]⁺ | C27H43NO3 | 1.33 | 93.08 | 430.32,412.32,138.12 | Sipeimine or its isomer | Alkaloid | 48 | 61825-98-7 |
| 270 | 13.70 | 428.3150 | [M+H]⁺ | C27H41NO3 | 2.52 | 87.56 | 428.31,114.09 | Peimisine | Alkaloid | 32,48 | 19773-24-1 |
| 271 | 13.72 | 352.1178 | [M+H]⁺ | C20H17NO5 | 1.14 | 90.07 | 352.11,337.09,322.07,308.09,294.07 | 8-Oxoberberine or its isomer | Alkaloid | 2,16,25,55,60 | 549-21-3 |
| 272 | 13.73 | 590.2964 | [M+H]⁺ | C31H43NO10 | 2.51 | 85.90 | 590.29,540.25,508.23,105.03 | Benzoylmesaconine or its isomer | Alkaloid | 95 | 63238-67-5 |
| 273 | 13.74 | 225.1122 | [M+H]⁺ | C12H16O4 | 0.34 | 80.09 | 225.11,207.10,133.10,91.05 | Senkyunolide H or its isomer | Esters | 42,84 | 94596-27-7 |
| 274 | 13.79 | 417.1157 | [M+H]⁺ | C21H20O9 | 2.83 | 87.73 | 417.11,399.10,381.09,363.08,351.08,335.09,321.07,307.09,297.07,279.06,267.06 | Puerarin or its isomer | Isoflavone | 47,52 | 3681-99-0 |
| 275 | 13.79 | 287.0549 | [M+H]⁺ | C15H10O6 | 1.64 | 93.62 | 287.05,153.01 | Luteolin or its isomer | Flavonoids | 31,39 | 491-70-3 |
| 276 | 13.80 | 305.0656 | [M+H]⁺ | C15H12O7 | 1.72 | 86.10 | 305.06,287.05,259.06,231.06,195.02,179.03,167.03,153.01,123.04 | Taxifolin or its isomer | Flavonoids | 20 | 480-18-2 |
| 277 | 13.82 | 449.1070 | [M+H]⁺ | C21H20O11 | 3.12 | 85.18 | 449.10,287.05 | Luteolin-7-O-β-D-glucoside or its isomer | Flavonoids | 89 | 5373-11-5 |
| 278 | 13.82 | 579.1703 | [M+H]⁺ | C27H30O14 | 4.19 | 83.44 | 579.17,433.11,271.06,129.05 | Rhoifolin | Flavonoids | 45 | 17306-46-6 |
| 279 | 13.83 | 354.1331 | [M+H]⁺ | C20H19NO5 | 1.13 | 89.90 | 354.13,336.12,323.09,305.08,275.07 | Chelidonine or its isomer | Alkaloid | 32 | 476-32-4 |
| 280 | 13.85 | 581.1857 | [M+H]⁺ | C27H32O14 | 1.48 | 90.48 | 581.18,527.15,435.12,419.13,401.12,383.10,315.08,273.07,129.05,85.02 | Naringin | Flavonoids | 40 | 10236-47-2 |
| 281 | 13.85 | 354.1329 | [M+H]⁺ | C20H19NO5 | 1.72 | 87.12 | 354.13,336.12,323.08,305.08,293.08,275.07,247.07,206.08,188.07,165.05,149.05 | Protopine | Alkaloid | 22,94 | 130-86-9 |
| 282 | 13.86 | 368.1482 | [M+H]⁺ | C21H21NO5 | 2.68 | 88.43 | 368.14,337.10,319.09,298.18,177.05,71.08 | Corynoline or its isomer | Alkaloid | 2,55 | 18797-79-0 |
| 283 | 13.87 | 273.0759 | [M+H]⁺ | C15H12O5 | 0.62 | 82.90 | 273.07,231.06,153.01 | Naringenin chalcone or its isomer | Chalcones | 95 | 73692-50-9 |
| 284 | 13.88 | 303.0490 | [M+H]⁺ | C15H10O7 | 2.66 | 96.69 | 303.04,257.04 | Morin or its isomer | Flavonoids | 31 | 480-16-0 |
| 285 | 13.89 | 449.1059 | [M+H]⁺ | C21H20O11 | 3.82 | 90.57 | 449.10,303.04,129.05,85.02 | Quercitrin | Flavonoids | 59 | 522-12-3 |
| 286 | 13.89 | 338.1389 | [M+H]⁺ | C20H20NO4 | 4.71 | 84.17 | 338.13,192.10 | Palmatrubin | Alkaloid | 41 | 16176-68-4 |
| 287 | 13.90 | 303.0499 | [M+H]⁺ | C15H10O7 | 1.34 | 95.24 | 303.04,285.02,257.04,229.04,201.05,165.01,153.01,137.02,121.02 | Quercetin or its isomer | Flavonoids | 59 | 117-39-5 |
| 288 | 13.95 | 479.1163 | [M+H]⁺ | C22H22O12 | 5.00 | 82.85 | 479.11,317.06 | Isorhamnetin -3-O-b-D-galactoside or its isomer | Flavonoids | 31 | 6743-92-6 |
| 289 | 13.96 | 777.2201 | [M+Na]⁺ | C34H42O19 | 3.61 | 88.25 | 777.22,409.11 | 3,6’-Disinapoyl sucrose | Phenylpropanoids | 65 | 139891-98-8 |
| 290 | 13.98 | 609.1811 | [M+H]⁺ | C28H32O15 | 1.08 | 98.72 | 609.18,301.07,286.04 | Neodiosmin | Flavonoids | 11,25,86,95 | 38665-01-9 |
| 291 | 13.98 | 303.0857 | [M+H]⁺ | C16H14O6 | 2.78 | 92.08 | 303.08,261.07,219.06,201.05,177.05,153.01,117.03,89.03,67.01 | Hesperetin or its isomer | Flavonoids | 34,40,52 | 520-33-2 |
| 292 | 13.99 | 433.1125 | [M+H]⁺ | C21H20O10 | 1.72 | 91.14 | 433.11,271.06 | Apigenin-7-glucoside or its isomer | Flavonoids | 31,67,80 | 578-74-5 |
| 293 | 14.03 | 181.0386 | [M+H]⁺ | C9H8O4 | 0.81 | 94.37 | 181.04.163.03,145.02,135.04,117.03 | Caffeic acid or its isomer | Phenolics | 7,10,44 | 331-39-5 |
| 294 | 14.06 | 447.0915 | [M+H]⁺ | C21H18O11 | 2.72 | 97.96 | 447.09,271.05 | Apigenin 7-O-beta-D-glucuronide | Flavonoids | 54,55 | 29741-09-1 |
| 295 | 14.06 | 432.3463 | [M+H]⁺ | C27H45NO3 | 1.13 | 91.01 | 432.34,109.03 | Peimine or its isomer | Alkaloid | 32,63 | 23496-41-5 |
| 296 | 14.06 | 785.2281 | [M+H]⁺ | C38H40O18 | 3.12 | 80.82 | 785.23,429.11 | 6'''-Feruloylspinosin | Flavonoids | 25 | 77690-92-7 |
| 297 | 14.07 | 301.0694 | [M+H]⁺ | C16H12O6 | -4.16 | 87.74 | 301.07,286.04 | Tectorigenin or its isomer | Isoflavone | 77 | 548-77-6 |
| 298 | 14.18 | 481.1702 | [M+H]⁺ | C23H28O11 | -2.90 | 91.35 | 481.16,319.11,301.10,267.08,197.08,179.07,151.07,133.06,121.06,105.03,85.02,69.03 | Albiflorin or its isomer | Terpenoids | 16,25,40,69,70,86 | 39011-90-0 |
| 299 | 14.18 | 287.0549 | [M+H]⁺ | C21H20O11 | -1.91 | 93.10 | 449.10,287.05 | Kaempferol 7-O-β-D-glucoside or its isomer | Flavonoids | 39 | 16290-07-6 |
| 300 | 14.19 | 287.0543 | [M+H]⁺ | C15H10O6 | 2.43 | 94.93 | 287.05,258.04,165.01,153.01,68.99 | Kaempferol or its isomer | Flavonoids | 31 | 520-18-3 |
| 301 | 14.22 | 163.0384 | [M+H]⁺ | C9H6O3 | 4.73 | 90.09 | 163.03,145.02,117.03 | 7-Hydroxycoumarin or its isomer | Coumarins | 15,25,26,32,54,89,95 | 93-35-6 |
| 302 | 14.22 | 517.1330 | [M+H]⁺ | C25H24O12 | 0.75 | 95.79 | 517.13,449.12,355.11,319.08,163.03,145.02 | 1,3-O-Dicaffeoylquinic acid | Phenolics | 89 | 19870-46-3 |
| 303 | 14.23 | 223.0600 | [M+H]⁺ | C11H10O5 | 0.20 | 91.69 | 223.06,208.02,190.02,179.03,163.04,149.02,135.04,107.04,89.05,78.04,59.04 | Isofraxidin or its isomer | Coumarins | 39 | 486-21-5 |
| 304 | 14.25 | 305.1019 | [M+H]⁺ | C16H16O6 | -1.33 | 78.65 | 305.10,203.03,147.04,85.06,59.04 | Oxypeucedanin hydrate or its isomer | Coumarins | 5 | 2643-85-8 |
| 305 | 14.27 | 517.1325 | [M+H]⁺ | C25H24O12 | 2.20 | 96.84 | 517.13,163.03 | 1,4-Dicaffeoylquinic acid | Phenolics | 7 | 1182-34-9 |
| 306 | 14.31 | 247.0959 | [M+H]⁺ | C14H14O4 | 9.38 | 89.11 | 247.09,229.08,175.03 | Columbianetin or its isomer | Coumarins | 13,87 | 3804-70-4 |
| 307 | 14.33 | 225.1101 | [M+H]⁺ | C12H16O4 | -5.72 | 83.65 | 225.11,207.10,133.10,91.05 | Senkyunolide H or its isomer | Esters | 84 | 94596-27-7 |
| 308 | 14.33 | 368.1472 | [M+H]⁺ | C21H21NO5 | 3.92 | 87.31 | 368.14,337.10,319.09,298.18,177.05,71.08 | Corynoline or its isomer | Alkaloid | 16,22,30 | 18797-79-0 |
| 309 | 14.33 | 247.0600 | [M+H]⁺ | C13H10O5 | 0.58 | 81.29 | 247.05,232.03,217.01,189.01,173.13,161.02 | Isopimpinellin or its isomer | Coumarins | 7 | 482-27-9 |
| 310 | 14.35 | 287.0533 | [M+H]⁺ | C15H10O6 | 7.63 | 82.55 | 287.05,153.01 | Luteolin or its isomer | Flavonoids | 2 | 491-70-3 |
| 311 | 14.36 | 663.2267 | [M+H]⁺ | C32H38O15 | 2.05 | 96.48 | 663.22,517.17,355.11,299.05,129.05,85.02,71.04 | Epimedoside A or its isomer | Flavonoids | 89 | 39012-04-9 |
| 312 | 14.38 | 303.0857 | [M+H]⁺ | C16H14O6 | 1.41 | 90.31 | 303.08,261.07,219.06,201.05,177.05,153.01,117.03,89.03,67.01 | Hesperetin or its isomer | Flavonoids | 40 | 520-33-2 |
| 313 | 14.39 | 284.1299 | [M+H]⁺ | C17H17NO3 | -4.49 | 85.12 | 284.12,147.04,121.06 | Paprazine or it isomer | Phenylpropanoids | 30,75 | 36417-86-4 |
| 314 | 14.41 | 203.0333 | [M+H]⁺ | C11H6O4 | -2.20 | 83.74 | 203.03,175.03,159.04,147.04,131.05,91.05 | Xanthotoxol or its isomer | Coumarins | 52,87 | 2009-24-7 |
| 315 | 14.42 | 181.0492 | [M+H]⁺ | C9H8O4 | 1.76 | 84.74 | 181.04.163.03,145.02,135.04,117.03 | Caffeic acid or its isomer | Phenolics | 32 | 331-39-5 |
| 316 | 14.44 | 356.1857 | [M+H]⁺ | C21H25NO4 | 1.28 | 89.69 | 356.18,192.10 | Tetrahydropalmatine or its isomer | Alkaloid | 16,94 | 2934-97-6 |
| 317 | 14.44 | 319.0447 | [M+H]⁺ | C15H10O8 | 0.73 | 94.78 | 319.04,301.03,273.03,245.04,217.04,179.03,165.01,153.01,137.02 | Myricetin or its isomer | Flavonoids | 39,67 | 529-44-2 |
| 318 | 14.44 | 271.0593 | [M+H]⁺ | C15H10O5 | -3.84 | 82.60 | 271.05,215.06 | 4',6,7-Trihydroxyisoflavone | Isoflavone | 57 | 17817-31-1 |
| 319 | 14.44 | 203.0333 | [M+H]⁺ | C11H6O4 | -2.37 | 84.99 | 203.03,175.03,159.04,147.04,131.04,119.04,103.05,91.05,77.03,65.03 | Bergaptol or its isomer | Coumarins | 5 | 486-60-2 |
| 320 | 14.50 | 301.0708 | [M+H]⁺ | C16H12O6 | 1.62 | 90.03 | 301.07,286.04 | Tectorigenin or its isomer | Flavonoids | 57 | 548-77-6 |
| 321 | 14.50 | 449.1064 | [M+H]⁺ | C21H20O11 | 1.82 | 97.78 | 449.10,287.05 | Luteolin-7-O-β-D-glucoside or its isomer | Flavonoids | 31 | 5373-11-5 |
| 322 | 14.50 | 430.3311 | [M+H]⁺ | C27H43NO3 | 0.54 | 97.40 | 430.33,412.16 | Peiminine or its isomer | Alkaloid | 32 | 18059-10-4 |
| 323 | 14.51 | 338.1393 | [M+H]⁺ | C20H19NO4 | -0.26 | 88.90 | 338.13,322.10,308.09,294.11,280.09,262.08,250.08,222.09 | Jatrorrhizine or its isomer | Alkaloid | 2,16,22,60,72,94 | 3621-38-3 |
| 324 | 14.54 | 359.1500 | [M+H]⁺ | C20H22O6 | -3.71 | 83.02 | 359.14,323.12,291.10,263.10,231.08,205.08,189.09,163.07,137.05 | Matairesinol or its isomer | Lignans | 9 | 580-72-3 |
| 325 | 14.58 | 263.0902 | [M+H]⁺ | C14H14O5 | -3.22 | 90.85 | 263.09,203.07 | (+)-Ciskhellactone | Esters | 44 | 24144-61-4 |
| 326 | 14.59 | 336.1228 | [M+H]⁺ | C20H18NO4 | 0.25 | 99.06 | 336.12,320.09 | Epiberberine or its isomer | Alkaloid | 60,72 | 6873-09-2 |
| 327 | 14.61 | 579.1694 | [M+H]⁺ | C27H30O14 | 1.22 | 96.23 | 579.17,417.11,255.06,85.02 | Chrysin 7-O-β-gentiobioside | Flavonoids | 98 | 88640-89-5 |
| 328 | 14.61 | 324.1224 | [M+H]⁺ | C19H17NO4 | 3.27 | 90.19 | 324.12,176.06 | Stylopine | Alkaloid | 94 | 84-39-9 |
| 329 | 14.62 | 303.0499 | [M+H]⁺ | C15H10O7 | -0.05 | 88.86 | 303.04,285.02,257.04,229.04,201.05,165.01,153.01,137.02 | Quercetin or its isomer | Flavonoids | 31,67 | 117-39-5 |
| 330 | 14.63 | 271.0588 | [M+H]⁺ | C15H10O5 | -3.41 | 82.13 | 271.06,215.06,153.01,119.04 | Apigenin or its isomer | Flavonoids | 3 | 520-36-5 |
| 331 | 14.63 | 465.1022 | [M+H]⁺ | C21H20O12 | -0.26 | 98.37 | 465.10,303.05 | Isoquercitrin or its isomer | Flavonoids | 67 | 21637-25-2 |
| 332 | 14.64 | 590.2957 | [M+H]⁺ | C31H43NO10 | -0.02 | 95.06 | 590.29,540.25,508.23,105.03 | Benzoylmesaconine or its isomer | Alkaloid | 86,87 | 63238-67-5 |
| 333 | 14.66 | 118.0860 | [M+H]⁺ | C5H11NO2 | 4.18 | 94.35 | 118.08,59.07 | Betaine or its isomer | Alkaloid | 87 | 107-43-7 |
| 334 | 14.67 | 322.1068 | [M+H]⁺ | C19H16NO4 | -2.91 | 87.37 | 322.10,307.08 | Groenlandicine | Alkaloid | 72 | 38691-95-1 |
| 335 | 14.67 | 574.3015 | [M+H]⁺ | C31H43NO9 | -3.24 | 83.04 | 574.29,540.26,508.22,105.03 | Benzoylhypaconine or its isomer | Alkaloid | 86 | 63238-66-4 |
| 336 | 14.69 | 809.2866 | [M+H]⁺ | C38H48O19 | 0.94 | 92.00 | 809.28,664.22,517.16,355.11,300.05,179.07 | Baohuoside V | Flavonoids | 89 | 118544-18-6 |
| 337 | 14.69 | 257.0798 | [M+H]⁺ | C15H12O4 | -2.37 | 80.73 | 257.08,239.06,211.07,163.03,147.04,137.02 | Isoliquiritigenin or its isomer | Chalcones | 5,77 | 961-29-5 |
| 338 | 14.71 | 419.1338 | [M+H]⁺ | C21H22O9 | 1.71 | 90.03 | 419.13,257.08,137.02 | Liquiritin | Chalcones | 5,11 | 551-15-5 |
| 339 | 14.71 | 551.1775 | [M+H]⁺ | C26H30O13 | 2.01 | 90.16 | 551.17,419.13,257.08 | Isoliquiritin apioside | Chalcones | 28 | 120926-46-7 |
| 340 | 14.71 | 419.1333 | [M+H]⁺ | C21H22O9 | -0.28 | 91.95 | 419.03,257.08 | Pinocembrin 7-O-bate-D-glucoside or its isomer | Flavonoids | 92 | 75829-43-5 |
| 341 | 14.72 | 314.1372 | [M+H]⁺ | C18H19NO4 | 3.41 | 89.23 | 314.13,177.05,145.02,121.06 | N-trans-Feruloyltyramine or its isomer | Alkaloid | 22,29,30,59,63,82,88 | 66648-43-9 |
| 342 | 14.72 | 663.2267 | [M+H]⁺ | C32H38O15 | 1.64 | 96.46 | 663.22,517.17,355.11,299.05,129.05,85.02,71.04 | Epimedoside A or its isomer | Flavonoids | 12,89 | 39012-04-9 |
| 343 | 14.73 | 320.0924 | [M+H]⁺ | C19H14ClNO4 | -0.51 | 97.78 | 320.09,307.08,292.09,277.07 | Coptisine chloride or its iosmer | Alkaloid | 60 | 6020-18-4 |
| 344 | 14.74 | 322.1069 | [M+H]⁺ | C19H15NO4 | -1.23 | 93.08 | 322.10,307.08,292.05,279.08,264.06,250.08,221.08 | Berberrubine or its isomer | Alkaloid | 16 | 17388-19-1 |
| 345 | 14.77 | 338.1402 | [M+H]⁺ | C20H20NO4 | 2.01 | 93.80 | 338.13,322.10,308.09,294.11,280.09,262.08,250.08,222.09 | Jatrorrhizine or its isomer | Alkaloid | 25,55,60,72,95 | 3621-38-3 |
| 346 | 14.78 | 225.1011 | [M+H]⁺ | C12H16O4 | 4.27 | 84.81 | 225.11,207.10,133.10,91.05 | Senkyunolide H or its isomer | Esters | 23,42 | 94596-27-7 |
| 347 | 14.78 | 574.3009 | [M+H]⁺ | C31H43NO9 | -0.47 | 89.13 | 574.29,540.26,508.22,105.03 | Benzoylhypaconine or its isomer | Alkaloid | 95 | 63238-66-4 |
| 348 | 14.79 | 465.1022 | [M+H]⁺ | C21H20O12 | -1.44 | 82.87 | 465.10,303.04 | Quercetin-7-O-β-D-glucopyranoside or its isomer | Flavonoids | 67 | 491-50-9 |
| 349 | 14.84 | 331.0807 | [M+H]⁺ | C17H14O7 | 0.34 | 99.12 | 331.08,298.04 | Jaceosidin or its isomer | Flavonoids | 41 | 18085-97-7 |
| 350 | 14.84 | 320.0923 | [M+H]⁺ | C19H14NO4 | -1.92 | 95.52 | 320.09,292.09 | Coptisine or its isomer | Alkaloid | 41,72 | 3486-66-6 |
| 351 | 14.86 | 431.1330 | [M+H]⁺ | C22H22O9 | -0.45 | 93.88 | 431.13,269.08 | Formononetin glucoside | Flavonoids | 9,11,12,16,22,23,36,47,55,56,77,86,92,95 | 486-62-4 |
| 352 | 14.91 | 493.1320 | [M+H]⁺ | C23H24O12 | -2.95 | 93.71 | 493.13,331.08 | IristectorinA or its isomer | Isoflavone | 41 | 37744-61-9 |
| 353 | 14.91 | 269.0799 | [M+H]⁺ | C16H12O4 | -1.71 | 83.03 | 269.08,254.05,237.05,213.09,197.05,136.01,118.04 | Formononetin or its isomer | Flavonoids | 19,77,91 | 485-72-3 |
| 354 | 14.93 | 447.0980 | [M+H]⁺ | C21H18O11 | 0.19 | 98.60 | 447.09,271.06,169.01,85.02 | Glychionide or its isomer | Flavonoids | 1,2,5,48,52,81,94,98 | 119152-50-0 |
| 355 | 14.93 | 189.0543 | [M+H]⁺ | C11H8O3 | -0.29 | 84.21 | 189.05,161.06,133.06 | Plumbagin | Quinones | 40,44,45 | 481-42-5 |
| 356 | 14.99 | 419.1331 | [M+H]⁺ | C21H22O9 | 0.11 | 91.46 | 419.03,257.08 | Pinocembrin 7-O-bate-D-glucoside or its isomer | Flavonoids | 5,28,36,40,69,86 | 75829-43-5 |
| 357 | 15.00 | 271.0594 | [M+H]⁺ | C15H10O5 | 2.33 | 93.56 | 271.05,253.04,225.05,169.01,123.00,103.05 | Baicalein or its isomer | Flavonoids | 2,3 | 491-67-8 |
| 358 | 15.01 | 463.0861 | [M+H]⁺ | C21H18O12 | -0.48 | 97.22 | 463.08,287.20 | Luteolin-7-O-β-D-glucuronide or its isomer | Flavonoids | 55 | 29741-10-4 |
| 359 | 15.02 | 433.1121 | [M+H]⁺ | C21H20O10 | 1.11 | 98.73 | 433.11,415.10,271.06,253.04,123.00 | Oroxin A or its isomer | Flavonoids | 98 | 57396-78-8 |
| 360 | 15.03 | 340.1543 | [M+H]⁺ | C20H21NO4 | 0.86 | 86.80 | 340.15,176.07,149.05 | Tetrahydroberberine | Alkaloid | 94 | 5096-57-1 |
| 361 | 15.11 | 419.1336 | [M+H]⁺ | C21H22O9 | -1.82 | 88.75 | 419.13,257.08,137.02 | Isoliquiritin or its isomer | Chalcones | 95 | 5041-81-6 |
| 362 | 15.11 | 284.1271 | [M+H]⁺ | C17H17NO3 | 0.78 | 96.51 | 284.12,147.04,121.06 | Paprazine or it isomer | Phenylpropanoids | 13,16,29,30 | 36417-86-4 |
| 363 | 15.12 | 370.2004 | [M+H]⁺ | C22H27NO4 | 1.94 | 92.54 | 370.20,192.10 | (+)- Corydaline | Alkaloid | 94 | 518-69-4 |
| 364 | 15.15 | 247.0965 | [M+H]⁺ | C14H14O4 | -0.41 | 96.36 | 247.09,229.08,175.03 | Columbianetin or its isomer | Coumarins | 5,29,42,48,54 | 3804-70-4 |
| 365 | 15.16 | 255.0644 | [M+H]⁺ | C15H10O4 | -2.41 | 82.70 | 255.06,237.05 | Chrysin or its isomer | Flavonoids | 28,40 | 480-40-0 |
| 366 | 15.16 | 277.1063 | [M+H]⁺ | C15H16O5 | -2.91 | 89.48 | 277.10,205.04 | Hamaudol or its isomer | Flavonoids | 29 | 735-46-6 |
| 367 | 15.17 | 447.0910 | [M+H]⁺ | C21H18O11 | 1.28 | 94.61 | 447.09,271.06,169.01,85.02 | Glychionide or its isomer | Flavonoids | 1,2,3,4,5,11,47,51,52,63,81,92,100 | 119152-50-0 |
| 368 | 15.19 | 247.0959 | [M+H]⁺ | C14H14O4 | -2.56 | 95.86 | 247.09,229.08,175.03 | Decursinol or its isomer | Coumarins | 86,87,95 | 23458-02-8 |
| 369 | 15.20 | 271.0594 | [M+H]⁺ | C15H10O5 | -1.00 | 83.66 | 271.06,215.06,153.01,119.04 | Apigenin or its isomer | Flavonoids | 7 | 520-36-5 |
| 370 | 15.21 | 352.1540 | [M+H]⁺ | C21H21NO4 | 1.99 | 87.38 | 352.15,336.12,322.10,308.12 | Palmatine or its isomer | Alkaloid | 41,94 | 3486-67-7 |
| 371 | 15.21 | 368.1493 | [M+H]⁺ | C21H21NO5 | 1.96 | 83.41 | 368.14,337.10,319.09,298.18,177.05,71.08 | Corynoline or its isomer | Alkaloid | 2 | 18797-79-0 |
| 372 | 15.22 | 338.1388 | [M+H]⁺ | C20H19NO4 | 2.28 | 88.12 | 338.13,322.10,308.09,294.11,280.09,262.08,250.08,222.09 | Jatrorrhizine or its isomer | Alkaloid | 55 | 3621-38-3 |
| 373 | 15.24 | 604.3117 | [M+H]⁺ | C32H45NO10 | 0.18 | 92.12 | 604.31,554.27,522.24,105.03,58.06 | Benzoylaconitine | Alkaloid | 86,87,95 | 466-24-0 |
| 374 | 15.26 | 419.1347 | [M+H]⁺ | C21H22O9 | -5.20 | 86.35 | 419.03,257.08 | Pinocembrin 7-O-bate-D-glucoside or its isomer | Flavonoids | 28,77,86 | 75829-43-5 |
| 375 | 15.26 | 223.0610 | [M+H]⁺ | C11H10O5 | 3.92 | 77.76 | 223.06,208.02,190.02,179.03,163.04,149.02,135.04,107.04,89.05,78.04,59.04 | Isofraxidin or its isomer | Coumarins | 9 | 486-21-5 |
| 376 | 15.26 | 247.0976 | [M+H]⁺ | C14H14O4 | -1.87 | 84.91 | 247.09,229.08,175.04, | Nodakenitin | Coumarins | 90 | 495-32-9 |
| 377 | 15.30 | 416.3521 | [M+H]⁺ | C27H45NO2 | 1.20 | 90.19 | 416.35,398.34,356.13,273.22 | Tomatidine | Alkaloid | 32 | 77-59-8 |
| 378 | 15.31 | 322.1067 | [M+H]⁺ | C19H15NO4 | 1.19 | 92.18 | 322.10,307.08,292.05,279.08,264.06,250.08,221.08 | Berberrubine or its isomer | Alkaloid | 2,25,95 | 17388-19-1 |
| 379 | 15.32 | 373.1646 | [M+H]⁺ | C21H24O6 | 4.66 | 74.57 | 374.16,147.07,137.05,122.03,94.04 | Tetrahydrocurcumin | Phenolics | 54 | 36062-04-1 |
| 380 | 15.33 | 922.4942 | [M+H]⁺ | C45H76O19 | -4.01 | 87.27 | 903.49,741.43,417.33,273.22,255.20 | Officinalisinin I | Saponin | 14,89 | 57944-18-0 |
| 381 | 15.40 | 463.1231 | [M+H]⁺ | C22H22O11 | 0.09 | 95.09 | 463.12,301.06 | Diosmetin-7-O-β-D-glucopyranoside or its isomer | Flavonoids | 98 | 20126-59-4 |
| 382 | 15.41 | 314.1378 | [M+H]⁺ | C18H19NO4 | 5.27 | 87.94 | 314.13,177.05,145.02,121.06 | N-trans-Feruloyltyramine or its isomer | Alkaloid | 2,13,16,30,40,59,65,67 | 66648-43-9 |
| 383 | 15.41 | 287.0923 | [M+H]⁺ | C16H14O5 | 1.13 | 76.73 | 287.09,273.14,245.08,193.05,167.03,151.04,121.01 | Licochalcone B | Chalcones | 5,56 | 58749-23-8 |
| 384 | 15.41 | 473.1404 | [M+Na]⁺ | C22H26O10 | -2.43 | 87.00 | 473.14,311.08 | Asebotin | Phenolics | 42 | 11075-15-3 |
| 385 | 15.42 | 247.0962 | [M+H]⁺ | C14H14O4 | -0.74 | 95.16 | 247.09,229.08,175.03 | Columbianetin or its isomer | Coumarins | 42 | 3804-70-4 |
| 386 | 15.43 | 255.0639 | [M+H]⁺ | C15H10O4 | -5.08 | 86.86 | 255.06,227.07,209.05,181.06,165.06,153.06,135.04,121.02 | Chrysophanol or its isomer | Anthraquinone | 22,45 | 481-74-3 |
| 387 | 15.45 | 255.0644 | [M+H]⁺ | C15H10O4 | 2.13 | 78.18 | 255.06,237.05,227.07,199.07,137.02 | Daidzein or its isomer | Isoflavone | 11,55,89 | 486-66-8 |
| 388 | 15.46 | 368.1484 | [M+H]⁺ | C21H21NO5 | 1.58 | 90.18 | 368.14,337.10,319.09,298.18,177.05,71.08 | Corynoline or its isomer | Alkaloid | 22 | 18797-79-0 |
| 389 | 15.47 | 336.1228 | [M+H]⁺ | C20H18NO4 | 1.01 | 91.40 | 336.12,321.09,292.09,275.09 | Berberine or its isomer | Alkaloid | 60 | 2086-83-1 |
| 390 | 15.47 | 447.0921 | [M+H]⁺ | C21H18O11 | 0.51 | 98.31 | 447.09,271.06,169.01,85.02 | Glychionide or its isomer | Flavonoids | 1,2,3,5,7,10,11,47,48,52,81,92,100 | 119152-50-0 |
| 391 | 15.48 | 273.0753 | [M+H]⁺ | C15H12O5 | -1.50 | 85.43 | 273.07,231.06,153.01 | Naringenin chalcone or its isomer | Chalcones | 3,11 | 73692-50-9 |
| 392 | 15.51 | 247.0964 | [M+H]⁺ | C14H14O4 | -0.32 | 96.59 | 247.09,229.08,175.03 | Decursinol or its isomer | Coumarins | 95 | 23458-02-8 |
| 393 | 15.51 | 352.1183 | [M+H]⁺ | C20H17NO5 | -3.61 | 90.53 | 352.11,337.08,322.07,308.09 | 8-Oxo-epiberberine | Alkaloid | 60 | 19716-60-0 |
| 394 | 15.51 | 313.1805 | [M+H]⁺ | C20H24O3 | -1.67 | 92.53 | 313.17,253.12,227.10 | Triptophenolide | Terpenoids | 78 | 74285-86-2 |
| 395 | 15.51 | 285.0746 | [M+H]⁺ | C16H12O5 | -3.74 | 98.13 | 285.07,270.05 | 7-O-Methylbaicalein or its isomer | Flavonoids | 11,51,52,53,63,81 | 29550-13-8 |
| 396 | 15.53 | 352.1171 | [M+H]⁺ | C20H17NO5 | 2.66 | 94.68 | 352.11,337.09,322.07,308.09,294.07 | 8-Oxoberberine or its isomer | Alkaloid | 2,16 | 549-21-3 |
| 397 | 15.55 | 285.0752 | [M+H]⁺ | C16H12O5 | -1.24 | 90.61 | 285.07,270.05 | Wogonin or its isomer | Flavonoids | 1 | 632-85-9 |
| 398 | 15.57 | 461.1401 | [M+H]⁺ | C23H24O10 | -0.71 | 97.50 | 461.14,299.09 | 8-Methylretusin-7-O-glucopyranoside | Flavonoids | 91 | 68862-13-5 |
| 399 | 15.59 | 233.1535 | [M+H]⁺ | C15H20O2 | -0.08 | 84.21 | 233.15,177.08 | Pterosin Z or its isomer | Terpenoids | 20 | 34169-69-2 |
| 400 | 15.60 | 334.1077 | [M+H]⁺ | C20H15NO4 | 2.52 | 75.82 | 334.10,319.08 | Dihydrosanguinarine | Alkaloid | 72 | 3606-45-9 |
| 401 | 15.60 | 257.0812 | [M+H]⁺ | C15H12O4 | 0.45 | 96.31 | 257.08,239.06,211.07,163.03,147.04,137.02 | Isoliquiritigenin or its isomer | Chalcones | 5,9,28,40,47,56,63,69,77,86 | 961-29-5 |
| 402 | 15.61 | 315.0858 | [M+H]⁺ | C17H14O6 | -2.19 | 88.97 | 315.08,300.06 | Kumatakenin or its isomer | Flavonoids | 10 | 3301-49-3 |
| 403 | 15.61 | 233.1528 | [M+H]⁺ | C15H20O2 | 2.25 | 83.11 | 233.15,167.08,157.07,145.07,131.08,117.06,105.06,91.05 | Costunolide or its isomer | Terpenoids | 97 | 553-21-9 |
| 404 | 15.64 | 271.0598 | [M+H]⁺ | C15H10O5 | 1.38 | 79.86 | 271.05,253.04,225.05,169.01,123.00,103.05 | Baicalein or its isomer | Flavonoids | 81 | 491-67-8 |
| 405 | 15.66 | 271.0600 | [M+H]⁺ | C15H10O5 | -0.97 | 85.13 | 271.06,169.01,105.03,69.00 | Galangin | Flavonoids | 10 | 548-83-4 |
| 406 | 15.66 | 257.0803 | [M+H]⁺ | C15H12O4 | 4.68 | 80.95 | 257.07,137.02 | Pinocembrin or its isomer | Flavonoids | 95 | 480-39-7 |
| 407 | 15.69 | 203.0337 | [M+H]⁺ | C11H6O4 | 3.51 | 94.44 | 203.03,185.02,175.03,159.04,147.04,131.05,119.04,103.05,91.05,77.03 | Xanthotoxol or its isomer | Coumarins | 5,40,42,86,87,95 | 2009-24-7 |
| 408 | 15.69 | 432.3463 | [M+H]⁺ | C27H45NO3 | 0.32 | 91.67 | 432.34,109.03 | Peimine or its isomer | Alkaloid | 32 | 23496-41-5 |
| 409 | 15.71 | 373.1646 | [M+H]⁺ | C21H24O6 | -0.62 | 95.40 | 373.16,355.15,323.12,305.11,237.10,177.09,151.0,137.05 | Arctigenin or its isomer | Lignans | 54 | 7770-78-7 |
| 410 | 15.72 | 574.3014 | [M+H]⁺ | C31H43NO9 | -0.48 | 91.05 | 574.29,540.26,508.22,105.03 | Benzoylhypaconine or its isomer | Alkaloid | 86,87,95 | 63238-66-4 |
| 411 | 15.75 | 336.1254 | [M+H]⁺ | C20H18NO4 | 1.81 | 94.54 | 336.12,321.09,292.09,275.09 | Berberine or its isomer | Alkaloid | 3,20,42,45 | 2086-83-1 |
| 412 | 15.75 | 352.1540 | [M+H]⁺ | C21H21NO4 | 1.22 | 86.10 | 352.15,336.12,322.10,308.12 | Palmatine | Alkaloid | 94 | 10605-02-4 |
| 413 | 15.76 | 305.1026 | [M+H]⁺ | C16H16O6 | -1.21 | 92.49 | 305.10,203.03,147.04,85.06,59.04 | Oxypeucedanin hydrate or its isomer | Coumarins | 73,86,87,95 | 2643-85-8 |
| 414 | 15.76 | 839.2977 | [M+H]⁺ | C39H50O20 | 0.94 | 95.98 | 839.29,678.23,531.19,369.12 | Epimedin A1 | Flavonoids | 12,89 | 140147-77-9 |
| 415 | 15.76 | 285.0747 | [M+H]⁺ | C16H12O5 | -3.74 | 98.88 | 285.07,270.05 | 7-O-Methylbaicalein or its isomer | Flavonoids | 10,48 | 29550-13-8 |
| 416 | 15.76 | 285.0756 | [M+H]⁺ | C16H12O5 | -0.08 | 81.82 | 285.07,123.11 | Calycosin | Flavonoids | 45 | 20575-57-9 |
| 417 | 15.76 | 301.0697 | [M+H]⁺ | C16H12O6 | -6.10 | 74.58 | 301.07,286.04,168.00 | Hispidulin or its isomer | Flavonoids | 92 | 1447-88-7 |
| 418 | 15.79 | 531.1854 | [M+H]⁺ | C27H30O11 | -0.57 | 82.74 | 531.18,369.13,313.07,243.06,187.07,135.04 | Anhydroicaritin-7-O-glucoside or its isomer | Flavonoids | 12,89 | 56725-99-6 |
| 419 | 15.80 | 593.1857 | [M+H]⁺ | C28H32O14 | 0.39 | 91.87 | 593.18,285.07,85.02 | Buddleoside | Flavonoids | 15 | 480-36-4 |
| 420 | 15.80 | 177.0557 | [M+H]⁺ | C10H8O3 | -5.13 | 83.44 | 177.05,161.09,133.06,121.06,77.03 | 7-Methoxycoumarin | Coumarins | 13 | 531-59-9 |
| 421 | 15.84 | 352.1543 | [M+H]⁺ | C21H22NO4 | -1.91 | 88.35 | 352.15,336.12,322.10,308.12,294.11 | Palmatine or its isomer | Alkaloid | 25,41,55,60,95 | 3486-67-7 |
| 422 | 15.86 | 463.1231 | [M+H]⁺ | C22H22O11 | 1.01 | 96.48 | 463.12,301.06 | Diosmetin-7-O-β-D-glucopyranoside or its isomer | Flavonoids | 63 | 20126-59-4 |
| 423 | 15.87 | 417.1170 | [M+H]⁺ | C21H20O9 | 3.75 | 90.03 | 417.11,255.06 | Daidzin/Daidzoside or its isomer | Isoflavone | 98 | 552-66-9 |
| 424 | 15.88 | 303.1227 | [M+H]⁺ | C17H18O5 | -1.12 | 86.97 | 303.12,167.07,76.07 | Isomucronulatol or its isomer | Flavonoids | 76 | 52250-35-8 |
| 425 | 15.93 | 677.2431 | [M+H]⁺ | C33H40O15 | 1.38 | 86.97 | 677.24,531.18,370.12,314.08,129.05,71.04 | Icariin or its isomer | Flavonoids | 12,89 | 489-32-7 |
| 426 | 15.93 | 447.0903 | [M+H]⁺ | C21H18O11 | -2.97 | 97.67 | 447.09,271.06,169.01,85.02 | Glychionide or its isomer | Flavonoids | 1,4,10,46,47,49,50,48,81 | 119152-50-0 |
| 427 | 15.93 | 577.3726 | [M+H]⁺ | C33H52O8 | 1.64 | 91.14 | 577.37,433.25,253.19 | Diosgenin glucosid or its isomer | Saponin | 75 | 14144-06-0 |
| 428 | 15.97 | 579.1700 | [M+H]⁺ | C27H30O14 | -1.03 | 93.00 | 579.17,285.07 | 6''-O-xylosyl-glycitin | Flavonoids | 91 | 231288-18-9 |
| 429 | 15.98 | 336.1231 | [M+H]⁺ | C20H18NO4 | -4.09 | 83.66 | 336.12,320.09 | Epiberberine or its isomer | Alkaloid | 76 | 6873-09-2 |
| 430 | 15.98 | 461.1078 | [M+H]⁺ | C22H20O11 | 1.40 | 93.83 | 461.10,285.07 | Wogonin 7-O-glucuronide or its isomer | Flavonoids | 2,7,10,94 | 51059-44-0 |
| 431 | 16.02 | 285.0759 | [M+H]⁺ | C16H12O5 | 0.48 | 93.91 | 285.07,270.05,242.05 | Genkwanin | Flavonoids | 77 | 437-64-9 |
| 432 | 16.04 | 317.1019 | [M+H]⁺ | C17H16O6 | -0.90 | 95.04 | 317.10.299.09,273.07,257.08,231.02,215.03,203.03,175.03,85.06,67.05 | Byakangelicol or its isomer | Coumarins | 4,73,86,87 | 26091-79-2 |
| 433 | 16.04 | 303.0496 | [M+H]⁺ | C15H10O7 | 1.03 | 96.32 | 303.04,285.02,257.04,229.04,201.05,165.01,153.01,137.02,121.02 | Quercetin or its isomer | Flavonoids | 39,67,96 | 117-39-5 |
| 434 | 16.05 | 287.0907 | [M+H]⁺ | C16H14O5 | 1.97 | 90.63 | 287.09,219.06,153.01,135.08,121.06 | Isosakuranetin or its isomer | Flavonoids | 40 | 480-43-3 |
| 435 | 16.08 | 373.1646 | [M+H]⁺ | C21H24O6 | 0.37 | 82.05 | 373.16,355.15,323.12,305.11,237.10,177.09,151.0,137.05 | Arctigenin or its isomer | Lignans | 54 | 7770-78-7 |
| 436 | 16.08 | 167.0698 | [M+H]⁺ | C9H10O3 | 2.36 | 91.25 | 167.07,139.03,121.02,95.04 | Ethyl 4-hydroxybenzoate | Esters | 12,62,88,90 | 120-47-8 |
| 437 | 16.10 | 139.0387 | [M+H]⁺ | C7H6O3 | 1.96 | 91.88 | 139.03,121.02,95.04,77.03 | 4-Hydroxybenzoic acid | Organic acid | 62,79,88,90,100 | 99-67-7 |
| 438 | 16.14 | 139.0388 | [M+H]⁺ | C7H6O3 | 1.37 | 94.46 | 139.03,121.02,93.03,65.03 | Salicylic acid | Phenolics | 12 | 69-72-7 |
| 439 | 16.14 | 287.0543 | [M+H]⁺ | C15H10O6 | 3.54 | 91.41 | 287.05,153.01 | Luteolin or its isomer | Flavonoids | 31 | 491-70-3 |
| 440 | 16.15 | 531.1855 | [M+H]⁺ | C27H30O11 | 0.96 | 99.12 | 531.18,369.13,313.07,243.06,187.07,135.04 | Anhydroicaritin-7-O-glucoside or its isomer | Flavonoids | 89 | 56725-99-6 |
| 441 | 16.15 | 439.0997 | [M+H]⁺ | C21H20O9 | 1.05 | 93.84 | 439.09,277.04 | Chrysophanol-1-O-b-D-glucoside | Anthraquinone | 63,92 | 4839-60-5 |
| 442 | 16.15 | 364.1903 | [M+H]⁺ | C23H25NO3 | 2.09 | 92.59 | 364.19,295.13,70.06 | 7-Demethoxytylophorine | Alkaloid | 65 | 32671-82-2 |
| 443 | 16.16 | 255.0654 | [M+H]⁺ | C15H10O4 | 1.29 | 90.84 | 255.06,227.07,209.05,181.06,165.06,153.06,135.04,121.02 | Chrysophanol or its isomer | Anthraquinone | 3,63,81,92,93 | 481-74-3 |
| 444 | 16.16 | 233.1528 | [M+H]⁺ | C15H20O2 | 3.44 | 81.04 | 233.15,167.08,157.07,145.07,131.08,117.06,105.06,91.05 | Costunolide or its isomer | Terpenoids | 97 | 553-21-9 |
| 445 | 16.17 | 209.0801 | [M+H]⁺ | C11H12O4 | 3.92 | 88.09 | 209.08,163.03,145.02,135.04,117.03,107.04,89.03,79.05 | Caffeic acid ethyl ester | Phenylpropanoids | 87 | 102-37-4 |
| 446 | 16.18 | 320.0924 | [M+H]⁺ | C19H14ClNO4 | -4.07 | 81.82 | 320.09,307.08,292.09,277.07 | Coptisine chloride or its iosmer | Alkaloid | 60 | 6020-18-4 |
| 447 | 16.18 | 320.0888 | [M+H]⁺ | C19H14NO4 | 3.76 | 83.54 | 320.09,292.09 | Coptisine or its isomer | Alkaloid | 2 | 3486-66-6 |
| 448 | 16.19 | 271.0600 | [M+H]⁺ | C15H10O5 | 0.14 | 93.02 | 271.05,197.06 | Emodin or its isomer | Anthraquinone | 3,12,42,92,93 | 518-82-1 |
| 449 | 16.19 | 352.1541 | [M+H]⁺ | C21H22NO4 | -5.63 | 83.33 | 352.15,336.12,322.10,308.12,294.11 | Palmatine or its isomer | Alkaloid | 72 | 3486-67-7 |
| 450 | 16.20 | 336.1243 | [M+H]⁺ | C20H18NO4 | -1.28 | 94.74 | 336.12,321.09,292.09,275.09 | Berberine or its isomer | Alkaloid | 3,72 | 2086-83-1 |
| 451 | 16.21 | 447.0931 | [M+H]⁺ | C21H18O11 | 2.36 | 99.57 | 447.09,271.06,169.01,85.02 | Glychionide or its isomer | Flavonoids | 2,3,7 | 119152-50-0 |
| 452 | 16.21 | 447.1289 | [M+H]⁺ | C22H22O10 | -0.95 | 91.28 | 447.12,285.07 | Tilianin or its isomer | Flavonoids | 41,61 | 4291-60-5 |
| 453 | 16.25 | 247.0964 | [M+H]⁺ | C14H14O4 | -0.66 | 97.80 | 247.09,229.08,175.03 | Decursinol or its isomer | Coumarins | 95 | 23458-02-8 |
| 454 | 16.25 | 247.0960 | [M+H]⁺ | C14H14O4 | -0.81 | 91.18 | 247.09,229.08,175.03 | Columbianetin or its isomer | Coumarins | 5,86 | 3804-70-4 |
| 455 | 16.30 | 315.0854 | [M+H]⁺ | C17H14O6 | -2.25 | 93.61 | 315.08,300.06 | Kumatakenin or its isomer | Flavonoids | 76 | 3301-49-3 |
| 456 | 16.31 | 285.0753 | [M+H]⁺ | C16H12O5 | -1.17 | 98.59 | 285.07,270.05 | 7-O-Methylbaicalein or its isomer | Flavonoids | 1,2,3,5,7,10,11,47,48,49,63,92,100 | 29550-13-8 |
| 457 | 16.31 | 461.1078 | [M+H]⁺ | C22H20O11 | 0.66 | 95.12 | 461.10,285.07 | Wogonin 7-O-glucuronide or its isomer | Flavonoids | 2,3,46,48,49,50,94 | 51059-44-0 |
| 458 | 16.32 | 677.2430 | [M+H]⁺ | C33H40O15 | 0.78 | 96.92 | 677.24,531.18,370.12,314.08,129.05,71.04 | Icariin or its isomer | Flavonoids | 12,89 | 489-32-7 |
| 459 | 16.35 | 531.1854 | [M+H]⁺ | C27H30O11 | -0.35 | 97.01 | 531.18,369.13,313.07,243.06,187.07,135.04 | Anhydroicaritin-7-O-glucoside or its isomer | Flavonoids | 12,89 | 56725-99-6 |
| 460 | 16.38 | 285.0748 | [M+H]⁺ | C16H12O5 | -4.58 | 95.43 | 285.07,270.05 | Wogonin or its isomer | Flavonoids | 33 | 632-85-9 |
| 461 | 16.45 | 163.0392 | [M+H]⁺ | C9H6O3 | 0.86 | 90.38 | 163.03,145.02,117.03 | 7-Hydroxycoumarin or its isomer | Coumarins | 12,13,14,32,42,52,60,61,67,69,70,73,74,75,79,87,88,90,96 | 93-35-6 |
| 462 | 16.47 | 118.0859 | [M+H]⁺ | C5H11NO2 | 0.05 | 86.41 | 118.08,59.07 | Betaine or its isomer | Alkaloid | 16,23,73 | 107-43-7 |
| 463 | 16.49 | 336.1225 | [M+H]⁺ | C20H18NO4 | -1.66 | 94.33 | 336.12,321.09,292.09,275.09 | Berberine or its isomer | Alkaloid | 3,72 | 2086-83-1 |
| 464 | 16.50 | 447.0900 | [M+H]⁺ | C21H18O11 | -0.47 | 99.28 | 447.09,271.06,169.01,85.02 | Glychionide or its isomer | Flavonoids | 7 | 119152-50-0 |
| 465 | 16.53 | 461.1066 | [M+H]⁺ | C22H20O11 | -0.29 | 97.37 | 461.10,285.07 | Wogonin 7-O-glucuronide or its isomer | Flavonoids | 10,11,52,53,100 | 51059-44-0 |
| 466 | 16.63 | 331.0796 | [M+H]⁺ | C17H14O7 | -1.53 | 96.74 | 331.08,298.04 | Jaceosidin or its isomer | Flavonoids | 47 | 18085-97-7 |
| 467 | 16.69 | 299.0910 | [M+H]⁺ | C17H14O5 | 1.19 | 82.83 | 299.09,284.06 | Moslosooflavone or its isomer | Flavonoids | 55 | 740-33-0 |
| 468 | 16.70 | 531.1854 | [M+H]⁺ | C27H30O11 | 2.42 | 96.55 | 531.18,369.13,313.07,243.06,187.07,135.04 | Anhydroicaritin-7-O-glucoside or its isomer | Flavonoids | 12 | 56725-99-6 |
| 469 | 16.72 | 187.0392 | [M+H]⁺ | C11H6O3 | 1.15 | 96.66 | 187.04,159.04,143.04,131.04,115.05 | Psoralen or its isomer | Coumarins | 5,61,86,87,89, | 66-97-7 |
| 470 | 16.73 | 352.1540 | [M+H]⁺ | C21H21NO4 | 1.73 | 82.20 | 352.15,336.12,322.10,308.12,294.11 | Palmatine or its isomer | Alkaloid | 2,70 | 3486-67-7 |
| 471 | 16.80 | 271.0592 | [M+H]⁺ | C15H10O5 | -2.52 | 84.95 | 271.05,197.06 | Emodin or its isomer | Anthraquinone | 93 | 518-82-1 |
| 472 | 16.90 | 358.1382 | [M+H]⁺ | C20H22O6 | 3.76 | 80.90 | 358.13,341.13,137.05 | (-)-Pinoresinol | Lignans | 7 | 81446-29-9 |
| 473 | 16.91 | 632.3058 | [M+H]⁺ | C33H45NO11 | 0.34 | 88.64 | 632.30,572.28 | Mesaconitine | Alkaloid | 87,95 | 2752-64-9 |
| 474 | 16.93 | 187.0386 | [M+H]⁺ | C11H6O3 | 2.49 | 86.47 | 187.03,159.04,143.04,131.04,115.05 | Isopsoralen or its isomer | Coumarins | 89 | 523-50-2 |
| 475 | 17.02 | 331.0810 | [M+H]⁺ | C17H14O7 | -0.05 | 98.73 | 331.08,298.04 | Jaceosidin or its isomer | Flavonoids | 11,52 | 18085-97-7 |
| 476 | 17.07 | 273.0754 | [M+H]⁺ | C15H12O5 | 0.10 | 90.00 | 273.07,153.01 | Naringenin chalcone or its isomer | Chalcones | 40 | 73692-50-9 |
| 477 | 17.08 | 973.4277 | [M+Na]⁺ | C44H70O22 | 0.32 | 99.22 | 973.42,811.37 | Rebaudioside C | Terpenoids | 63 | 63550-99-2 |
| 478 | 17.09 | 233.1526 | [M+H]⁺ | C15H20O2 | -2.75 | 76.01 | 233.15,105.06,91.05 | Isoalantolactone | Terpenoids | 72 | 470-17-7 |
| 479 | 17.10 | 233.1540 | [M+H]⁺ | C15H20O2 | 2.34 | 77.88 | 233.15,177.08 | Pterosin Z or its isomer | Terpenoids | 20 | 34169-69-2 |
| 480 | 17.14 | 271.0954 | [M+H]⁺ | C16H14O4 | -2.36 | 83.80 | 271.09,229.08,121.02 | Echinatin | Chalcones | 5,28,47,56,77 | 34221-41-5 |
| 481 | 17.15 | 359.1485 | [M+H]⁺ | C20H22O6 | 1.56 | 87.45 | 359.14,323.12,291.10,263.10,231.08,205.08,189.09,163.07,137.05 | Matairesinol or its isomer | Lignans | 54 | 580-72-3 |
| 482 | 17.15 | 301.0712 | [M+H]⁺ | C16H12O6 | 0.04 | 82.77 | 301.07,286.04,168.00 | Hispidulin or its isomer | Flavonoids | 1,81 | 1447-88-7 |
| 483 | 17.18 | 271.0600 | [M+H]⁺ | C15H10O5 | 0.71 | 93.10 | 271.06,215.06,153.01,119.04 | Apigenin or its isomer | Flavonoids | 55 | 520-36-5 |
| 484 | 17.20 | 271.0594 | [M+H]⁺ | C15H10O5 | -2.98 | 84.66 | 271.05,215.06,91.05 | Genistein | Flavonoids | 11 | 446-72-0 |
| 485 | 17.21 | 255.0645 | [M+H]⁺ | C15H10O4 | -1.28 | 86.66 | 255.06,227.07,209.05,181.06,165.06,153.06,135.04,121.02 | Chrysophanol or its isomer | Anthraquinone | 93 | 481-74-3 |
| 486 | 17.21 | 351.2139 | [M+H]⁺ | C20H30O5 | -3.99 | 77.72 | 351.21,277.20 | Andrographolide | Terpenoids | 76 | 5508-58-7 |
| 487 | 17.24 | 285.0749 | [M+H]⁺ | C16H12O5 | -4.23 | 85.81 | 285.07,186.02 | Prunetin | Isoflavone | 91 | 552-59-0 |
| 488 | 17.25 | 217.0491 | [M+H]⁺ | C12H8O4 | 2.11 | 90.72 | 217.04,202.02,189.05,173.05,161.05 | Bergapten or its isomer | Coumarins | 41,54,73,84,86,87,90 | 484-20-8 |
| 489 | 17.26 | 285.0757 | [M+H]⁺ | C16H12O5 | -0.46 | 91.03 | 285.07,270.05 | 7-O-Methylbaicalein or its isomer | Flavonoids | 63,81 | 29550-13-8 |
| 490 | 17.26 | 301.0705 | [M+H]⁺ | C16H12O6 | -0.62 | 88.33 | 301.07,286.04 | Tectorigenin or its isomer | Flavonoids | 7 | 548-77-6 |
| 491 | 17.27 | 331.0810 | [M+H]⁺ | C17H14O7 | -0.73 | 99.51 | 331.08,298.04 | Jaceosidin or its isomer | Flavonoids | 10 | 18085-97-7 |
| 492 | 17.29 | 187.0386 | [M+H]⁺ | C11H6O3 | 2.90 | 88.47 | 187.03,159.04,143.04,131.04,115.05 | Isopsoralen or its isomer | Coumarins | 89 | 523-50-2 |
| 493 | 17.32 | 247.0960 | [M+H]⁺ | C14H14O4 | 6.00 | 90.86 | 247.09,229.08,175.03 | Decursinol or its isomer | Coumarins | 86 | 23458-02-8 |
| 494 | 17.35 | 230.0814 | [M+H]⁺ | C13H11NO3 | -0.73 | 96.94 | 230.08,215.05,200.03,186.05,172.03 | Fagarine or its isomer | Alkaloid | 16,60 | 524-15-2 |
| 495 | 17.42 | 287.0549 | [M+H]⁺ | C15H10O6 | -0.34 | 96.61 | 287.05,258.04,165.01,153.01,68.99 | Kaempferol or its isomer | Flavonoids | 39 | 520-18-3 |
| 496 | 17.44 | 118.0851 | [M+H]⁺ | C5H11NO2 | -9.69 | 91.17 | 118.08,59.07 | Betaine or its isomer | Alkaloid | 23 | 107-43-7 |
| 497 | 17.45 | 367.1999 | [M+H]⁺ | C22H26N2O3 | -3.09 | 71.50 | 367.19,170.09,144.08 | Hirsuteine | Alkaloid | 41 | 35467-43-7 |
| 498 | 17.47 | 301.0706 | [M+H]⁺ | C16H12O6 | 1.79 | 79.99 | 301.07,286.04,258.05 | Diosmetin or its isomer | Flavonoids | 61 | 520-34-3 |
| 499 | 17.50 | 303.0857 | [M+H]⁺ | C16H14O6 | 2.42 | 87.00 | 303.08,261.07,219.06,201.05,177.05,153.01,117.03,89.03,67.01 | Hesperetin or its isomer | Flavonoids | 40 | 520-33-2 |
| 500 | 17.57 | 355.1174 | [M+H]⁺ | C20H18O6 | 0.17 | 89.16 | 355.11,299.05,121.02 | Licoflavonol or its isomer | Flavonoids | 69 | 60197-60-6 |
| 501 | 17.57 | 361.0916 | [M+H]⁺ | C18H16O8 | 0.55 | 98.44 | 361.09,346.06 | 5,7,3'-Trihydroxy-6,4',5'-trimethoxyflavone or its isomer | Flavonoids | 5 | 78417-26-2 |
| 502 | 17.59 | 341.1380 | [M+H]⁺ | C20H20O5 | 0.97 | 91.70 | 341.13,323.12,297.11,281.11,263.10,235.11,203.06,149.02 | 6,7-Dimethoxy-2-[2-(4-methoxyphenyl)ethyl]chromone or its isomer | Chromones | 89 | 117596-92-6 |
| 503 | 17.63 | 163.0747 | [M+H]⁺ | C10H10O2 | 4.67 | 89.23 | 163.07,103.05,77.03,51.02 | Methyl cinnamate | Coumarins | 16 | 103-26-4 |
| 504 | 17.66 | 793.4691 | [M+Na]⁺ | C41H70O13 | -1.99 | 99.06 | 793.47,659.10,424.23 | Ginsenoside F5 | Saponin | 27 | 189513-26-6 |
| 505 | 17.66 | 577.3726 | [M+H]⁺ | C33H52O8 | 1.20 | 87.05 | 577.37,433.25,253.19 | Diosgenin glucosid or its isomer | Saponin | 75 | 14144-06-0 |
| 506 | 17.70 | 219.1734 | [M+H]⁺ | C22H15O | -3.31 | 85.01 | 219.17,201.16,177.12,163.10,151.11,135.08,123.11,109.10,97.06,81.06 | (-)-γ-Cuparenol or its isomer | Terpenoids | 15,42,55,61,62,69,71,87 | 1357469-94-3 |
| 507 | 17.73 | 303.2314 | [M+H]⁺ | C20H30O2 | 1.88 | 80.82 | 303.23,199.14,187.14,173.13,159.11,145.10,133.10,121.10,107.08,93.06,81.06,67.05,55.05 | Abietic acid or its isomer | Terpenoids | 84,97 | 514-10-3 |
| 508 | 17.73 | 616.3114 | [M+H]⁺ | C33H45NO10 | 0.05 | 94.90 | 616.31,556.29,524.26,338.18 | Hypaconitine | Alkaloid | 87,95 | 6900-87-4 |
| 509 | 17.74 | 271.0601 | [M+H]⁺ | C15H10O5 | 0.54 | 95.23 | 271.05,253.04,225.05,169.01,123.00,103.05 | Baicalein or its isomer | Flavonoids | 10,46,49,50,51,53 | 491-67-8 |
| 510 | 17.76 | 331.0790 | [M+H]⁺ | C17H14O7 | 1.09 | 94.36 | 331.07,316.05,298.04 | Aurantio-obtusifolin | Anthraquinone | 48,63 | 67979-25-3 |
| 511 | 17.77 | 301.0703 | [M+H]⁺ | C16H12O6 | -2.66 | 90.03 | 301.07,286.04 | Tectorigenin or its isomer | Flavonoids | 5,48,92 | 548-77-6 |
| 512 | 17.77 | 377.1583 | [M+H]⁺ | C20H24O7 | -3.31 | 88.25 | 377.15,259.09,191.03,160.05,131.04 | Angelol B | Coumarins | 29 | 83156-04-1 |
| 513 | 17.78 | 271.0594 | [M+H]⁺ | C15H10O5 | -1.77 | 87.27 | 271.06,215.06,153.01,119.04 | Apigenin or its isomer | Flavonoids | 7,10 | 520-36-5 |
| 514 | 17.84 | 207.0644 | [M+H]⁺ | C11H10O4 | -2.83 | 85.69 | 207.06,91.05 | Citropten | Coumarins | 5 | 487-06-9 |
| 515 | 17.91 | 501.1743 | [M+H]⁺ | C26H28O10 | 1.03 | 92.72 | 501.17,355.11,299.05,129.05,85.02 | Baohuoside II or its isomer | Flavonoids | 89 | 55395-07-8 |
| 516 | 17.94 | 373.1278 | [M+H]⁺ | C20H20O7 | -0.09 | 92.28 | 373.12,343.08 | Isosinensetin or its isomer | Flavonoids | 73 | 17290-70-9 |
| 517 | 17.96 | 301.0712 | [M+H]⁺ | C16H12O6 | -1.53 | 87.71 | 301.07,286.04,258.05 | Diosmetin or its isomer | Flavonoids | 98 | 520-34-3 |
| 518 | 17.98 | 373.1277 | [M+H]⁺ | C20H20O7 | 0.21 | 90.42 | 373.12,358.10,343.07 | Tangeretin or its isomer | Flavonoids | 40 | 481-53-8 |
| 519 | 18.07 | 231.1012 | [M+H]⁺ | C14H14O3 | 0.72 | 85.62 | 231.10,175.03 | 7-Demethylsuberosin or its isomer | Coumarins | 89 | 21422-04-8 |
| 520 | 18.09 | 118.0854 | [M+H]⁺ | C5H11NO2 | -7.76 | 92.45 | 118.08,59.07 | Betaine or its isomer | Alkaloid | 23,82 | 107-43-7 |
| 521 | 18.11 | 401.1956 | [M+H]⁺ | C23H28O6 | 2.63 | 80.39 | 401.19,386.17,370.17,333.14 | Schisandrin B or its isomer | Lignans | 13,54 | 61281-37-6 |
| 522 | 18.15 | 217.0493 | [M+H]⁺ | C12H8O4 | 1.19 | 94.40 | 217.04,202.02,189.05,173.05,161.05 | Bergapten or its isomer | Coumarins | 29,40,42,45,52,61,73,86,87,95 | 484-20-8 |
| 523 | 18.19 | 389.1225 | [M+H]⁺ | C20H20O8 | 4.82 | 86.25 | 389.12,374.09,359.07 | 5-O-Demethylnobiletin or its isomer | Flavonoids | 10 | 2174-59-6 |
| 524 | 18.23 | 191.1073 | [M+H]⁺ | C12H14O2 | 2.56 | 72.80 | 191.10,173.09,163.11,145.10,133.06,117.07,105.06,91.05,79.05 | Z-Ligustilide or its isomer | Phthalides | 23 | 4431-01-0 |
| 525 | 18.24 | 343.1541 | [M+H]⁺ | C20H22O5 | 0.61 | 89.58 | 343.15,219.10 | Wulignan A1 | Lignans | 25 | 117047-76-4 |
| 526 | 18.25 | 247.0601 | [M+H]⁺ | C13H10O5 | -0.14 | 93.80 | 247.05,232.03,217.01,189.01,173.13,161.02 | Isopimpinellin or its isomer | Coumarins | 29,52,61,73,86,87 | 482-27-9 |
| 527 | 18.25 | 257.0803 | [M+H]⁺ | C15H12O4 | 3.41 | 79.43 | 257.08,239.06,211.07,163.03,147.04,137.02,123.03 | Isoliquiritigenin or its isomer | Chalcones | 9,47,73,77,95 | 961-29-5 |
| 528 | 18.25 | 277.1071 | [M+H]⁺ | C15H16O5 | -1.64 | 96.17 | 277.10,205.04 | Hamaudol or its isomer | Flavonoids | 29 | 735-46-6 |
| 529 | 18.26 | 337.1064 | [M+H]⁺ | C20H16O5 | 1.63 | 83.42 | 337.10,319.09,279.06 | Alpinumisoflavone | Flavonoids | 89 | 34086-50-5 |
| 530 | 18.34 | 255.0644 | [M+H]⁺ | C15H10O4 | -2.79 | 91.42 | 255.06,227.07,209.05,181.06,165.06,153.06,135.04,121.02 | Chrysophanol or its isomer | Anthraquinone | 3 | 481-74-3 |
| 531 | 18.34 | 269.0808 | [M+H]⁺ | C16H12O4 | 0.68 | 89.91 | 269.08,254.05,237.05,213.09,197.05,136.01,118.04 | Formononetin or its isomer | Flavonoids | 9,44,73 | 485-72-3 |
| 532 | 18.36 | 501.1743 | [M+H]⁺ | C26H28O10 | 3.03 | 91.04 | 501.17,355.11,299.05,129.05,85.02 | Baohuoside II or its isomer | Flavonoids | 89 | 55395-07-8 |
| 533 | 18.37 | 269.0806 | [M+H]⁺ | C16H12O4 | -0.87 | 95.44 | 269.08,254.05,226.06,139.10 | Tectochrysin | Flavonoids | 12,23,40,55,56,86,95 | 520-28-5 |
| 534 | 18.42 | 235.1690 | [M+H]⁺ | C15H22O2 | -1.41 | 76.97 | 235.16,217.15,201.16,189.16,175.11,161.09,147.11,133.10,119.08,107.08,85.06,71.04,59.04 | Curcumenol or its isomer | Terpenoids | 72 | 19431-84-6 |
| 535 | 18.46 | 373.1646 | [M+H]⁺ | C21H24O6 | 0.33 | 95.00 | 373.16,355.15,323.12,305.11,237.10,177.09,151.0,137.05 | Arctigenin or its isomer | Lignans | 54 | 7770-78-7 |
| 536 | 18.53 | 341.1380 | [M+H]⁺ | C20H20O5 | 2.41 | 88.63 | 341.13,285.07,270.10,149.02 | 8-Prenylnaringenin | Flavonoids | 89 | 53846-50-7 |
| 537 | 18.55 | 287.2003 | [M+H]⁺ | C19H26O2 | 0.47 | 89.66 | 287.20,269.10,109.06.97.06 | Androstenedione or its isomer | Steroids | 55 | 63-05-8 |
| 538 | 18.63 | 289.0701 | [M+H]⁺ | C15H12O6 | 2.97 | 79.05 | 289.07,163.03 | Eriodictyol or its isomer | Flavonoids | 89 | 552-58-9 |
| 539 | 18.64 | 118.0857 | [M+H]⁺ | C5H11NO2 | 2.43 | 91.13 | 118.08,59.07 | Betaine or its isomer | Alkaloid | 82 | 107-43-7 |
| 540 | 18.70 | 287.0920 | [M+H]⁺ | C16H14O5 | 0.06 | 94.03 | 287.09,203.03,85.06 | Oxypeucedanin or its isomer | Coumarins | 73,86,87,95 | 737-52-0 |
| 541 | 18.76 | 317.1015 | [M+H]⁺ | C17H16O6 | 0.44 | 82.48 | 317.10.299.09,273.07,257.08,231.02,215.03,203.03,175.03,85.06,67.05 | Byakangelicol or its isomer | Coumarins | 87 | 26091-79-2 |
| 542 | 18.76 | 373.1278 | [M+H]⁺ | C20H20O7 | 0.13 | 92.61 | 373.12,358.10,343.07 | Tangeretin or its isomer | Flavonoids | 73 | 481-53-8 |
| 543 | 18.76 | 373.1277 | [M+H]⁺ | C20H20O7 | 1.99 | 93.61 | 373.12,343.08 | Isosinensetin or its isomer | Flavonoids | 40,99 | 17290-70-9 |
| 544 | 18.78 | 529.3527 | [M+H]⁺ | C32H48O6 | 0.40 | 88.82 | 529.35,511.34,469.33,451.31,405.31 | 23-Acetyl alisol C or its isomer | Terpenoids | 47 | 26575-93-9 |
| 545 | 18.80 | 373.1272 | [M+H]⁺ | C20H20O7 | 3.38 | 76.55 | 373.12,343.08,312.09 | Sinensetin | Flavonoids | 39 | 2306-27-6 |
| 546 | 18.81 | 343.1182 | [M+H]⁺ | C19H18O6 | -2.05 | 96.07 | 343.11,328.09,313.07,285.07 | 5,7,8,4'-Tetramethoxyflavone or its isomer | Flavonoids | 40,73 | 6601-66-7 |
| 547 | 18.89 | 231.1012 | [M+H]⁺ | C14H14O3 | -1.13 | 92.83 | 231.10,175.03,147.04,119.04,91.05,65.03 | Osthenol | Coumarins | 5 | 484-14-0 |
| 548 | 18.91 | 339.1218 | [M+H]⁺ | C20H18O5 | 1.74 | 90.75 | 339.12,283.05 | Corylifol C or its isomer | Flavonoids | 89 | 775351-91-2 |
| 549 | 18.91 | 369.1323 | [M+H]⁺ | C21H20O6 | 1.77 | 98.29 | 369.13,313.06 | Glycycoumarin or its isomer | Coumarins | 5,47,89 | 94805-82-0 |
| 550 | 18.91 | 303.1226 | [M+H]⁺ | C17H18O5 | 0.17 | 80.98 | 303.12,167.07,76.07 | Isomucronulatol or its isomer | Flavonoids | 76 | 52250-35-8 |
| 551 | 18.93 | 285.0760 | [M+H]⁺ | C16H12O5 | -2.50 | 93.15 | 285.07,270.05 | 7-O-Methylbaicalein or its isomer | Flavonoids | 1,3,10 | 29550-13-8 |
| 552 | 18.93 | 285.0768 | [M+H]⁺ | C16H12O5 | 2.76 | 99.29 | 285.07,270.05 | Wogonin or its isomer | Flavonoids | 33 | 632-85-9 |
| 553 | 19.01 | 317.2103 | [M+H]⁺ | C20H28O3 | -0.79 | 79.63 | 317.20,281.19 | Cafestol or its isomer | Terpenoids | 52 | 469-83-0 |
| 554 | 19.06 | 395.1456 | [M+H]⁺ | C23H22O6 | 7.66 | 80.01 | 395.14,192.07 | Rotenone | Flavonoids | 40 | 83-79-4 |
| 555 | 19.08 | 203.0330 | [M+H]⁺ | C11H6O4 | 3.19 | 94.11 | 203.03,185.02,175.03,159.04,147.04,131.05,119.04,103.05,91.05,77.03 | Xanthotoxol or its isomer | Coumarins | 40 | 2009-24-7 |
| 556 | 19.10 | 285.0747 | [M+H]⁺ | C16H12O5 | -3.40 | 89.75 | 285.07,168.00 | Acacetin | Flavonoids | 33 | 480-44-4 |
| 557 | 19.11 | 361.0903 | [M+H]⁺ | C18H16O8 | -0.17 | 99.33 | 361.09,346.06 | 5,7,3'-Trihydroxy-6,4',5'-trimethoxyflavone or its isomer | Flavonoids | 10 | 78417-26-2 |
| 558 | 19.13 | 325.1413 | [M+H]⁺ | C20H20O4 | 7.70 | 82.12 | 325.14,307.12,283.14,252.10 | Bavachalcone or its isomer | Chalcones | 32 | 28448-85-3 |
| 559 | 19.15 | 233.1536 | [M+H]⁺ | C15H20O2 | 2.56 | 85.07 | 233.15,177.08 | Pterosin Z or its isomer | Terpenoids | 58 | 34169-69-2 |
| 560 | 19.19 | 531.1855 | [M+H]⁺ | C27H30O11 | 2.22 | 97.81 | 531.18,369.13,313.07,243.06,187.07,135.04 | Anhydroicaritin-7-O-glucoside or its isomer | Flavonoids | 12,29,89,94 | 56725-99-6 |
| 561 | 19.22 | 294.1488 | [M+H]⁺ | C19H19NO2 | 0.67 | 91.75 | 294.14,279.12,263.13 | Dehydronuciferin | Alkaloid | 32 | 7630-74-2 |
| 562 | 19.27 | 257.0804 | [M+H]⁺ | C15H12O4 | 1.72 | 88.30 | 257.07,137.02 | Pinocembrin or its isomer | Flavonoids | 56,65 | 480-39-7 |
| 563 | 19.33 | 322.1074 | [M+H]⁺ | C19H15NO4 | -2.3 | 89.88 | 322.10,308.09,293.10,278.07,262.08,234.09 | Menisporphine | Alkaloid | 66 | 83287-02-9 |
| 564 | 19.34 | 369.1338 | [M+H]⁺ | C21H20O6 | 0.77 | 92.62 | 369.13,313.06 | Glycycoumarin or its isomer | Coumarins | 29,89,95 | 94805-82-0 |
| 565 | 19.37 | 345.0967 | [M+H]⁺ | C18H16O7 | -1.16 | 88.65 | 345.09,330.07,315.04 | Lysionotin or its isomer | Flavonoids | 7 | 152743-19-6 |
| 566 | 19.43 | 515.1900 | [M+H]⁺ | C27H30O10 | 0.77 | 81.94 | 515.19,369.12,313.07,71.05 | Baohuoside I or its isomer | Flavonoids | 89 | 113558-15-9 |
| 567 | 19.46 | 285.0753 | [M+H]⁺ | C16H12O5 | -1.68 | 99.65 | 285.07,270.05 | 7-O-Methylbaicalein or its isomer | Flavonoids | 1,3,4,5,7,11,47,48,63,92,94,100 | 29550-13-8 |
| 568 | 19.46 | 401.1949 | [M+H]⁺ | C23H28O6 | 2.24 | 82.06 | 401.19,386.17,371.18,331.11,300.09 | Gomisin N or its isomer | Lignans | 25 | 69176-52-9 |
| 569 | 19.48 | 529.3503 | [M+H]⁺ | C32H48O6 | 3.96 | 87.95 | 529.35,511.34,469.33,451.31,415.28 | 23-Acetyl alisol C or its isomer | Terpenoids | 23 | 26575-93-9 |
| 570 | 19.50 | 197.0791 | [M+H]⁺ | C10H12O4 | -9.73 | 85.33 | 197.07,181.06 | Xanthoxylin | Alkaloid | 62 | 90-24-4 |
| 571 | 19.52 | 338.1388 | [M+H]⁺ | C20H19NO4 | 0.49 | 85.49 | 338.13,322.10,308.09,294.11,280.09,262.08,250.08,222.09 | Jatrorrhizine or its isomer | Alkaloid | 55 | 3621-38-3 |
| 572 | 19.53 | 285.0776 | [M+H]⁺ | C16H12O5 | 5.14 | 95.65 | 285.07,270.05 | Wogonin or its isomer | Flavonoids | 81 | 632-85-9 |
| 573 | 19.59 | 255.0644 | [M+H]⁺ | C15H10O4 | 0.93 | 91.28 | 255.06,237.05 | Chrysin or its isomer | Flavonoids | 98 | 480-40-0 |
| 574 | 19.64 | 323.1253 | [M+H]⁺ | C20H18O4 | 1.17 | 87.43 | 323.12,267.06 | 8-Prenyldaidzein or its isomer | Flavonoids | 56 | 135384-00-8 |
| 575 | 19.65 | 315.0853 | [M+H]⁺ | C17H14O6 | -2.04 | 92.20 | 315.08,300.06 | Kumatakenin or its isomer | Flavonoids | 1,5,33,48,52,92 | 3301-49-3 |
| 576 | 19.72 | 343.1169 | [M+H]⁺ | C19H18O6 | 3.37 | 88.48 | 343.11,328.09,313.07,285.07 | 5,7,8,4'-Tetramethoxyflavone or its isomer | Flavonoids | 40,73,89 | 6601-66-7 |
| 577 | 19.76 | 357.1696 | [M+H]⁺ | C21H24O5 | 8.70 | 79.13 | 357.16,233.11,218.09,191.10,165.05 | (-)-Holostyligone | Phenylpropanoids | 25 | 887501-28-2 |
| 578 | 19.79 | 317.1015 | [M+H]⁺ | C17H16O6 | 0.53 | 91.14 | 317.10.299.09,273.07,257.08,231.02,215.03,203.03,175.03,85.06,67.05 | Byakangelicol or its isomer | Coumarins | 73,87 | 26091-79-2 |
| 579 | 19.79 | 375.1066 | [M+H]⁺ | C19H18O8 | -2.04 | 89.83 | 375.10,360.08 | Vitexicarpin | Flavonoids | 94 | 479-91-4 |
| 580 | 19.81 | 285.0748 | [M+H]⁺ | C16H12O5 | -3.68 | 95.47 | 285.07,270.05 | Wogonin or its isomer | Flavonoids | 5,10,33,48,50,52,81 | 632-85-9 |
| 581 | 19.82 | 341.1380 | [M+H]⁺ | C20H20O5 | -0.66 | 90.16 | 341.13,323.12,297.11,281.11,263.10,235.11,203.06,149.02 | 6,7-Dimethoxy-2-[2-(4-methoxyphenyl)ethyl]chromone or its isomer | Chromones | 89 | 117596-92-6 |
| 582 | 19.83 | 277.1799 | [M+H]⁺ | C17H24O3 | -0.28 | 94.42 | 277.17,137.05,122.03 | 6-Shogaol or its isomer | Phenolics | 39 | 555-66-8 |
| 583 | 19.86 | 352.1183 | [M+H]⁺ | C20H17NO5 | -0.54 | 97.83 | 352.11,337.09,322.07,308.09,294.07 | 8-Oxoberberine or its isomer | Alkaloid | 3,16,25,60,72 | 549-21-3 |
| 584 | 19.86 | 287.0917 | [M+H]⁺ | C16H14O5 | -0.43 | 88.97 | 287.09,203.03,85.06 | Oxypeucedanin or its isomer | Coumarins | 73,95 | 737-52-0 |
| 585 | 19.87 | 287.0912 | [M+H]⁺ | C16H14O5 | 1.56 | 89.65 | 287.09,203.07 | Isosakuranetin or its isomer | Flavonoids | 86 | 480-43-3 |
| 586 | 19.88 | 285.0753 | [M+H]⁺ | C16H12O5 | -1.56 | 99.33 | 285.07,270.05 | 7-O-Methylbaicalein or its isomer | Flavonoids | 1,10,11,100 | 29550-13-8 |
| 587 | 19.92 | 209.1171 | [M+H]⁺ | C12H16O3 | 0.64 | 90.38 | 209.11,194.09,181.08,168.07,153.05 | Elemicin | Phenylpropanoids | 95 | 487-11-6 |
| 588 | 19.92 | 369.1323 | [M+H]⁺ | C21H20O6 | 2.52 | 95.78 | 369.13,313.06 | Glycycoumarin or its isomer | Coumarins | 5,12,29,63,89,92 | 94805-82-0 |
| 589 | 19.92 | 301.0695 | [M+H]⁺ | C16H12O6 | 0.77 | 81.60 | 301.07,286.04 | Tectorigenin or its isomer | Flavonoids | 65 | 548-77-6 |
| 590 | 19.93 | 433.2219 | [M+H]⁺ | C24H32O7 | 1.10 | 86.92 | 433.22,415.21,400.18,384.19,373.16,359.14,346.14,329.13 | Schisandrol A | Lignans | 13,54,56,83,88,98 | 7432-28-2 |
| 591 | 19.94 | 315.0863 | [M+H]⁺ | C17H14O6 | 0.59 | 90.45 | 315.08,300.06 | Kumatakenin or its isomer | Flavonoids | 3,5,7,47 | 3301-49-3 |
| 592 | 19.95 | 515.1900 | [M+H]⁺ | C27H30O10 | 2.69 | 96.76 | 515.19,369.12,313.07,71.05 | Baohuoside I or its isomer | Flavonoids | 89 | 113558-15-9 |
| 593 | 20.06 | 341.1376 | [M+H]⁺ | C20H20O5 | 2.00 | 97.70 | 341.13,323.12,297.11,281.11,263.10,235.11,203.06,149.02 | 6,7-Dimethoxy-2-[2-(4-methoxyphenyl)ethyl]chromone or its isomer | Chromones | 94 | 117596-92-6 |
| 594 | 20.10 | 231.1014 | [M+H]⁺ | C14H14O3 | 1.48 | 93.10 | 231.10,175.03 | 7-Demethylsuberosin or its isomer | Coumarins | 86,95 | 21422-04-8 |
| 595 | 20.11 | 323.1269 | [M+H]⁺ | C20H18O4 | -0.90 | 94.44 | 323.12,267.06 | 8-Prenyldaidzein or its isomer | Flavonoids | 71,89 | 135384-00-8 |
| 596 | 20.13 | 433.1490 | [M+H]⁺ | C22H24O9 | 0.37 | 96.90 | 433.14,418.12,403.10 | 3',4',3,5,6,7,8-Heptamethoxyflavone | Flavonoids | 34,39,40,72,73,85 | 1178-24-1 |
| 597 | 20.16 | 235.1687 | [M+H]⁺ | C15H22O2 | 2.08 | 83.35 | 235.16,217.15,201.16,189.16,175.11,161.09,147.11,133.10,119.08,107.08,85.06,71.04,59.04 | Curcumenol or its isomer | Terpenoids | 81,97 | 19431-84-6 |
| 598 | 20.17 | 303.2314 | [M+H]⁺ | C20H30O2 | 1.92 | 82.69 | 303.23,199.14,187.14,173.13,159.11,145.10,133.10,119.08,105.06,93.06,81.06,67.05,55.05 | Abietic acid or its isomer | Terpenoids | 84,97 | 514-10-3 |
| 599 | 20.25 | 311.1272 | [M+H]⁺ | C19H18O4 | 0.42 | 97.38 | 311.12,220.07,205.04,91.05 | 6,7-Dimethoxy-2-phenetylchromone | Chromones | 94 | 84294-87-1 |
| 600 | 20.26 | 193.1221 | [M+H]⁺ | C12H16O2 | 1.58 | 92.37 | 193.12,175.11,165.12,157.10,147.11,137.05,119.08,105.06,93.06,81.07,69.07,55.05 | Senkyunolide | Phthalides | 5,13,16,24,40,41,42,55,84,86,87,94 | 63038-10-8 |
| 601 | 20.31 | 231.1381 | [M+H]⁺ | C15H18O2 | 1.10 | 79.59 | 233.15,215.14,197.13,187.14,177.09,151.07,133.06,95.08 | Atractylenolide II or its isomer | Terpenoids | 77 | 73069-13-3 |
| 602 | 20.32 | 331.0796 | [M+H]⁺ | C17H14O7 | -2.11 | 92.77 | 331.07,316.05,298.04 | Aurantio-obtusifolin | Anthraquinone | 62 | 67979-25-3 |
| 603 | 20.35 | 345.0956 | [M+H]⁺ | C18H16O7 | -3.39 | 97.30 | 345.09,330.07,315.04 | Lysionotin or its isomer | Flavonoids | 3,5,47,48,81 | 152743-19-6 |
| 604 | 20.37 | 323.1277 | [M+H]⁺ | C20H18O4 | 1.76 | 94.39 | 323.12,267.06,239.06 | Licoflavone A | Flavonoids | 89 | 61153-77-3 |
| 605 | 20.37 | 271.0961 | [M+H]⁺ | C16H14O4 | 0.64 | 89.48 | 271.09,229.04 | Alloimperatorin | Coumarins | 73 | 642-05-7 |
| 606 | 20.40 | 163.0380 | [M+H]⁺ | C9H6O3 | -7.12 | 85.76 | 163.03,145.02,117.03 | 7-Hydroxycoumarin or its isomer | Coumarins | 5 | 93-35-6 |
| 607 | 20.47 | 373.1278 | [M+H]⁺ | C20H20O7 | 1.30 | 94.16 | 373.12,358.10,343.07 | Tangeretin or its isomer | Flavonoids | 40,73 | 481-53-8 |
| 608 | 20.48 | 341.1380 | [M+H]⁺ | C20H20O5 | 1.16 | 87.80 | 341.13,323.12,297.11,281.11,263.10,235.11,203.06,149.02 | 6,7-Dimethoxy-2-[2-(4-methoxyphenyl)ethyl]chromone or its isomer | Chromones | 25,32,89 | 117596-92-6 |
| 609 | 20.49 | 315.0849 | [M+H]⁺ | C17H14O6 | 3.61 | 88.32 | 315.08,300.06 | Kumatakenin or its isomer | Flavonoids | 56 | 3301-49-3 |
| 610 | 20.49 | 286.1425 | [M+H]⁺ | C17H19NO3 | 3.65 | 84.16 | 286.14,201.05 | Piperine | Alkaloid | 65 | 94-62-2 |
| 611 | 20.51 | 325.1431 | [M+H]⁺ | C20H20O4 | -0.81 | 95.87 | 325.14,307.12,283.14,252.10 | Bavachalcone or its isomer | Chalcones | 89 | 28448-85-3 |
| 612 | 20.52 | 191.1069 | [M+H]⁺ | C12H14O2 | 0.18 | 86.68 | 191.10,173.09,163.11,145.10,133.06,117.07,105.06,91.05,79.05 | Z-Ligustilide or its isomer | Phthalides | 5,41,42 | 4431-01-0 |
| 613 | 20.53 | 191.1065 | [M+H]⁺ | C12H14O2 | 1.90 | 85.85 | 191.10,173.09,155.09,145.10,135.04,117.06,105.07,91.05,79.05,67.05,57.03 | 3-n-Butylphathlide or its isomer | Phthalides | 13,16,23,28,40,55,61,84,87 | 6066-49-5 |
| 614 | 20.57 | 373.1291 | [M+H]⁺ | C20H20O7 | -0.18 | 82.77 | 373.12,343.08 | Isosinensetin or its isomer | Flavonoids | 34 | 17290-70-9 |
| 615 | 20.65 | 288.1126 | [M+H]⁺ | C18H13N3O | -1.67 | 88.24 | 288.11,273.08,244.08,216.08,169.07,145.03,120.04,92.04,65.03 | Rutaecarpine | Alkaloid | 72 | 84-26-4 |
| 616 | 20.96 | 303.2314 | [M+H]⁺ | C20H30O2 | 2.71 | 76.35 | 303.23,199.14,187.14,173.13,159.11,145.10,133.10,119.08,105.06,93.06,81.06,67.05,55.05 | Abietic acid or its isomer | Terpenoids | 97 | 514-10-3 |
| 617 | 21.02 | 355.1177 | [M+H]⁺ | C20H18O6 | 0.33 | 93.67 | 355.11,299.05,121.02 | Licoflavonol or its isomer | Flavonoids | 47 | 60197-60-6 |
| 618 | 21.04 | 325.1413 | [M+H]⁺ | C20H20O4 | 7.45 | 71.35 | 325.14,307.12,283.14,252.10 | Bavachalcone or its isomer | Chalcones | 26 | 28448-85-3 |
| 619 | 21.13 | 191.1057 | [M+H]⁺ | C12H14O2 | 3.69 | 90.66 | 191.10,173.09,163.11,145.10,133.06,117.07,105.06,91.05,79.05 | Z-Ligustilide or its isomer | Phthalides | 5,13,16,28,40,41,42,47,55,56,60,61,87,94 | 4431-01-0 |
| 620 | 21.14 | 317.1022 | [M+H]⁺ | C17H16O6 | 0.24 | 82.09 | 317.10.299.09,273.07,257.08,231.02,215.03,203.03,175.03,85.06,67.05 | Byakangelicol or its isomer | Coumarins | 52 | 26091-79-2 |
| 621 | 21.15 | 339.1589 | [M+H]⁺ | C21H22O4 | 0.44 | 94.20 | 339.15,297.14,271.09,245.11,219.10,147.04,121.02,93.03 | Licochalcone E | Chalcones | 56 | 864232-34-8 |
| 622 | 21.16 | 417.1801 | [M+H]⁺ | C23H28O7 | 2.92 | 90.77 | 417.18,384.15,369.16,357.13,337.14,330.10 | Gomisin O | Lignans | 54 | 72960-22-6 |
| 623 | 21.16 | 339.1591 | [M+H]⁺ | C21H22O4 | -0.21 | 90.40 | 339.15,297.14,271.09,245.11,121.02 | Licochalcone A | Chalcones | 73 | 58749-22-7 |
| 624 | 21.19 | 321.1124 | [M+H]⁺ | C20H16O4 | -0.68 | 94.95 | 321.11,306.08,293.11,279.06,211.07,137.02 | Corylin | Chalcones | 89 | 53947-92-5 |
| 625 | 21.23 | 303.2314 | [M+H]⁺ | C20H30O2 | 1.55 | 81.92 | 303.23,199.14,187.14,173.13,157.10,145.09,133.10,119.08,105.06,95.08,81.06,67.05,55.05 | Abietic acid or its isomer | Terpenoids | 97 | 514-10-3 |
| 626 | 21.23 | 275.2001 | [M+H]⁺ | C18H26O2 | 1.03 | 91.74 | 275.20,257.18,199.14,145.10,109.06 | Nandrolone | Steroids | 25 | 434-22-0 |
| 627 | 21.30 | 163.0393 | [M+H]⁺ | C9H6O3 | 1.72 | 87.12 | 163.03,145.02,117.03 | 7-Hydroxycoumarin or its isomer | Coumarins | 18,59,60,65,69,70,73,74,78,80,81,90 | 93-35-6 |
| 628 | 21.32 | 271.0970 | [M+H]⁺ | C16H14O4 | -0.80 | 93.19 | 271.09,203.03,175.03,147.04,131.04,69.07 | Isoimperatorin or its isomer | Coumarins | 86 | 482-45-1 |
| 629 | 21.34 | 389.1225 | [M+H]⁺ | C20H20O8 | 3.44 | 86.51 | 389.12,374.09,359.07 | 5-O-Demethylnobiletin or its isomer | Flavonoids | 40 | 2174-59-6 |
| 630 | 21.34 | 203.0337 | [M+H]⁺ | C11H6O4 | -5.66 | 92.77 | 203.03,185.02,175.03,159.04,147.04,131.05,119.04,103.05,91.05,77.03 | Xanthotoxol or its isomer | Coumarins | 5,42,52,86,87,90 | 2009-24-7 |
| 631 | 21.36 | 323.1277 | [M+H]⁺ | C20H18O4 | -0.81 | 95.71 | 323.12,267.06,203.06,147.04 | Licoflavone A | Flavonoids | 89 | 61153-77-3 |
| 632 | 21.37 | 339.1230 | [M+H]⁺ | C20H18O5 | 1.54 | 95.23 | 339.12,283.05 | Corylifol C or its isomer | Flavonoids | 47,77 | 775351-91-2 |
| 633 | 21.38 | 311.1272 | [M+H]⁺ | C19H18O4 | 2.19 | 96.48 | 311.12,190.06,151.03,121.06,91.05,77.03 | 6-Methoxy-2-[2-(3-methoxyphenyl)ethyl]chromone | Chromones | 94 | 111286-05-6 |
| 634 | 21.39 | 401.1958 | [M+H]⁺ | C23H28O6 | 3.06 | 82.75 | 401.19,386.17,370.17,333.14 | Schisandrin B or its isomer | Lignans | 56 | 61281-37-6 |
| 635 | 21.39 | 271.0963 | [M+H]⁺ | C16H14O4 | -0.04 | 96.65 | 271.09,215.03,203.03,147.04,69.06 | Imperatorin or its isomer | Coumarins | 87 | 482-44-0 |
| 636 | 21.40 | 231.1380 | [M+H]⁺ | C15H18O2 | 0.53 | 86.76 | 231.13,165.06,128.06,91.05,55.05 | Dehydrocostuslactone or its isomer | Terpenoids | 97 | 477-43-0 |
| 637 | 21.41 | 401.1975 | [M+H]⁺ | C23H28O6 | 0.08 | 89.14 | 401.19,386.17,371.18,331.11,300.09 | Gomisin N or its isomer | Lignans | 13,54,88 | 69176-52-9 |
| 638 | 21.48 | 195.1378 | [M+H]⁺ | C12H18O2 | 0.58 | 91.73 | 195.13,177.12,159.11,149,13,135.11,125.05,111.04,97.06,79.05,67.05,57.03 | Neocnidilide | Phthalides | 5,13,16,24,40,41,42,61,86,87 | 4567-33-3 |
| 639 | 21.50 | 325.1431 | [M+H]⁺ | C20H20O4 | 1.31 | 94.72 | 325.14,269.07,257.08,227.07,205.08,163.03,149.02,121.02,93.03,69.06 | Bavachin | Chalcones | 89 | 19879-32-4 |
| 640 | 21.54 | 337.1064 | [M+H]⁺ | C20H16O5 | 2.93 | 83.93 | 337.10,305.07,281.04,253.04,237.05,209.05,181.06,152.06,121.02,68.99 | Psoralidin | Coumarins | 89 | 18642-23-4 |
| 641 | 21.56 | 299.1278 | [M+H]⁺ | C18H18O4 | 0.12 | 96.64 | 299.12,281.11,239.10,203.07,177.05,105.06 | Phenylethyl-3-methylcaffeate | Phenolics | 5,42 | 71835-85-3 |
| 642 | 21.57 | 391.2114 | [M+H]⁺ | C22H30O6 | 0.43 | 94.32 | 391.20,359.18,327.15,289.14,237.14,205.12,167.06 | Pregomisin | Lignans | 13,54,83 | 66280-26-0 |
| 643 | 21.59 | 389.1239 | [M+H]⁺ | C20H20O8 | -1.77 | 86.83 | 389.12,374.09,359.07 | 5-O-Demethylnobiletin or its isomer | Flavonoids | 45 | 2174-59-6 |
| 644 | 21.59 | 191.1063 | [M+H]⁺ | C12H14O2 | 1.09 | 93.83 | 191.10,173.09,163.11,145.10,133.06,117.07,105.06,91.05,79.05 | Z-Ligustilide or its isomer | Phthalides | 5,13,16,28,40,41,42,47,55,56,60,61,86,87,94 | 4431-01-0 |
| 645 | 21.61 | 203.0333 | [M+H]⁺ | C11H6O4 | -3.84 | 85.46 | 203.03,175.03,159.04,147.04,131.04,119.04,103.05,91.05,77.03,65.03 | Bergaptol or its isomer | Coumarins | 52 | 486-60-2 |
| 646 | 21.61 | 279.1015 | [M+H]⁺ | C18H14O3 | 0.83 | 95.16 | 279.10,261.09,233.09,205.10 | Dihydrotanshinone I or its isomer | Quinones | 13,23,25,26,32,56,69,89 | 87205-99-0 |
| 647 | 21.65 | 231.1380 | [M+H]⁺ | C15H18O2 | 1.36 | 81.94 | 231.13,165.06,128.06,91.05,55.05 | Dehydrocostuslactone or its isomer | Terpenoids | 97 | 477-43-0 |
| 648 | 21.71 | 299.0910 | [M+H]⁺ | C17H14O5 | 0.79 | 93.01 | 299.09,284.06 | Moslosooflavone or its isomer | Flavonoids | 10,55 | 740-33-0 |
| 649 | 21.73 | 219.1742 | [M+H]⁺ | C15H22O | 0.53 | 91.45 | 219.17,177.12,159.11,135.07,121.06,97.10,77.03,55.05 | Aristolone | Terpenoids | 25,86 | 6831-17-0 |
| 650 | 21.76 | 401.1956 | [M+H]⁺ | C23H28O6 | 2.28 | 88.28 | 401.19,386.17,371.18,331.11,300.09 | Gomisin N or its isomer | Lignans | 13 | 69176-52-9 |
| 651 | 21.80 | 385.1651 | [M+H]⁺ | C22H24O6 | -0.34 | 87.09 | 385.16,355.15,285.07 | Schisandrin C or its isomer | Lignans | 83,94 | 61301-33-5 |
| 652 | 21.82 | 529.3514 | [M+H]⁺ | C32H48O6 | 2.10 | 85.63 | 529.35,511.34,469.33,451.31,415.28 | 23-Acetyl alisol C or its isomer | Terpenoids | 16 | 26575-93-9 |
| 653 | 21.84 | 233.1530 | [M+H]⁺ | C15H20O2 | -0.08 | 89.37 | 233.15,177.08 | Pterosin Z or its isomer | Terpenoids | 25 | 34169-69-2 |
| 654 | 21.86 | 401.1958 | [M+H]⁺ | C23H28O6 | 0.55 | 89.03 | 401.19,386.17,371.18,339.15,331.11 | Schisandrin B or its isomer | Lignans | 13,56,83 | 61281-37-6 |
| 655 | 21.88 | 245.1163 | [M+H]⁺ | C15H16O3 | 5.09 | 84.94 | 245.11,189.05 | Osthole | Coumarins | 40,99 | 484-12-8 |
| 656 | 21.91 | 297.1515 | [M+H]⁺ | C19H20O3 | 0.54 | 92.31 | 297.14,279.13,268.10,251.14,237.09,209.09 | Cryptotanshinone or its isomer | Quinones | 25 | 35825-57-1 |
| 657 | 21.92 | 301.1066 | [M+H]⁺ | C17H16O5 | 0.87 | 94.63 | 301.10,245.04,233.04,218.02,69.07 | Phellopterin | Coumarins | 86,95 | 2543-94-4 |
| 658 | 21.93 | 401.1975 | [M+H]⁺ | C23H28O6 | -2.12 | 86.53 | 401.19,386.17,371.18,331.11,300.09 | Gomisin N or its isomer | Lignans | 54 | 69176-52-9 |
| 659 | 21.95 | 301.1071 | [M+H]⁺ | C17H16O5 | -0.91 | 96.34 | 301.10,233.04,218.02,173.02,69.07 | Cnidilin or its isomer | Coumarins | 87 | 14348-22-2 |
| 660 | 21.97 | 305.2109 | [M+H]⁺ | C19H28O3 | 0.90 | 90.27 | 305.21,161.05,145.06,137.05,122.03,94.04 | 8-Shogaol | Phenolics | 60 | 36700-45-5 |
| 661 | 22.03 | 233.1515 | [M+H]⁺ | C15H20O2 | -9.06 | 83.14 | 233.15,167.08,145.09,131.08,105.06,91.05 | Alantolactone | Terpenoids | 23 | 546-43-0 |
| 662 | 22.05 | 303.2314 | [M+H]⁺ | C20H30O2 | 0.89 | 84.24 | 303.23,199.14,187.14,173.13,157.10,145.09,133.10,119.08,105.06,95.08,81.06,67.05,55.05 | Abietic acid or its isomer | Terpenoids | 97 | 514-10-3 |
| 663 | 22.13 | 191.1056 | [M+H]⁺ | C12H14O2 | -3.50 | 82.84 | 191.10,173.09,155.09,145.10,135.04,117.06,105.07,91.05,79.05,67.05,57.03 | 3-n-Butylphathlide or its isomer | Phthalides | 84 | 6066-49-5 |
| 664 | 22.17 | 233.1542 | [M+H]⁺ | C15H20O2 | 3.00 | 90.41 | 233.15,215.14,197.13,187.14,177.09,151.07,133.06,95.08 | Atractylenolide II or its isomer | Terpenoids | 77,94 | 73069-14-4 |
| 665 | 22.18 | 329.1388 | [M+H]⁺ | C19H20O5 | -2.10 | 94.61 | 329.13,247.09,229.08,214.06,83.04 | Decursin | Coumarins | 86 | 5928-25-6 |
| 666 | 22.19 | 723.4315 | [M+H]⁺ | C39H62O12 | -0.05 | 87.82 | 723.43,579.31,253.19,85.02 | Paris saponin V | Saponin | 75 | 19057-67-1 |
| 667 | 22.20 | 233.1533 | [M+H]⁺ | C15H20O2 | -0.01 | 81.00 | 233.15,167.08,157.07,145.07,131.08,117.06,105.06,91.05 | Costunolide or its isomer | Terpenoids | 86 | 553-21-9 |
| 668 | 22.26 | 301.1071 | [M+H]⁺ | C17H16O5 | 0.72 | 92.39 | 301.10,233.04,218.02,173.02,69.07 | Cnidilin or its isomer | Coumarins | 87 | 14348-22-2 |
| 669 | 22.27 | 231.1357 | [M+H]⁺ | C15H18O2 | -9.52 | 78.56 | 231.14,213.12,189.12,173.09,161.05,149.05,135.08,121.06,109.09,95.04,83.08,69.07,55.05 | Furanodienon | Terpenoids | 48,97 | 24268-41-5 |
| 670 | 22.28 | 233.1530 | [M+H]⁺ | C15H20O2 | -2.51 | 82.58 | 233.15,177.08 | Pterosin Z or its isomer | Terpenoids | 28 | 34169-69-2 |
| 671 | 22.28 | 149.0593 | [M+H]⁺ | C9H8O2 | 1.77 | 89.72 | 149.05,91.05,77.03,65.03,51.02 | 2-Jiangjirouguiquan | Phenylpropanoids | 97 | 3541-42-2 |
| 672 | 22.33 | 271.0970 | [M+H]⁺ | C16H14O4 | -6.16 | 88.78 | 271.09,203.03,175.03,147.04,131.04,69.07 | Isoimperatorin or its isomer | Coumarins | 40,52,86 | 482-45-1 |
| 673 | 22.34 | 203.0342 | [M+H]⁺ | C11H6O4 | 1.32 | 92.16 | 203.03,175.03,159.04,147.04,131.04,119.04,103.05,91.05,77.03,65.03 | Bergaptol or its isomer | Coumarins | 5,52,86,95 | 486-60-2 |
| 674 | 22.36 | 303.2314 | [M+H]⁺ | C20H30O2 | 0.87 | 80.59 | 303.23,199.14,187.14,173.13,157.10,145.09,133.10,119.08,105.06,95.08,81.06,67.05,55.05 | Abietic acid or its isomer | Terpenoids | 97 | 514-10-3 |
| 675 | 22.37 | 231.1371 | [M+H]⁺ | C15H18O2 | -3.50 | 89.13 | 231.13,165.06,128.06,91.05,55.05 | Dehydrocostuslactone or its isomer | Terpenoids | 97 | 477-43-0 |
| 676 | 22.39 | 325.1413 | [M+H]⁺ | C20H20O4 | -0.17 | 85.78 | 325.14,307.12,283.14,252.10 | Bavachalcone or its isomer | Chalcones | 32 | 28448-85-3 |
| 677 | 22.41 | 203.0343 | [M+H]⁺ | C11H6O4 | 1.72 | 95.48 | 203.03,185.02,175.03,159.04,147.04,131.05,119.04,103.05,91.05,77.03 | Xanthotoxol or its isomer | Coumarins | 42,87 | 2009-24-7 |
| 678 | 22.45 | 219.1742 | [M+H]⁺ | C15H22O | 1.30 | 90.96 | 219.17,201.16,177.12,163.10,151.11,135.08,123.11,109.10,97.06,81.06 | (-)-γ-Cuparenol or its isomer | Terpenoids | 25,86,94 | 1357469-94-3 |
| 679 | 22.45 | 277.1792 | [M+H]⁺ | C17H24O3 | 0.88 | 88.82 | 277.17,137.05,122.03 | 6-Shogaol or its isomer | Phenolics | 60,73 | 555-66-8 |
| 680 | 22.48 | 295.1326 | [M+H]⁺ | C19H18O3 | 2.51 | 92.54 | 295.13,227.12,249.12 | Tanshinone IIA or its isomer | Quinones | 25,32,89 | 568-72-9 |
| 681 | 22.59 | 245.1169 | [M+H]⁺ | C15H16O3 | 0.70 | 90.96 | 245.11,177.05 | Suberosin | Coumarins | 87 | 581-31-7 |
| 682 | 22.63 | 271.0962 | [M+H]⁺ | C16H14O4 | 1.01 | 94.18 | 271.09,203.03,175.03,147.04,131.04,69.07 | Isoimperatorin or its isomer | Coumarins | 95 | 482-45-1 |
| 683 | 22.71 | 271.0967 | [M+H]⁺ | C16H14O4 | 6.18 | 82.10 | 271.09,215.03,203.03,147.04,69.06 | Imperatorin or its isomer | Coumarins | 42 | 482-44-0 |
| 684 | 22.73 | 203.0340 | [M+H]⁺ | C11H6O4 | 4.74 | 87.70 | 203.03,175.03,159.04,147.04,131.05,91.05 | Xanthotoxol or its isomer | Coumarins | 95 | 2009-24-7 |
| 685 | 22.74 | 393.2073 | [M+H]⁺ | C25H28O4 | 2.87 | 94.06 | 393.20,149.02,93.02,65.03 | Xanthoangelol | Chalcones | 28,56,77,92 | 62949-76-2 |
| 686 | 22.75 | 403.2107 | [M+H]⁺ | C23H30O6 | 2.02 | 91.78 | 403.21,371.18,301.10,271.09,227.07 | Schisanhenol or its isomer | Lignans | 13,54,56 | 69363-14-0 |
| 687 | 22.77 | 403.2458 | [M+H]⁺ | C24H34O5 | 3.47 | 81.24 | 403.24,349.21,145.10 | 19-Hydroxybufalin | Steroids | 88 | 39844-86-5 |
| 688 | 22.82 | 231.1380 | [M+H]⁺ | C15H18O2 | 0.29 | 84.34 | 231.13,165.06,128.06,91.05,55.05 | Dehydrocostuslactone or its isomer | Terpenoids | 97 | 477-43-0 |
| 689 | 22.86 | 219.1742 | [M+H]⁺ | C15H22O | 0.25 | 90.06 | 219.17,156.09,143.08,135.08,129.06,91.05,79.05,67.05,55.05 | Germacrone or its isomer | Terpenoids | 25 | 6902-91-6 |
| 690 | 22.89 | 219.1734 | [M+H]⁺ | C15H22O | 6.21 | 82.09 | 219.17,201.16,177.12,163.10,151.11,135.08,123.11,109.10,97.06,81.06 | (-)-γ-Cuparenol or its isomer | Terpenoids | 94 | 1357469-94-3 |
| 691 | 22.92 | 339.1622 | [M+H]⁺ | C21H22O4 | -4.76 | 84.70 | 339.19,271.09,219.10,147.04 | Bavachinin or its isomer | Flavonoids | 89 | 19879-30-2 |
| 692 | 23.03 | 277.0857 | [M+H]⁺ | C18H12O3 | -0.09 | 92.01 | 277.08,262.06,249.09,234.06,221.09,202.07,193.10,178.07,169.06,141.06 | Tanshinone Ⅰor its isomer | Quinones | 32 | 568-73-0 |
| 693 | 23.08 | 303.2314 | [M+H]⁺ | C20H30O2 | 1.20 | 82.71 | 303.23,199.14,187.14,173.13,157.10,145.09,133.10,119.08,105.06,95.08,81.06,67.05,55.05 | Abietic acid or its isomer | Terpenoids | 97 | 514-10-3 |
| 694 | 23.12 | 471.3463 | [M+H]⁺ | C30H46O4 | 1.74 | 85.70 | 471.34,453.33,317.21,161.13 | Glycyrrhetic acid or its isomer | Terpenoids | 97 | 471-53-4 |
| 695 | 23.19 | 317.2080 | [M+H]⁺ | C20H28O3 | -5.11 | 76.67 | 317.20,123.11 | 11-Hydroxy-sugiol | Terpenoids | 62 | 88664-08-8 |
| 696 | 23.20 | 255.0650 | [M+H]⁺ | C15H10O4 | -0.80 | 93.85 | 255.06,227.07,209.05,181.06,165.06,153.06,135.04,121.02 | Chrysophanol | Anthraquinone | 3,92 | 481-74-3 |
| 697 | 23.20 | 219.1741 | [M+H]⁺ | C15H22O | 2.27 | 89.55 | 219.17,201.16,177.12,163.10,151.11,135.08,123.11,109.10,97.06,81.06 | (-)-γ-Cuparenol or its isomer | Terpenoids | 62,73 | 1357469-94-3 |
| 698 | 23.20 | 297.1481 | [M+H]⁺ | C19H20O3 | 1.11 | 96.04 | 297.14,279.13,268.10,251.14,237.09,209.09 | Cryptotanshinone or its isomer | Quinones | 23,25,26,56,69 | 35825-57-1 |
| 699 | 23.31 | 391.1907 | [M+H]⁺ | C25H26O4 | -0.39 | 96.54 | 391.19,335.12,309.11,281.08,267.06,255.06,137.02,69.07 | Corylifol A | Chalcones | 89 | 775351-88-7 |
| 700 | 23.38 | 277.0859 | [M+H]⁺ | C18H12O3 | 0.17 | 99.06 | 277.08,262.06,249.09,231.08,221.09,193.10 | Tanshinone Ⅰor its isomer | Quinones | 18,23,25,26,56,62,72,89 | 568-73-0 |
| 701 | 23.39 | 403.2107 | [M+H]⁺ | C23H30O6 | 1.70 | 93.52 | 403.21,371.18,301.10,271.09,227.07 | Schisanhenol or its isomer | Lignans | 13,25,54,56,83 | 69363-14-0 |
| 702 | 23.47 | 471.3463 | [M+H]⁺ | C30H46O4 | 1.31 | 86.98 | 471.34,453.33,317.21,161.13 | Glycyrrhetic acid or its isomer | Terpenoids | 97 | 471-53-4 |
| 703 | 23.53 | 323.1277 | [M+H]⁺ | C20H18O4 | -0.14 | 89.44 | 323.12,267.06 | 8-Prenyldaidzein or its isomer | Flavonoids | 89 | 135384-00-8 |
| 704 | 23.61 | 219.1742 | [M+H]⁺ | C15H22O | -0.60 | 83.36 | 219.17,201.16,177.12,163.10,151.11,135.08,123.11,109.10,97.06,81.06 | (-)-γ-Cuparenol or its isomer | Terpenoids | 25 | 1357469-94-3 |
| 705 | 23.63 | 369.1330 | [M+H]⁺ | C21H20O6 | 0.43 | 98.13 | 369.13,313.06 | Glycycoumarin or its isomer | Coumarins | 29 | 94805-82-0 |
| 706 | 23.64 | 219.1741 | [M+H]⁺ | C15H22O | 0.20 | 85.61 | 219.17,156.09,143.08,135.08,129.06,91.05,79.05,67.05,55.05 | Germacrone or its isomer | Terpenoids | 86 | 6902-91-6 |
| 707 | 23.74 | 277.0857 | [M+H]⁺ | C18H12O3 | 2.25 | 82.89 | 277.08,262.06,249.09,234.06,221.09,202.07,193.10,178.07,169.06,141.06 | Tanshinone Ⅰor its isomer | Quinones | 32,62 | 568-73-0 |
| 708 | 23.82 | 348.1223 | [M+H]⁺ | C21H18NO4 | 2.81 | 90.41 | 348.12,333.09,304.09 | Chelerythrine | Alkaloid | 41 | 34316-15-9 |
| 709 | 23.86 | 401.1949 | [M+H]⁺ | C23H28O6 | 1.69 | 82.86 | 401.19,386.17,371.18,331.11,300.09 | Gomisin N or its isomer | Lignans | 25 | 69176-52-9 |
| 710 | 23.95 | 323.1277 | [M+H]⁺ | C20H18O4 | 4.60 | 86.46 | 323.12,267.06 | 8-Prenyldaidzein or its isomer | Flavonoids | 89 | 135384-00-8 |
| 711 | 24.00 | 279.1014 | [M+H]⁺ | C18H14O3 | 0.28 | 89.69 | 279.10,261.09,233.09,205.10 | Dihydrotanshinone I or its isomer | Quinones | 32,89 | 87205-99-0 |
| 712 | 24.02 | 303.2314 | [M+H]⁺ | C20H30O2 | 0.75 | 82.71 | 303.23,199.14,187.14,173.13,157.10,145.09,133.10,119.08,105.06,95.08,81.06,67.05,55.05 | Abietic acid or its isomer | Terpenoids | 97 | 514-10-3 |
| 713 | 24.08 | 473.3617 | [M+H]⁺ | C30H48O4 | 0.71 | 89.62 | 473.36,215.17,203.17,189.16,163.14,149.13,133.10,119.05,95.08,81.06,69.06,57.07 | Hederagenin or its isomer | Terpenoids | 39 | 465-99-6 |
| 714 | 24.33 | 317.2091 | [M+H]⁺ | C20H28O3 | 6.47 | 79.12 | 317.20,281.19 | Cafestol or its isomer | Terpenoids | 40 | 469-83-0 |
| 715 | 24.35 | 332.0911 | [M+H]⁺ | C20H14NO4 | -5.01 | 84.09 | 332.09,317.06,274.08,218.09 | Sanguinarine | Alkaloid | 41 | 2447-54-3 |
| 716 | 24.44 | 339.1586 | [M+H]⁺ | C21H22O4 | -1.31 | 91.37 | 339.19,271.09,219.10,147.04 | Bavachinin or its isomer | Flavonoids | 89 | 19879-30-2 |
| 717 | 24.50 | 279.1592 | [M+H]⁺ | C16H22O4 | 0.72 | 91.28 | 279.15,149.02,121.02,57.07 | Dibutyl phthalate | Esters | 1,3,4,5,7,9,10,11,12,13,14,15,16,18,19,20,21,23,24,25,26,27,28,29,30,31,32,33,34,36,37,38,39,40,41,42,44,45,47,48,52,54,55,56,57,58,59,60,61,63,65,66,67,69,70,71,72,73,74,75,76,77,78,79,80,81,82,84,85,86,87,88,89,90,91,92,93,94,95,96,98,100 | 84-74-2 |
| 718 | 24.65 | 381.2056 | [M+H]⁺ | C24H28O4 | -1.34 | 95.23 | 381.20,191.10,135.04,91.05 | Levistilide A or its isomer | Phenolics | 16,23,40,55,56,86,94 | 88182-33-6 |
| 719 | 24.68 | 191.1069 | [M+H]⁺ | C12H14O2 | 1.60 | 85.11 | 191.10,173.09,155.09,145.10,135.04,117.06,105.07,91.05,79.05,67.05,57.03 | 3-n-Butylphathlide or its isomer | Phthalides | 84 | 6066-49-5 |
| 720 | 24.73 | 417.2329 | [M+H]⁺ | C24H32O6 | -2.06 | 94.30 | 417.23,402.20,386.20,371.18,355.15,347.15,316.13,301.10,270.08,242.09,212.08 | Schisandrin A | Lignans | 13,56,81,96 | 61281-38-7 |
| 721 | 24.75 | 471.3478 | [M+H]⁺ | C30H46O4 | 1.63 | 83.55 | 471.34,453.33,317.21,161.13 | Glycyrrhetic acid or its isomer | Terpenoids | 73 | 471-53-4 |
| 722 | 24.76 | 191.1063 | [M+H]⁺ | C12H14O2 | 2.85 | 90.16 | 191.10,173.09,163.11,145.10,133.06,117.07,105.06,91.05,79.05 | Z-Ligustilide or its isomer | Phthalides | 5,16,28,40,41,42,55,94 | 4431-01-0 |
| 723 | 24.81 | 154.1339 | [M+H]⁺ | C10H18O | -5.98 | 85.58 | 154.13,141.11,81.06 | Linalool | Terpenoids | 5 | 78-70-6 |
| 724 | 25.00 | 381.2056 | [M+H]⁺ | C24H28O4 | -6.09 | 90.93 | 381.20,191.10,135.04,91.05 | Levistilide A or its isomer | Phenolics | 5,13,16,23,24,40,42,55,84,86,87,94 | 88182-33-6 |
| 725 | 25.02 | 336.1215 | [M+H]⁺ | C20H18NO4 | 4.91 | 89.12 | 336.12,320.09 | Epiberberine or its isomer | Alkaloid | 45 | 6873-09-2 |
| 726 | 25.03 | 295.1328 | [M+H]⁺ | C19H18O3 | 0.99 | 95.49 | 295.13,227.12,249.12 | Tanshinone IIA or its isomer | Quinones | 19,23,25,26,56,62,71 | 568-72-9 |
| 727 | 25.06 | 191.1071 | [M+H]⁺ | C12H14O2 | 2.79 | 90.51 | 191.10,173.09,163.11,145.10,133.06,117.07,105.06,91.05,79.05 | Z-Ligustilide or its isomer | Phthalides | 5,28,40,41,42,47,55,94 | 4431-01-0 |
| 728 | 25.06 | 381.2082 | [M+H]⁺ | C24H28O4 | -1.92 | 91.30 | 381.21,191.10,89.05 | Senkyunolide O | Phthalides | 29,41 | 142797-35-1 |
| 729 | 25.13 | 203.0321 | [M+H]⁺ | C11H6O4 | -6.65 | 78.91 | 203.03,175.03,159.04,147.04,131.05,91.05 | Xanthotoxol or its isomer | Coumarins | 5,52 | 2009-24-7 |
| 730 | 25.21 | 279.1601 | [M+H]⁺ | C16H22O4 | 2.65 | 80.32 | 279.15,149.02,121.02,57.07 | Dibutyl phthalate | Esters | 19,23,45,52,57,62,80,85,93,99 | 84-74-2 |
| 731 | 25.38 | 401.1956 | [M+H]⁺ | C23H28O6 | 0.04 | 96.69 | 401.19,386.17,371.18,331.11,300.09 | Gomisin N or its isomer | Lignans | 13,24,54,56,81,96 | 69176-52-9 |
| 732 | 25.49 | 485.2524 | [M+H]⁺ | C28H36O7 | 2.35 | 90.94 | 485.25,403.21,83.04 | Negsehisandrin G | Lignans | 13,54 | 1023744-69-5 |
| 733 | 25.51 | 423.1781 | [M+H]⁺ | C25H26O6 | 4.82 | 80.59 | 423.17,367.14 | Kuwanon C | Flavonoids | 56 | 62949-79-5 |
| 734 | 25.59 | 401.1961 | [M+H]⁺ | C23H28O6 | -3.21 | 91.03 | 401.19,386.17,371.18,331.11 | Schisandrin B or its isomer | Lignans | 88 | 61281-37-6 |
| 735 | 25.59 | 191.1065 | [M+H]⁺ | C12H14O2 | -0.83 | 89.83 | 191.10,173.09,163.11,145.10,133.06,117.07,105.06,91.05,79.05 | Z-Ligustilide or its isomer | Phthalides | 16,55 | 4431-01-0 |
| 736 | 25.59 | 336.1272 | [M+H]⁺ | C20H18NO4 | -5.92 | 77.44 | 336.12,320.09 | Epiberberine or its isomer | Alkaloid | 20 | 6873-09-2 |
| 737 | 25.74 | 381.2056 | [M+H]⁺ | C24H28O4 | 0.70 | 93.85 | 381.20,191.10,135.04,91.05 | Levistilide A or its isomer | Phenolics | 55 | 88182-33-6 |
| 738 | 25.81 | 471.3502 | [M+H]⁺ | C30H46O4 | -0.29 | 99.14 | 471.34,453.33,435.32,407.32,333.20,287.23,265.14,247.13,219.14,173.13,149.09,121.10,95.08,81.06,69.07 | 11-Keto-beta-boswellic acid or its isomer | Terpenoids | 70,84,86,97 | 17019-92-0 |
| 739 | 25.85 | 417.1797 | [M+H]⁺ | C23H28O7 | -5.65 | 78.95 | 417.17,100.10 | Epigomisin O | Lignans | 24 | 73036-31-4 |
| 740 | 25.90 | 485.2535 | [M+H]⁺ | C28H36O7 | 0.44 | 96.32 | 485.25,403.21,83.04 | Negsehisandrin G | Lignans | 13,54,56,83 | 1023744-69-5 |
| 741 | 25.95 | 385.1651 | [M+H]⁺ | C22H24O6 | -1.30 | 94.23 | 385.16,355.15,285.07 | Schisandrin C or its isomer | Lignans | 13,54,56 | 61301-33-5 |
| 742 | 26.14 | 203.0332 | [M+H]⁺ | C11H6O4 | -2.46 | 93.37 | 203.03,175.03,159.04,147.04,131.05,91.05 | Xanthotoxol or its isomer | Coumarins | 5 | 2009-24-7 |
| 743 | 26.41 | 529.3515 | [M+H]⁺ | C32H48O6 | 1.95 | 89.61 | 529.35,511.34,469.33,451.31,405.31 | 23-Acetyl alisol C or its isomer | Terpenoids | 97 | 26575-93-9 |
| 744 | 26.43 | 287.2002 | [M+H]⁺ | C19H26O2 | 0.64 | 91.52 | 287.20,269.10,109.06.97.06 | Androstenedione or its isomer | Steroids | 32 | 63-05-8 |
| 745 | 26.44 | 381.2056 | [M+H]⁺ | C24H28O4 | 1.09 | 91.73 | 381.20,191.10,135.04,91.05 | Levistilide A or its isomer | Phenolics | 40 | 88182-33-6 |
| 746 | 26.46 | 303.2314 | [M+H]⁺ | C20H30O2 | 1.13 | 82.17 | 303.23,197.12,185.12,173.13,159.11,145.09,133.10,119.08,105.06,93.06,81.06,67.05,55.05 | Abietic acid or its isomer | Terpenoids | 70,97 | 514-10-3 |
| 747 | 26.61 | 333.2420 | [M+H]⁺ | C21H32O3 | -3.62 | 95.50 | 333.24,137.05 | 10-Shogaol | Phenolics | 60 | 36752-54-2 |
| 748 | 26.63 | 319.2254 | [M+H]⁺ | C20H30O3 | -5.25 | 83.94 | 319.22,165.12 | Steviol Hydroxydehydrostevic acid or its isomer | Terpenoids | 99 | 471-80-7 |
| 749 | 26.78 | 457.3665 | [M+H]⁺ | C30H48O3 | -6.17 | 88.51 | 457.36,393.35,231.21,203.17,189.16,175.14,147.11,121.10,95.08,69.07,55.05 | Epibetulinic acid | Terpenoids | 39 | 38736-77-5 |
| 750 | 26.83 | 515.3727 | [M+H]⁺ | C32H50O5 | 0.57 | 89.46 | 515.37,497.36,479.35,437.34,383.29,357.27,313.25,219.17,151.11,97.06 | Alisol B 23-acetate | Terpenoids | 16 | 26575-95-1 |
| 751 | 26.94 | 233.1537 | [M+H]⁺ | C15H18O2 | -4.75 | 86.19 | 233.15,215.14,197.13,187.14,177.09,151.07,133.06,95.08 | Atractylenolide II or its isomer | Terpenoids | 94 | 73069-14-4 |
| 752 | 26.96 | 297.1485 | [M+H]⁺ | C19H20O3 | -1.52 | 87.64 | 297.14,279.13,268.10,251.14,237.09,209.09 | Cryptotanshinone or its isomer | Quinones | 32 | 35825-57-1 |
| 753 | 27.05 | 287.2002 | [M+H]⁺ | C19H26O2 | 2.26 | 90.15 | 287.20,269.10,109.06.97.06 | Androstenedione or its isomer | Steroids | 32 | 63-05-8 |
| 754 | 27.13 | 303.2305 | [M+H]⁺ | C20H30O2 | -4.10 | 89.64 | 303.23,199.14,187.14,173.13,159.11,145.10,133.10,121.10,107.08,93.06,81.06,67.05,55.05 | Abietic acid or its isomer | Terpenoids | 99 | 514-10-3 |
| 755 | 27.14 | 471.3463 | [M+H]⁺ | C30H46O4 | 0.99 | 84.74 | 471.34,453.33,435.32,407.32,333.20,269.21,215.17,173.13,145.10,121.10,107.08,95.08,81.06,69.07 | 11-Keto-beta-boswellic acid or its isomer | Terpenoids | 97 | 17019-92-0 |
| 756 | 27.14 | 471.3466 | [M+H]⁺ | C30H46O4 | -0.01 | 82.50 | 471.34,453.33,317.21,161.13 | Glycyrrhetic acid or its isomer | Terpenoids | 70 | 471-53-4 |
| 757 | 27.27 | 319.2262 | [M+H]⁺ | C20H30O3 | -0.74 | 83.64 | 319.22,165.12 | Steviol Hydroxydehydrostevic acid or its isomer | Terpenoids | 99 | 471-80-7 |
| 758 | 27.48 | 295.1332 | [M+H]⁺ | C19H18O3 | 2.01 | 82.88 | 295.13,227.12,249.12 | Tanshinone IIA or its isomer | Quinones | 32 | 568-72-9 |
| 759 | 27.55 | 297.1485 | [M+H]⁺ | C19H20O3 | 0.63 | 81.93 | 297.14,279.13,268.10,251.14,237.09,209.09 | Cryptotanshinone or its isomer | Quinones | 32 | 35825-57-1 |
| 760 | 27.80 | 473.3517 | [M+H]⁺ | C30H48O4 | -6.02 | 76.69 | 473.36,215.17,203.17,189.16,163.14,149.13,133.10,119.05,95.08,81.06,69.06,57.07 | Hederagenin or its isomer | Terpenoids | 84 | 465-99-6 |
| 761 | 27.83 | 287.2002 | [M+H]⁺ | C19H26O2 | 0.99 | 87.52 | 287.20,269.10,109.06.97.06 | Androstenedione or its isomer | Steroids | 32 | 63-05-8 |

ESI+: Electrospray Ionization Positive Mode

# Table S3 673 chemical components characterized in 100 Traditional Chinese Medicine prescriptions (ESI-)

| No. | RT(min) | | Mass(ppm) | Precursors/Adduction | | Formula | | Error(ppm) | | Score | Product ion | Identification | Class | Source | CAS |
| --- | --- | --- | --- | --- | --- | --- | --- | --- | --- | --- | --- | --- | --- | --- | --- |
| 1 | 1.02 | | 181.0792 | [M-H]- | | C_6_H_14_O_6_ | | 1.11 | | 94.29 | 181.07,113.02,101.02,89.02,71.01,59.01 | Mannitol | Polyhydric alcohols | 21,36,42,54,57,64,65,69,87,88, | 69-65-8 |
| 2 | 1.02 | | 179.0634 | [M-H]- | | C_6_H_12_O_6_ | | -0.17 | | 93.15 | 179.05,119.03,101.02,89.02,71.01,59.01 | D-Mannose | Carbohydrate | 12,36,42,85,90 | 3458-28-4 |
| 3 | 1.02 | | 173.1117 | [M-H]- | | C_6_H_14_N_4_O_2_ | | -0.29 | | 92.52 | 173.11,131.08 | L(+)-Arginine | Amino acid | 19,23,34,37,40,41,48,52,65,70,73,75,84,87,93,98 | 74-79-3 |
| 4 | 1.02 | | 179.0638 | [M-H]- | | C_6_H_12_O_6_ | | 2.54 | | 96.08 | 179.06,113.02,101.02,89.02,71.01,59.01 | Glucose | Carbohydrate | 24,44,60,91 | 50-99-7 |
| 5 | 1.02 | | 179.0627 | [M-H]- | | C_6_H_12_O_6_ | | 3.59 | | 89.00 | 179.05,71.01,59.01 | D(-)-Fructose | Carbohydrate | 94 | 57-48-7 |
| 6 | 1.04 | | 181.0802 | [M-H]- | | C_6_H_14_O_6_ | | 3.93 | | 91.15 | 181.07,119.03,101.02,59.01 | D-Sorbitol | Polyhydric alcohols | 5,20,34,47,68,79 | 50-70-4 |
| 7 | 1.08 | | 209.0380 | [M-H]- | | C6H10O8 | | -2.15 | | 91.47 | 209.03,133.01,85.02 | Galactaric Acid | Organic acid | 19,53,83 | 526-99-8 |
| 8 | 1.13 | | 387.1159 | [M+HCOO]- | | C_12_H_22_O_11_ | | -0.99 | | 96.73 | 341.11,179.05,161.04,143.03,119.03,101.02,89.02,71.01,59.01 | Sucrose or its isomer | Carbohydrate | 2,6,12,23,24,26,27,31,32,33,34,35,36,37,38,40,45,55,58,59,66,67,69,73,75,76,81,82,84,85,88,90,93,94,99 | 57-50-1 |
| 9 | 1.14 | | 149.0165 | [M-H]- | | C_4_H_6_O_6_ | | -0.24 | | 92.96 | 149.01,131.00,103.00,87.00,73.00,59.01 | DL-Tartaric acid or its isomer | Organic acid | 30,49 | 133-37-9 |
| 10 | 1.15 | | 191.0629 | [M-H]- | | C7H12O6 | | 2.67 | | 93.08 | 191.06,173.04,127.03,87.03,59.01 | D-(-)-Quinic acid or its isomer | Phenolics | 7,8,29,33,73,83,91,95,98 | 36413-60-2 |
| 11 | 1.25 | | 133.0216 | [M-H]- | | C_4_H_6_O_5_ | | -0.52 | | 96.43 | 133.01,115.00,89.02,71.01,59.01 | Malic acid or its isomer | Organic acid | 1,3,5,6,7,8,9,10,11,16,19,20,21,22,23,25,26,28,29,30,35,36,37,38,41,42,43,44,45,46,47,50,51,52,53,54,55,56,57,61,62,64,65,67,68,69,71,70,72,73,75,76,77,79,80,83,84,86,88,91,94,95,98,100 | 6915-15-7 |
| 12 | 1.32 | | 115.0110 | [M-H]- | | C_4_H_4_O_4_ | | -0.26 | | 95.71 | 115.01,71.01 | Fumaric acid or its isomer | Organic acid | 13,16,30,54,73,83 | 110-17-8 |
| 13 | 1.34 | | 191.0268 | [M-H]- | | C_6_H_8_O_7_ | | -1.01 | | 95.30 | 191.01,173.00,129.01,111.00 | Citric acid or its isomer | Organic acid | 21,23,34,48,52,55,60,65,72,73,75,76,81,84,85,91,95,98 | 77-92-9 |
| 14 | 1.35 | | 275.0285 | [M-H]- | | C_13_H_8_O_7_ | | 5.46 | | 80.20 | 275.02,201.01 | 3,4,8,9,10-Pentahydroxy Urolithin or its isomer | Phenolics | 25 | 91485-02-8 |
| 15 | 1.37 | | 701.2236 | [M+HCOO]- | | C_24_H_42_O_21_ | | -2.58 | | 92.89 | 665.22,485.15,341.10 | Nystose | Carbohydrate | 5,19,26,32,56,77 | 13133-07-8 |
| 16 | 1.39 | | 115.0108 | [M-H]- | | C_4_H_4_O_4_ | | 1.50 | | 95.76 | 115.00,71.01 | Maleic acid | Organic acid | 29,43,50,64,83 | 110-16-7 |
| 17 | 1.51 | | 133.0220 | [M-H]- | | C_4_H_6_O_5_ | | 3.27 | | 73.96 | 133.01,115.00,89.02,71.01,59.01 | Malic acid or its isomer | Organic acid | 5,9,13,20,25,28,41,49,54,57,61,64,68,71,79,91, | 6915-15-7 |
| 18 | 1.53 | | 387.1159 | [M+HCOO]- | | C_12_H_22_O_11_ | | -0.94 | | 97.92 | 341.11,179.05,161.04,143.03,119.03,101.02,89.02,71.01,59.01 | Sucrose or its isomer | Carbohydrate | 12,15,33,37,85,87,90, | 57-50-1 |
| 19 | 1.55 | | 503.1690 | [M+HCOO]- | | C_18_H_32_O_16_ | | 0.78 | | 81.18 | 503.16,341.09,179.05,89.02 | 1-Kestose | Carbohydrate | 25 | 470-69-9 |
| 20 | 1.57 | | 191.0265 | [M-H]- | | C_6_H_8_O_7_ | | -2.39 | | 95.16 | 191.01,173.00,129.01,111.00 | Citric acid or its isomer | Organic acid | 4,17,21,29,30,34,35,36,37,38,40,42,44,47,50,51,52,53,58,61,62,63,64,65,68,69,70,71,72,73,76,82,83,84,85,87,88,90,91,93,98,100 | 77-92-9 |
| 21 | 1.84 | | 191.0269 | [M-H]- | | C_6_H_8_O_7_ | | 0.28 | | 97.60 | 191.01,173.00,129.01,111.00 | Citric acid or its isomer | Organic acid | 1,2,3,4,5,6,7,8,9,10,11,15,16,17,19,21,20,22,23,25,28,29,30,35,38,41,42,43,44,45,46,47,48,49,50,51,53,54,55,56,58,60,61,62,63,64,65,68,70,71,72,73,75,77,80,81,83,85,86,91,92,93,94,95,97,98,100 | 77-92-9 |
| 22 | 2.01 | | 115.0103 | [M-H]- | | C_4_H_4_O_4_ | | -5.98 | | 93.06 | 115.01,71.01 | Fumaric acid or its isomer | Organic acid | 16,48 | 110-17-8 |
| 23 | 2.04 | | 387.1159 | [M+HCOO]- | | C_12_H_22_O_11_ | | -1.17 | | 89.66 | 341.11,179.05,161.04,143.03,119.03,101.02,89.02,71.01,59.01 | Sucrose or its isomer | Carbohydrate | 12,69,82,85,88,90 | 57-50-1 |
| 24 | 2.09 | | 117.0266 | [M-H]- | | C_4_H_6_O_4_ | | -0.10 | | 92.13 | 117.02,99.00,73.02 | Succinic acid | Organic acid | 8,10,13,15,16,17,19,21,23,25,29,30,37,42,43,48,52,54,55,56,57,58,59,64,65,66,69,70,72,75,80,81,84,85,86,88,90,93,98,100 | 110-15-6 |
| 25 | 2.11 | | 173.0160 | [M-H]- | | C_6_H_6_O_6_ | | 2.56 | | 95.03 | 173.01,129.01,113.00,85.02 | Trans-Aconitic acid | Organic acid | 75 | 4023-65-8 |
| 26 | 2.16 | | 151.0332 | [M-H]- | | C_5_H_4_N_4_O_2_ | | 1.52 | | 87.70 | 151.03,109.01 | 2,6-Dihydroxypurine | Alkaloid | 66,70,84 | 69-89-6 |
| 27 | 2.29 | | 243.0691 | [M-H]- | | C9H12N2O6 | | 1.61 | | 85.12 | 243.06,151.03,130.03,109.02,81.02 | Uridine | Alkaloid | 13,19,21,29,48,52,55,57,58,64,67,73,76,80,81,83,95 | 58-96-8 |
| 28 | 2.35 | | 387.1159 | [M+HCOO]- | | C12H22O11 | | 0.41 | | 93.74 | 341.11,179.05,161.04,143.03,119.03,101.02,89.02,71.01,59.01 | Sucrose or its isomer | Carbohydrate | 12,69,85,88 | 57-50-1 |
| 29 | 2.35 | | 191.0267 | [M-H]- | | C6H8O7 | | 1.65 | | 77.34 | 191.01,173.00,129.01,111.00 | Citric acid or its isomer | Organic acid | 13,36,47,52,55,60,61,71,73,77,79,81,83 | 77-92-9 |
| 30 | 2.75 | | 827.2774 | [M-H]- | | C_30_H_52_O_26_ | | 3.56 | | 89.33 | 827.27,647.20,557.17,485.15,395.11,323.09,189.05 | 1F-fructofuranosylnystose | Carbohydrate | 58,86 | 59432-60-9 |
| 31 | 2.76 | | 421.1358 | [M+HCOO]- | | C_16_H_24_O_10_ | | -3.06 | | 92.70 | 375.13,213.07,169.08,113.02,89.02 | 8-Epiloganicacid or its isomer | Iridoids | 16,25,79 | 82509-41-9 |
| 32 | 3.22 | | 167.0423 | [M-H]- | | C_8_H_8_O_4_ | | 0.02 | | 88.73 | 167.04,123.02,93.02,67.04 | Isovanillic acid or its isomer | Phenolics | 11 | 645-08-9 |
| 33 | 3.30 | | 133.0218 | [M-H]- | | C4H6O5 | | -2.37 | | 84.31 | 133.01,115.00,89.02,71.01,59.01 | Malic acid or its isomer | Organic acid | 83 | 6915-15-7 |
| 34 | 3.52 | | 169.0214 | [M-H]- | | C_7_H_6_O_5_ | | -0.67 | | 90.46 | 169.02,125.02,107.01,81.03,69.03,53.03 | Gallic acid or its isomer | Phenolics | 2,8,9,12,16,23,30,35,41,42,44,46,55,61,63,68,69,70,72,81,82,86,88,90,91,93,95,98 | 149-91-7 |
| 35 | 3.54 | | 312.0960 | [M+HCOO]- | | C_10_H_13_N_5_O_4_ | | -2.86 | | 86.83 | 267.09,133.04 | Adenosine or its isomer | Alkaloid | 67,74 | 58-61-7 |
| 36 | 3.82 | | 169.0209 | [M-H]- | | C_7_H_6_O_5_ | | -3.41 | | 90.47 | 169.02,125.02,107.01,81.03,69.03,53.03 | Gallic acid or its isomer | Phenolics | 3,13,25,28,35,43,55,59,61,63,70,72,79,82,92,98 | 149-91-7 |
| 37 | 3.85 | | 191.0630 | [M-H]- | | C_7_H_12_O_6_ | | 2.14 | | 82.28 | 191.06,173.04,127.03,87.03,59.01 | D-(-)-Quinic acid or its isomer | Phenolics | 33,55,72,80 | 36413-60-2 |
| 38 | 3.87 | | 121.0358 | [M-H]- | | C_7_H_6_O_2_ | | -7.90 | | 87.00 | 121.03,93.02,65.04 | p-Hydroxybenzaldehyde or its isomer | Phenolics | 65 | 123-08-0 |
| 39 | 4.03 | | 312.0970 | [M+HCOO]- | | C10H13N5O4 | | -1.00 | | 94.47 | 267.09,133.04 | Adenosine or its isomer | Alkaloid | 17,19,43,48,65,67,70,73,83,95 | 58-61-7 |
| 40 | 4.19 | | 282.0935 | [M-H]- | | C_10_H_13_N_5_O_5_ | | -6.44 | | 88.21 | 282.09,150.04,133.01,109.02 | Crotonoside or its isomer | Alkaloid | 21,52,55,57,64,67, | 1818-71-9 |
| 41 | 4.51 | | 282.0929 | [M-H]- | | C_10_H_13_N_5_O_5_ | | -4.18 | | 90.82 | 283.08,151.04,134.01 | Guanosine or its isomer | Alkaloid | 21,51,55,57,61,64,67,74,81 | 118-00-3 |
| 42 | 4.62 | | 267.0805 | [M-H]- | | C_10_H_12_N_4_O_5_ | | -0.98 | | 91.48 | 267.08,135.02 | Inosine or its isomer | Alkaloid | 49,50,65,67,70,81,84,92 | 58-63-9 |
| 43 | 4.62 | | 167.0418 | [M-H]- | | C_8_H_8_O_4_ | | -2.44 | | 87.49 | 167.04,123.02,93.02,67.04 | Isovanillic acid or its isomer | Phenolics | 7,9 | 645-08-9 |
| 44 | 4.68 | | 421.1336 | [M+HCOO]- | | C_16_H_24_O_10_ | | 1.98 | | 92.48 | 375.13,213.07,169.08,113.02,89.02 | 8-Epiloganicacid or its isomer | Iridoids | 25 | 82509-41-9 |
| 45 | 5.42 | | 282.0921 | [M-H]- | | C10H13N5O5 | | -1.69 | | 94.13 | 283.08,151.04,134.01 | Guanosine or its isomer | Alkaloid | 4,5,17,20,23,32,37,40,41,49,50,51,58,67,70,72,73,75,76,77,83,84,91,93,95 | 118-00-3 |
| 46 | 5.44 | | 282.0913 | [M-H]- | | C_10_H_13_N_5_O_5_ | | -1.26 | | 88.84 | 282.09,150.04,133.01,109.02 | Crotonoside or its isomer | Alkaloid | 19,21 | 1818-71-9 |
| 47 | 5.53 | | 267.0795 | [M-H]- | | C_10_H_12_N_4_O_5_ | | -4.71 | | 81.21 | 267.08,135.02 | Inosine or its isomer | Alkaloid | 52,70 | 58-63-9 |
| 48 | 5.55 | | 1151.3845 | [M-H]- | | C_42_H_72_O_36_ | | -3.21 | | 80.61 | 1151.38,971.30,809.25,665.21,503.16 | Fructo-oligosaccharide DP7/GF6 | Carbohydrate | 53 | 62512-20-3 |
| 49 | 5.84 | | 515.1338 | [M-H]- | | C_25_H_24_O_12_ | | 0.98 | | 80.74 | 515.13,353.08,173.04 | Isochlorogenic acid B or its isomer | Phenolics | 81 | 14534-61-3 |
| 50 | 5.92 | | 989.3313 | [M-H]- | | C_36_H_62_O_31_ | | 2.08 | | 84.79 | 989.33,809.25 | 1,1,1,1-Kestohexaose | Carbohydrate | 58 | 62512-19-0 |
| 51 | 6.47 | | 197.0537 | [M-H]- | | C_9_H_10_O_5_ | | -4.59 | | 96.39 | 197.05,179.03,151.04,135.04,123.04,109.02 | Salvianic acid A or its isomer | Phenolics | 2,13,15,23,26,60,72 | 76822-21-4 |
| 52 | 6.58 | | 137.0315 | [M-H]- | | C_7_H_6_O_3_ | | -1.48 | | 95.26 | 137.02,93.03 | 4-Hydroxybenzoic acid or its isomer | Phenolics | 68 | 99-67-7 |
| 53 | 6.62 | | 153.0270 | [M-H]- | | C_7_H_6_O_4_ | | -2.57 | | 93.43 | 153.02,109.02,91.01,65.03 | Protocatechuic acid or its isomer | Organic acid | 12,13,15,16,29,34,42,44,48,55,60,67,70,73,74,79,85,91,95,98 | 99-50-3 |
| 54 | 6.70 | | 283.0766 | [M-H]- | | C_10_H_12_N_4_O_6_ | | -3.14 | | 94.02 | 283.07,151.02 | Xanthosine | Alkaloid | 49,52,65,81 | 146-80-5 |
| 55 | 6.72 | | 167.0418 | [M-H]- | | C_8_H_8_O_4_ | | -2.76 | | 87.23 | 167.04,123.02,93.02,67.04 | Isovanillic acid or its isomer | Phenolics | 70 | 645-08-9 |
| 56 | 6.83 | | 109.0361 | [M-H]- | | C_6_H_6_O_2_ | | 6.36 | | 79.81 | 109.03,91.01,65.00,53.03 | Catechol or its isomer | Phenolics | 9,12,29,42,44,50,55,67,68,76,79,90,91,93,98,100 | 120-80-9 |
| 57 | 6.99 | | 153.0265 | [M-H]- | | C_7_H_6_O_4_ | | 0.73 | | 92.54 | 153.02,109.02,81.03 | 2,5-dihydroxybenzoic acid or its isomer | Phenolics | 5,8,9,10,11,20,25,30,35,41,49,52,54,58,59,81,83,86,90,93,100 | 490-79-9 |
| 58 | 7.01 | | 179.0424 | [M-H]- | | C_9_H_8_O_4_ | | 0.94 | | 87.59 | 179.03,135.04,107.04,91.05 | Caffeic acid or its isomer | Phenolics | 71,72 | 331-39-5 |
| 59 | 7.03 | | 153.0266 | [M-H]- | | C_7_H_6_O_4_ | | -0.04 | | 92.93 | 153.02,109.02,91.01,65.03 | Protocatechuic acid or its isomer | Organic acid | 7,47,50,51,53,61,62,68 | 99-50-3 |
| 60 | 7.05 | | 197.0527 | [M-H]- | | C_9_H_10_O_5_ | | 0.39 | | 97.04 | 197.05,179.03,151.04,135.04,123.04,109.02 | Salvianic acid A or its isomer | Phenolics | 19,20,25,28,41,43,46,52,56,61,62,69,71,72,88 | 76822-21-4 |
| 61 | 7.14 | | 353.0956 | [M-H]- | | C_16_H_18_O_9_ | | -1.53 | | 98.33 | 353.09,161.02 | Chlorogenic Acid or its isomer | Phenolics | 15,95 | 327-97-9 |
| 62 | 7.45 | | 387.1211 | [M-H]- | | C_16_H_22_O_11_ | | -4.98 | | 85.23 | 389.10,345.11,209.04,165.05,121.06,89.02 | Secoxyloganic acid or its isomer | Iridoids | 7,51 | 59472-23-0 |
| 63 | 7.46 | | 375.1367 | [M-H]- | | C_16_H_24_O_10_ | | 6.20 | | 76.39 | 375.13,213.07,169.08,113.02,89.02 | 8-Epiloganicacid or its isomer | Iridoids | 47 | 82509-41-9 |
| 64 | 7.48 | | 137.0316 | [M-H]- | | C_7_H_6_O_3_ | | 0.94 | | 97.74 | 137.03,109.02,93.02,81.03 | Protocatechuic aldehyde | Phenolics | 13,15 | 139-85-5 |
| 65 | 7.58 | | 331.1096 | [M-H]- | | C_14_H_20_O_9_ | | 3.45 | | 80.67 | 331.10,153.01,137.99,125.02,109.00,81.00 | Leonuriside A | Phenolics | 55 | 121748-12-7 |
| 66 | 7.63 | | 353.0956 | [M-H]- | | C_16_H_18_O_9_ | | -1.18 | | 92.39 | 353.09,191.05,179.03,161.02,135.04,85.02 | 1-O-Caffeoylquinic acid or its isomer | Phenolics | 5,13,15,48,52,61 | 1241-87-8 |
| 67 | 7.79 | | 191.0630 | [M-H]- | | C_7_H_12_O_6_ | | 2.06 | | 81.17 | 191.06,173.04,127.03,87.03,59.01 | D-(-)-Quinic acid or its isomer | Phenolics | 13,44 | 36413-60-2 |
| 68 | 7.92 | | 137.0320 | [M-H]- | | C_7_H_6_O_3_ | | -2.17 | | 92.54 | 137.02,93.03 | 4-Hydroxybenzoic acid or its isomer | Phenolics | 30,81 | 99-67-7 |
| 69 | 7.95 | | 373.1207 | [M-H]- | | C_16_H_22_O_10_ | | 1.50 | | 94.93 | 373.11,211.06,165.07,149.06,123.04,89.02,71.01 | Geniposidic acid | Iridoids | 29,42 | 27741-01-1 |
| 70 | 8.09 | | 177.0270 | [M-H]- | | C_9_H_6_O_4_ | | -1.99 | | 79.20 | 177.02,133.02,105.03 | Daphnetin or its isomer | Coumarins | 15 | 486-35-1 |
| 71 | 8.12 | | 353.0962 | [M-H]- | | C_16_H_18_O_9_ | | -3.06 | | 87.81 | 353.09,161.02 | Chlorogenic Acid or its isomer | Phenolics | 58,85,90 | 327-97-9 |
| 72 | 8.15 | | 179.0425 | [M-H]- | | C_9_H_8_O_4_ | | -1.20 | | 92.70 | 179.03,135.04,107.04,91.05 | Caffeic acid or its isomer | Phenolics | 15,49,50,52 | 331-39-5 |
| 73 | 8.25 | | 149.0168 | [M-H]- | | C_4_H_6_O_6_ | | -2.30 | | 93.87 | 149.01,131.00,103.00,87.00,73.00,59.01 | DL-Tartaric acid or its isomer | Organic acid | 49,50,52 | 133-37-9 |
| 74 | 8.32 | | 121.0372 | [M-H]- | | C_7_H_6_O_2_ | | -3.48 | | 91.49 | 121.03,93.02,65.04 | p-Hydroxybenzaldehyde or its isomer | Phenolics | 13 | 123-08-0 |
| 75 | 8.35 | | 203.0906 | [M-H]- | | C_11_H_12_N_2_O_2_ | | 3.65 | | 96.51 | 203.09,185.05,159.09,141.06,115.05,73.02 | L-Tryptophan | Amino acid | 4,21,23,41,48,61,70,74,84,87,93, | 73-22-3 |
| 76 | 8.37 | | 375.1379 | [M-H]- | | C_16_H_24_O_10_ | | -2.61 | | 88.83 | 375.13,213.07,169.08,113.02,89.02 | 8-Epiloganicacid or its isomer | Iridoids | 58 | 82509-41-9 |
| 77 | 8.39 | | 137.0315 | [M-H]- | | C_7_H_6_O_3_ | | 1.34 | | 90.85 | 137.03,109.02,93.02,81.03 | Protocatechuic aldehyde or its isomer | Phenolics | 6,10,12,16,19,22,23,26,29,30,32,33,35,36,42,43,45,52,54,55,56,58,59,64,65,69,70,72,73,74,75,76,81,83,84,86,87,88,90,92,93,95,97,98,100 | 139-85-5 |
| 78 | 8.45 | | 353.0958 | [M-H]- | | C_16_H_18_O_9_ | | 1.92 | | 97.39 | 353.09,191.05,179.03,161.02,135.04,85.02 | 1-O-Caffeoylquinic acid or its isomer | Phenolics | 48,60,98 | 1241-87-8 |
| 79 | 8.45 | | 353.0935 | [M-H]- | | C_16_H_18_O_9_ | | 4.37 | | 95.86 | 353.09,161.02 | Chlorogenic Acid or its isomer | Phenolics | 12,23,29,33,36,42,52,55,58,69,73,83,85,88,90,93,95 | 327-97-9 |
| 80 | 8.52 | | 137.0313 | [M-H]- | | C_7_H_6_O_3_ | | -2.94 | | 91.02 | 137.02,93.03 | 4-Hydroxybenzoic acid or its isomer | Phenolics | 5,9,28,41,50,60,61,62,63,68,80 | 99-67-7 |
| 81 | 8.56 | | 191.0632 | [M-H]- | | C7H12O6 | | 1.24 | | 79.61 | 191.06,173.04,127.03,87.03,59.01 | D-(-)-Quinic acid or its isomer | Phenolics | 95 | 36413-60-2 |
| 82 | 8.60 | | 179.0440 | [M-H]- | | C_9_H_8_O_4_ | | -9.57 | | 77.32 | 179.03,135.04,107.04,91.05 | Caffeic acid or its isomer | Phenolics | 13,44 | 331-39-5 |
| 83 | 8.62 | | 311.0504 | [M-H]- | | C_13_H_12_O_9_ | | -5.63 | | 71.91 | 311.05,135.05,87.01 | Caftaric acid | Organic acid | 49 | 67879-58-7 |
| 84 | 8.72 | | 137.0317 | [M-H]- | | C_7_H_6_O_3_ | | 0.21 | | 90.78 | 137.03,109.02,93.02,81.03 | Protocatechuic aldehyde or its isomer | Phenolics | 26 | 139-85-5 |
| 85 | 8.77 | | 375.1363 | [M-H]- | | C_16_H_24_O_10_ | | 1.83 | | 91.06 | 375.13,213.07,169.08,113.02,89.02 | 8-Epiloganicacid or its isomer | Iridoids | 30,42,47,92 | 82509-41-9 |
| 86 | 8.80 | | 177.0631 | [M-H]- | | C_10_H_10_O_3_ | | -0.74 | | 82.36 | 177.06,151.03,135.04 | 3,4-Dihydroxybenzalacetone or its isomer | Phenolics | 13 | 37079-84-8 |
| 87 | 8.81 | | 137.0311 | [M-H]- | | C_7_H_6_O_3_ | | 4.02 | | 90.28 | 137.02,93.03 | 4-Hydroxybenzoic acid or its isomer | Phenolics | 2,12,23,25,31,35,54,66,67,70,72,73,82,83,84,90,98, | 99-67-7 |
| 88 | 8.82 | | 325.0931 | [M-H]- | | C_15_H_18_O_8_ | | 4.86 | | 96.12 | 325.09,119.05 | (Z)-p-Coumaric acid 4-O-β-D-glucopyranside or its isomer | Coumarins | 31,62.68 | 117405-48-8 |
| 89 | 8.84 | | 137.0317 | [M-H]- | | C_7_H_6_O_3_ | | 0.09 | | 92.89 | 137.03,93.03 | Salicylic Acid or its isomer | Organic acid | 3,30,55,69,76,81,88,93 | 69-72-7 |
| 90 | 8.84 | | 135.0530 | [M-H]- | | C_8_H_8_O_2_ | | -4.37 | | 84.66 | 135.04,121.02 | 4’-Hydroxyacetophenone | Phenolics | 13 | 99-93-4 |
| 91 | 8.93 | | 299.1223 | [M-H]- | | C_14_H_20_O_7_ | | -3.47 | | 88.76 | 299.12,119.05 | Salidroside | Phenolics | 8 | 10338-51-9 |
| 92 | 8.97 | | 133.0217 | [M-H]- | | C_4_H_6_O_5_ | | -1.24 | | 76.85 | 133.01,115.00,89.02,71.01,59.01 | Malic acid or its isomer | Organic acid | 81 | 6915-15-7 |
| 93 | 9.00 | | 165.0628 | [M-H]- | | C_9_H_10_O_3_ | | -0.99 | | 90.01 | 165.06,121.06,93.03 | p-hydroxylphenylpropanol | Phenolics | 1,11,12,16,36,40,44,47,56,61,69,72,77,86,88,95 | 501-97-3 |
| 94 | 9.06 | | 339.0797 | [M-H]- | | C_15_H_16_O_9_ | | 0.64 | | 93.22 | 339.07,177.01 | Aesculin | Coumarins | 42,44,49,50,52,55,90,98 | 531-75-9 |
| 95 | 9.19 | | 137.0309 | [M-H]- | | C_7_H_6_O_3_ | | 5.50 | | 91.60 | 137.03,109.02,93.02,81.03 | Protocatechuic aldehyde or its isomer | Phenolics | 15 | 139-85-5 |
| 96 | 9.23 | | 375.1368 | [M-H]- | | C_16_H_24_O_10_ | | 0.48 | | 91.71 | 375.13,213.07,169.08,113.02,89.02 | 8-Epiloganicacid or its isomer | Iridoids | 25,26,30,33,41 | 82509-41-9 |
| 97 | 9.25 | | 183.0367 | [M-H]- | | C_8_H_8_O_5_ | | 2.33 | | 93.28 | 183.03,125.01 | Methyl gallate | Phenolics | 23,28,29,32,35,41,43,58,72,81,86,95 | 99-24-1 |
| 98 | 9.26 | | 459.1225 | [M-H]- | | C_19_H_24_O_13_ | | -1.78 | | 98.87 | 459.11,397.11,173.00,129.01,111.00,87.00 | Parishin E or its isomer | Phenylpropanoids | 42,61,65,86 | 952068-57-4 |
| 99 | 9.29 | | 577.1401 | [M-H]- | | C_30_H_26_O_12_ | | 4.11 | | 90.29 | 577.14,451.10,425.08,407.07,289.07 | Procyanidin B1 or its isomer | Flavonoids | 23,93 | 20315-25-7 |
| 100 | 9.29 | | 193.0595 | [M-H]- | | C_10_H_10_O_4_ | | -8.08 | | 88.45 | 193.05,177.02,149.06,133.03 | Ferulic acid or its isomer | Phenylpropanoids | 13 | 1135-24-6 |
| 101 | 9.29 | | 391.1613 | [M+HCOO]- | | C_16_H_26_O_8_ | | -3.02 | | 82.70 | 345.16,161.04 | Jasminoside B | Terpenoids | 11 | 214125-04-9 |
| 102 | 9.33 | | 521.1364 | [M-H]- | | C_24_H_26_O_13_ | | 1.73 | | 90.61 | 521.12,359.08 | Iridin | Isoflavone | 15 | 491-74-7 |
| 103 | 9.33 | | 191.0276 | [M-H]- | | C_6_H_8_O_7_ | | 2.95 | | 89.40 | 191.01,173.00,129.01,111.00 | Citric acid or its isomer | Organic acid | 65 | 77-92-9 |
| 104 | 9.41 | | 325.0931 | [M-H]- | | C_15_H_18_O_8_ | | 0.31 | | 95.52 | 325.09,119.05 | (Z)-p-Coumaric acid 4-O-β-D-glucopyranside or its isomer | Coumarins | 54,82 | 117405-48-8 |
| 105 | 9.41 | | 495.1577 | [M-H]- | | C_23_H_28_O_12_ | | 0.80 | | 88.93 | 495.15,137.02 | Oxypaeoniflorin | Terpenoids | 11,23,28,30,70,79 | 39011-91-1 |
| 106 | 9.48 | | 447.1021 | [M-H]- | | C_21_H_20_O_11_ | | 2.93 | | 83.74 | 447.10,301.03,179.00 | Quercitrin or its isomer | Flavonoids | 91 | 522-12-3 |
| 107 | 9.51 | | 121.0367 | [M-H]- | | C_7_H_6_O_2_ | | -0.56 | | 95.13 | 121.03,77.03 | Benzoic Acid or its isomer | Organic acid | 29,41,42,47,55,61,72,84 | 65-85-0 |
| 108 | 9.61 | | 577.1398 | [M-H]- | | C_30_H_26_O_12_ | | 4.46 | | 73.84 | 577.13,451.10,425.08,407.07,289.07 | Proanthocyanidin B2 | Flavonoids | 3,23 | 29106-49-8 |
| 109 | 9.63 | | 515.1296 | [M-H]- | | C_25_H_24_O_12_ | | -5.44 | | 75.85 | 515.12,353.08,191.05 | 3,5-Di-O-caffeoylquinic acid or its isomer | Phenolics | 13 | 89919-62-0 |
| 110 | 9.70 | | 135.0521 | [M-H]- | | C_8_H_8_O_2_ | | -2.30 | | 81.56 | 135.04,121.02 | 4’-Hydroxyacetophenone or its isomer | Phenolics | 48 | 99-93-4 |
| 111 | 9.73 | | 331.1994 | [M-H]- | | C_20_H_28_O_4_ | | -1.91 | | 85.48 | 331.19,303.19 | Carnosic acid or its isomer | Terpenoids | 78 | 3650-09-7 |
| 112 | 9.74 | | 167.0433 | [M-H]- | | C8H8O4 | | 5.94 | | 75.82 | 167.04,123.02,93.02,67.04 | Isovanillic acid or its isomer | Phenolics | 40,44,56 | 645-08-9 |
| 113 | 9.76 | | 353.0946 | [M-H]- | | C_16_H_18_O_9_ | | 1.28 | | 95.19 | 353.09,161.02 | Chlorogenic Acid or its isomer | Phenolics | 33 | 327-97-9 |
| 114 | 9.77 | | 191.0626 | [M-H]- | | C_7_H_12_O_6_ | | 4.31 | | 85.48 | 191.06,173.04,127.03,87.03,59.01 | D-(-)-Quinic acid or its isomer | Phenolics | 4,5,6,7,10,12,29,33,40,42,44,48,52,53,55,58,61,72,83,84,85,87,90,91,93,95,98 | 36413-60-2 |
| 115 | 9.77 | | 353.0978 | [M-H]- | | C_16_H_18_O_9_ | | -7.56 | | 77.58 | 353.09,191.05,179.03,161.02,135.04,85.02 | 1-O-Caffeoylquinic acid or its isomer | Phenolics | 7,9,23,24,29,36,42,43,52,55,58,67,69,73,77,79,80,83,84,87,88,90,93,94,95 | 1241-87-8 |
| 116 | 9.78 | | 567.1798 | [M-H]- | | C_26_H_32_O_14_ | | -1.07 | | 86.46 | 567.17,405.11,243.06 | Mulberroside A | Phenolics | 23 | 102841-42-9 |
| 117 | 9.80 | | 515.1289 | [M-H]- | | C_25_H_24_O_12_ | | -4.14 | | 92.16 | 515.11,353.08,191.05,173.04,135.04,111.04,93.03,71.01 | 4,5-Dicaffeoylquinic acid or its isomer | Phenolics | 15 | 57378-72-0 |
| 118 | 9.80 | | 153.0256 | [M-H]- | | C_7_H_6_O_4_ | | 6.23 | | 89.08 | 153.02,109.02,81.03 | 2,5-dihydroxybenzoic acid or its isomer | Phenolics | 38,64 | 490-79-9 |
| 119 | 9.80 | | 289.0789 | [M-H]- | | C_15_H_14_O_6_ | | 0.46 | | 90.88 | 289.07,245.08,221.07,205.06,179.03,165.01,151.04,137.02,125.02,109.02 | (-)-Epicatechin or its isomer | Flavonoids | 59,82 | 490-46-0 |
| 120 | 9.83 | | 459.1259 | [M-H]- | | C_19_H_24_O_13_ | | 2.78 | | 98.22 | 459.11,397.11,173.00,129.01,111.00,87.00 | Parishin E or its isomer | Phenylpropanoids | 65 | 952068-57-4 |
| 121 | 9.84 | | 611.1684 | [M-H]- | | C_27_H_32_O_16_ | | 1.10 | | 95.26 | 611.16,593.15,521.13,491.11,403.10,325.07,283.06,207.05,161.06 | Hydroxysafflor yellow A | Chalcones | 23 | 78281-02-4 |
| 122 | 9.85 | | 109.0370 | [M-H]- | | C_6_H_6_O_2_ | | -1.64 | | 98.14 | 109.03,91.01,65.00,53.03 | Catechol or its isomer | Phenolics | 64 | 120-80-9 |
| 123 | 9.85 | | 389.1162 | [M-H]- | | C_16_H_22_O_11_ | | -0.04 | | 94.44 | 389.10,345.11,209.04,165.05,121.06,89.02 | Secoxyloganic acid or its isomer | Iridoids | 8,9,10,33,43,79,95,100 | 59472-23-0 |
| 124 | 9.88 | | 289.0797 | [M-H]- | | C_15_H_14_O_6_ | | -2.38 | | 96.81 | 289.07,271.06,245.08,221.08,203.07,179.03,151.04,125.01,109.02,97.02,81.03,57.03 | (+)-Catechin or its isomer | Flavonoids | 55,93 | 154-23-4 |
| 125 | 9.89 | | 153.0273 | [M-H]- | | C_7_H_6_O_4_ | | -4.42 | | 84.71 | 153.02,109.02,91.01,65.03 | Protocatechuic acid or its isomer | Organic acid | 44,47,50,61,63,64,81,83,90 | 99-50-3 |
| 126 | 9.90 | | 325.0931 | [M-H]- | | C_15_H_18_O_8_ | | -3.89 | | 94.92 | 325.09,119.05 | (Z)-p-Coumaric acid 4-O-β-D-glucopyranside or its isomer | Coumarins | 31,62 | 117405-48-8 |
| 127 | 9.91 | | 431.1064 | [M-H]- | | C_21_H_20_O_10_ | | 3.79 | | 90.90 | 431.10,311.05 | Isovitexin | Flavonoids | 22,68 | 29702-25-8 |
| 128 | 9.92 | | 163.0459 | [M-H]- | | C_9_H_8_O_3_ | | -8.66 | | 95.56 | 163.04,121.02 | Trans-2-Hydrroxycinnamic acid or its isomer | Phenolics | 31 | 614-60-8 |
| 129 | 9.97 | | 359.0854 | [M-H]- | | C_18_H_16_O_8_ | | -2.57 | | 95.18 | 359.08,197.04,179.03,161.02,135.04,123.04,71.99 | Rosmarinic Acid or its isomer | Phenolics | 13,15 | 20283-92-5 |
| 130 | 9.98 | | 373.1216 | [M-H]- | | C_16_H_22_O_10_ | | -0.68 | | 99.24 | 373.12,149.06,89.02,71.01 | Swertiamarin | Iridoids | 6,33,100 | 17388-39-5 |
| 131 | 10.01 | | 625.1508 | [M-H]- | | C_27_H_30_O_17_ | | -4.05 | | 79.69 | 625.15,463.09,301.03 | Quercetin 3-O-gentiobioside or its isomer | Flavonoids | 42 | 7431-83-6 |
| 132 | 10.01 | | 367.1108 | [M-H]- | | C_17_H_20_O_9_ | | 0.05 | | 98.43 | 367.11,135.03,93.03 | 5-Feruloylquinic acid or its isomer | Phenolics | 60 | 40242-06-6 |
| 133 | 10.02 | | 121.0375 | [M-H]- | | C_7_H_6_O_2_ | | -6.30 | | 93.55 | 121.03,93.02,65.04 | p-Hydroxybenzaldehyde or its isomer | Phenolics | 4,12,16,32,42,43,52,55,56,57,58,61,64,74,75,76,81,85,86,90,96,100 | 123-08-0 |
| 134 | 10.02 | | 137.0313 | [M-H]- | | C7H6O3 | | 2.85 | | 77.13 | 137.02,93.03 | 4-Hydroxybenzoic acid or its isomer | Phenolics | 32,53 | 99-67-7 |
| 135 | 10.03 | | 193.0584 | [M-H]- | | C_10_H_10_O_4_ | | 2.67 | | 88.96 | 193.05,177.02,149.06,133.03 | Ferulic acid or its isomer | Phenylpropanoids | 60 | 1135-24-6 |
| 136 | 10.05 | | 121.0367 | [M-H]- | | C_7_H_6_O_2_ | | -0.80 | | 90.51 | 121.03,77.03 | Benzoic Acid or its isomer | Organic acid | 54,65,70,98 | 65-85-0 |
| 137 | 10.07 | | 353.0942 | [M-H]- | | C_16_H_18_O_9_ | | 2.57 | | 95.33 | 353.09,161.02 | Chlorogenic Acid or its isomer | Phenolics | 93 | 327-97-9 |
| 138 | 10.07 | | 456.1592 | [M-H]- | | C20H27NO11 | | -1.62 | | 97.05 | 456.15,324.09,264.08,222.06,180.05,144.03,90.02 | L-Amygdalin | Cyanoside | 35,40 | 29883-16-7 |
| 139 | 10.08 | | 456.1574 | [M-H]- | | C_20_H_27_NO_11_ | | 2.17 | | 93.31 | 456.15,324.09,264.07,222.06,162.04,90.02 | Amygdalin | Cyanoside | 23,40 | 477-90-7 |
| 140 | 10.08 | | 865.2084 | [M-H]- | | C_45_H_38_O_18_ | | -4.98 | | 87.77 | 865.20,713.15,575.11,287.05 | Procyanidin C1 | Flavonoids | 93 | 37064-30-5 |
| 141 | 10.10 | | 177.0278 | [M-H]- | | C_9_H_6_O_4_ | | -6.75 | | 82.50 | 177.02,133.02,105.03,89.03 | Aesculetin | Coumarins | 43,49,52 | 305-01-1 |
| 142 | 10.10 | | 177.0261 | [M-H]- | | C_9_H_6_O_4_ | | -2.87 | | 92.88 | 177.02,133.02,105.03 | Daphnetin or its isomer | Coumarins | 17,29,41,44,50,54,79,90,98 | 486-35-1 |
| 143 | 10.14 | | 353.0952 | [M-H]- | | C_16_H_18_O_9_ | | 0.22 | | 98.96 | 353.09,191.05,179.03,161.02,135.04,85.02 | 1-O-Caffeoylquinic acid or its isomer | Phenolics | 51,60 | 1241-87-8 |
| 144 | 10.21 | | 312.1305 | [M-H]- | | C_18_H_19_NO_4_ | | 3.03 | | 87.41 | 312.12,297.10,282.07 | Norisoboldine or its isomer | Alkaloid | 66 | 23599-69-1 |
| 145 | 10.24 | | 179.0425 | [M-H]- | | C_9_H_8_O_4_ | | 1.08 | | 94.78 | 179.03,135.04,107.04,91.05 | Caffeic acid or its isomer | Phenolics | 4,5,7,8,9,10,11,16,17,20,26,30,31,32,41,42,44,47,49,50,52,53,55,58,69,71,72,73,79,83,84,85,87,88,93,95,98,100 | 331-39-5 |
| 146 | 10.34 | | 463.0981 | [M-H]- | | C_21_H_20_O_12_ | | -5.64 | | 86.46 | 463.09,301.03 | Hyperoside or its isomer | Flavonoids | 81 | 482-36-0 |
| 147 | 10.38 | | 727.2151 | [M-H]- | | C_32_H_40_O_19_ | | 2.07 | | 91.09 | 727.21,441.10,423.09 | Parishin C | Phenylpropanoids | 65 | 174972-80-6 |
| 148 | 10.40 | | 577.1454 | [M-H]- | | C_30_H_26_O_12_ | | -5.12 | | 85.83 | 577.14,451.10,425.08,407.07,289.07 | Procyanidin B1 or its isomer | Flavonoids | 23,93 | 20315-25-7 |
| 149 | 10.43 | | 593.1591 | [M-H]- | | C_27_H_30_O_15_ | | -0.98 | | 90.20 | 593.15,473.10 | Vicenin II or its isomer | Flavonoids | 38,80 | 23666-13-9 |
| 150 | 10.54 | | 485.1912 | [M-H]- | | C_26_H_30_O_9_ | | -4.50 | | 84.29 | 485.19,397.20 | Rutaevin | Alkaloid | 18 | 33237-37-5 |
| 151 | 10.56 | | 275.0980 | [M-H]- | | C_15_H_16_O_5_ | | 6.32 | | 84.88 | 275.09,257.08,217.05 | Hamaudol or its isomer | Flavonoids | 66 | 735-46-6 |
| 152 | 10.63 | | 493.1226 | [M-H]- | | C_26_H_22_O_10_ | | -2.60 | | 91.35 | 493.12,313.07,295.06,277.05,185.02,159.04,135.04,109.02 | Salvianolic acid A or its isomer | Phenolics | 13,15 | 96574-01-5 |
| 153 | 10.72 | | 353.0955 | [M-H]- | | C_16_H_18_O_9_ | | -1.13 | | 94.88 | 353.09,161.02 | Chlorogenic Acid or its isomer | Phenolics | 73 | 327-97-9 |
| 154 | 10.74 | | 191.0637 | [M-H]- | | C_7_H_12_O_6_ | | -1.41 | | 92.77 | 191.06,173.04,127.03,87.03,59.01 | D-(-)-Quinic acid or its isomer | Phenolics | 93 | 36413-60-2 |
| 155 | 10.74 | | 353.0958 | [M-H]- | | C_16_H_18_O_9_ | | 2.16 | | 75.98 | 353.09,191.05,179.03,161.02,135.04,85.02 | 1-O-Caffeoylquinic acid or its isomer | Phenolics | 23,86 | 1241-87-8 |
| 156 | 10.75 | | 729.2643 | [M+HCOO]- | | C_32_H_44_O_16_ | | 2.01 | | 96.82 | 683.26,521.20,359.15 | Clemastanin B | Flavonoids | 31 | 112747-98-5 |
| 157 | 10.77 | | 331.1990 | [M-H]- | | C_20_H_28_O_4_ | | -0.69 | | 95.20 | 331.19,303.19 | Carnosic acid or its isomer | Terpenoids | 78 | 3650-09-7 |
| 158 | 10.79 | | 415.1148 | [M-H]- | | C_21_H_20_O_9_ | | -9.75 | | 81.97 | 415.11,295.06,267.06 | Puerarin or its isomer | Isoflavone | 71,85 | 3681-99-0 |
| 159 | 10.85 | | 403.1326 | [M-H]- | | C17H24O11 | | -1.89 | | 90.90 | 403.13,121.02,113.02,101.02,85.02,71.01 | Oleoside 11-methyl ester | Iridoids | 95 | 60539-23-3 |
| 160 | 10.97 | | 289.0796 | [M-H]- | | C_15_H_14_O_6_ | | -1.80 | | 90.65 | 289.07,245.08,221.07,205.06,179.03,165.01,151.04,137.02,125.02,109.02 | (-)-Epicatechin or its isomer | Flavonoids | 23 | 490-46-0 |
| 161 | 10.97 | | 445.1233 | [M-H]- | | C_22_H_22_O_10_ | | 0.79 | | 98.52 | 445.12,325.07 | 3'-Methoxypuerarin or its isomer | Isoflavone | 68,71 | 117047-07-1 |
| 162 | 11.00 | | 289.0796 | [M-H]- | | C_15_H_14_O_6_ | | -1.79 | | 90.31 | 289.07,271.06,245.08,221.08,203.07,179.03,151.04,125.01,109.02,97.02,81.03,57.03 | (+)-Catechin or its isomer | Flavonoids | 93 | 154-23-4 |
| 163 | 11.06 | | 593.1606 | [M-H]- | | C_27_H_30_O_15_ | | -3.61 | | 93.80 | 593.15,473.10 | Vicenin II or its isomer | Flavonoids | 8,17,43,44,45,54,55,73,83 | 23666-13-9 |
| 164 | 11.17 | | 525.1612 | [M+HCOO]- | | C23H28O11 | | 4.07 | | 90.08 | 479.16,357.11,317.10,283.08,121.02,77.03 | Albiflorin or its isomer | Terpenoids | 35,40 | 39011-90-0 |
| 165 | 11.22 | | 577.1611 | [M-H]- | | C_27_H_30_O_14_ | | 3.93 | | 87.60 | 577.16,413.08,293.04 | 2''-O-Rhamnosylvitexin or its isomer | Flavonoids | 91 | 64820-99-1 |
| 166 | 11.24 | | 135.0522 | [M-H]- | | C8H8O2 | | 1.69 | | 92.23 | 135.04,121.02 | 4’-Hydroxyacetophenone or its isomer | Phenolics | 62,83 | 99-93-4 |
| 167 | 11.25 | | 479.0904 | [M-H]- | | C21H20O13 | | 0.04 | | 80.52 | 479.09,317.03 | Myricetin 3'-O-β-D-glucopyranoside or its isomer | Flavonoids | 67 | 520-14-9 |
| 168 | 11.27 | | 515.1292 | [M-H]- | | C_25_H_24_O_12_ | | -4.76 | | 94.07 | 515.12,353.08,191.05 | 3,5-Di-O-caffeoylquinic acid or its isomer | Phenolics | 52,83,95 | 89919-62-0 |
| 169 | 11.27 | | 515.1289 | [M-H]- | | C_25_H_24_O_12_ | | -3.74 | | 96.20 | 515.12,353.08,179.03,135.04 | 1,4-Dicaffeoylquinic acid or its isomer | Phenolics | 7,11,17,29,44,61,72 | 1182-34-9 |
| 170 | 11.28 | | 209.0485 | [M+HCOO]- | | C_9_H_8_O_3_ | | 6.90 | | 78.11 | 163.04,121.02 | Trans-2-Hydrroxycinnamic acid or its isomer | Phenolics | 54 | 614-60-8 |
| 171 | 11.28 | | 133.0217 | [M-H]- | | C_4_H_6_O_5_ | | 1.66 | | 87.36 | 133.01,115.00,89.02,71.01,59.01 | Malic acid or its isomer | Organic acid | 49,50 | 6915-15-7 |
| 172 | 11.29 | | 209.0473 | [M+HCOO]- | | C_9_H_8_O_3_ | | -0.47 | | 82.97 | 163.04,119.05 | p-Coumaric acid or its isomer | Phenolics | 70 | 501-98-4 |
| 173 | 11.29 | | 515.1292 | [M-H]- | | C_25_H_24_O_12_ | | -4.62 | | 85.09 | 515.12,353.08,179.03 | 1,3-O-Dicaffeoylquinic acid or its isomer | Phenolics | 42 | 19870-46-3 |
| 174 | 11.41 | | 191.0628 | [M-H]- | | C_7_H_12_O_6_ | | -3.31 | | 83.74 | 191.06,173.04,127.03,87.03,59.01 | D-(-)-Quinic acid or its isomer | Phenolics | 16,60,72 | 36413-60-2 |
| 175 | 11.41 | | 433.1238 | [M-H]- | | C_21_H_22_O_10_ | | -5.86 | | 85.33 | 433.12,271.06,151.00,119.05 | Naringenin-7-O-β-D-glucoside or its isomer | Flavonoids | 45,73 | 529-55-5 |
| 176 | 11.42 | | 367.1116 | [M-H]- | | C17H20O9 | | -2.30 | | 94.66 | 367.11,135.03,93.03 | 5-Feruloylquinic acid or its isomer | Phenolics | 2,17,20,29,49,51,55,67,95 | 40242-06-6 |
| 177 | 11.57 | | 403.1333 | [M-H]- | | C17H24O11 | | -3.54 | | 93.32 | 403.12,371.09,223.06,179.05,143.03,119.03,59.01 | Secoxyloganin | Iridoids | 7,8,33,95,100 | 58822-47-2 |
| 178 | 11.59 | | 247.0265 | [M+HCOO]- | | C_11_H_6_O_4_ | | -0.34 | | 94.28 | 201.02,173.02,145.02,117.03 | Bergaptol or its isomer | Coumarins | 98 | 486-60-2 |
| 179 | 11.60 | | 525.1637 | [M+HCOO]- | | C23H28O11 | | -1.15 | | 95.13 | 479.15,165.05,121.02,77.03 | Paeoniflorin or its isomer | Terpenoids | 35 | 23180-57-6 |
| 180 | 11.63 | | 623.2061 | [M-H]- | | C29H36O15 | | -1.09 | | 97.53 | 623.20,461.17 | Isoacteoside or its isomer | Flavonoids | 73 | 61303-13-7 |
| 181 | 11.63 | | 325.0931 | [M-H]- | | C_15_H_18_O_8_ | | -1.70 | | 93.99 | 325.09,119.05 | (Z)-p-Coumaric acid 4-O-β-D-glucopyranside or its isomer | Coumarins | 11,58 | 117405-48-8 |
| 182 | 11.64 | | 609.1557 | [M-H]- | | C27H30O16 | | -3.86 | | 91.83 | 609.15,301.03 | Rutin or its isomer | Flavonoids | 83 | 153-18-4 |
| 183 | 11.65 | | 625.1524 | [M-H]- | | C_27_H_30_O_17_ | | -1.08 | | 95.39 | 625.15,301.14 | Quercetin 3-O-sophoroside or its isomer | Flavonoids | 74 | 18609-17-1 |
| 184 | 11.67 | | 167.0421 | [M-H]- | | C_8_H_8_O_4_ | | -1.07 | | 85.02 | 167.04,123.02,93.02,67.04 | Isovanillic acid or its isomer | Phenolics | 96 | 645-08-9 |
| 185 | 11.68 | | 275.0271 | [M-H]- | | C_13_H_8_O_7_ | | 0.26 | | 85.50 | 275.02,201.01 | 3,4,8,9,10-Pentahydroxy Urolithin or its isomer | Phenolics | 43,98 | 91485-02-8 |
| 186 | 11.69 | | 563.1526 | [M-H]- | | C_26_H_28_O_14_ | | -0.55 | | 95.81 | 563.15,473.10,425.08,353.06 | Vicenin I | Flavonoids | 11,49,54,61,95 | 35927-38-9 |
| 187 | 11.70 | | 405.1268 | [M-H]- | | C20H22O9 | | -0.98 | | 98.71 | 405.12,243.06 | Piceatannol 3'-O-glucoside or its isomer | Stilbene glycosides | 12,42 | 94356-26-0 |
| 188 | 11.75 | | 755.2115 | [M-H]- | | C_33_H_40_O_20_ | | 0.31 | | 99.81 | 755.21,299.02 | Alcesefoliside | Flavonoids | 31,62 | 124151-38-8 |
| 189 | 11.79 | | 163.0476 | [M-H]- | | C_9_H_8_O_3_ | | -1.35 | | 91.28 | 163.04,121.02 | Trans-2-Hydrroxycinnamic acid or its isomer | Phenolics | 8,9,20,25,31,40,41,49,53,58,64,69,81,88,90,95 | 614-60-8 |
| 190 | 11.79 | | 163.0473 | [M-H]- | | C_9_H_8_O_3_ | | -0.07 | | 89.39 | 163.04,119.05 | p-Coumaric acid or its isomer | Phenolics | 44,45,50,51,53,60,68,74,79,82,98 | 501-98-4 |
| 191 | 11.84 | | 461.0821 | [M-H]- | | C_21_H_18_O_12_ | | -2.10 | | 96.18 | 461.07,285.04,113.02 | Luteolin-3-D-glucuronide or its isomer | Flavonoids | 7 | 53527-42-7 |
| 192 | 11.90 | | 639.2010 | [M-H]- | | C_29_H_36_O_16_ | | -4.06 | | 89.67 | 639.20,477.12,315.10,161.02 | Plantamajoside | Flavonoids | 9,10 | 104777-68-6 |
| 193 | 11.92 | | 625.1510 | [M-H]- | | C27H30O17 | | -4.35 | | 72.71 | 625.15,463.09,301.03 | Quercetin 3-O-gentiobioside or its isomer | Flavonoids | 67 | 7431-83-6 |
| 194 | 11.97 | | 289.0795 | [M-H]- | | C_15_H_14_O_6_ | | 1.65 | | 96.87 | 289.07,271.06,245.08,221.08,203.07,179.03,151.04,125.01,109.02,97.02,81.03,57.03 | (+)-Catechin or its isomer | Flavonoids | 60 | 154-23-4 |
| 195 | 12.01 | | 479.0911 | [M-H]- | | C21H20O13 | | -1.50 | | 98.43 | 479.09,317.03 | Myricetin 3'-O-β-D-glucopyranoside or its isomer | Flavonoids | 31,43,67 | 520-14-9 |
| 196 | 12.06 | | 577.1648 | [M-H]- | | C_27_H_30_O_14_ | | -2.12 | | 85.95 | 577.16,417.15 | Rhoifolin or its isomer | Flavonoids | 55 | 17306-46-6 |
| 197 | 12.08 | | 179.0426 | [M-H]- | | C_9_H_8_O_4_ | | 1.66 | | 89.88 | 179.03,135.04,107.04,91.05 | Caffeic acid or its isomer | Phenolics | 49,98 | 331-39-5 |
| 198 | 12.08 | | 161.0314 | [M-H]- | | C_9_H_6_O_3_ | | 2.08 | | 94.08 | 161.02,133.02,105.03,89.03,77.04,66.00 | 7-Hydroxycoumarin or its isomer | Coumarins | 5,40,42,44,45,58,61,68,86 | 93-35-6 |
| 199 | 12.11 | | 595.1413 | [M-H]- | | C_26_H_28_O_16_ | | 0.58 | | 99.64 | 595.14,301.03 | Quercetin-3-O-β-D-ribosyl-(1→2)-β-D-glucoside | Flavonoids | 19,44,49,50,74 | 83048-35-5 |
| 200 | 12.12 | | 577.1655 | [M-H]- | | C_27_H_30_O_14_ | | -4.17 | | 86.35 | 577.16,413.08,293.04 | 2''-O-Rhamnosylvitexin or its isomer | Flavonoids | 98 | 64820-99-1 |
| 201 | 12.13 | | 525.1637 | [M+HCOO]- | | C23H28O11 | | 0.67 | | 95.62 | 479.16,165.05,121.02,77.03 | Paeoniflorin or its isomer | Terpenoids | 35 | 23180-57-6 |
| 202 | 12.16 | | 191.0635 | [M-H]- | | C_7_H_12_O_6_ | | 0.76 | | 81.69 | 191.06,173.04,127.03,87.03,59.01 | D-(-)-Quinic acid or its isomer | Phenolics | 16,51 | 36413-60-2 |
| 203 | 12.17 | | 367.1115 | [M-H]- | | C17H20O9 | | -2.07 | | 88.30 | 367.11,161.02,135.04,85.02 | 3-O-Caffeoylquinic acid methyl ester | Phenolics | 95 | 123483-19-2 |
| 204 | 12.18 | | 567.2098 | [M+HCOO]- | | C_26_H_34_O_11_ | | -3.05 | | 85.80 | 521.20,359.15,161.04 | Lariciresinol-4-O-β-D-glucopyranoside | Lignans | 9 | 143663-00-7 |
| 205 | 12.19 | | 639.2021 | [M-H]- | | C29H36O16 | | -2.71 | | 77.87 | 639.20,161.02 | Plantainoside D | Phenylpropanoids | 73 | 147331-98-4 |
| 206 | 12.20 | | 625.1480 | [M-H]- | | C27H30O17 | | 0.52 | | 97.47 | 625.15,301.14 | Quercetin 3-O-sophoroside or its isomer | Flavonoids | 67 | 18609-17-1 |
| 207 | 12.21 | | 469.1365 | [M+HCOO]- | | C20H24O10 | | 0.99 | | 87.21 | 423.13,261.07,243.06,179.05,161.04,143.03,119.03 | Smyrindioloside | Carbohydrate | 95 | 87592-77-6 |
| 208 | 12.29 | | 739.2172 | [M-H]- | | C_33_H_40_O_19_ | | 1.13 | | 99.66 | 739.21,309.15 | Clitorin | Flavonoids | 31,62 | 55804-74-5 |
| 209 | 12.40 | | 785.2600 | [M-H]- | | C_35_H_46_O_20_ | | -2.15 | | 96.96 | 785.26,623.21 | Echinacoside | Phenylethanoid glycosides | 65 | 82854-37-3 |
| 210 | 12.43 | | 623.2073 | [M-H]- | | C_29_H_36_O_15_ | | -3.09 | | 93.19 | 623.20,461.17 | Isoacteoside or its isomer | Flavonoids | 33 | 61303-13-7 |
| 211 | 12.44 | | 473.0810 | [M-H]- | | C_22_H_18_O_12_ | | -2.52 | | 95.54 | 473.07,293.03,179.03,161.02,149.00,135.04,113.00 | Cichoric acid | Phenylpropanoids | 52,69,88 | 70831-56-0 |
| 212 | 12.44 | | 193.0583 | [M-H]- | | C_10_H_10_O_4_ | | 2.07 | | 92.63 | 193.05,177.02,149.06,133.03 | Ferulic acid or its isomer | Phenylpropanoids | 5,16,17,23,41,42,51,55,60,61,72,73,84,86,87,95 | 1135-24-6 |
| 213 | 12.44 | | 787.1071 | [M-H]- | | C34H28O22 | | 0.10 | | 78.07 | 787.10,617.07,465.06,423.05,295.04,169.01 | 1,2,3,6-Tetra-O-galloy-β-D-glucopyranose | Carbohydrate | 35 | 79886-50-3 |
| 214 | 12.45 | | 463.0964 | [M-H]- | | C21H20O12 | | -1.92 | | 88.16 | 463.09,301.03 | Hyperoside or its isomer | Flavonoids | 83 | 482-36-0 |
| 215 | 12.46 | | 249.0835 | [M-H]- | | C_13_H_14_O_5_ | | 2.33 | | 90.53 | 249.08,205.08,177.09,161.09 | Citrinin | Polyketides | 64 | 518-75-2 |
| 216 | 12.48 | | 479.0918 | [M-H]- | | C21H20O13 | | -2.97 | | 94.66 | 479.09,317.03 | Myricetin 3-O-galactoside | Flavonoids | 62,67 | 15648-86-9 |
| 217 | 12.54 | | 525.1636 | [M+HCOO]- | | C23H28O11 | | -0.86 | | 94.09 | 479.15,165.05,121.02,77.03 | Paeoniflorin or its isomer | Terpenoids | 35 | 23180-57-6 |
| 218 | 12.54 | | 595.1777 | [M-H]- | | C_27_H_32_O_15_ | | -6.07 | | 86.19 | 595.17,459.11,287.05,151.00 | Neoeriocitrin or its isomer | Flavonoids | 45 | 13241-32-2 |
| 219 | 12.56 | | 477.1497 | [M-H]- | | C_23_H_26_O_11_ | | -3.67 | | 84.94 | 477.14,161.02 | Calceolarioside B or its isomer | Phenylethanoid glycosides | 9 | 105471-98-5 |
| 220 | 12.57 | | 431.1068 | [M-H]- | | C_21_H_20_O_10_ | | -2.75 | | 96.66 | 431.10,371.07,293.04,269.04,253.05,163.04,101.02 | Aloe-emodin-8-O-β-D-glucopyranoside | Anthraquinone | 93 | 33037-46-6 |
| 221 | 12.57 | | 549.1699 | [M-H]- | | C26H30O13 | | -2.21 | | 97.76 | 549.16,255.06 | Isoliquiritin apioside or its isomer | Flavonoids | 1,5,11,12,16,17,28,40,44,47,61,73,77 | 120926-46-7 |
| 222 | 12.59 | | 161.0320 | [M-H]- | | C_9_H_6_O_3_ | | 1.79 | | 77.10 | 161.02,133.02,105.03,89.03,77.04,66.00 | 7-Hydroxycoumarin or its isomer | Coumarins | 51,53 | 93-35-6 |
| 223 | 12.61 | | 269.0543 | [M-H]- | | C_15_H_10_O_5_ | | -5.47 | | 89.90 | 269.05,241.04 | Aloeemodin or its isomer | Anthraquinone | 93 | 481-72-1 |
| 224 | 12.61 | | 223.0695 | [M-H]- | | C_11_H_12_O_5_ | | 4.56 | | 95.24 | 223.06,207.03 | Sinapic acid | Phenylpropanoids | 51 | 530-59-6 |
| 225 | 12.62 | | 577.1633 | [M-H]- | | C_27_H_30_O_14_ | | 0.43 | | 87.08 | 577.16,417.15 | Rhoifolin or its isomer | Flavonoids | 45 | 17306-46-6 |
| 226 | 12.64 | | 577.1658 | [M-H]- | | C_27_H_30_O_14_ | | -1.78 | | 96.79 | 577.16,413.08,293.04 | 2''-O-Rhamnosylvitexin or its isomer | Flavonoids | 86 | 64820-99-1 |
| 227 | 12.64 | | 609.1541 | [M-H]- | | C27H30O16 | | -1.15 | | 98.31 | 609.15,301.02 | Quercetin-3-O-D-glucosyl-(1-2)-L-rhamnoside or its isomer | Flavonoids | 59,67,95,98 | 143016-74-4 |
| 228 | 12.68 | | 417.1263 | [M-H]- | | C_21_H_22_O_9_ | | 0.21 | | 95.28 | 417.12,257.06,135.00 | Liquiritin or its isomer | Flavonoids | 12,36,69,73,88,95 | 551-15-5 |
| 229 | 12.70 | | 445.1229 | [M-H]- | | C_22_H_22_O_10_ | | 3.61 | | 96.87 | 445.12,325.07 | 3'-Methoxypuerarin or its isomer | Isoflavone | 68,91 | 117047-07-1 |
| 230 | 12.71 | | 431.1073 | [M-H]- | | C_21_H_20_O_10_ | | -3.89 | | 88.39 | 431.10,341.06,311.05,283.06 | Vitexin | Flavonoids | 38,83 | 3681-93-4 |
| 231 | 12.71 | | 609.1584 | [M-H]- | | C_27_H_30_O_16_ | | -4.27 | | 86.41 | 609.15,301.03 | Rutin or its isomer | Flavonoids | 20,41,79,80 | 153-18-4 |
| 232 | 12.71 | | 417.0897 | [M-H]- | | C20H18O10 | | 0.83 | | 87.29 | 417.08,175.03 | Salvianolic acid D or its isomer | Phenolics | 32 | 142998-47-8 |
| 233 | 12.72 | | 417.1225 | [M-H]- | | C_21_H_22_O_9_ | | -8.67 | | 84.71 | 417.12,255.06 | Isoliquiritin or its isomer | Chalcones | 24 | 5041-81-6 |
| 234 | 12.75 | | 593.1603 | [M-H]- | | C_27_H_30_O_15_ | | -3.16 | | 95.51 | 593.16,285.04,175.02,113.02,59.01 | Kaempferol-3-O-glucorhamnoside or its isomer | Flavonoids | 52 | 40437-72-7 |
| 235 | 12.77 | | 623.2069 | [M-H]- | | C_29_H_36_O_15_ | | -2.44 | | 97.37 | 623.20,461.17,415.10 | Forsythoside I or its isomer | Lignans | 7,19,33,80 | 79916-77-1 |
| 236 | 12.79 | | 463.0960 | [M-H]- | | C_21_H_20_O_12_ | | -1.09 | | 93.97 | 463.09,301.03 | Hyperoside or its isomer | Flavonoids | 49,52 | 482-36-0 |
| 237 | 12.79 | | 301.0062 | [M-H]- | | C14H6O8 | | 0.35 | | 93.77 | 301.00,285.00,259.01,230.01,201.01,185.02,145.02,129.03 | Ellagic acid | Tannin | 25,28,30,35,41,43,55,59,79 | 476-66-4 |
| 238 | 12.80 | | 449.1183 | [M-H]- | | C_21_H_22_O_11_ | | -2.19 | | 92.10 | 449.11,431.09,287.05,151.00 | (2R)-and (2S)-eriodictyol-7-O-β-D-glucopyranoside | Flavonoids | 61 | 38965-51-4 |
| 239 | 12.82 | | 537.1121 | [M-H]- | | C_27_H_22_O_12_ | | -1.86 | | 94.78 | 537.11,493.11,313.07,295.06,277.04,185.02,109.03 | Lithospermic acid or its isomer | Organic acid | 26,32,71 | 28831-65-4 |
| 240 | 12.83 | | 525.3088 | [M+HCOO]- | | C_27_H_44_O_7_ | | 0.26 | | 93.80 | 479.30,319.19,159.10 | Hydroxyecdysone | Organic acid | 40 | 5289-74-7 |
| 241 | 12.84 | | 441.0893 | [M-H]- | | C_22_H_18_O_10_ | | 1.54 | | 91.59 | 441.08,331.05,289.07,169.01 | Epicatechin gallate | Flavonoids | 82,93 | 1257-08-5 |
| 242 | 12.84 | | 463.0957 | [M-H]- | | C_21_H_20_O_12_ | | -0.50 | | 97.20 | 463.09,343.04,299.02,271.02,177.99 | Isoquercitrin or its isomer | Flavonoids | 41,42,43,44,54,68 | 21637-25-2 |
| 243 | 12.86 | | 445.0865 | [M-H]- | | C_21_H_18_O_11_ | | -3.55 | | 90.22 | 445.08,325.03,309.02,283.02,251.04 | Rhein-8-O-β-D-glucopyranoside | Anthraquinone | 81,82,93 | 34298-86-7 |
| 244 | 12.87 | | 193.0574 | [M-H]- | | C_10_H_10_O_4_ | | 2.69 | | 90.08 | 193.05,177.02,149.06,133.03 | Ferulic acid or its isomer | Phenylpropanoids | 84,87 | 1135-24-6 |
| 245 | 12.89 | | 595.1763 | [M-H]- | | C27H32O15 | | -3.58 | | 90.23 | 595.17,459.11,287.05,151.00 | Neoeriocitrin or its isomer | Flavonoids | 45,95 | 13241-32-2 |
| 246 | 12.92 | | 405.1277 | [M-H]- | | C20H22O9 | | -3.21 | | 96.31 | 405.12,243.06 | Piceatannol 3'-O-glucoside or its isomer | Stilbene glycosides | 12,42 | 94356-26-0 |
| 247 | 12.92 | | 193.0582 | [M-H]- | | C_10_H_10_O_4_ | | -1.36 | | 87.44 | 193.05,161.02,133.03 | Caffeic Acid Methyl Ester | Phenylpropanoids | 29 | 3843-74-1 |
| 248 | 12.92 | | 121.0379 | [M-H]- | | C_7_H_6_O_2_ | | 9.58 | | 93.66 | 121.03,77.03 | Benzoic Acid or its isomer | Organic acid | 12,44,51,53,54,85,90,100 | 65-85-0 |
| 249 | 12.92 | | 415.1109 | [M-H]- | | C_21_H_20_O_9_ | | 0.29 | | 90.38 | 415.11,295.06,267.06 | Puerarin or its isomer | Isoflavone | 71 | 3681-99-0 |
| 250 | 12.93 | | 243.0736 | [M-H]- | | C_14_H_12_O_4_ | | -0.25 | | 93.00 | 243.07,225.05,201.05,175.07 | Piceatannol | Stilbene glycosides | 42 | 10083-24-6 |
| 251 | 12.96 | | 631.1757 | [M-H]- | | C_30_H_32_O_15_ | | -2.54 | | 90.20 | 631.17,613.15,313.05 | Galloyl paeoniflorin | Terpenoids | 23,30,35,42,69,88 | 122965-41-7 |
| 252 | 12.97 | | 463.0977 | [M-H]- | | C21H20O12 | | -4.90 | | 74.33 | 463.09,301.03 | Quercetin-7-O-β-D-glucopyranoside or its isomer | Flavonoids | 7,79,83,95 | 491-50-9 |
| 253 | 12.97 | | 461.0822 | [M-H]- | | C_21_H_18_O_12_ | | -5.16 | | 82.59 | 461.08,285.04,113.02,85.02 | Scutellarin B | Flavonoids | 2,52,81 | 27740-01-8 |
| 254 | 12.98 | | 461.0811 | [M-H]- | | C_21_H_18_O_12_ | | 2.74 | | 92.05 | 461.08,285.04,175.02,113.02,59.01 | Kaempferol-3-O-β-D-glucuronide | Flavonoids | 3,4,5,7,10,48,49,50,51,53,62,100 | 22688-78-4 |
| 255 | 12.98 | | 525.1640 | [M+HCOO]- | | C23H28O11 | | -1.81 | | 95.08 | 479.15,165.05,121.02,77.03 | Paeoniflorin or its isomer | Terpenoids | 35 | 23180-57-6 |
| 256 | 12.98 | | 449.1184 | [M-H]- | | C_21_H_22_O_11_ | | 4.80 | | 95.25 | 449.11,303.05,151.00 | Neoisoastilbin or its isomer | Flavonoids | 16 | 54141-72-9 |
| 257 | 13.00 | | 461.0793 | [M-H]- | | C_21_H_18_O_12_ | | 1.19 | | 97.59 | 461.07,285.04,113.02 | Luteolin-3-D-glucuronide or its isomer | Flavonoids | 1,16,43,54,55 | 53527-42-7 |
| 258 | 13.00 | | 269.0544 | [M-H]- | | C_15_H_10_O_5_ | | -5.67 | | 92.84 | 269.05,241.05,225.05,197.06,181.06 | Emodin or its isomer | Quinones | 9,57,71 | 518-82-1 |
| 259 | 13.03 | | 593.1627 | [M-H]- | | C_27_H_30_O_15_ | | -7.18 | | 87.51 | 593.16,285.03,151.00 | Kaempferol 3-rutinoside or its isomer | Flavonoids | 33 | 17650-84-9 |
| 260 | 13.03 | | 447.1017 | [M-H]- | | C_21_H_20_O_11_ | | -2.47 | | 94.00 | 447.10,285.04 | Kaemferol 7-O-b-D-glucoside | Flavonoids | 6,8,10,17,52,80 | 16290-07-6 |
| 261 | 13.04 | | 447.1042 | [M-H]- | | C_21_H_20_O_11_ | | -8.03 | | 87.27 | 447.10,285.04 | Luteolin-7-O-β-D-glucoside or its isomer | Flavonoids | 33,90,95 | 5373-11-5 |
| 262 | 13.06 | | 623.2079 | [M-H]- | | C_29_H_36_O_15_ | | -3.96 | | 89.51 | 623.20,461.17 | Isoacteoside or its isomer | Flavonoids | 30 | 61303-13-7 |
| 263 | 13.07 | | 593.1590 | [M-H]- | | C_27_H_30_O_15_ | | -0.83 | | 85.43 | 593.15,413.08 | Vitexin -4''-O-glucoside | Flavonoids | 6 | 178468-00-3 |
| 264 | 13.11 | | 623.2082 | [M-H]- | | C_29_H_36_O_15_ | | 4.41 | | 89.30 | 623.20,461.16,161.02,113.02 | Verbascoside or its isomer | Phenylpropanoids | 5,40 | 61276-17-3 |
| 265 | 13.13 | | 491.0908 | [M-H]- | | C_22_H_20_O_13_ | | -1.34 | | 94.03 | 491.09,327.02 | 3,3'-Di-O-methylellagic acid 4'-glucoside | Tannin | 43 |  |
| 266 | 13.21 | | 479.0908 | [M-H]- | | C21H20O13 | | -0.92 | | 98.67 | 479.09,317.03 | Myricetin 3'-O-β-D-glucopyranoside or its isomer | Flavonoids | 67 | 520-14-9 |
| 267 | 13.24 | | 521.1383 | [M-H]- | | C24H26O13 | | -1.84 | | 97.57 | 521.13,323.07,197.04,179.03,161.02 | Salviaflaside | Phenylpropanoids | 25,32,54,72 | 178895-25-5 |
| 268 | 13.28 | | 477.1491 | [M-H]- | | C23H26O11 | | -3.39 | | 89.26 | 477.14,161.02 | Calceolarioside B or its isomer | Phenylethanoid glycosides | 73 | 105471-98-5 |
| 269 | 13.29 | | 515.1269 | [M-H]- | | C_25_H_24_O_12_ | | 0.26 | | 98.27 | 515.13,353.08,191.05 | 3,5-Di-O-caffeoylquinicacid or its isomer | Phenolics | 98 | 2450-53-5 |
| 270 | 13.33 | | 515.1266 | [M-H]- | | C25H24O12 | | 0.30 | | 86.33 | 515.12,353.08,179.03 | 1,3-O-Dicaffeoylquinic acid or its isomer | Phenolics | 33,83 | 19870-46-3 |
| 271 | 13.35 | | 137.0319 | [M-H]- | | C_7_H_6_O_3_ | | -1.52 | | 96.02 | 137.03,93.03 | Salicylic Acid or its isomer | Organic acid | 12,29,41,52,54,55,64,69,76,79,81,85,88,90,99 | 69-72-7 |
| 272 | 13.36 | | 939.1217 | [M-H]- | | C41H32O26 | | -3.73 | | 75.25 | 939.12,259.02 | 1,2,3,4,6-pentagalloylglucose | Carbohydrate | 35 | 14937-32-7 |
| 273 | 13.38 | | 609.1558 | [M-H]- | | C_27_H_30_O_16_ | | 3.68 | | 95.13 | 609.15,301.02 | Quercetin-3-O-D-glucosyl-(1-2)-L-rhamnoside or its isomer | Flavonoids | 62 | 143016-74-4 |
| 274 | 13.38 | | 519.1988 | [M-H]- | | C_26_H_32_O_11_ | | -8.27 | | 82.24 | 519.19,357.13 | Matairesinoside or its isomer | Lignans | 29 | 23202-85-9 |
| 275 | 13.39 | | 137.0317 | [M-H]- | | C_7_H_6_O_3_ | | -0.14 | | 90.43 | 137.02,93.03 | 4-Hydroxybenzoic acid or its isomer | Phenolics | 5,8,16,17,23,38,46,47,50,56,63,65,67,70,72,73,80,83,84,86,95,98 | 99-67-7 |
| 276 | 13.43 | | 515.1292 | [M-H]- | | C_25_H_24_O_12_ | | -4.75 | | 87.41 | 515.12,353.08,179.03,135.04 | 1,4-Dicaffeoylquinic acid or its isomer | Phenolics | 29,54 | 1182-34-9 |
| 277 | 13.45 | | 515.1296 | [M-H]- | | C_25_H_24_O_12_ | | -5.45 | | 91.01 | 515.13,353.08,173.04 | Isochlorogenic acid B or its isomer | Phenolics | 6,7,8,44,52,55,58,61,93,95 | 14534-61-3 |
| 278 | 13.45 | | 579.1804 | [M-H]- | | C27H32O14 | | -2.11 | | 92.36 | 579.18,459.11,313.07,271.06 | Naringin or its isomer | Flavonoids | 34,45,73 | 10236-47-2 |
| 279 | 13.47 | | 593.1581 | [M-H]- | | C_27_H_30_O_15_ | | 0.59 | | 91.98 | 593.16,285.04,175.02,113.02,59.01 | Kaempferol-3-O-glucorhamnoside or its isomer | Flavonoids | 23 | 40437-72-7 |
| 280 | 13.48 | | 593.1604 | [M-H]- | | C27H30O15 | | 3.21 | | 87.53 | 593.16,285.03,151.00 | Kaempferol 3-rutinoside or its isomer | Flavonoids | 59,62,95 | 17650-84-9 |
| 281 | 13.50 | | 593.1576 | [M-H]- | | C_27_H_30_O_15_ | | 2.67 | | 93.03 | 593.15,333.49 | Kaempferol-3-O-neohesperidoside | Flavonoids | 8,96 | 32602-81-6 |
| 282 | 13.52 | | 685.2465 | [M-H]- | | C_31_H_42_O_17_ | | 1.78 | | 93.52 | 685.24,523,18,453.14,299.11,223.06,89.02 | Specnuezhenide | Terpenoids | 79 | 39011-92-2 |
| 283 | 13.53 | | 623.2061 | [M-H]- | | C_29_H_36_O_15_ | | -1.01 | | 97.24 | 623.20,461.16,161.02,113.02 | Verbascoside or its isomer | Phenylpropanoids | 30,52 | 61276-17-3 |
| 284 | 13.53 | | 623.2080 | [M-H]- | | C_29_H_36_O_15_ | | -4.14 | | 95.58 | 623.20,461.17,415.10 | Forsythoside I or its isomer | Lignans | 56,81 | 79916-77-1 |
| 285 | 13.55 | | 623.2060 | [M-H]- | | C_29_H_36_O_15_ | | -0.92 | | 98.56 | 623.20,461.17 | Isoacteoside or its isomer | Flavonoids | 9,42,72,73 | 61303-13-7 |
| 286 | 13.55 | | 541.1421 | [M-H]- | | C_27_H_26_O_12_ | | 0.53 | | 95.34 | 541.14,313.05,169.01 | 4-[(1E)-2-(3,5-Dihydroxyphenyl)ethenyl]phenyl β-D-glucopyranoside 6-(3,4,5-trihydroxybenzoate) | Flavonoids | 9,93 | 64898-03-9 |
| 287 | 13.58 | | 767.4312 | [M+HCOO]- | | C_39_H_62_O_12_ | | 3.54 | | 89.71 | 721.43,460.45 | Liriopesides B | Terpenoids | 18 | 87425-34-1 |
| 288 | 13.59 | | 579.2176 | [M-H]- | | C_28_H_36_O_13_ | | -3.47 | | 84.25 | 579.21,417.15,181.05 | Episyringaresinol 4'-O-β-D-glncopyranoside | Lignans | 29 | 137038-13-2 |
| 289 | 13.62 | | 537.1117 | [M-H]- | | C27H22O12 | | -1.14 | | 93.18 | 537.11,493.11,313.07,295.06,277.04,185.02,109.03 | Lithospermic acid or its isomer | Organic acid | 32 | 28831-65-4 |
| 290 | 13.62 | | 623.1713 | [M-H]- | | C_28_H_32_O_16_ | | 3.60 | | 95.93 | 623.17,315.05 | Narcissoside | Flavonoids | 62 | 604-80-8 |
| 291 | 13.64 | | 623.1688 | [M-H]- | | C28H32O16 | | 0.33 | | 77.35 | 623.16,313.04 | Isorhamnetin-3-O-neohespeidoside | Flavonoids | 95 | 55033-90-4 |
| 292 | 13.72 | | 515.1280 | [M-H]- | | C_25_H_24_O_12_ | | -2.32 | | 98.00 | 515.13,353.08,191.05 | 3,5-Di-O-caffeoylquinicacid or its isomer | Phenolics | 7,10,16,40,42,44,48,55,61,83,93,95 | 2450-53-5 |
| 293 | 13.74 | | 449.1178 | [M-H]- | | C_21_H_22_O_11_ | | -2.40 | | 93.08 | 449.11,303.05,151.00 | Neoisoastilbin or its isomer | Flavonoids | 16 | 54141-72-9 |
| 294 | 13.74 | | 625.1470 | [M-H]- | | C27H30O17 | | 2.07 | | 90.70 | 625.15,463.09,301.03 | Quercetin 3-O-gentiobioside or its isomer | Flavonoids | 67 | 7431-83-6 |
| 295 | 13.77 | | 417.0899 | [M-H]- | | C20H18O10 | | 0.17 | | 98.20 | 417.08,175.03 | Salvianolic acid D or its isomer | Phenolics | 32 | 142998-47-8 |
| 296 | 13.78 | | 259.0313 | [M-H]- | | C13H8O6 | | 2.88 | | 84.64 | 259.03,213.01,159.04 | 3,8,9,10-Tetrahydroxy urolithin | Phenolics | 35 | 1006683-97-1 |
| 297 | 13.80 | | 579.1807 | [M-H]- | | C_27_H_32_O_14_ | | -2.54 | | 95.50 | 579.18,459.11,313.07,271.06 | Naringin or its isomer | Flavonoids | 45,69,88,95 | 10236-47-2 |
| 298 | 13.80 | | 415.1111 | [M-H]- | | C_21_H_20_O_9_ | | -0.78 | | 83.35 | 415.11,295.06,267.06 | Puerarin or its isomer | Isoflavone | 46,52,81 | 3681-99-0 |
| 299 | 13.80 | | 353.0944 | [M-H]- | | C_16_H_18_O_9_ | | 1.96 | | 91.86 | 353.09,161.02 | Chlorogenic Acid or its isomer | Phenolics | 93 | 327-97-9 |
| 300 | 13.80 | | 477.1170 | [M-H]- | | C_22_H_22_O_12_ | | 2.07 | | 96.55 | 477.11,313.04 | Isorhamnetin -3-O-b-D-galactoside | Flavonoids | 62,74 | 6743-92-6 |
| 301 | 13.81 | | 565.1938 | [M+HCOO]- | | C_26_H_32_O_11_ | | -5.21 | | 82.28 | 519.19,357.13,151.04 | (+)-Pinoresinol-4-O-β-D-glucopyranoside | Lignans | 11 | 14907-98-3 |
| 302 | 13.81 | | 811.4591 | [M+HCOO]- | | C_41_H_66_O_13_ | | -4.05 | | 86.11 | 765.44,603.38 | Kudinoside H | Terpenoids | 18 | 35286-58-9 |
| 303 | 13.82 | | 447.0991 | [M-H]- | | C_21_H_20_O_11_ | | 3.29 | | 94.34 | 447.10,285.04 | Luteolin-7-O-β-D-glucoside or its isomer | Flavonoids | 42 | 5373-11-5 |
| 304 | 13.83 | | 1007.5542 | [M+HCOO]- | | C_48_H_82_O_19_ | | 0.65 | | 99.84 | 961.50,799.48 | Mogroside III or its isomer | Terpenoids | 18,27,74 | 130567-83-8 |
| 305 | 13.85 | | 577.1645 | [M-H]- | | C_27_H_30_O_14_ | | -1.65 | | 90.78 | 577.16,417.15 | Rhoifolin or its isomer | Flavonoids | 6,33 | 17306-46-6 |
| 306 | 13.89 | | 447.1006 | [M-H]- | | C21H20O11 | | 0.06 | | 96.70 | 447.10,301.03,179.00 | Quercitrin or its isomer | Flavonoids | 8,44,59,100 | 522-12-3 |
| 307 | 13.89 | | 961.5418 | [M+HCOO]- | | C_48_H_82_O_19_ | | -3.12 | | 90.24 | 915.54,637.43,305.08,263.07,221.06,179.05 | Mogroside IIIA1 | Terpenoids | 18 | 88901-42-2 |
| 308 | 13.89 | | 541.1652 | [M-H]- | | C_24_H_30_O_14_ | | -2.97 | | 94.96 | 541.15,379.10,347.07,309.06,277.07,195.06,169.01,125.02,81.03 | Cornuside | Terpenoids | 30 | 131189-57-6 |
| 309 | 13.91 | | 577.1657 | [M-H]- | | C_27_H_30_O_14_ | | 3.42 | | 87.60 | 577.16,413.08,293.04 | 2''-O-Rhamnosylvitexin or its isomer | Flavonoids | 10 | 64820-99-1 |
| 310 | 13.93 | | 433.1251 | [M-H]- | | C_21_H_22_O_10_ | | -8.78 | | 76.60 | 433.12,271.06,151.00,119.05 | Naringenin-7-O-β-D-glucoside or its isomer | Flavonoids | 28,45 | 529-55-5 |
| 311 | 13.95 | | 753.2327 | [M-H]- | | C34H42O19 | | 5.75 | | 79.83 | 753.23,547.16,367.10,205.05 | 3,6'-Disinapoyl sucrose | Phenylpropanoids | 56 | 139891-98-8 |
| 312 | 14.00 | | 607.1721 | [M-H]- | | C_28_H_32_O_15_ | | 3.32 | | 91.19 | 607.17,299.05 | Neodiosmin | Flavonoids | 11,28,42,52,61,95 | 38665-01-9 |
| 313 | 14.00 | | 431.1085 | [M-H]- | | C_21_H_20_O_10_ | | 2.91 | | 95.23 | 431.10,267.03 | Aloe-emodin-3-(hydroxymethyl)-O-β-D-glucopyranoside | Quinones | 61 | 29010-56-8 |
| 314 | 14.00 | | 431.1072 | [M-H]- | | C_21_H_20_O_10_ | | -2.15 | | 98.17 | 431.10,271.05 | Sophoricoside or its isomer | Flavonoids | 31,62 | 152-95-4 |
| 315 | 14.01 | | 431.1088 | [M-H]- | | C_21_H_20_O_10_ | | -4.16 | | 86.69 | 431.10,271.06 | Emodin-8-glucoside | Anthraquinone | 43 | 23313-21-5 |
| 316 | 14.02 | | 609.1911 | [M-H]- | | C_28_H_34_O_15_ | | -2.11 | | 96.98 | 609.18,325.07,301.07,285.04 | Hesperidin | Flavonoids | 34,45,52,73 | 520-26-3 |
| 317 | 14.04 | | 433.1228 | [M-H]- | | C_21_H_22_O_10_ | | 3.35 | | 92.10 | 433.12,269.04,181.01 | Engeletin or its isomer | Flavonoids | 16 | 572-31-6 |
| 318 | 14.07 | | 515.1265 | [M-H]- | | C_25_H_24_O_12_ | | 0.56 | | 95.97 | 515.11,353.08,191.05,173.04,135.04,111.04,93.03,71.01 | 4,5-Dicaffeoylquinic acid or its isomer | Phenolics | 6,16,29,33,44,52,53,55,58,61,79,83,93,95,98 | 57378-72-0 |
| 319 | 14.07 | | 515.1262 | [M-H]- | | C25H24O12 | | 1.13 | | 94.72 | 515.12,353.08,179.03 | 1,3-O-Dicaffeoylquinic acid or its isomer | Phenolics | 42,84,93,95 | 19870-46-3 |
| 320 | 14.08 | | 325.0931 | [M-H]- | | C_15_H_18_O_8_ | | 1.80 | | 94.63 | 325.09,119.05 | (Z)-p-Coumaric acid 4-O-β-D-glucopyranside or its isomer | Coumarins | 31 | 117405-48-8 |
| 321 | 14.15 | | 525.1653 | [M+HCOO]- | | C23H28O11 | | -4.44 | | 89.83 | 479.15,165.05,121.02,77.03 | Paeoniflorin or its isomer | Terpenoids | 23,35,79 | 23180-57-6 |
| 322 | 14.16 | | 717.1545 | [M-H]- | | C36H30O16 | | -1.55 | | 81.37 | 717.14,537.10,519.09,493.11,339.05,321.04,295.06,249.05,185.02,109.02,71.99 | Salvianolic acid B or its isomer | Phenolics | 32 | 121521-90-2 |
| 323 | 14.20 | | 717.1552 | [M-H]- | | C_36_H_30_O_16_ | | -2.52 | | 81.62 | 717.15,537.08,519.09,339.05,321.03 | Salvianolic acid E | Phenolics | 26 | 142998-46-7 |
| 324 | 14.27 | | 515.1300 | [M-H]- | | C_25_H_24_O_12_ | | -3.12 | | 85.00 | 515.12,353.08,179.03,135.04 | 1,4-Dicaffeoylquinic acid or its isomer | Phenylpropanoids | 4 | 1182-34-9 |
| 325 | 14.29 | | 525.1636 | [M+HCOO]- | | C23H28O11 | | -0.88 | | 90.20 | 479.16,357.11,317.10,283.08,121.02,77.03 | Albiflorin or its isomer | Terpenoids | 35 | 39011-90-0 |
| 326 | 14.29 | | 193.0592 | [M-H]- | | C_10_H_10_O_4_ | | 6.56 | | 79.61 | 193.05,177.02,149.06,133.03 | Ferulic acid or its isomer | Phenylpropanoids | 50,80 | 1135-24-6 |
| 327 | 14.38 | | 359.0846 | [M-H]- | | C_18_H_16_O_8_ | | -0.28 | | 97.01 | 359.08,197.04,179.03,161.02,135.04,123.04,71.99 | Rosmarinic Acid or its isomer | Phenolics | 4,11,19,20,23,26,28,41,52,54,56,61,62,69,71,88 | 20283-92-5 |
| 328 | 14.40 | | 1007.5491 | [M+HCOO]- | | C_48_H_82_O_19_ | | 0.46 | | 99.47 | 961.50,799.48 | Mogroside III or its isomer | Terpenoids | 18,27,74 | 130567-83-8 |
| 329 | 14.41 | | 331.1983 | [M-H]- | | C_20_H_28_O_4_ | | 1.40 | | 90.93 | 331.19,303.19 | Carnosic acid or its isomer | Terpenoids | 55 | 3650-09-7 |
| 330 | 14.41 | | 513.1507 | [M-H]- | | C_25_H_24_O_12_ | | 2.61 | | 90.38 | 513.15,351.08 | 3,5-Di-O-caffeoylquinicacid | Phenylpropanoids | 8 | 2450-53-5 |
| 331 | 14.42 | | 783.2823 | [M-H]- | | C_36_H_48_O_19_ | | 3.21 | | 90.47 | 783.28,607.22,461.16,175.03 | Angoroside C | Organic acid | 19 | 115909-22-3 |
| 332 | 14.42 | | 609.1923 | [M-H]- | | C_28_H_34_O_15_ | | -4.21 | | 93.52 | 609.18,489.14,403.10,343.08 | Neohesperidin | Alkaloid | 45 | 13241-33-3 |
| 333 | 14.46 | | 283.0320 | [M-H]- | | C_15_H_8_O_6_ | | 0.22 | | 89.47 | 283.03,239.03,211.04,183.04,167.05,155.05,139.05,127.05 | Rhein or its isomer | Anthraquinone | 93 | 478-43-3 |
| 334 | 14.48 | | 507.1177 | [M+HCOO]- | | C_22_H_22_O_11_ | | -3.21 | | 98.13 | 461.11,299.05 | Diosmetin-7-O-β-D-glucopyranoside or its isomer | Flavonoids | 61 | 20126-59-4 |
| 335 | 14.50 | | 461.0850 | [M-H]- | | C_21_H_18_O_12_ | | -1.56 | | 97.99 | 461.07,285.04,113.02 | Luteolin-3-D-glucuronide or its isomer | Flavonoids | 4 | 53527-42-7 |
| 336 | 14.52 | | 269.0543 | [M-H]- | | C_15_H_10_O_5_ | | -5.43 | | 90.55 | 269.05,241.05,225.05,197.06,181.06 | Emodin or its isomer | Anthraquinone | 13 | 518-82-1 |
| 337 | 14.52 | | 433.1259 | [M-H]- | | C_21_H_22_O_10_ | | -3.87 | | 83.93 | 433.12,269.04,181.01 | Engeletin or its isomer | Flavonoids | 16 | 572-31-6 |
| 338 | 14.53 | | 519.1988 | [M-H]- | | C_26_H_32_O_11_ | | 3.08 | | 95.78 | 519.19,357.13 | Matairesinoside or its isomer | Lignans | 54 | 23202-85-9 |
| 339 | 14.56 | | 493.1221 | [M-H]- | | C_26_H_22_O_10_ | | -1.65 | | 92.82 | 493.12,313.07,295.06,277.05,185.02,159.04,135.04,109.02 | Salvianolic acid A or its isomer | Phenolics | 19,23,32,69,88 | 96574-01-5 |
| 340 | 14.57 | | 977.5369 | [M+HCOO]- | | C_47_H_80_O_18_ | | -1.12 | | 97.45 | 931.52,799.48,637.43,89.02 | Notoginsenoside R1 | Terpenoids | 18,27,58,75,81,84 | 80418-24-2 |
| 341 | 14.58 | | 537.1113 | [M-H]- | | C27H22O12 | | -0.28 | | 81.47 | 537.11,493.11,313.07,295.06,277.04,185.02,109.03 | Lithospermic acid or its isomer | Organic acid | 32 | 28831-65-4 |
| 342 | 14.58 | | 435.1358 | [M-H]- | | C_21_H_24_O_10_ | | 4.92 | | 87.30 | 435.13,273.07,167.03 | Phloridzin | Flavonoids | 3 | 60-81-1 |
| 343 | 14.61 | | 463.0963 | [M-H]- | | C21H20O12 | | -1.87 | | 95.78 | 463.09,301.03 | Quercetin-7-O-β-D-glucopyranoside or its isomer | Flavonoids | 67 | 491-50-9 |
| 344 | 14.63 | | 345.0707 | [M-H]- | | C_17_H_14_O_8_ | | 2.67 | | 99.15 | 345.06,329.03,315.01 | Ganhuangenin or its isomer | Flavonoids | 49 | 92519-91-0 |
| 345 | 14.65 | | 315.0594 | [M-H]- | | C_16_H_12_O_7_ | | -5.34 | | 84.52 | 315.05,149.00 | Isorhamnetin or its isomer | Flavonoids | 10 | 480-19-3 |
| 346 | 14.65 | | 515.1302 | [M-H]- | | C_25_H_24_O_12_ | | -5.23 | | 87.41 | 515.12,353.08,179.03 | 1,3-O-Dicaffeoylquinic acid or its isomer | Phenolics | 7 | 19870-46-3 |
| 347 | 14.65 | | 431.1055 | [M-H]- | | C_21_H_20_O_10_ | | 1.78 | | 91.13 | 431.10,269.04 | Genistin or its isomer | Flavonoids | 92 | 529-59-9 |
| 348 | 14.67 | | 577.1669 | [M-H]- | | C_27_H_30_O_14_ | | -0.74 | | 98.41 | 577.16,253.05 | Chrysin 7-O-β-gentiobioside | Flavonoids | 41 | 88640-89-5 |
| 349 | 14.69 | | 431.1064 | [M-H]- | | C_21_H_20_O_10_ | | -1.81 | | 85.72 | 431.10,271.06 | Emodin-8-glucoside | Anthraquinone | 81 | 23313-21-5 |
| 350 | 14.69 | | 807.2795 | [M-H]- | | C_38_H_48_O_19_ | | -3.95 | | 93.39 | 807.27,515.12,191.05,173.04,161.02 | Baohuoside V | Flavonoids | 29 | 118544-18-6 |
| 351 | 14.69 | | 461.1163 | [M-H]- | | C_22_H_22_O_11_ | | -0.22 | | 96.21 | 461.11,313.05,271.04,169.01,147.04 | 2-O-Cinnamoyl-1-O-galloyl-β-D-glucose | Phenolics | 82,93 | 791836-69-6 |
| 352 | 14.71 | | 431.1044 | [M-H]- | | C_21_H_20_O_10_ | | 2.84 | | 96.73 | 431.09,269.04 | Oroxin A or its isomer | Flavonoids | 93 | 57396-78-8 |
| 353 | 14.73 | | 661.2174 | [M-H]- | | C_32_H_38_O_15_ | | 5.60 | | 85.11 | 661.21,515.15,353.09 | Epimedoside A | Flavonoids | 12,29 | 39012-04-9 |
| 354 | 14.73 | | 549.1672 | [M-H]- | | C_26_H_30_O_13_ | | -2.17 | | 92.14 | 549.16,255.06 | Isoliquiritin apioside or its isomer | Flavonoids | 1,8,9,11,17,28,40,47,56,61,63,72,77,86,92 | 120926-46-7 |
| 355 | 14.79 | | 431.1081 | [M-H]- | | C_21_H_20_O_10_ | | 4.95 | | 80.00 | 431.10,285.04,151.00 | Afzelin | Phenolics | 8,54 | 482-39-3 |
| 356 | 14.88 | | 447.1023 | [M-H]- | | C21H20O11 | | -3.94 | | 77.26 | 447.10,301.03,151.00 | Quercetin 7-rhamnoside | Flavonoids | 83 | 22007-72-3 |
| 357 | 14.92 | | 463.1005 | [M-H]- | | C_21_H_20_O_12_ | | -3.82 | | 82.82 | 463.09,301.03 | Hyperoside or its isomer | Flavonoids | 54 | 482-36-0 |
| 358 | 14.95 | | 725.2219 | [M-H]- | | C_30_H_46_O_16_S_2_ | | -1.17 | | 98.53 | 725.22,645.15 | Atractyloside | Terpenoids | 4,48 | 1398-13-6 |
| 359 | 14.96 | | 445.0861 | [M-H]- | | C21H18O11 | | -2.68 | | 93.16 | 445.07,269.06,175.02,113.02.85.02,59.01 | Glychionide or its isomer | Flavonoids | 2,6,11,33,52,56,81,90,94 | 119152-50-0 |
| 360 | 14.97 | | 461.0827 | [M-H]- | | C_21_H_18_O_12_ | | -6.29 | | 92.01 | 461.07,285.04,113.02 | Luteolin-3-D-glucuronide or its isomer | Flavonoids | 55 | 53527-42-7 |
| 361 | 14.98 | | 549.1675 | [M-H]- | | C26H30O13 | | 2.03 | | 89.31 | 549.16,255.06 | Liguirtigenin-7-O-D-apiosyl-4'-O-D-Glucoside | Flavonoids | 73 | 19979-12-8 |
| 362 | 14.99 | | 845.4931 | [M+HCOO]- | | C_42_H_72_O_14_ | | 1.32 | | 97.69 | 799.48,637.42,475.37,161.04,89.02 | Ginsenoside Rg1 or its isomer | Terpenoids | 18,26,27,29,58,75,76,81,84,93,95 | 22427-39-0 |
| 363 | 14.99 | | 445.0879 | [M-H]- | | C_21_H_18_O_11_ | | 0.87 | | 91.37 | 445.08,269.04,113.02 | Apigenin 7-O-beta-D-glucuronide | Flavonoids | 65 | 29741-09-1 |
| 364 | 14.99 | | 461.1171 | [M-H]- | | C_22_H_22_O_11_ | | -1.93 | | 90.72 | 461.11,313.05,271.04 | Isoscoparin or its isomer | Flavonoids | 82,93 | 20013-23-4 |
| 365 | 15.00 | | 992.5515 | [M+HCOO]- | | C_48_H_82_O_18_ | | 2.09 | | 85.43 | 946.55,842.24 | Ginsenoside Re or its isomer | Terpenoids | 25,27,58,75 | 52286-59-6 |
| 366 | 15.01 | | 845.5079 | [M+HCOO]- | | C_42_H_72_O_14_ | | -3.16 | | 87.00 | 799.50,637.43 | Mogroside ⅡA1 | Terpenoids | 20,74 | 88901-44-4 |
| 367 | 15.02 | | 417.1280 | [M-H]- | | C_21_H_22_O_9_ | | -3.79 | | 84.08 | 417.12,255.06 | Isoliquiritin or its isomer | Chalcones | 8,9,17,36,44,56,73,86,95 | 5041-81-6 |
| 368 | 15.03 | | 269.0537 | [M-H]- | | C_15_H_10_O_5_ | | -3.26 | | 85.95 | 269.05,241.04 | Aloeemodin or its isomer | Anthraquinone | 6,10 | 481-72-1 |
| 369 | 15.03 | | 417.1275 | [M-H]- | | C_21_H_22_O_9_ | | -2.77 | | 85.33 | 417.12,257.06,135.00 | Liquiritin or its isomer | Flavonoids | 1,11,69,88 | 551-15-5 |
| 370 | 15.05 | | 445.0857 | [M-H]- | | C_21_H_18_O_11_ | | 1.70 | | 98.03 | 445.07,325.03,307.02,283.02,267.02,239.03,165.04 | Rhein-8-O-β-D-glucopyranoside | Anthraquinone | 46 | 34298-86-7 |
| 371 | 15.07 | | 285.0493 | [M-H]- | | C_15_H_10_O_6_ | | 5.39 | | 79.16 | 285.04,257.04,213.05 | Kaempferol or its isomer | Flavonoids | 54 | 520-18-3 |
| 372 | 15.09 | | 345.0704 | [M-H]- | | C_17_H_14_O_8_ | | -4.31 | | 83.42 | 345.06,329.03,315.01 | Ganhuangenin or its isomer | Flavonoids | 2,7,11,46,49,50,51 | 92519-91-0 |
| 373 | 15.09 | | 269.0542 | [M-H]- | | C_15_H_10_O_5_ | | -5.27 | | 90.63 | 269.04,241.03,223.04,197.06,169.06,135.98 | Baicalein or its isomer | Flavonoids | 52,100 | 491-67-8 |
| 374 | 15.09 | | 717.1549 | [M-H]- | | C_36_H_30_O_16_ | | -2.15 | | 98.33 | 717.14,537.10,519.09,493.11,339.05,321.04,295.06,249.05,185.02,109.02,71.99 | Salvianolic acid B or its isomer | Phenolics | 23,69,88 | 121521-90-2 |
| 375 | 15.09 | | 121.0361 | [M-H]- | | C_7_H_6_O_2_ | | -5.70 | | 87.34 | 121.03,93.02,65.04 | p-Hydroxybenzaldehyde or its isomer | Phenolics | 9 | 123-08-0 |
| 376 | 15.12 | | 599.1819 | [M-H]- | | C_30_H_32_O_13_ | | 3.93 | | 84.25 | 599.18,477.13,333.10,137.02 | Benzoyloxypaeoniflorin or its isomer | Terpenoids | 30 | 72896-40-3 |
| 377 | 15.20 | | 515.1295 | [M-H]- | | C_25_H_24_O_12_ | | -2.02 | | 90.04 | 515.11,353.08,191.05,173.04,135.04,111.04,93.03,71.01 | 4,5-Dicaffeoylquinic acid or its isomer | Phenolics | 61 | 57378-72-0 |
| 378 | 15.23 | | 515.1250 | [M-H]- | | C_25_H_24_O_12_ | | 3.41 | | 89.32 | 515.12,353.08,179.03 | 1,3-O-Dicaffeoylquinic acid or its isomer | Phenolics | 93 | 19870-46-3 |
| 379 | 15.24 | | 463.0957 | [M-H]- | | C_21_H_20_O_12_ | | -0.40 | | 95.30 | 463.09,343.04,299.02,271.02,177.99 | Isoquercitrin or its isomer | Flavonoids | 41 | 21637-25-2 |
| 380 | 15.27 | | 493.1228 | [M-H]- | | C26H22O10 | | -2.96 | | 94.39 | 493.12,313.07,295.06,277.05,185.02,159.04,135.04,109.02 | Salvianolic acid A or its isomer | Phenolics | 32,72 | 96574-01-5 |
| 381 | 15.30 | | 417.1300 | [M-H]- | | C_21_H_22_O_9_ | | -3.81 | | 88.89 | 417.13,255.06 | Pinocembrin 7-O-bate-D-glucoside | Flavonoids | 9,63 | 75829-43-5 |
| 382 | 15.33 | | 717.1508 | [M-H]- | | C_36_H_30_O_16_ | | -2.19 | | 93.55 | 717.15,537.08,519.09,339.05,321.03 | Salvianolic acid E | Phenolics | 23,25,56 | 142998-46-7 |
| 383 | 15.34 | | 301.0427 | [M-H]- | | C_15_H_10_O_7_ | | -0.20 | | 92.21 | 301.04,151.00 | Morin | Flavonoids | 93 | 480-16-0 |
| 384 | 15.35 | | 275.1046 | [M-H]- | | C_15_H_16_O_5_ | | -2.96 | | 87.75 | 275.09,257.08,217.05 | Hamaudol or its isomer | Flavonoids | 65 | 735-46-6 |
| 385 | 15.41 | | 579.2143 | [M+HCOO]- | | C_27_H_34_O_11_ | | -2.10 | | 91.51 | 533.21,371.15,83.01 | Arctiin | Lignans | 80 | 20362-31-6 |
| 386 | 15.42 | | 461.1168 | [M-H]- | | C_22_H_22_O_11_ | | -1.28 | | 87.15 | 461.11,313.05,271.04 | Isoscoparin or its isomer | Flavonoids | 3 | 20013-23-4 |
| 387 | 15.42 | | 312.1331 | [M-H]- | | C_18_H_19_NO_4_ | | -5.27 | | 86.32 | 312.12,297.10,282.07 | Norisoboldine or its isomer | Alkaloid | 29,66,81 | 23599-69-1 |
| 388 | 15.42 | | 253.0581 | [M-H]- | | C_15_H_10_O_4_ | | -0.68 | | 96.66 | 253.05,225.04,209.06,197.06,135.00 | Daidzein or its isomer | Isoflavone | 21,22,45,57,64 | 486-66-8 |
| 389 | 15.45 | | 253.0586 | [M-H]- | | C_15_H_10_O_4_ | | -2.78 | | 90.01 | 253.05,225.05,197.06 | Chrysophanol or its isomer | Anthraquinone | 85 | 481-74-3 |
| 390 | 15.46 | | 285.0845 | [M-H]- | | C_16_H_14_O_5_ | | 1.15 | | 85.58 | 285.08,163.04 | Brazilin | Flavonoids | 47 | 474-07-7 |
| 391 | 15.47 | | 285.0878 | [M-H]- | | C_16_H_14_O_5_ | | -1.73 | | 90.13 | 285.08,149.03 | Licochalcone B | Chalcones | 1,17,40,77,92 | 58749-23-8 |
| 392 | 15.51 | | 271.0693 | [M-H]- | | C_15_H_12_O_5_ | | -3.03 | | 92.16 | 271.06,253.05 | Pinobanksin | Flavonoids | 52 | 548-82-3 |
| 393 | 15.54 | | 445.0861 | [M-H]- | | C_21_H_18_O_11_ | | 2.66 | | 97.74 | 445.07,269.06,175.02,113.02.85.02,59.01 | Glychionide or its isomer | Flavonoids | 3,4,5,10,11,33,47,49,52,81,92 | 119152-50-0 |
| 394 | 15.55 | | 285.0482 | [M-H]- | | C_15_H_10_O_6_ | | -1.60 | | 74.97 | 285.03,257.04,213.05 | Kaempferol or its isomer | Flavonoids | 55,57 | 520-18-3 |
| 395 | 15.59 | | 599.1888 | [M-H]- | | C_30_H_32_O_13_ | | -7.55 | | 84.79 | 599.18,477.13,333.10,137.02 | Benzoyloxypaeoniflorin or its isomer | Terpenoids | 30 | 72896-40-3 |
| 396 | 15.62 | | 255.0747 | [M-H]- | | C_15_H_12_O_4_ | | 4.31 | | 96.40 | 255.06,213.05,135.00,119.05 | Isoliquiritigenin or its isomer | Chalcones | 1,5,8,11,16,17,28,36,44,47,56,61,63,69,73,86,88,92 | 961-29-5 |
| 397 | 15.62 | | 514.2942 | [M-H]- | | C_26_H_45_NO_7_S | | -4.95 | | 88.21 | 514.28,392.98,124.00 | Taurocholic acid or its isomer | Steroids | 45 | 81-24-3 |
| 398 | 15.64 | | 845.4973 | [M+HCOO]- | | C_42_H_72_O_14_ | | -0.21 | | 93.05 | 799.48,637.42,475.37,161.04,89.02 | Ginsenoside Rg1 or its isomer | Terpenoids | 18 | 22427-39-0 |
| 399 | 15.65 | | 1071.3652 | [M-H]- | | C_48_H_64_O_27_ | | 2.10 | | 91.28 | 1071.36,909.30,685.23,523.18,403.12,223.06 | Ligustroflavone G13 or its isomer | Terpenoids | 79 | 60037-39-0 |
| 400 | 15.65 | | 253.0599 | [M-H]- | | C_15_H_10_O_4_ | | 2.01 | | 100.00 | 253.05,135.01 | 3′,4′-Dihydroxyflavone | Flavonoids | 68 | 4143-64-0 |
| 401 | 15.66 | | 461.1147 | [M-H]- | | C_22_H_22_O_11_ | | 3.23 | | 89.73 | 461.11,313.05,271.04 | Isoscoparin or its isomer | Flavonoids | 8,82,93 | 20013-23-4 |
| 402 | 15.66 | | 273.0489 | [M-H]- | | C_14_H_10_O_6_ | | 4.18 | | 84.53 | 273.04,257.01 | Bellidifolin | Phenolics | 7 | 2798-25-6 |
| 403 | 15.66 | | 325.1009 | [M-H]- | | C_15_H_18_O_8_ | | 2.18 | | 83.95 | 325.09,119.05 | (Z)-p-Coumaric acid 4-O-β-D-glucopyranside or its isomer | Coumarins | 31 | 117405-48-8 |
| 404 | 15.68 | | 395.2147 | [M+HCOO]- | | C_20_H_30_O_5_ | | 3.27 | | 87.25 | 349.21,331.19,287.20 | Andrographolide | Terpenoids | 55,98 | 5508-58-7 |
| 405 | 15.70 | | 201.0278 | [M-H]- | | C_11_H_6_O_4_ | | 5.65 | | 88.68 | 201.02,173.02,145.02,117.03 | Bergaptol or its isomer | Coumarins | 42,52,95 | 486-60-2 |
| 406 | 15.71 | | 637.1798 | [M+HCOO]- | | C_28_H_32_O_14_ | | -1.72 | | 90.49 | 591.18,283.06 | Buddleoside | Flavonoids | 11,61 | 480-36-4 |
| 407 | 15.72 | | 283.0693 | [M-H]- | | C_16_H_12_O_5_ | | 2.94 | | 94.29 | 283.06,119.05 | Biochanin A or its isomer | Flavonoids | 71,91 | 491-80-5 |
| 408 | 15.73 | | 717.1530 | [M-H]- | | C36H30O16 | | 0.49 | | 82.34 | 717.14,537.10,519.09,493.11,339.05,321.04,295.06,249.05,185.02,109.02,71.99 | Salvianolic acid B or its isomer | Phenolics | 32 | 121521-90-2 |
| 409 | 15.76 | | 651.2399 | [M-H]- | | C_31_H_40_O_15_ | | -2.19 | | 85.23 | 651.23,475.18,175.04 | Epimeredinoside A | Phenylethanoid glycosides | 9 | 848599-79-1 |
| 410 | 15.79 | | 493.1204 | [M-H]- | | C_26_H_22_O_10_ | | 1.90 | | 96.18 | 493.12,313.07,295.06,277.05,185.02,159.04,135.04,109.02 | Salvianolic acid A or its isomer | Phenolics | 32,69,88 | 96574-01-5 |
| 411 | 15.79 | | 283.0691 | [M-H]- | | C_16_H_12_O_5_ | | -2.35 | | 89.04 | 283.06,267.05 | 7-O-Methylbaicalein or its isomer | Flavonoids | 21,24,57 | 29550-13-8 |
| 412 | 15.80 | | 299.0655 | [M-H]- | | C_16_H_12_O_6_ | | -2.06 | | 91.14 | 299.06,283.03,255.03 | 3'-Methoxyapigenin or its isomer | Flavonoids | 49 | 491-71-4 |
| 413 | 15.80 | | 287.0615 | [M-H]- | | C_15_H_12_O_6_ | | 6.67 | | 80.46 | 287.05,151.00,135.04 | Eriodictyol | Flavonoids | 93 | 552-58-9 |
| 414 | 15.82 | | 675.2394 | [M-H]- | | C33H40O15 | | -3.95 | | 90.47 | 675.22,367.12 | Baohuoside VII or its isomer | Flavonoids | 12 | 119730-89-1 |
| 415 | 15.82 | | 445.0870 | [M-H]- | | C_21_H_18_O_11_ | | 0.95 | | 98.25 | 445.07,269.06,175.02,113.02.85.02,59.01 | Glychionide or its isomer | Flavonoids | 5,11,47,48,49,51,52,53,63,81,100 | 119152-50-0 |
| 416 | 15.85 | | 299.0635 | [M-H]- | | C_16_H_12_O_6_ | | 0.43 | | 97.76 | 299.06,285.03 | Kaempferide or its isomer | Flavonoids | 17 | 491-54-3 |
| 417 | 15.87 | | 403.1451 | [M-H]- | | C_21_H_24_O_8_ | | 5.10 | | 89.28 | 403.13,195.06 | Albaspidin AA | Phenolics | 66 | 3570-40-9 |
| 418 | 15.89 | | 1093.5503 | [M+HCOO]- | | C_51_H_84_O_22_ | | -2.93 | | 92.16 | 1047.55,901.47 | Protodioscin | Terpenoids | 75 | 55056-80-9 |
| 419 | 15.89 | | 431.1057 | [M-H]- | | C_21_H_20_O_10_ | | 3.01 | | 82.58 | 431.10,269.04 | Genistin or its isomer | Flavonoids | 10 | 529-59-9 |
| 420 | 15.89 | | 815.4799 | [M+HCOO]- | | C_41_H_70_O_13_ | | -2.82 | | 96.64 | 769.47,637.43,149.04 | Ginsenoside F5 or its isomer | Terpenoids | 18,27 | 189513-26-6 |
| 421 | 15.90 | | 683.1862 | [M+HCOO]- | | C_29_H_34_O_16_ | | 2.41 | | 98.47 | 637.18,329.06 | Ombuoside | Flavonoids | 62 | 20188-85-6 |
| 422 | 15.90 | | 461.1176 | [M-H]- | | C_22_H_22_O_11_ | | 4.10 | | 93.21 | 461.11,299.05 | Diosmetin-7-O-β-D-glucopyranoside or its isomer | Flavonoids | 51 | 20126-59-4 |
| 423 | 15.94 | | 285.0480 | [M-H]- | | C_15_H_10_O_6_ | | -1.08 | | 93.35 | 285.03,257.04,213.05 | Kaempferol or its isomer | Flavonoids | 6,33,81,93 | 520-18-3 |
| 424 | 15.95 | | 431.1102 | [M-H]- | | C_21_H_20_O_10_ | | 2.01 | | 99.24 | 431.10,271.05 | Sophoricoside or its isomer | Flavonoids | 4,48 | 152-95-4 |
| 425 | 15.95 | | 177.0628 | [M-H]- | | C_10_H_10_O_3_ | | 1.31 | | 92.46 | 177.06,151.03,135.04 | 3,4-Dihydroxybenzalacetone or its isomer | Phenolics | 57 | 37079-84-8 |
| 426 | 15.96 | | 1071.3701 | [M-H]- | | C_48_H_64_O_27_ | | 5.01 | | 84.40 | 1071.36,909.30,685.23,523.18,403.12,223.06 | Ligustroflavone G13 or its isomer | Terpenoids | 79 | 60037-39-0 |
| 427 | 16.00 | | 459.1017 | [M-H]- | | C_22_H_20_O_11_ | | -2.50 | | 93.87 | 459.09,283.06,268.03,175.02,113.02,99.00,85.02,59.01 | Wogonin 7-O-glucuronide or its isomer | Flavonoids | 2,6,32,33,52,63,81,94 | 51059-44-0 |
| 428 | 16.00 | | 417.1276 | [M-H]- | | C_21_H_22_O_9_ | | -2.88 | | 92.64 | 417.12,255.06 | Isoliquiritin or its isomer | Chalcones | 93 | 5041-81-6 |
| 429 | 16.03 | | 283.0701 | [M-H]- | | C_16_H_12_O_5_ | | 5.70 | | 97.86 | 283.07,267.04 | Genkwanin or its isomer | Flavonoids | 4,71,72,76,79 | 437-64-9 |
| 430 | 16.04 | | 285.0481 | [M-H]- | | C_15_H_10_O_6_ | | -1.19 | | 97.40 | 285.04,241.05,217.04,199.04,175.04,151.00,133.02 | Luteolin | Flavonoids | 55 | 491-70-3 |
| 431 | 16.05 | | 275.1046 | [M-H]- | | C_15_H_16_O_5_ | | 2.01 | | 87.75 | 275.09,257.08,217.05 | Hamaudol or its isomer | Flavonoids | 23,65 | 735-46-6 |
| 432 | 16.05 | | 407.1429 | [M-H]- | | C_20_H_24_O_9_ | | 0.29 | | 98.69 | 407.13,245.08,230.05 | Torachrysone-8-O-β-D-glucopyranoside | Phenolics | 2,9,42,82,93 | 64032-49-1 |
| 433 | 16.06 | | 301.0428 | [M-H]- | | C_15_H_10_O_7_ | | 0.53 | | 91.29 | 301.03,151.00 | Morin | Flavonoids | 8,44 | 480-16-0 |
| 434 | 16.08 | | 475.0959 | [M-H]- | | C_22_H_20_O_12_ | | -0.93 | | 91.58 | 475.08,285.04 | Scutellarin methylester | Flavonoids | 2,52,81 | 119262-68-9 |
| 435 | 16.11 | | 301.0435 | [M-H]- | | C15H10O7 | | -2.92 | | 90.40 | 301.03,179.00,151.00,121.03 | Quercetin | Flavonoids | 67,95 | 117-39-5 |
| 436 | 16.12 | | 493.1223 | [M-H]- | | C26H22O10 | | -1.95 | | 95.45 | 493.12,313.07,295.06,277.05,185.02,159.04,135.04,109.02 | Salvianolic acid A or its isomer | Phenolics | 32 | 96574-01-5 |
| 437 | 16.16 | | 165.0629 | [M-H]- | | C_9_H_10_O_3_ | | 0.62 | | 97.12 | 165.05,137.02,92.02 | Ethyl 4-hydroxybenzoate | Esters | 12,18,75,90,97 | 120-47-8 |
| 438 | 16.17 | | 137.0320 | [M-H]- | | C_7_H_6_O_3_ | | 2.44 | | 90.95 | 137.02,93.03 | 4-Hydroxybenzoic acid or its isomer | Phenolics | 100 | 99-67-7 |
| 439 | 16.18 | | 137.0322 | [M-H]- | | C_7_H_6_O_3_ | | -3.57 | | 93.84 | 137.03,93.03 | Salicylic Acid or its isomer | Organic acid | 90 | 69-72-7 |
| 440 | 16.18 | | 253.0577 | [M-H]- | | C_15_H_10_O_4_ | | -1.01 | | 88.24 | 253.05,225.05,197.06 | Chrysophanol or its isomer | Anthraquinone | 2,8,81,82,92,93 | 481-74-3 |
| 441 | 16.18 | | 659.2437 | [M-H]- | | C_33_H_40_O_14_ | | -2.88 | | 95.34 | 659.23,365.11 | 2''-O-Rhamnosylicariside II or its isomer | Flavonoids | 12,29,89 | 135293-13-9 |
| 442 | 16.18 | | 815.4799 | [M+HCOO]- | | C_41_H_70_O_13_ | | 2.09 | | 96.58 | 769.47,637.43,149.04 | Ginsenoside F5 or its isomer | Terpenoids | 18,27 | 189513-26-6 |
| 443 | 16.19 | | 445.0846 | [M-H]- | | C_21_H_18_O_11_ | | -3.12 | | 95.52 | 445.07,269.06,175.02,113.02.85.02,59.01 | Glychionide or its isomer | Flavonoids | 1,2,5,11,52,81 | 119152-50-0 |
| 444 | 16.20 | | 431.1071 | [M-H]- | | C_21_H_20_O_10_ | | -3.32 | | 97.38 | 445.07,269.06,175.02,113.02.85.02,59.01 | Glychionide or its isomer | Flavonoids | 12,42,81,82,93 | 57396-78-8 |
| 445 | 16.20 | | 943.4971 | [M+HCOO]- | | C_46_H_74_O_17_ | | 1.03 | | 97.39 | 897.49,765.46 | Bacopasaponin C | Saponin | 96 | 178064-13-6 |
| 446 | 16.23 | | 431.1065 | [M-H]- | | C_21_H_20_O_10_ | | 4.10 | | 91.09 | 431.10,269.04 | Genistin or its isomer | Flavonoids | 16,92 | 529-59-9 |
| 447 | 16.25 | | 283.0688 | [M-H]- | | C_16_H_12_O_5_ | | 1.18 | | 94.88 | 283.06,267.03,243.01 | Acacetin | Flavonoids | 68 | 480-44-4 |
| 448 | 16.25 | | 253.0594 | [M-H]- | | C_15_H_10_O_4_ | | 5.82 | | 76.67 | 253.05,225.04,209.06,197.06,135.00 | Daidzein or its isomer | Isoflavone | 63 | 486-66-8 |
| 449 | 16.27 | | 415.1116 | [M-H]- | | C_21_H_20_O_9_ | | 2.10 | | 93.63 | 415.10,277.05,253.05,239.07 | Chrysophanol-8-O-β-D-glucopyranoside | Anthraquinone | 2,8,82,92,93 | 13241-28-6 |
| 450 | 16.31 | | 357.1097 | [M-H]- | | C_19_H_18_O_7_ | | 3.12 | | 95.50 | 358.10,342.07,327.05 | Chrysoobtusin or its isomer | Quinones | 41 | 70588-06-6 |
| 451 | 16.33 | | 299.0611 | [M-H]- | | C_16_H_12_O_6_ | | 2.01 | | 96.44 | 299.06,283.03,255.03 | 3'-Methoxyapigenin or its isomer | Flavonoids | 7 | 491-71-4 |
| 452 | 16.34 | | 459.1024 | [M-H]- | | C_22_H_20_O_11_ | | -4.02 | | 93.78 | 459.09,283.06,268.03,175.02,113.02,99.00,85.02,59.01 | Wogonin 7-O-glucuronide or its isomer | Flavonoids | 2,6,10,32,33,48,50,51,52,81,83,94,100 | 51059-44-0 |
| 453 | 16.34 | | 498.2980 | [M-H]- | | C26H45NO6S | | -2.5 | | 93.46 | 498.29,124.00 | Tauroursodeoxycholic acid or its isomer | Steroids | 83 | 14605-22-2 |
| 454 | 16.37 | | 283.0684 | [M-H]- | | C_16_H_12_O_5_ | | -0.09 | | 97.82 | 283.07,267.04 | Genkwanin or its isomer | Flavonoids | 5,7,10,41,46,47,48,49,50,51,53 | 437-64-9 |
| 455 | 16.39 | | 721.2392 | [M+HCOO]- | | C33H40O15 | | -3.72 | | 93.88 | 675.23,513.17,409.13,367.11 | Icariin | Flavonoids | 12 | 489-32-7 |
| 456 | 16.40 | | 283.0680 | [M-H]- | | C_16_H_12_O_5_ | | -1.74 | | 93.21 | 283.06,267.05 | 7-O-Methylbaicalein or its isomer | Flavonoids | 1,52 | 29550-13-8 |
| 457 | 16.41 | | 513.1856 | [M-H]- | | C27H30O10 | | -3.22 | | 92.05 | 513.17,365.11,351.08,323.09 | Baohuoside I or its isomer | Flavonoids | 12 | 113558-15-9 |
| 458 | 16.43 | | 491.1075 | [M-H]- | | C26H20O10 | | -3.79 | | 91.21 | 491.10,311.05,293.04,197.04 | Salvianolic acid C or its isomer | Phenolics | 32 | 115841-09-3 |
| 459 | 16.44 | | 433.1208 | [M-H]- | | C_21_H_22_O_10_ | | 2.01 | | 84.20 | 433.12,271.06,151.00,119.05 | Naringenin-7-O-β-D-glucoside or its isomer | Flavonoids | 41 | 529-55-5 |
| 460 | 16.49 | | 178.0951 | [M-H]- | | C_10_H_13_NO_2_ | | 2.79 | | 80.89 | 178.08,150.06 | (-)-Salsolinol | Alkaloid | 65,81 | 27740-96-1 |
| 461 | 16.53 | | 331.1991 | [M-H]- | | C_20_H_28_O_4_ | | 1.12 | | 81.67 | 331.19,303.19 | Carnosic acid or its isomer | Terpenoids | 55,68 | 3650-09-7 |
| 462 | 16.62 | | 275.1044 | [M-H]- | | C_15_H_16_O_5_ | | -0.20 | | 87.75 | 275.09,257.08,217.05 | Hamaudol or its isomer | Flavonoids | 65,97 | 735-46-6 |
| 463 | 16.74 | | 121.0371 | [M-H]- | | C_7_H_6_O_2_ | | 2.62 | | 74.50 | 121.03,77.03 | Benzoic Acid or its isomer | Organic acid | 54 | 65-85-0 |
| 464 | 16.75 | | 285.0487 | [M-H]- | | C_15_H_10_O_6_ | | 3.42 | | 77.76 | 285.04,257.04,241.05 | 3-Hydroxymorindone or its isomer | Anthraquinone | 10 | 80368-74-7 |
| 465 | 16.86 | | 315.0596 | [M-H]- | | C_16_H_12_O_7_ | | 3.96 | | 85.65 | 315.05,149.00 | Isorhamnetin or its isomer | Flavonoids | 10 | 480-19-3 |
| 466 | 16.97 | | 445.0884 | [M-H]- | | C_21_H_18_O_11_ | | 2.10 | | 98.24 | 445.07,269.06,175.02,113.02.85.02,59.01 | Glychionide or its isomer | Flavonoids | 49 | 119152-50-0 |
| 467 | 17.03 | | 629.1908 | [M+HCOO]- | | C30H32O12 | | -2.44 | | 91.94 | 583.19,121.02,77.03 | Benzoylpaeoniflorin or its isomer | Terpenoids | 35 | 38642-49-8 |
| 468 | 17.04 | | 845.4915 | [M+HCOO]- | | C_42_H_72_O_14_ | | 2.06 | | 98.99 | 799.48,637.43 | Mogroside IIA2 | Terpenoids | 17,28,29,58,74,76 | 88901-45-5 |
| 469 | 17.04 | | 329.0733 | [M-H]- | | C_17_H_14_O_7_ | | -1.91 | | 91.06 | 329.07,313.04,299.01 | Jaceosidin or its isomer | Flavonoids | 10,11 | 18085-97-7 |
| 470 | 17.07 | | 845.4915 | [M+HCOO]- | | C_42_H_72_O_14_ | | -4.12 | | 79.99 | 799.49,637.43,475.37 | Ginsenoside Rf | Terpenoids | 25 | 52286-58-5 |
| 471 | 17.08 | | 329.0752 | [M-H]- | | C_17_H_14_O_7_ | | -3.92 | | 93.25 | 329.06,314.04,299.01 | Aurantio-obtusifolin | Anthraquinone | 52 | 67979-25-3 |
| 472 | 17.08 | | 799.5001 | [M+HCOO]- | | C_42_H_72_O_14_ | | 2.01 | | 91.39 | 799.50,653.42 | Pseudoginsenoside F11 | Terpenoids | 70 | 69884-00-0 |
| 473 | 17.10 | | 271.0698 | [M-H]- | | C_15_H_12_O_5_ | | 5.04 | | 72.74 | 271.06,151.00 | Naringenin chalcone | Chalcones | 77 | 73692-50-9 |
| 474 | 17.10 | | 491.1053 | [M-H]- | | C26H20O10 | | 0.68 | | 95.53 | 491.10,311.05,293.04,197.04 | Salvianolic acid C or its isomer | Phenolics | 25,32 | 115841-09-3 |
| 475 | 17.12 | | 949.4387 | [M-H]- | | C_44_H_70_O_22_ | | 1.01 | | 99.91 | 949.43,787.38 | Rebaudioside C | Terpenoids | 11,41,50 | 63550-99-2 |
| 476 | 17.16 | | 269.0899 | [M-H]- | | C_16_H_14_O_4_ | | 2.46 | | 91.55 | 269.08,253.04 | Echinatin or its isomer | Chalcones | 5,28,47,56,77 | 34221-41-5 |
| 477 | 17.17 | | 299.0628 | [M-H]- | | C_16_H_12_O_6_ | | -2.09 | | 92.17 | 299.06,283.03,255.03 | 3'-Methoxyapigenin or its isomer | Flavonoids | 1,3,4,9,46,47,50,53 | 491-71-4 |
| 478 | 17.18 | | 357.1444 | [M-H]- | | C_20_H_22_O_6_ | | 2.17 | | 96.04 | 357.13,313.14,191.10,167.03,123.04 | Matairesinol or its isomer | Lignans | 54 | 580-72-3 |
| 479 | 17.18 | | 514.2914 | [M-H]- | | C_26_H_45_NO_7_S | | 0.49 | | 96.75 | 514.28,392.98,124.00 | Taurocholic acid or its isomer | Steroids | 36,45,97 | 81-24-3 |
| 480 | 17.18 | | 299.0668 | [M-H]- | | C_16_H_12_O_6_ | | 4.10 | | 92.66 | 299.05,283.03 | Tectorigenin or its isomer | Isoflavone | 2,5,7,52 | 548-77-6 |
| 481 | 17.18 | | 269.0516 | [M-H]- | | C_15_H_10_O_5_ | | -4.49 | | 84.13 | 269.05,241.05,225.05,197.06,181.06 | Emodin or its isomer | Quinones | 11,54,57,61,64,68,91 | 518-82-1 |
| 482 | 17.19 | | 269.0535 | [M-H]- | | C_15_H_10_O_5_ | | -2.35 | | 95.71 | 269.04,225.05,201.05,181.06,151.00,117.03,83.01,65.00 | Apigenin | Flavonoids | 55,62 | 520-36-5 |
| 483 | 17.20 | | 275.1042 | [M-H]- | | C_15_H_16_O_5_ | | 3.10 | | 87.75 | 275.09,257.08,217.05 | Hamaudol or its isomer | Flavonoids | 65,86 | 735-46-6 |
| 484 | 17.20 | | 269.0518 | [M-H]- | | C_15_H_10_O_5_ | | -3.68 | | 86.96 | 269.05,241.04 | Aloeemodin or its isomer | Anthraquinone | 17,31,43 | 481-72-1 |
| 485 | 17.21 | | 299.0639 | [M-H]- | | C_16_H_12_O_6_ | | 1.67 | | 91.78 | 299.06,285.04 | Hispidulin or its isomer | Flavonoids | 16,33,46,49,51 | 1447-88-7 |
| 486 | 17.21 | | 253.0582 | [M-H]- | | C_15_H_10_O_4_ | | -1.11 | | 95.36 | 253.05,225.05,197.06 | Chrysophanol or its isomer | Anthraquinone | 93 | 481-74-3 |
| 487 | 17.21 | | 269.0544 | [M-H]- | | C_15_H_10_O_5_ | | -6.01 | | 89.64 | 269.04,240.04,225.05,197.06 | 4',6,7-Trihydroxyisoflavone | Isoflavone | 21 | 17817-31-1 |
| 488 | 17.25 | | 283.0690 | [M-H]- | | C_16_H_12_O_5_ | | -1.88 | | 99.07 | 283.07,267.04 | Genkwanin or its isomer | Flavonoids | 3,8,9,42,63,82,91,92,93 | 437-64-9 |
| 489 | 17.27 | | 301.0782 | [M-H]- | | C16H14O6 | | 2.65 | | 73.29 | 301.07,286.04,164.01 | Hesperetin | Flavonoids | 45,95 | 520-33-2 |
| 490 | 17.28 | | 445.1212 | [M-H]- | | C_22_H_22_O_10_ | | -0.26 | | 87.37 | 445.12,239.04 | Rheochrysin | Anthraquinone | 9 | 23451-01-6 |
| 491 | 17.29 | | 331.2003 | [M-H]- | | C_20_H_28_O_4_ | | -4.49 | | 86.62 | 331.19,303.19 | Carnosic acid or its isomer | Terpenoids | 55 | 3650-09-7 |
| 492 | 17.31 | | 815.4861 | [M+HCOO]- | | C_41_H_70_O_13_ | | 2.05 | | 97.10 | 769.48,637.43 | 20(S)-NotoginsenosideR2 | Terpenoids | 19,27,74,91 | 80418-25-3 |
| 493 | 17.31 | | 359.0813 | [M-H]- | | C_18_H_16_O_8_ | | 2.65 | | 80.60 | 359.07,345.05,330.03 | 5,7,3'-Trihydroxy-6,4',5'-trimethoxyflavone or its isomer | Flavonoids | 11 | 78417-26-2 |
| 494 | 17.36 | | 815.4803 | [M-H]- | | C_41_H_70_O_13_ | | 1.72 | | 93.28 | 769.47,637.42 | 20(R)-Notoginsenoside | Terpenoids | 18,26,27,29,32,58,75,81,84 | 948046-15-9 |
| 495 | 17.45 | | 285.0468 | [M-H]- | | C_15_H_10_O_6_ | | 3.20 | | 85.61 | 285.03,257.04,213.05 | Kaempferol or its isomer | Flavonoids | 2,42,82,93 | 520-18-3 |
| 496 | 17.50 | | 247.1408 | [M-H]- | | C_15_H_20_O_3_ | | 1.98 | | 88.77 | 247.13,203.14 | Atractylenolide III | Terpenoids | 52 | 73030-71-4 |
| 497 | 17.52 | | 299.0635 | [M-H]- | | C_16_H_12_O_6_ | | 2.82 | | 99.39 | 299.06,285.03 | Kaempferide or its isomer | Flavonoids | 41 | 491-54-3 |
| 498 | 17.54 | | 299.0621 | [M-H]- | | C_16_H_12_O_6_ | | -4.19 | | 94.50 | 299.06,283.03,255.03 | 3'-Methoxyapigenin or its isomer | Flavonoids | 1,4,5,10,11,47,49,51,61 | 491-71-4 |
| 499 | 17.55 | | 269.0540 | [M-H]- | | C_15_H_10_O_5_ | | -4.47 | | 91.82 | 269.05,241.05,225.05 | 6-Hydroxyrubiadin or its isomer | Anthraquinone | 81 | 87686-86-0 |
| 500 | 17.55 | | 299.0644 | [M-H]- | | C_16_H_12_O_6_ | | -3.22 | | 91.36 | 299.05,283.03 | Tectorigenin or its isomer | Isoflavone | 2,52,81,92 | 548-77-6 |
| 501 | 17.58 | | 815.4828 | [M+HCOO]- | | C_41_H_70_O_13_ | | 2.05 | | 91.20 | 769.47,637.43,149.04 | Ginsenoside F5 or its isomer | Terpenoids | 76 | 189513-26-6 |
| 502 | 17.59 | | 269.0533 | [M-H]- | | C_15_H_10_O_5_ | | -1.59 | | 93.43 | 269.04,241.03,223.04,197.06,169.06,135.98 | Baicalein or its isomer | Flavonoids | 2,4,5,33,49,51,52,81,92,98 | 491-67-8 |
| 503 | 17.61 | | 829.4988 | [M+HCOO]- | | C_42_H_72_O_13_ | | -0.28 | | 97.52 | 783.49,637.43,475.37 | 20(R)-Ginsenoside Rg2 or its isomer | Terpenoids | 18,27,29,70,75,84 | 80952-72-3 |
| 504 | 17.62 | | 315.0557 | [M-H]- | | C_16_H_12_O_7_ | | -8.17 | | 76.74 | 315.05,149.00 | Isorhamnetin or its isomer | Flavonoids | 40 | 480-19-3 |
| 505 | 17.63 | | 687.3282 | [M+HCOO]- | | C_32_H_50_O_13_ | | 3.01 | | 97.18 | 641.32,521.27,479.26 | Rubusoside | Terpenoids | 63 | 64849-39-4 |
| 506 | 17.64 | | 1092.5724 | [M+HCOO]- | | C_52_H_86_O_21_ | | -4.91 | | 79.24 | 1046.57,913.51,751.46 | Gypenoside XLIX | Saponin | 96 | 94987-08-3 |
| 507 | 17.65 | | 285.0484 | [M-H]- | | C_15_H_10_O_6_ | | -2.40 | | 92.75 | 285.04,257.04,241.05 | 3-Hydroxymorindone or its isomer | Anthraquinone | 81 | 80368-74-7 |
| 508 | 17.65 | | 829.4985 | [M+HCOO]- | | C_42_H_72_O_13_ | | 2.98 | | 94.20 | 783.49,637.43 | 20(S)-Ginsenoside Rg3 or its isomer | Terpenoids | 76 | 14197-60-5 |
| 509 | 17.68 | | 829.4959 | [M+HCOO]- | | C42H72O13 | | 1.78 | | 96.14 | 784.49,638.42 | 20(S)-Ginsenoside Rg2 | Terpenoids | 32,58 | 52286-74-5 |
| 510 | 17.68 | | 329.0745 | [M-H]- | | C_17_H_14_O_7_ | | 1.68 | | 98.34 | 329.07,313.04,299.01 | Jaceosidin or its isomer | Flavonoids | 5,10,49,51,53 | 18085-97-7 |
| 511 | 17.70 | | 448.3150 | [M-H]- | | C26H43NO5 | | -2.05 | | 96.2 | 448.31,74.02 | Glycoursodeoxycholic acid or its isomer | Steroids | 83 | 64480-66-6 |
| 512 | 17.71 | | 973.5089 | [M+HCOO]- | | C_47_H_76_O_18_ | | 2.38 | | 98.69 | 927.50,610.24 | Bacopaside II | Saponin | 96 | 382146-66-9 |
| 513 | 17.72 | | 269.0524 | [M-H]- | | C_15_H_10_O_5_ | | 1.44 | | 91.06 | 269.05,241.04 | Aloeemodin or its isomer | Anthraquinone | 2,94 | 481-72-1 |
| 514 | 17.73 | | 683.4394 | [M+HCOO]- | | C36H62O9 | | 0.01 | | 93.69 | 637.43,476.37 | 20(S)-Ginsenoside Rh1 | Terpenoids | 19,32 | 63223-86-9 |
| 515 | 17.77 | | 299.0679 | [M-H]- | | C_16_H_12_O_6_ | | 2.06 | | 96.85 | 299.06,283.03,255.03 | 3'-Methoxyapigenin or its isomer | Flavonoids | 5,7,48,51,81 | 491-71-4 |
| 516 | 17.82 | | 299.0619 | [M-H]- | | C_16_H_12_O_6_ | | -2.10 | | 93.93 | 299.06,285.04 | Hispidulin or its isomer | Flavonoids | 10,48,49 | 1447-88-7 |
| 517 | 17.88 | | 955.5021 | [M-H]- | | C_48_H_76_O_19_ | | 1.58 | | 90.44 | 955.49,793.44,731.44,523.37,455.35,157.01,89.02 | Ginsenoside Ro | Terpenoids | 76,93 | 34367-04-9 |
| 518 | 17.89 | | 629.1905 | [M+HCOO]- | | C30H32O12 | | -1.95 | | 87.56 | 583.19,121.02,77.03 | Benzoylpaeoniflorin or its isomer | Terpenoids | 35 | 38642-49-8 |
| 519 | 17.96 | | 1123.5963 | [M+HCOO]- | | C_53_H_90_O_22_ | | 1.68 | | 91.88 | 1077.58,945.54,915.52,783.48,293.08,149.04 | Ginsenoside Rb3 | Terpenoids | 18 | 68406-26-8 |
| 520 | 18.03 | | 269.0529 | [M-H]- | | C_15_H_10_O_5_ | | 0.46 | | 73.96 | 269.04,241.03,223.04,197.06,169.06,135.98 | Baicalein or its isomer | Flavonoids | 71 | 491-67-8 |
| 521 | 18.09 | | 665.4347 | [M+HCOO]- | | C_36_H_60_O_8_ | | 3.07 | | 86.67 | 619.43,512.45 | Ginsenoside Rk3 | Terpenoids | 74 | 364779-15-7 |
| 522 | 18.10 | | 283.0679 | [M-H]- | | C_16_H_12_O_5_ | | 2.00 | | 97.72 | 283.07,267.04 | Genkwanin or its isomer | Flavonoids | 93 | 437-64-9 |
| 523 | 18.19 | | 943.5370 | [M-H]- | | C_48_H_80_O_18_ | | 2.76 | | 88.52 | 943.52,781.47,619.42,161.04,89.02 | 5,6-Dehydrogensenoside Rd | Terpenoids | 18 | 1268459-68-2 |
| 524 | 18.23 | | 275.1051 | [M-H]- | | C_15_H_16_O_5_ | | 3.83 | | 87.75 | 275.09,257.08,217.05 | Hamaudol or its isomer | Flavonoids | 65 | 735-46-6 |
| 525 | 18.25 | | 253.0576 | [M-H]- | | C_15_H_10_O_4_ | | 1.18 | | 92.18 | 253.05,225.05,197.06 | Chrysophanol or its isomer | Anthraquinone | 81,92,93 | 481-74-3 |
| 526 | 18.26 | | 255.0742 | [M-H]- | | C_15_H_12_O_4_ | | 2.59 | | 96.35 | 255.06,213.05,135.00,119.05 | Isoliquiritigenin or its isomer | Chalcones | 1,5,8,9,16,28,44,47,56,69,72,73,77,86,88 | 961-29-5 |
| 527 | 18.28 | | 464.3083 | [M-H]- | | C_26_H_43_NO_6_ | | 1.49 | | 97.37 | 464.30,402.30 | Glycocholic acid | Terpenoids | 97 | 475-31-0 |
| 528 | 18.32 | | 331.1971 | [M-H]- | | C20H28O4 | | 4.93 | | 90.36 | 331.19,303.19 | Carnosic acid or its isomer | Terpenoids | 32 | 3650-09-7 |
| 529 | 18.34 | | 359.0842 | [M-H]- | | C_18_H_16_O_8_ | | -0.82 | | 95.00 | 359.07,345.05,330.03 | 5,7,3'-Trihydroxy-6,4',5'-trimethoxyflavone or its isomer | Flavonoids | 11 | 78417-26-2 |
| 530 | 18.37 | | 448.3146 | [M-H]- | | C_26_H_43_NO_5_ | | -0.96 | | 96.77 | 448.31,74.02 | Glycoursodeoxycholic acid or its isomer | Steroids | 37 | 64480-66-6 |
| 531 | 18.38 | | 637.4437 | [M-H]- | | C_36_H_62_O_9_ | | -4.19 | | 88.40 | 637.44,475.37 | 20(S)-Ginsenoside F1 | Terpenoids | 19 | 53963-43-2 |
| 532 | 18.38 | | 259.1103 | [M-H]- | | C_15_H_16_O_4_ | | 3.86 | | 87.76 | 259.09,187.01 | Linderane or its isomer | Terpenoids | 65 | 13476-25-0 |
| 533 | 18.39 | | 267.0743 | [M-H]- | | C_16_H_12_O_4_ | | 2.73 | | 93.05 | 267.06,251.04,223.03,195.04,131.02 | Formononetin | Flavonoids | 4,5,6,8,9,11,19,23,24,28,29,41,47,55,56,68,73,76,77,79,86,92 | 485-72-3 |
| 534 | 18.42 | | 992.5555 | [M+HCOO]- | | C_48_H_82_O_18_ | | -2.97 | | 96.85 | 946.55,842.24 | Ginsenoside Re or its isomer | Terpenoids | 20,77,93 | 52286-59-6 |
| 535 | 18.43 | | 498.2974 | [M-H]- | | C26H45NO6S | | -1.32 | | 94.73 | 498.29,124.00 | Tauroursodeoxycholic acid or its isomer | Steroids | 36,45,83,97 | 14605-22-2 |
| 536 | 18.46 | | 371.1598 | [M-H]- | | C_21_H_24_O_6_ | | 2.07 | | 83.34 | 371.15,259.10,83.01 | Arctigenin | Flavonoids | 54 | 7770-78-7 |
| 537 | 18.49 | | 991.5508 | [M+HCOO]- | | C48H82O18 | | -0.69 | | 99.24 | 945.56,799.47,469.10 | Ginsenoside Rd or its isomer | Terpenoids | 18,27,32,58,75,76,81,84 | 52705-93-8 |
| 538 | 18.57 | | 793.4464 | [M-H]- | | C42H66O14 | | -1.48 | | 95.79 | 793.43,631.37 | Chikusetsusaponin IVa | Terpenoids | 95 | 51415-02-2 |
| 539 | 18.66 | | 821.4045 | [M-H]- | | C42H62O16 | | -0.84 | | 97.65 | 821.39,351.05,193.03 | Glycyrrhizic acid or its isomer | Terpenoids | 6,12,24,36,69,73,88,90,95 | 1405-86-3 |
| 540 | 18.70 | | 349.2091 | [M-H]- | | C_20_H_30_O_5_ | | 2.76 | | 87.33 | 349.20,69.03 | Andropanolide | Terpenoids | 55 | 869807-57-8 |
| 541 | 18.72 | | 331.1990 | [M-H]- | | C_20_H_28_O_4_ | | 4.01 | | 82.77 | 331.19,303.19 | Carnosic acid or its isomer | Terpenoids | 55,68 | 3650-09-7 |
| 542 | 18.74 | | 357.1459 | [M-H]- | | C_20_H_22_O_6_ | | -3.26 | | 85.67 | 357.14,237.08,145.01 | Triptonide | Terpenoids | 77 | 38647-11-9 |
| 543 | 18.80 | | 329.0739 | [M-H]- | | C_17_H_14_O_7_ | | -0.01 | | 89.26 | 329.07,313.04,299.01 | Jaceosidin or its isomer | Flavonoids | 48,49 | 18085-97-7 |
| 544 | 18.88 | | 269.0543 | [M-H]- | | C_15_H_10_O_5_ | | -5.56 | | 88.38 | 269.05,241.04 | Aloeemodin or its isomer | Anthraquinone | 82 | 481-72-1 |
| 545 | 18.90 | | 229.0944 | [M-H]- | | C_14_H_14_O_3_ | | -0.44 | | 93.14 | 229.08,173.03 | 7-Demethylsuberosin or its isomer | Coumarins | 29,44,45,52,95 | 21422-04-8 |
| 546 | 18.91 | | 283.0695 | [M-H]- | | C_16_H_12_O_5_ | | 3.65 | | 99.13 | 283.07,267.04 | Genkwanin or its isomer | Flavonoids | 7 | 437-64-9 |
| 547 | 18.93 | | 675.2403 | [M-H]- | | C_33_H_40_O_15_ | | -5.34 | | 89.03 | 675.22,367.12 | Baohuoside VII or its isomer | Flavonoids | 29 | 119730-89-1 |
| 548 | 18.94 | | 269.0541 | [M-H]- | | C_15_H_10_O_5_ | | -4.91 | | 90.65 | 269.05,241.05,225.05 | 6-Hydroxyrubiadin or its isomer | Anthraquinone | 93 | 87686-86-0 |
| 549 | 18.95 | | 991.5525 | [M+HCOO]- | | C_48_H_82_O_18_ | | 2.97 | | 90.50 | 946.55,842.24 | Ginsenoside Re or its isomer | Terpenoids | 18,58,81 | 52286-59-6 |
| 550 | 18.98 | | 357.1436 | [M-H]- | | C_20_H_22_O_6_ | | -5.43 | | 85.63 | 357.13,313.14,191.10,167.03,123.04 | Matairesinol or its isomer | Lignans | 84 | 580-72-3 |
| 551 | 19.06 | | 259.1094 | [M-H]- | | C_15_H_16_O_4_ | | -3.86 | | 87.76 | 259.09,187.01 | Linderane or its isomer | Terpenoids | 65,70 | 13476-25-0 |
| 552 | 19.08 | | 367.1261 | [M-H]- | | C_21_H_20_O_6_ | | 1.64 | | 92.33 | 367.12,309.04,203.07 | Glycycoumarin or its isomer | Coumarins | 5,17,44,47,77 | 94805-82-0 |
| 553 | 19.09 | | 941.5219 | [M-H]- | | C_48_H_78_O_18_ | | 3.51 | | 90.35 | 941.51,879.50,615,38,85.02 | Soyasaponin Bb | Terpenoids | 21,57,64 | 51330-27-9 |
| 554 | 19.11 | | 961.5418 | [M+HCOO]- | | C_47_H_80_O_17_ | | -5.62 | | 84.78 | 915.53,783.48,621.42 | Notoginsenoside Ft1 | Terpenoids | 18,27,76 | 155683-00-4 |
| 555 | 19.12 | | 331.1987 | [M-H]- | | C20H28O4 | | 0.05 | | 87.60 | 331.19,303.19 | Carnosic acid or its isomer | Terpenoids | 32,99 | 3650-09-7 |
| 556 | 19.12 | | 359.0863 | [M-H]- | | C_18_H_16_O_8_ | | 1.65 | | 90.96 | 359.07,345.05,330.03 | 5,7,3'-Trihydroxy-6,4',5'-trimethoxyflavone or its isomer | Flavonoids | 5,10,81 | 78417-26-2 |
| 557 | 19.13 | | 825.4663 | [M+HCOO]- | | C_42_H_68_O_13_ | | 0.44 | | 98.18 | 779.46,617.41 | Saikosaponin D or its isomer | Steroids | 40 | 20874-52-6 |
| 558 | 19.18 | | 313.0797 | [M-H]- | | C_17_H_14_O_6_ | | -2.09 | | 87.28 | 313.07,297.04.283.02 | Kumatakenin or its isomer | Flavonoids | 45 | 3301-49-3 |
| 559 | 19.24 | | 821.4062 | [M-H]- | | C42H62O16 | | -2.94 | | 90.13 | 821.39,351.05,193.03 | Glycyrrhizic acid or its isomer | Terpenoids | 28,95 | 1405-86-3 |
| 560 | 19.25 | | 825.4767 | [M+HCOO]- | | C_42_H_68_O_13_ | | -3.19 | | 92.29 | 779.47,618.40 | Saikosaponin A | Terpenoids | 16 | 20736-09-8 |
| 561 | 19.26 | | 529.1809 | [M-H]- | | C_27_H_30_O_11_ | | -4.00 | | 83.08 | 529.17,409.13 | Anhydroicaritin-7-O-glucoside | Flavonoids | 29 | 56725-99-6 |
| 562 | 19.27 | | 283.0320 | [M-H]- | | C_15_H_8_O_6_ | | 0.21 | | 97.02 | 283.03,239.03,211.04,183.04,167.05,155.05,139.05,127.05 | Rhein or its isomer | Anthraquinone | 2,8,81,82,93 | 478-43-3 |
| 563 | 19.33 | | 659.2446 | [M-H]- | | C_33_H_40_O_14_ | | -4.22 | | 93.48 | 659.23,365.11 | 2''-O-Rhamnosylicariside II or its isomer | Flavonoids | 29 | 135293-13-9 |
| 564 | 19.43 | | 353.1462 | [M-H]- | | C_21_H_22_O_5_ | | 1.58 | | 81.01 | 353.14,254.05 | Xanthohumol or its isomer | Flavonoids | 42 | 56754-58-1 |
| 565 | 19.44 | | 537.0938 | [M-H]- | | C_30_H_18_O_10_ | | -7.07 | | 80.40 | 537.08,375.05,234.02 | Amentoflavone | Flavonoids | 81 | 1617-53-4 |
| 566 | 19.48 | | 283.0680 | [M-H]- | | C_16_H_12_O_5_ | | -1.58 | | 98.64 | 283.07,267.04 | Genkwanin or its isomer | Flavonoids | 1,2,3,4,5,7,9,10,11,33,41,42,46,47,48,49,50,51,52,53,81,92,94,100 | 437-64-9 |
| 567 | 19.50 | | 437.2922 | [M+HCOO]- | | C24H40O4 | | 1.24 | | 94.06 | 391.29,323.29 | Ursodeoxycholic Acid or its isomer | Steroids | 83 | 128-13-2 |
| 568 | 19.61 | | 253.0583 | [M-H]- | | C_15_H_10_O_4_ | | -1.49 | | 92.69 | 253.05,225.04,209.06,197.06,135.00 | Daidzein or its isomer | Isoflavone | 2,33 | 486-66-8 |
| 569 | 19.62 | | 253.0579 | [M-H]- | | C_15_H_10_O_4_ | | 0.12 | | 95.44 | 253.05,225.05,209.06,185.06,165.07,143.05,107.01,89.00,63.02 | Chrysin | Flavonoids | 4,5,49,51,52,53,81,92,100 | 480-40-0 |
| 570 | 19.70 | | 313.0791 | [M-H]- | | C_17_H_14_O_6_ | | 0.10 | | 98.27 | 313.07,297.04.283.02 | Kumatakenin or its isomer | Flavonoids | 2,3,4,5,7,10,46,47,48,49,50,51,53,55,81,100 | 3301-49-3 |
| 571 | 19.71 | | 407.2876 | [M-H]- | | C_24_H_40_O_5_ | | -0.06 | | 93.91 | 407.28,389.26,371.26,343,26,289.21,251.20,205.15,123.08,95.05,69.03 | Cholic acid | Steroids | 36,45,97 | 81-25-4 |
| 572 | 19.73 | | 285.0869 | [M-H]- | | C_16_H_14_O_5_ | | -4.95 | | 80.56 | 285.08,165,01,93.03 | 5,7,4’-Trihydroxy-8-methylflavanone | Flavonoids | 62 | 916917-28-7 |
| 573 | 19.75 | | 373.0995 | [M-H]- | | C_19_H_18_O_8_ | | 1.81 | | 88.35 | 373.09,358.06,343.04.328.02,300.02 | Chrysosplenetin B | Flavonoids | 2,33,52,94 | 603-56-5 |
| 574 | 19.75 | | 915.4631 | [M+HCOO]- | | C_44_H_70_O_17_ | | 0.74 | | 96.13 | 869.45,737.41 | Polyphyllin II | Terpenoids | 75 | 76296-72-5 |
| 575 | 19.77 | | 283.0705 | [M-H]- | | C_16_H_12_O_5_ | | 7.05 | | 90.82 | 283.06,119.05 | Biochanin A or its isomer | Flavonoids | 25,61 | 491-80-5 |
| 576 | 19.77 | | 825.4683 | [M+HCOO]- | | C_42_H_68_O_13_ | | 4.72 | | 88.30 | 779.46,618.41 | Saikosaponin B2 | Terpenoids | 16 | 58316-41-9 |
| 577 | 19.79 | | 373.1013 | [M-H]- | | C_19_H_18_O_8_ | | -2.92 | | 92.70 | 373.09,358.06,343.04 | Vitexicarpin | Flavonoids | 32,81 | 479-91-4 |
| 578 | 19.81 | | 811.4889 | [M+HCOO]- | | C_42_H_70_O_12_ | | -1.75 | | 96.41 | 765.48,619.42 | Ginsenoside Rg4 or its isomer | Terpenoids | 27,29,74,76 | 126223-28-7 |
| 579 | 19.84 | | 331.1985 | [M-H]- | | C_20_H_28_O_4_ | | 0.78 | | 97.21 | 331.19,303.19 | Carnosic acid or its isomer | Terpenoids | 23,84,97 | 3650-09-7 |
| 580 | 19.85 | | 283.0691 | [M-H]- | | C_16_H_12_O_5_ | | -2.13 | | 91.71 | 283.07,267.04 | Genkwanin or its isomer | Flavonoids | 3,4,5,7,10,11,46,47,48,50,51,52,53,62,81,92,100 | 437-64-9 |
| 581 | 19.87 | | 283.0668 | [M-H]- | | C_16_H_12_O_5_ | | -5.99 | | 95.69 | 283.07,267.03 | Wogonin | Flavonoids | 1,2,4,49 | 632-85-9 |
| 582 | 19.90 | | 283.0678 | [M-H]- | | C_16_H_12_O_5_ | | 2.32 | | 94.50 | 283.06,267.05 | 7-O-Methylbaicalein or its isomer | Flavonoids | 33 | 29550-13-8 |
| 583 | 19.90 | | 448.3128 | [M-H]- | | C_26_H_43_NO_5_ | | 2.87 | | 90.52 | 448.31,74.02 | Glycoursodeoxycholic acid or its isomer | Steroids | 37,83,97 | 64480-66-6 |
| 584 | 19.97 | | 513.1842 | [M-H]- | | C_27_H_30_O_10_ | | -0.54 | | 93.23 | 513.17,365.11,351.08,323.09 | Baohuoside I or its isomer | Flavonoids | 12,29,94 | 113558-15-9 |
| 585 | 19.98 | | 313.0781 | [M-H]- | | C_17_H_14_O_6_ | | -0.56 | | 91.06 | 313.07,163.00 | Pectolinarigenin | Flavonoids | 92 | 520-12-7 |
| 586 | 20.01 | | 329.0764 | [M-H]- | | C_17_H_14_O_7_ | | 7.54 | | 96.20 | 329.07,313.04,299.01 | Jaceosidin or its isomer | Flavonoids | 60 | 18085-97-7 |
| 587 | 20.04 | | 437.2930 | [M+HCOO]- | | C24H40O4 | | -0.97 | | 98.96 | 391.29,337.00 | Chenodeoxycholic acid or its isomer | Steroids | 83 | 474-25-9 |
| 588 | 20.11 | | 229.0944 | [M-H]- | | C_14_H_14_O_3_ | | -0.66 | | 94.75 | 229.08,173.03 | 7-Demethylsuberosin or its isomer | Coumarins | 42,52,86,86,95 | 21422-04-8 |
| 589 | 20.11 | | 391.2928 | [M-H]- | | C24H40O4 | | -0.45 | | 89.44 | 391.29,323.29 | Ursodeoxycholic Acid or its isomer | Steroids | 63,83 | 128-13-2 |
| 590 | 20.13 | | 353.1143 | [M-H]- | | C_20_H_18_O_6_ | | -3.63 | | 85.44 | 353.11,297.04,175.07 | Licoflavonol or its isomer | Flavonoids | 47,77 | 60197-60-6 |
| 591 | 20.13 | | 453.2013 | [M-H]- | | C_26_H_30_O_7_ | | -4.64 | | 83.73 | 453.19,409.20,391.19 | Obacunone | Terpenoids | 64 | 751-03-1 |
| 592 | 20.17 | | 321.1220 | [M-H]- | | C_20_H_18_O_4_ | | 4.59 | | 88.83 | 321.12,279.99 | Glabrene | Phenolics | 68,71 | 60008-03-9 |
| 593 | 20.19 | | 367.1270 | [M-H]- | | C_21_H_20_O_6_ | | 2.76 | | 98.08 | 367.12,309.04,203.07 | Glycycoumarin or its isomer | Coumarins | 1,5,17,28,40,47,56,95 | 94805-82-0 |
| 594 | 20.26 | | 331.1978 | [M-H]- | | C_20_H_28_O_4_ | | -2.85 | | 94.22 | 331.19,303.19 | Carnosic acid or its isomer | Terpenoids | 8,9,32 | 3650-09-7 |
| 595 | 20.31 | | 302.1356 | [M-H]- | | C_19_H_17_N_3_O | | -5.17 | | 95.61 | 302.13,170.07 | Evodiamine | Alkaloid | 72 | 518-17-2 |
| 596 | 20.31 | | 343.0926 | [M-H]- | | C_18_H_16_O_7_ | | 2.54 | | 84.21 | 343.09,259.06 | Usnic acid | Phenolics | 7,46,53 | 7562-61-0 |
| 597 | 20.35 | | 343.0909 | [M-H]- | | C_18_H_16_O_7_ | | 3.75 | | 90.89 | 343.09,241.05 | Lysionotin | Flavonoids | 4,11 | 152743-19-6 |
| 598 | 20.41 | | 315.2046 | [M-H]- | | C_20_H_28_O_3_ | | -2.25 | | 83.61 | 315.20,287.20 | 15-Hydroxydehydroabietic acid or its isomer | Terpenoids | 99 | 54113-95-0 |
| 599 | 20.42 | | 269.0885 | [M-H]- | | C16H14O4 | | 2.57 | | 92.22 | 269.08,254.05,214.02,201.01 | Imperatorin or its isomer | Coumarins | 6,42,52,73,87,95 | 482-44-0 |
| 600 | 20.47 | | 307.1061 | [M-H]- | | C_19_H_16_O_4_ | | -3.96 | | 90.38 | 307.09,187.04,143.05,119.05 | Bisdemethoxycurcumin | Phenolics | 81 | 33171-05-0 |
| 601 | 20.49 | | 313.0793 | [M-H]- | | C_17_H_14_O_6_ | | 0.68 | | 84.38 | 313.07,297.04.283.02 | Kumatakenin or its isomer | Flavonoids | 56,77 | 3301-49-3 |
| 602 | 20.64 | | 353.1130 | [M-H]- | | C_20_H_18_O_6_ | | -7.23 | | 81.99 | 353.11,297.04,175.07 | Licoflavonol or its isomer | Flavonoids | 5,77 | 60197-60-6 |
| 603 | 20.77 | | 269.0896 | [M-H]- | | C_16_H_14_O_4_ | | -1.42 | | 90.09 | 269.08,253.04 | Echinatin or its isomer | Chalcones | 42,47,95 | 34221-41-5 |
| 604 | 20.77 | | 283.0703 | [M-H]- | | C_16_H_12_O_5_ | | 6.46 | | 97.78 | 283.07,267.04 | Genkwanin or its isomer | Flavonoids | 41,60 | 437-64-9 |
| 605 | 20.78 | | 825.4668 | [M+HCOO]- | | C_42_H_68_O_13_ | | 1.07 | | 96.34 | 779.46,617.41 | Saikosaponin D or its isomer | Steroids | 40 | 20874-52-6 |
| 606 | 20.86 | | 829.4985 | [M+HCOO]- | | C_42_H_72_O_13_ | | -3.02 | | 97.18 | 783.49,637.43 | 20(S)-Ginsenoside Rg3 or its isomer | Terpenoids | 18,29,32,75,76,81 | 14197-60-5 |
| 607 | 20.92 | | 829.4985 | [M+HCOO]- | | C_42_H_72_O_13_ | | 1.50 | | 94.75 | 783.49,637.43,475.37 | 20(R)-Ginsenoside Rg2 or its isomer | Terpenoids | 18,19,27,29,58 | 80952-72-3 |
| 608 | 20.94 | | 811.4887 | [M+HCOO]- | | C_42_H_70_O_12_ | | -2.65 | | 96.77 | 765.48,619.42 | Ginsenoside Rg4 or its isomer | Terpenoids | 18 | 126223-28-7 |
| 609 | 20.98 | | 645.3726 | [M-H]- | | C36H54O10 | | -1.35 | | 94.14 | 645.37,569.34,523.34,469.33,157.01 | Glycyrrhetic acid 3-O-mono-b-D-glucuronide | Terpenoids | 47,73 | 34096-83-8 |
| 610 | 21.01 | | 269.0889 | [M-H]- | | C16H14O4 | | 1.00 | | 90.26 | 269.08,254.05,214.02,201.01 | Imperatorin or its isomer | Coumarins | 42,95 | 482-44-0 |
| 611 | 21.04 | | 691.2283 | [M-H]- | | C_40_H_36_O_11_ | | -3.64 | | 93.28 | 691.21,581.18 | Kuwanon G | Flavonoids | 23 | 75629-19-5 |
| 612 | 21.16 | | 339.1335 | [M-H]- | | C_20_H_20_O_5_ | | 4.51 | | 87.48 | 339.13,219.06 | 8-Prenylnaringenin | Flavonoids | 5 | 53846-50-7 |
| 613 | 21.31 | | 269.0890 | [M-H]- | | C16H14O4 | | 0.95 | | 92.08 | 269.08,253.04 | Echinatin or its isomer | Chalcones | 42,52,87,95 | 34221-41-5 |
| 614 | 21.36 | | 337.1158 | [M-H]- | | C_20_H_18_O_5_ | | 3.25 | | 94.87 | 337.11,283.05,134.02 | Wighteone | Isoflavone | 17,47,63,77 | 51225-30-0 |
| 615 | 21.38 | | 337.1185 | [M-H]- | | C_20_H_18_O_5_ | | -1.63 | | 90.44 | 337.11,281.04,253.08 | Licoflavone C | Flavonoids | 5 | 72357-31-4 |
| 616 | 21.46 | | 515.3112 | [M-H]- | | C_30_H_44_O_7_ | | 1.02 | | 100.00 | 515.30,453.28 | Ganoderic acid A | Terpenoids | 97 | 81907-62-2 |
| 617 | 21.49 | | 269.0521 | [M-H]- | | C_15_H_10_O_5_ | | 2.52 | | 95.40 | 269.05,241.05,225.05,197.06,181.06 | Emodin or its isomer | Quinones | 2,3,8,9,12,41,42,63,76,81,82,91,93,99 | 518-82-1 |
| 618 | 21.49 | | 353.1466 | [M-H]- | | C_21_H_22_O_5_ | | 0.29 | | 87.87 | 353.14,254.05 | Xanthohumol or its isomer | Flavonoids | 42 | 56754-58-1 |
| 619 | 21.50 | | 269.0509 | [M-H]- | | C_15_H_10_O_5_ | | -7.09 | | 88.27 | 269.05,241.04 | Aloeemodin or its isomer | Anthraquinone | 16 | 481-72-1 |
| 620 | 21.55 | | 297.1232 | [M-H]- | | C_18_H_18_O_4_ | | 9.08 | | 91.32 | 297.11,281.08,191.05,133.02 | Phenylethyl-3-methylcaffeate | Phenylpropanoids | 5,42 | 71835-85-3 |
| 621 | 21.55 | | 269.0531 | [M-H]- | | C_15_H_10_O_5_ | | 1.08 | | 97.80 | 270.04,241.03,223.04,197.06,169.06,136.98 | Baicalein | Flavonoids | 79 | 491-67-8 |
| 622 | 21.55 | | 501.3291 | [M-H]- | | C_30_H_46_O_6_ | | -0.75 | | 97.82 | 501.32,315.33 | Medicagenic acid | Terpenoids | 71 | 599-07-5 |
| 623 | 21.56 | | 319.2375 | [M-H]- | | C_20_H_32_O_3_ | | -7.42 | | 87.09 | 319.22,275.24 | Ginkgoneolic acid or its isomer | Phenolics | 99 | 20261-38-5 |
| 624 | 21.60 | | 389.2069 | [M-H]- | | C_22_H_30_O_6_ | | -1.43 | | 87.70 | 390.19,374.17 | Pregomisin | Lignans | 54,81 | 66280-26-0 |
| 625 | 21.61 | | 913.4841 | [M+HCOO]- | | C_45_H_72_O_16_ | | 2.81 | | 85.65 | 867.47,721.41 | Dioscin | Terpenoids | 75 | 19057-60-4 |
| 626 | 21.63 | | 337.1165 | [M-H]- | | C_20_H_18_O_5_ | | 3.14 | | 86.36 | 337.11,321.08,281.05 | Corylifol C | Flavonoids | 77 | 775351-91-2 |
| 627 | 21.68 | | 929.4789 | [M+HCOO]- | | C_45_H_72_O_17_ | | -2.64 | | 87.75 | 883.47,763.42,721.41,509.32,247.08,161.04,89.02 | Gracillin | Terpenoids | 75 | 19083-00-2 |
| 628 | 21.72 | | 335.0977 | [M-H]- | | C_20_H_16_O_5_ | | 1.93 | | 80.50 | 335.10,319.06,291.10 | Atalantoflavone | Flavonoids | 28,56,63,71,77,92 | 119309-02-3 |
| 629 | 21.76 | | 323.1359 | [M-H]- | | C20H20O4 | | -0.73 | | 81.19 | 323.13,279.13,201.09,135.04 | Glabridin | Isoflavane | 56,86 | 59870-68-7 |
| 630 | 21.81 | | 899.4686 | [M+HCOO]- | | C_44_H_70_O_16_ | | 3.62 | | 93.77 | 853.49,721.41 | Polyphyllin I | Terpenoids | 75 | 50773-41-6 |
| 631 | 21.82 | | 437.2934 | [M+HCOO]- | | C24H40O4 | | -1.85 | | 98.59 | 391.29,337.00 | Chenodeoxycholic acid or its isomer | Steroids | 83 | 474-25-9 |
| 632 | 21.84 | | 391.2934 | [M-H]- | | C24H40O4 | | -1.94 | | 88.44 | 391.29,237.00,113.00 | Hyodeoxycholic acid | Steroids | 83 | 83-49-8 |
| 633 | 21.84 | | 421.1720 | [M-H]- | | C_25_H_26_O_6_ | | 2.15 | | 90.32 | 421.16,299.12,193.08 | Kuwanon C or its isomer | Flavonoids | 23 | 62949-79-5 |
| 634 | 21.98 | | 357.1083 | [M-H]- | | C_19_H_18_O_7_ | | -1.85 | | 95.00 | 358.10,342.07,327.05 | Chrysoobtusin or its isomer | Anthraquinone | 68 | 70588-06-6 |
| 635 | 22.09 | | 391.2933 | [M-H]- | | C_24_H_40_O_4_ | | 1.57 | | 88.27 | 391.29,323.29 | Ursodeoxycholic Acid or its isomer | Steroids | 63,97 | 128-13-2 |
| 636 | 22.13 | | 471.3571 | [M-H]- | | C_30_H_48_O_4_ | | 3.97 | | 78.41 | 471.34,407.33 | Echinocystic acid or its isomer | Terpenoids | 17 | 510-30-5 |
| 637 | 22.20 | | 767.4254 | [M+HCOO]- | | C_39_H_62_O_12_ | | 1.05 | | 93.76 | 721.41,575.35,247.08,163.06,89.02 | Paris saponin V | Terpenoids | 75 | 19057-67-1 |
| 638 | 22.33 | | 201.0264 | [M-H]- | | C11H6O4 | | 1.03 | | 86.83 | 201.02,173.02,145.02,117.03 | Bergaptol or its isomer | Coumarins | 5,42,95 | 486-60-2 |
| 639 | 22.34 | | 269.0894 | [M-H]- | | C_16_H_14_O_4_ | | -0.64 | | 88.99 | 269.08,254.05,214.02,201.01 | Imperatorin or its isomer | Coumarins | 42,95 | 482-44-0 |
| 640 | 22.35 | | 269.0887 | [M-H]- | | C_16_H_14_O_4_ | | 2.02 | | 86.72 | 269.08,253.04 | Echinatin or its isomer | Chalcones | 5,52,86 | 34221-41-5 |
| 641 | 22.41 | | 343.1622 | [M-H]- | | C_20_H_24_O_5_ | | -0.50 | | 83.11 | 343.16,281.00 | Eriocalyxin B | Terpenoids | 49 | 84745-95-9 |
| 642 | 22.44 | | 285.0484 | [M-H]- | | C_15_H_10_O_6_ | | -2.14 | | 92.59 | 285.04,257.04,241.05 | 3-Hydroxymorindone or its isomer | Anthraquinone | 3,9,42,92 | 80368-74-7 |
| 643 | 22.67 | | 811.4887 | [M+HCOO]- | | C_42_H_70_O_12_ | | -0.72 | | 96.71 | 765.48,619.42 | Ginsenoside Rg4 or its isomer | Terpenoids | 18,27,29,81 | 126223-28-7 |
| 644 | 22.71 | | 265.1286 | [M-H]- | | C_18_H_18_O_2_ | | -2.94 | | 89.32 | 265.12,247.11 | Magnolol | Lignans | 11 | 528-43-8 |
| 645 | 22.80 | | 391.1989 | [M-H]- | | C_25_H_28_O_4_ | | 0.27 | | 92.09 | 391.19,203.07,131.05 | Glabrol | Phenolics | 56,92 | 59870-65-4 |
| 646 | 22.82 | | 315.2041 | [M-H]- | | C20H28O3 | | -0.95 | | 84.03 | 315.20,287.20 | 15-Hydroxydehydroabietic acid or its isomer | Terpenoids | 32,62 | 54113-95-0 |
| 647 | 23.02 | | 265.1313 | [M-H]- | | C18H18O2 | | -2.32 | | 92.11 | 266.13,251.13 | Honokiol | Phenolics | 73 | 564-73-8 |
| 648 | 23.10 | | 471.3558 | [M-H]- | | C30H48O4 | | -1.07 | | 80.53 | 471.35,407.33 | Hederagenin or its isomer | Terpenoids | 35,52 | 465-99-6 |
| 649 | 23.11 | | 315.2047 | [M-H]- | | C20H28O3 | | -2.77 | | 95.33 | 316.20,300.16,286.15,258.11,245.11,230.08,216.07 | 11-hydroxy-sugiol | Terpenoids | 32 | 88664-08-8 |
| 650 | 23.15 | | 311.1763 | [M-H]- | | C_20_H_24_O_3_ | | 3.05 | | 81.84 | 311.17,295.13,251.14 | Triptophenolide | Terpenoids | 1,22,31,32,40,43,50,51,52,56,62,65,80,93,98 | 74285-86-2 |
| 651 | 23.19 | | 331.2002 | [M-H]- | | C20H28O4 | | -4.22 | | 84.25 | 331.19,303.19 | Carnosic acid or its isomer | Terpenoids | 32,62 | 3650-09-7 |
| 652 | 23.23 | | 471.3565 | [M-H]- | | C_30_H_48_O_4_ | | 2.53 | | 79.51 | 471.34,407.33 | Echinocystic acid or its isomer | Terpenoids | 8,28,71 | 510-30-5 |
| 653 | 23.25 | | 253.0573 | [M-H]- | | C_15_H_10_O_4_ | | -2.40 | | 71.38 | 253.05,225.04,209.06,197.06,135.00 | Daidzein or its isomer | Isoflavone | 3 | 486-66-8 |
| 654 | 23.27 | | 253.0587 | [M-H]- | | C_15_H_10_O_4_ | | 2.97 | | 87.23 | 253.05,225.05,197.06 | Chrysophanol or its isomer | Anthraquinone | 92 | 481-74-3 |
| 655 | 23.37 | | 319.2382 | [M-H]- | | C_20_H_32_O_3_ | | -9.48 | | 86.38 | 319.22,275.24 | Ginkgoneolic acid or its isomer | Phenolics | 68,84 | 20261-38-5 |
| 656 | 23.62 | | 367.1263 | [M-H]- | | C_21_H_20_O_6_ | | -0.97 | | 92.38 | 367.11,352.09,309.03,281.04,265.05,175.00 | Icaritin | Flavonoids | 29 | 5240-95-9 |
| 657 | 23.65 | | 483.3476 | [M-H]- | | C_31_H_48_O_4_ | | 2.11 | | 76.07 | 483.34,421.30,337.25 | Dehydrotumulosic acid | Terpenoids | 24,35 | 6754-16-1 |
| 658 | 23.78 | | 471.3557 | [M-H]- | | C_30_H_48_O_4_ | | -0.83 | | 92.38 | 471.34,393.31 | Maslinic acid or its isomer | Terpenoids | 25,29,30,32,52,60,62,85,90 | 4373-41-5 |
| 659 | 23.81 | | 421.1740 | [M-H]- | | C_25_H_26_O_6_ | | 2.47 | | 84.19 | 421.16,299.12,193.08 | Kuwanon C or its isomer | Flavonoids | 5,47,86,92 | 62949-79-5 |
| 660 | 23.93 | | 497.3365 | [M-H]- | | C_31_H_46_O_5_ | | -1.07 | | 95.00 | 497.33,423.28,197.80 | Poricoic acid A | Terpenoids | 86 | 137551-38-3 |
| 661 | 24.05 | | 297.1568 | [M-H]- | | C_19_H_22_O_3_ | | 0.16 | | 95.19 | 297.15,227.07,213.06,173.03 | Auraptene | Coumarins | 5,42 | 495-02-3 |
| 662 | 24.06 | | 319.2339 | [M-H]- | | C_20_H_32_O_3_ | | 3.97 | | 91.31 | 319.22,275.24 | Ginkgoneolic acid or its isomer | Phenolics | 99 | 20261-38-5 |
| 663 | 24.22 | | 467.3223 | [M-H]- | | C_30_H_44_O_4_ | | 3.65 | | 81.89 | 467.31,451.25,423.32,385.23,325.25,285.22,97.06 | Ganoderic acid DM | Terpenoids | 23,84,97 | 173075-45-1 |
| 664 | 24.28 | | 283.0678 | [M-H]- | | C_16_H_12_O_5_ | | -2.35 | | 98.70 | 283.07,267.04 | Genkwanin or its isomer | Flavonoids | 3,9 | 437-64-9 |
| 665 | 24.37 | | 471.3565 | [M-H]- | | C_30_H_48_O_4_ | | -2.62 | | 90.69 | 471.34,393.31 | Maslinic acid or its isomer | Terpenoids | 23,97 | 4373-41-5 |
| 666 | 24.98 | | 471.3552 | [M-H]- | | C_30_H_48_O_4_ | | 0.19 | | 80.88 | 471.35,407.33 | Hederagenin or its isomer | Terpenoids | 23,84 | 465-99-6 |
| 667 | 25.49 | | 319.2405 | [M-H]- | | C_20_H_32_O_3_ | | -3.74 | | 87.38 | 319.22,275.24 | Ginkgoneolic acid or its isomer | Phenolics | 1,9,20,25 | 20261-38-5 |
| 668 | 25.80 | | 471.3543 | [M-H]- | | C_30_H_48_O_4_ | | 1.97 | | 94.23 | 471.34,407.33 | Echinocystic acid or its isomer | Terpenoids | 23,84,97 | 510-30-5 |
| 669 | 25.93 | | 469.3432 | [M-H]- | | C_30_H_46_O_4_ | | 0.58 | | 94.03 | 469.34,407.33,391.30,375.27 | 11-Keto-beta-boswellic acid | Terpenoids | 23,70,84,86,97 | 17019-92-0 |
| 670 | 26.01 | | 301.2262 | [M-H]- | | C_20_H_30_O_2_ | | -5.20 | | 77.87 | 301.21,283.21,257.22 | Kaurenoic acid | Terpenoids | 23,99 | 6730-83-2 |
| 671 | 26.55 | | 277.2251 | [M-H]- | | C_18_H_30_O_2_ | | -1.80 | | 91.85 | 277.21,259.20 | α-Linolenic acid | Fatty acid | 16,17,21,23,30,41,42,47,48,52,54,55,56,57,60,62,64,65,67,70,75,76,81,84,86,92,93,94,95,96,98 | 463-40-1 |
| 672 | 26.75 | | 454.3532 | [M-H]- | | C_30_H_48_O_3_ | | -0.05 | | 91.95 | 455.34,377.32 | β-Boswellic acid | Terpenoids | 5,8,17,23,24,25,28,30,32,47,52,56,60,67,71,77,79,85,86,91,95 | 631-69-6 |
| 673 | 27.69 | 279.2405 | | | [M-H]- | | C_18_H_32_O_2_ | | 0.95 | 89.12 | 280.23,262.22 | Linoleic acid | Fatty acid | 3,4,8,9,10,11,16,17,18,20,21,23,23,27,28,29,30,31,34,36,40,41,42,45,47,48,49,52,54,55,56,57,58,59,60,62,64,65,66,67,68,69,70,71,72,73,74,75,76,77,79,81,82,83,84,86,87,88,91,92,93,94,95,96,97,98,99 | 60-33-3 |

ESI-: Electrospray Ionization Negative Mode
